# NHC-Ni(II)-catalyzed cyclopropene-isocyanide [5 + 1] benzannulation

**DOI:** 10.1038/s41467-022-31896-y

**Published:** 2022-07-16

**Authors:** Jian–Qiang Huang, Meng Yu, Xuefeng Yong, Chun–Yu Ho

**Affiliations:** 1 Guangdong Provincial Key Laboratory of Catalysis, Southern University of Science and Technology, Shenzhen, 518055 China; 2 Shenzhen Grubbs Institute, Southern University of Science and Technology, Shenzhen, 518055 China; 3 Department of Chemistry, Southern University of Science and Technology, Shenzhen, 518055 China

**Keywords:** Homogeneous catalysis, Synthetic chemistry methodology, Synthetic chemistry methodology

## Abstract

Isocyanides are common compounds in fine and bulk chemical syntheses. However, the direct addition of isocyanide to simple unactivated cyclopropene via transition metal catalysis is challenging. Most of the current approaches focus on 1,1-insertion of isocyanide to M-R or nucleophilc insertion. That is often complicated by the competitive homo-oligomerization reactivity occurring at room temperature, such as isocyanide 1,1-insertion by Ni(II). Here we show a (N-heterocyclic carbene)Ni(II) catalyst that enables cyclopropene-isocyanide [5 + 1] benzannulation. As shown in the broad substrate scope and a [*trans-*(N-heterocyclic carbene)Ni(isocyanide)Br_2_] crystal structure, the desired cross-reactivity is cooperatively controlled by the high reactivity of the cyclopropene, the sterically bulky N-heterocyclic carbene, and the strong coordination ability of the isocyanide. This direct addition strategy offers aromatic amine derivatives and complements the Dötz benzannulation and Semmelhack/Wulff 1,4-hydroquinone synthesis. Several sterically bulky, fused, and multi-substituted anilines and unsymmetric functionalized spiro-ring structures are prepared from those easily accessible starting materials expediently.

## Abstract (web summary)

The direct addition of isocyanides to cyclopropenes is challenging. Here, the authors report a catalytic cyclopropene-isocyanide [5 + 1] benzannulation catalyzed by an (N-heterocyclic carbene)Ni(II) complex; this method enables the preparation of fused and multi-substituted anilines and unsymmetrically functionalized spiro-ring structures.

## Introduction

**Fig. 1:**
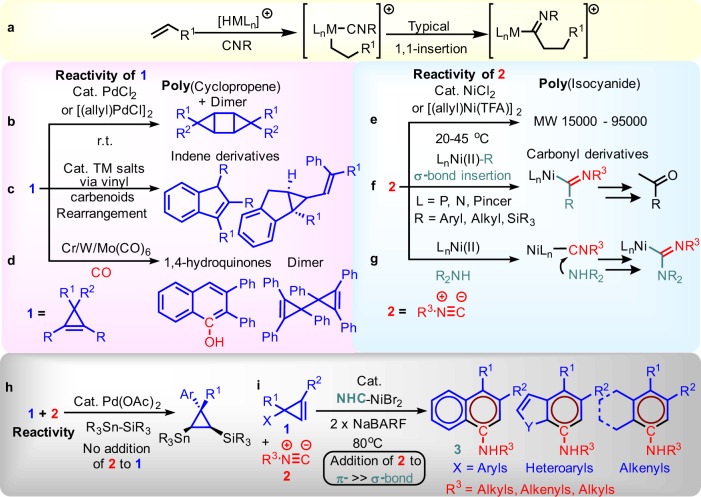
Challenges in catalytic intermolecular [5 + 1] benzannulation development by using cyclopropene and isocyanide as a substrate pair. **a** Transition-metal mediated two-steps alkene-isocyanide insertion strategy. **b** Pd(II)-catalyzed cyclopropene polymerization, and the dimerization product structure. **c** Transition-metal catalyzed cyclopropene rearrangement to indene derivatives and subsequent dimerization. **d** Semmelhack/Wulff 1,4-hydroquinones synthesis. **e** Ni-catalyzed isocyanide polymerization. **f** Isocyanide insertion to Ni(II)R bonds for carbonyl derivatives preparation. **g** Nucleophilic insertion on Ni(II) isocyanide complex. **h** Isocyanide as a ligand in Pd-catalyzed metalloid insertion to cyclopropenes. **i** (NHC)Ni(II) catalyzed [5 + 1] benzannulation by cyclopropene and isocyanide.

Alkenes and isocyanides are easily accessible primary starting materials that have often been used in both fine and bulk chemical syntheses through insertion, respectively (Fig. 1a-h)^1–8^. Their homo-dimerization and polymerization reactivity often occur readily in the presence of transition-metal salts at r.t. (Fig. 1b, c, e). Such a high homo-insertion reactivity implies that a selective cross-reaction is difficult to control. The current methods are mostly done indirectly by an in situ generated imidoyl-M species (i.e., a two-steps process)^4,8^, either through a 1,1-insertion of isocyanide to the M-R (Type 1: σ-bond insertion, Fig. 1f), such as L_n_Ni-R and L_n_Pd-R from an alkene or by a nucleophilic attack on the electrophilic isocyanide for a subsequent an alkene insertion (Type 2: nucleophilic insertion, Fig. 1g)^9–17^. There are only handful of examples that can join them together directly. Pioneering examples are those using trienes and cyclobutenes with a stoichiometric amount of Zirconocene and Titanocene complexes^18–21^, the corresponding catalytic version is still under development. Cyclopropene seems to be an obvious choice for further study, yet isocyanide just served as a ligand in metalloid insertion to cyclopropenes (Fig. 1h)^22^, and as an additive in Ni(II)/MAO catalyzed ethene polymerization at r.t^23^. Recently, cationic (NHC)Ni(II) has been developed as an efficient and selective catalyst for insertion and cyclopropene rearrangement^24–28^. This prompted us to investigate the (NHC)Ni(II) potential in directing a cross-reaction between a cyclopropene **1** and an isocyanide **2**.

In this work, instead of developing more practical imidoyl-M generation methods, we explore an alternate strategy that focuses on a formal addition of those two substrates in the absence of an additional component at the end. Here we show a catalytic intermolecular isocyanide-cyclopropene [5 + 1] benzannulation by NHC/NiBr_2_DME/NaBARF (1:1:2)^29–36^. This work is the rare C-C forming reaction between electronic neutral cyclopropene and isocyanide (Fig. 1i). It generally provides ring-expanding rather than the ring-opening products reported in the early 70 s’ that relies on activated cyclopropene^37^. In sharp contrast to those highly competitive homo reactivities of **1** or **2** as shown in Fig. 1^26,32,38–44^, a (hetero)fused aromatic amine product **3** is obtained. That serves as an aza-synthetic alternative to the Dötz benzannulation formed by metal carbene and alkyne, the Semmelhack/Wulff 1,4-hydroquinones synthesis mediated by Cr/Mo/W(CO)_n_ (Fig. 1d), as well as the catalytic [2 + 2 + 2]/[4 + 2] cycloadditions of π-systems^45–49^. This finding also complements the vinyl cyclopropanes [5 + 1] reactions reported recently as well as those imidoyl insertions reactivities^50–52^. Unlike several other intramolecular ring-expanding strategies based on **1** for the syntheses of phenols and saturated N-cycles (e.g., cycloisomerizations of **1** bearing 3,3-dicarbonyl^53^ and [4 + 3] cycloadditions of **1** bearing N-heteroaromatics^54^, respectively), our intermolecular strategy offers fused anilines and endocyclic dienes. Overall, it signifies an exciting catalytic synthesis of aromatic products with a broad substrate scope from two structurally diverse and readily accessible substrates.

## Results and discussion

### Catalyst development and optimization

**Fig. 2:**
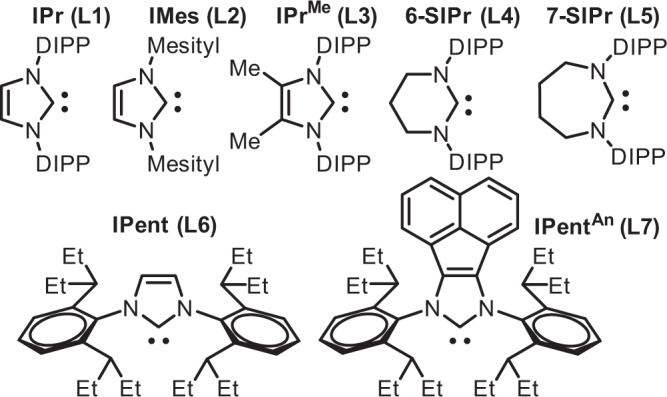
NHC structures employed in this work. NHC **L1-7**. DIPP 2,6-diisopropylphenyl.

Inspired partly by the Ni-catalyzed alkene cyanation^55^, and the high structural similarities between the Ni(II)isocyanide and the L.A. activated Ni(II)CN (L.A. = Al, B)^56,57^, we surmised that a cyclopropene may undergo an isocyanide addition by a cationic (NHC)Ni(II) under appropriate condition. We commenced our investigation by using **1a** and **2a** as a substrate pair and the **L1**/NiBr_2_DME/NaBARF (in 1:1:2 ratio, See Fig. 2 for **L** structure) as the catalyst in toluene for 12 hrs at 80 °C (Table 1, entry 1). Unlike the cross-hydroalkenylation rearrangement reactivity of **1a** and alkyne that we anticipated earlier^26^, an aromatic amine **3aa** was obtained unexpectedly in ~15% yield rather than other possible acyclic products. The structure of **3aa** was confirmed by crystallography, and it highly resembles a 1,4-hydroquinone derived from an LM(CO)_n_ mediated cyclopropene-CO coupling process.

**Table 1:** Screening NHC for Ni(II) catalyzed [5 + 1] benzannulation of 1 and 2.


Entry^a^	NHC	2	Conversion 1a^b^	Yield of 3	3:4a
1	**L1**	**2a**	60%	15%	>95:5
2^c^	**L1**	**2b**	>95%; 82%; 85%	48%; 49%; 43%	>95:5; >95:5; 91:9
3^d^			10%	-	-
4^e^			40%	30%	95:5
5 ^f^			20%	5%	-
6 ^g^	**L1**	**2b**	<5%	-	-
7 ^h^	-		<5%	-	-
8^i^	**-**		<5%	-	-
9	**-**		>95%	-	-
10	**L2**	**2b**	>95%	41%	>95:5
11	**L3**		>95%	49%	>95:5
12	**L4**		85%	53%	>95:5
13	**L5**		>95%	49%	>95:5
14	**L6**		>95%	57%	>95:5
15	**L7**		>95%	58%	>95:5
16 ^j^	**L7**	**2b**	>95%	89%	>95:5
17^j,k^		**2b**	>95%	38%	>95:5
18		**2a**	90%	35%	>95:5
19^j^		**2a**	>95%	68%	>95:5
20^j,l^	**L7**	**2b**	90%	61%	>95:5
21^j,m^		**-**	>95%	-	-

^a^ Substrate (cyclopropene **1a**: isocyanide **2** = 1: 2) was added to a mixture of 5 mol% [NHC/NiBr_2_DME/NaBARF] (0.025 mmol, in 1:1:2 ratio) catalyst in toluene and stirred for 12 h at 80 ^o^C. **4a** is a mixture of **1a** dimers and was assigned based on GCMS.

^b^Yield of product **3** and selectivity were determined by ^1^H NMR.

^c^NiBr2/NiCl2/NiI2 were used, respectively.

^d^By the following **L1**Ni(0) sources generated in situ from NHC **L1** + Ni(cod)_2_, **L1** + Ni(methyl methacrylate)_2_, **L1** + NiBr2/EtMgBr, and isolated **L1**Ni(**2a**)_3_.

^e^At 40 °C.

^f^No NaBARF.

^g–i^Only IPr; Only NaBARF; No catalyst, respectively. All in the absence of Ni at 80 ^o^C for 5 h, <10% **2b** conversion.

^j^10 mol% catalyst.

^k^L:Ni:NaBARF = 1:1:1.

^l^With 10 mol% TEMPO.

^m^1 was converted to gel in 10 min.

**Fig. 3:**
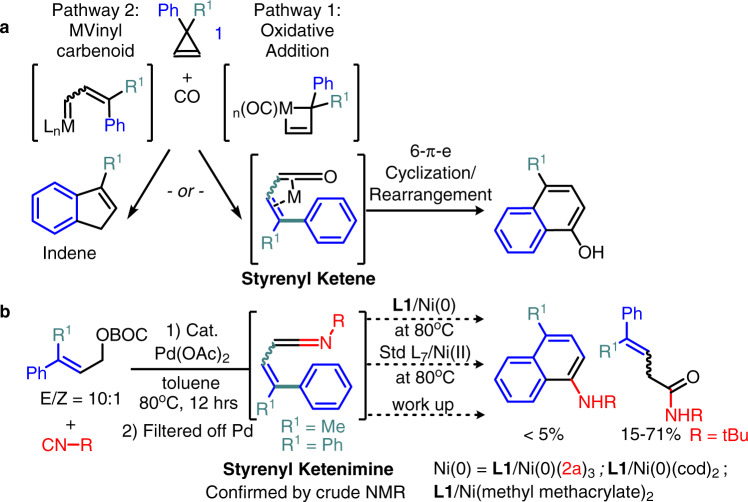
Styrenyl ketene/ketenimine as an intermediate for 6-π electron cyclization. **a** Dötz Benzannulation and Semmelhack/Wulff synthesis. **b** 6-π electron cyclization attempts via styrenyl ketenimine.

One might envision the [5 + 1] benzannulation of **1** and **2** involved a 6-π electron styrenyl ketenimine rearrangement process^36^ (c.f. Figs. 1d, 3a, typical operation temp: ~120–160 °C bearing electronic activators)^58–61^ instead of an isocyanide addition to an alkene. This is because a relevant styrenyl ketene intermediate can be obtained from **1** by an M(0) oxidative addition^62–65^ and/or acidic metal salts directed rearrangement to the vinylcarbenoid (then Type 3 isocyanide insertion to M(carbene)). So we decided to determine the active components and the catalyst oxidation state that are required for the desired reactivity before optimization. Interestingly, subjecting the independently synthesized styrenyl ketenimines to the (NHC)Ni(0)/(II) at 80 °C conditions did not offer the [5 + 1] benzannulation (Fig. 3b), and most of the ketenimine was hydrolyzed to amide after 12 hrs. Also, aromatic isocyanides (**2a**, **2b**, and **2g**, see structure later) did not participate in that reaction in either toluene at 80 °C or refluxing THF for 12 hrs. Indeed, the [5 + 1] benzannulation attempts were unproductive by various (NHC)Ni(0) species, like **L1**/Ni(methyl methacrylate)_2_^66^, **L1**/Ni(cod)_2_, isolated **L1**Ni(**2a**)_3_^67^, and in situ Ni(II) reduction^68^(Table 1, entry 3). Moreover, common cyclopropene rearrangement products derived from vinylcarbenoid^44^, S_E_Ar^43,69^, σ-bond metathesis or oxidative addition mechanisms, like the 1-methyl indene and indane derivatives **4a′** were not observed as well (Fig. 1c)^68,70–73^. The major side products obtained from **1** is a mixture of dimer **4a** and oligomerization, which contrasts to the M(vinylcarbenoid) mechanism predicted and is similar to the Pd(II) directed dimerization reactivity of **1** at r.t. (Fig. 1b). In other words, all the above results do not fit the rationale based on a typical 6-π electron styrenyl ketene rearrangement pathway. Thus, the similar performances of the Ni(II)halides were not caused by an unexpected reduction of Ni(II) to Ni(0) (entry 2). And it is not surprising to see a reasonably good reactivity by a **L1**Ni(II) at 40 °C, which is supposed to be a less favorable temperature for an oxidative cyclopropene-opening (entry 4 vs Fig. 3).

Control experiments identified that the NHC, the Ni(II) salts, and the NaBArF are all crucial components for the desired reactivity (entry 5-9). In particular, the NaBArF was proved essential for a high catalyst performance (entry 2 vs 5) like the typical Ni(II) catalyzed alkene insertions^74^. Meanwhile, the NHC steric optimization showed an increase in yield and selectivity of **3** (entry 2, 10–15, Fig. 2, **L1** vs **L2-3**, **L6** vs **L7**). A good balance of desired reactivity and yield was achieved by **L7**-NiBr_2_ and NaBARF in a 1:2 ratio (entry 16–17), and this catalyst seems promising to cover smaller isocyanides (entry 1 vs 2, 15 vs 18, and 16 vs 19). Such progress was attributed mainly to an optimal steric repulsion among the NHC-Ni(II) and **2**, whereby the strong coordination ability of **2** on cationic (NHC)Ni(II) was fine-tuned, and the desired reactivity of **1** was maintained (**L1-7**). Thus, a cationic Ni(II) catalyst free of NHC could not provide **3ab** from **1a** and bulky **2b** (entry 9). Ran the reaction in the presence of TEMPO did not result in a significant drop in yield (entry 20, 1:1 to Ni(II)) while using a (IPr)Ni(I)Cl dimer as a catalyst in chlorobenzene only gave <5% desired product (no matter with NaBARF or not, See SI), both results suggesting that Ni(I) was not the active catalyst. By using our optimized catalyst under forcing conditions (i.e., without **2**), the undesired conversion of **1a** was dominated by oligomerization, and dimer **4a** was still observed (entry 21)^75^. The [5 + 1] reaction can be done on a larger scale under a slightly modified condition (5 mmol), 1.28 g of **3ab** was obtained successfully (81% yield of **3ab**, **3**:**4** > 95/5, see SI).

### Scope of the (NHC)Ni(II) catalyzed [5 + 1] benzannulation

**Fig. 4:**
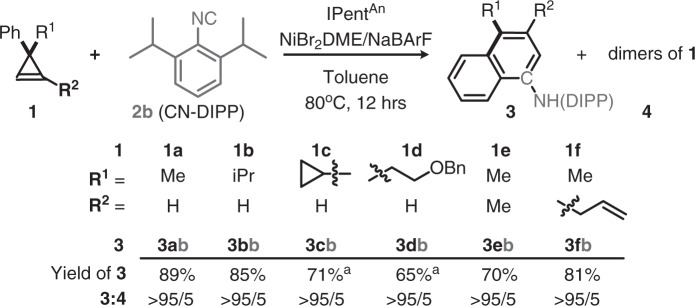
Scope of cyclopropene substituents at R^1^ and R^2^. Standard condition was followed: IPent^An^ **L7** (0.05 mmol), NiBr_2_DME, NaBARF = 1:1:2, cyclopropene **1**: isocyanide **2** = 1:2 (0.5 and 1.0 mmol), at 80 ^o^C in 2 mL toluene for 12 h. Products were characterized by NMR after isolation. Ratio was determined by ^1^H NMR. Superscript ^a^ indicates 2 mmol of **2** was used.

With the above basic information in mind, we decided to study the substrate scope and gain mechanistic insight accordingly. First, the scope of **1** was tested by **2b** as a substrate pair and **L7**/NiBr_2_/NaBArF as a catalyst in toluene at 80 °C for 12 h. To our delight, the scope of R^1^ and R^2^ on **1** are broad (Fig. 4), it offers an exemplary method for making a fused aromatic aniline **3** with various side chains at the p- and m-positions (**3ab**-**3fb**). It covers linear/branch alkyls, functional groups vulnerable to oxidative addition, and radical and nucleophile **2b** are compatible (e.g., cyclopropyl and benzyl ether). When a cyclopropene bearing a trisubstituted olefin, the ring-opening event occurs regioselectively, in which only a m- over o-substituted naphthamine regioisomers (**3eb** and **3fb**) was obtained from **1e-f** (Trisubstituted olefin with R^1^ = H, R^2^ = nBu, Aryl = p-C_6_H_4_CF_3_ could be used as a pair with **2b** at ~120 degree and 20 mol% catalyst loading (see SI, 58% yield, r.r. >95:5)). Besides, ring-strain relief was found as a key factor that governs the reaction^76,77^. Terminal alkene near the cyclopropene did not interfere with the desired reactivity and no new cyclopropane was formed there (**3fb**), suggesting that the postulated Ni(vinylcarbenoid) commonly found in Ni(II) reaction with cyclopropene is not an active intermediate in this reaction again.

**Fig. 5:**
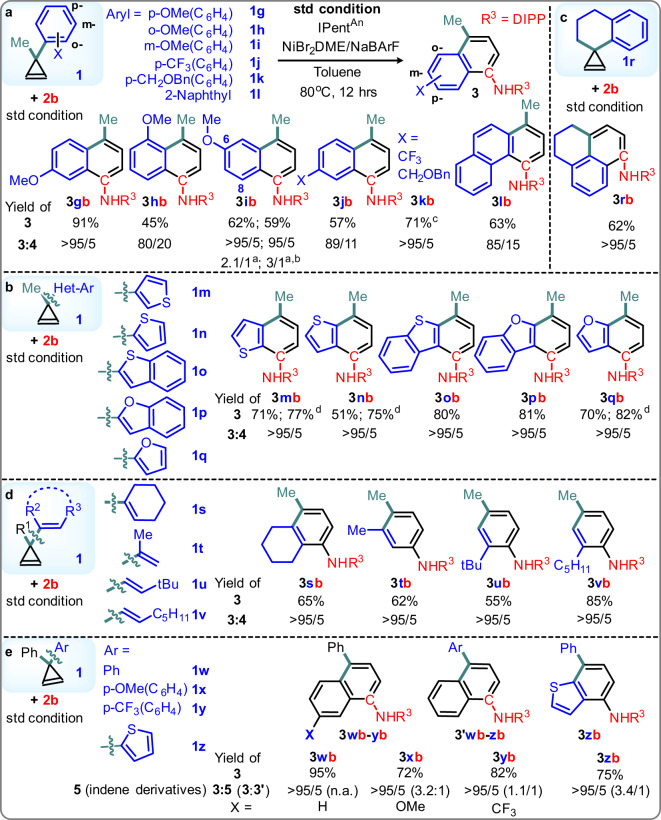
Scope of cyclopropene substituents at 3-position. Standard condition was followed. Ratio was determined by 1H NMR. **a** Aryls; **b** Heteroaryls; **c** Spiro; **d** Alkenyl; **e** Diaryls examples. Superscript ^a^ Ratio of 6-:8-OMe regioisomers. ^b^ at 60 ^o^C. ^c^ 2 mmol of **2**. ^d^ 0.5 mmol of **2** was added after 0.5 and 1 h.

Such a [5 + 1] reactivity is not limited to **1** bearing an unsubstituted Ph only. Other electronic activated phenyls (**1g**-**k**), naphthyl (**1** **l**), and heteroaryls (**1m**-**q**) are all possible substrates in this catalysis (Fig. 5a–c). Electronically activated naphthamines at 5-, 6-, 7-positions, and (hetero)aryl-fused anilines^78^ with different relative positions to the NHR were prepared, respectively. This method represents a general route to build several medicinally^79–88^ and photochemically^89–91^ important cores bearing different activators and features^92^. Notably, **1r** bearing a spiro ring was utilized successfully^93^. That opened up a route to prepare amine-functionalized 2,3-dihydro-1H-phenalenes, a common motif in OLEDs. After a closer look at the results, aromatic substituents on **1** which can donate a higher electron density to the cyclopropene 3-position are more effective substrates. This finding inspired us to consider a cyclopropane ring-strain relief mechanism by the donor-acceptor push-pull reactivity of 1,2-disubstituted cyclopropanes reported in the literature^30^, in which the isocyanide serves as acceptor and the aryl at 3-position serves as a donor (see discussion later).

The above results in Fig. 4 and Fig. 5a–c implies that the benzannulation scope is not limited to cyclopropene substituted with an aryl. Other electron-donors on the cyclopropene Csp^3^ (3-position) that may form the proposed 1,2-disubstituted cyclopropane for the donor-acceptor push-pull reactivity should be a possible substrate of this reaction. Cyclopropenes with simple alkenyls and unsymmetric diaryls at the 3-position were tested next (Fig. 5d, e, **1s**-**z**), assuming the isocyanide addition selectivity still follows the olefin ring-strain, the vinylcarbenoid formation remains slow, and the isocyanide addition is still accessible even when both the upper and lower sides of 3-position are sterically shielded heavily. Indeed, those substrates followed most of the above assumptions, revealed a route that can prepare alkyl-substituted anilines from non-aromatic cyclopropenes, and **3** substituted with different p-aryl substituents. In particular, cyclic and acyclic alkenyl groups with different substitution patterns are all compatible, 2,4-/3,4-dialkyl substituted anilines from isocyanide directly. Again, no cyclopropanation was detected here, in which a cyclohexenyl group was used to make an aniline fused with cycloalkyl structure (**3** **sb**) and indicated the isocyanide addition selectivity was not simply favored by a cyclic olefin. Up to 95% yield and reasonable π-system selectivity was observed in diaryl examples, despite the steric challenges is high and the electronic differences being moderate. However, the desired product was obtained with a small amount of indene derivative **5** (**3**:**5** > 95/5). This change in side product preference follows the typical cyclopropene reactivity trend reported in vinylcarbenoid formation literature.

**Fig. 6:**
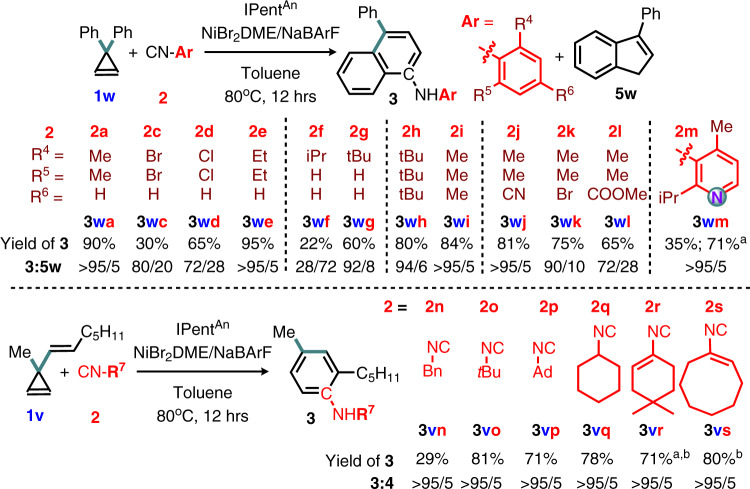
Scope of aryl, alkenyl, and alkyl-substituted isocyanides. Standard condition was followed. Superscript ^a^ 2 mmol of **2**. ^b^ Partial hydrolysis of the enamine was observed. Yield was determined after complete hydrolysis by stirring in MeOH/H_2_O.

Isocyanide scope exploration showed that an optimal steric interaction between the substrate and the NHC is one of the keys for achieving the desired reactivity and selectivity (Fig. 6). The **L7** can manage isocyanides with distinct and challenging structural characteristics under our standard conditions. The substituent is not limited to simple unfunctionalized phenyl groups, many sterically challenging and electronically diverse groups like unsymmetric and symmetric (hetero)aryls, alkenyls, and alkyls are also compatible. Functional groups sensitive to oxidative and nucleophilic additions in traditional Ni(0)-catalyzed C-N coupling for aromatic amine synthesis, like halides, ester, and nitrile (**2c-d** and **2j-l**), could be used directly without protection. Those groups can serve as versatile handles for reduction and cross-coupling whenever necessary. Undesired reactions like oligomerization of **2** and formation of **5w** are more obvious in some less effective pairs. Yet, a higher concentration of **2** improved the yield (e.g., **3wm**), suggesting a high concentration of **2** favors the desired reactivity more than the undesired oligomerization. No amidine and no guanidine product from **2** and **3** was observed in all cases examined, including the sterically less bulky and more electron-rich N-alkyl-N-aryl secondary amines. Thus, both the NH on the N,N-disubstituted amine **3** and the Br are unlikely nucleophilic enough to form an amidinyl and an imidoyl species as the reaction intermediate (c.f. the Type 2 isocyanide insertion).

**Fig. 7:**
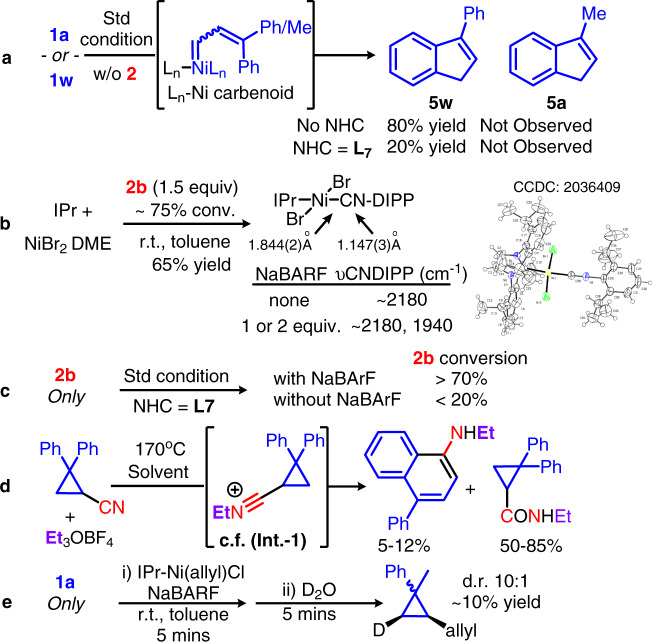
Mechanistic studies and reaction models. **a** Effect of NHC and the three-substituent on indene derivative formation; **b** [*trans*-IPr-NiBr_2_(CNDIPP)] crystal structure, IR studies, and NaBArF effect; **c** NHC effect on **2b** oligomerization; **d** A cyclopropyl carbonitrile alkylation directed rearrangement; **e** Syn-addition of a cationic (NHC)Ni(II) species to cyclopropene.

Other than the hints offered by the scope exploration and the relevant literature, several additional experiments were carried out to gain mechanistic insight into the reaction (Fig. 7). First, the **5** formation might be caused by the decomposed catalyst at an elevated temperature, since a parallel set of control experiments showed that the **5w** was formed mainly by a NiBr_2_DME/NaBARF catalyst free of NHC **L7** (Fig. 7a). This result indicated a strong NHC coordination to a Ni(II) center is important, it could suppress the 3,3-diaryl substituted cyclopropene rearrangement to the corresponding Ni(vinylcarbenoid). Second, we isolated the isocyanide complex (Fig. 7b). It was prepared in an excess amount of **2b**, but the spectroscopic information obtained from the crystal structure revealed a trans-configuration between the NHC and the isocyanide in a 1:1 ratio, and the axial positions are shielded sterically by the isocyanide and the NHC substituents. This complex implied that the NHC can suppress the simultaneous coordination of two isocyanides. Thus, the oligomerization of isocyanide was not observed and manipulations like a slow addition of **2** in a large excess amount are unnecessary for most of our [5 + 1] reactions. Indeed, the typical oligomerization reactivity of **2**, which can be occurred at r.t. by a number of metal salts, is now suppressed appreciably even for 12 h at 80 °C in the presence of NaBArF when an NHC is employed (Fig. 7c). Next, an IR shift from 2180 to 1940 cm^−1^ was observed by treating the [*trans*-NHC-NiBr_2_(**2b**)] precatalyst with NaBARF (Fig. 7b). This shift is in agreement with an increase in Ni-CNR backbonding character of a cationic species, rather than the NHC/P-Ni(II)(η^1^-/η^2^-N-aryliminoacyl)Br type of structures at IR range ~1730–1600 cm^−1,^^94^. The halide abstraction product is quite unstable. Some hydrolysis to amide was indicated by the peaks at ~ 3100, 1850, and 1800 cm^−1^ region in less than 10 min. No metathesis type of reactivity was observed in the typical [5 + 1] reaction condition. Using two equivalents of NaBArF did not result in a further drop in frequency, and this result supports a mono- rather than di-cationic complex formation^95^. The extra NaBArF employed in the [5 + 1] condition might be used for a better anion exchange efficiency. Interestingly, a 4-phenyl-1-naphthamine derivative, which is similar to our [5 + 1] benzannulation product structure, was obtained successfully from a cyclopropyl carbonitrile alkylation (Fig. 7d)^76^. This result suggests the donor-acceptor push-pull cyclopropane opening mechanism is plausible for an aryl and CNR substituted cyclopropane as Fig. 5a–c suggested, and the isocyanide addition to the olefin on the cyclopropene is likely involved. Trapping the intermediate from a reaction between **1a** and a cationic [(NHC)Ni(allyl)]BArF with D_2_O showed a D-labeled allylcyclopropane with syn-configuration. This result suggests a cyclopropene can undergo insertion like the other alkenes with (NHC)Ni(II), and it may be one of the possible pathways for introducing the (NHC)Ni(II)-CNR (1.844(2) Å from Fig. 7b) to the cyclopropene.

**Fig. 8:**
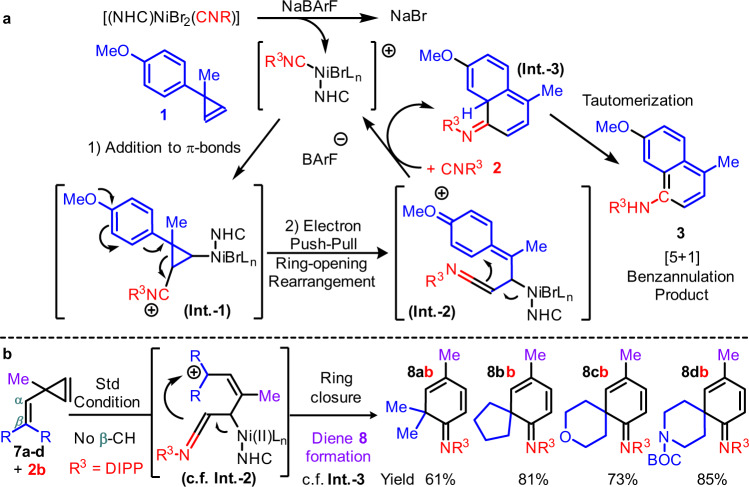
Working hypothesis of the cyclopropene and isocyanide [5 + 1] reactions by a (NHC)Ni(II) catalyst. **a** Benzannulation. **b** 1,3-Diene substituted spiro-rings. Postulated structures are in brackets, and the anion is omitted for clarity.

At this stage, the keys for the desired [5 + 1] reactivity and broad scope are attributed mainly to the high reactivity of an isocyanide addition to the cyclopropene π-bond by a cationic NHC-Ni(II) catalyst, and the high rearrangement reactivity after that (Fig. 8a). By the optimal coordination ability and steric effect from NHC, isocyanide and cyclopropene combinations, other typical pathways related to the acidic metal salt directed vinylcarbenoid formation and oligomerization are suppressed accordingly. One of the possible reaction sequences may involve a cationic (NHC)Ni(II) directed addition of **2** to **1**, similar to P-Pd(II)acetylide insertions to alkenes and resemble hydro-/alkyl-nickelation to **1** (Fig. 1a, 3)^32,96,97^. It may be assisted by (a) an optimal steric repulsion between NHC and **2** (otherwise NHC-Ni(II)(η^2^-iminoacyl) complex can be formed at −35 °C by isocyanide 1,1-insertion), (b) a halide abstraction by NaBArF^14,98–100^, and (c) a lowered vinylcarbenoid formation and oligomerization reactivity of **1** in the presence of strongly coordinating **2** and NHC (Table 1, entry 21)^101^. Next, a ring-strain relief triggered dearomatization may occur (Int.-1)^76,77^, similar to the donor-acceptor push-pull reactivity of 1,2-disubstituted cyclopropanes^30^. The substituent repulsions between the bulky **L7** and the cyclic tether from **1** may restrict the Int.-2 conformation. Hence, the ketenimine and the cyclic tethers were aligned on the same side, and the competing styrenyl ketenimine formation pathway was suppressed (See SI for details). Since the postulated Int.-2 has no β-H available for the Ni(II), ligand exchange with another **2** may facilitate the ring closure (Int.-3) and regenerate the catalyst. After a facile re-aromatization over imine hydrolysis, those highly substituted **3** can be formed.

Based on the working hypothesis, a catalytic [5 + 1] endocyclic diene synthesis was also achieved (Fig. 8b, 8ab-db) by intercepting the postulated re-aromatization step (Int.-3 to **3**). Unsymmetrically substituted spiro-ring structures were obtained from the corresponding **7** and **2** in just a step by the standard condition (e.g., spiro[4,5]deca-dien-imine), in which heterocycles are also tolerated. Notably, by a comparison of the side chains on cyclopropene **1** and **7**, the above examples do not support a [5 + 1] mechanism initiated by an aryl side chain imidoylation (by CH activation, then 1,1-insertion of **2**). That is simply because the alkenyl side chain on **7** has no H at the β-position.

### Regioselective post-modifications

**Fig. 9:**
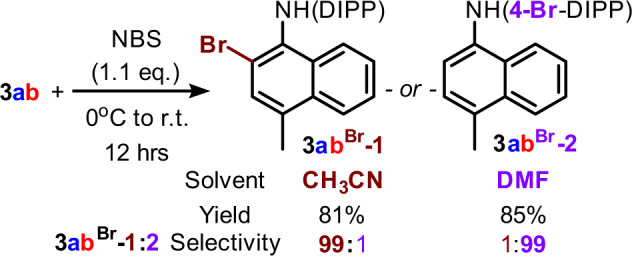
Post-modification of the product. Solvent-controlled regioselective bromination on unsymmetrically substituted diaryl amine.

Finally, the product **3** can serve as a key building block for a higher substituted aromatic amine synthesis easily. For instance, a solvent-controlled highly regioselective bromination on unsymmetric diaryl amine was achieved by simply using NBS as Br source (Fig. 9, ratio was determined by GCMS)^102,103^. These functionalized sites may serve as additional handles for preparing other related structures with similar sets of cores.

We have developed an intermolecular cyclopropene-isocyanide [5 + 1] benzannulation by a cationic NHC-Ni(II) catalyst with a BArF anion, in which those two substrates were unreactive to each other before and were dominated by their own reactivities. These combinations showed the strength of this catalyst design in broadening the use of those structurally diversified starting materials with remarkably good functional group compatibilities and regulating competitive reactivities of those two substrates at an elevated temperature. This method serves as an aza-synthetic alternative for products structurally similar to those in Dötz benzannulation and Semmelhack/Wulff synthesis based on Cr/Mo/W(CO)n as well as those in styrenyl ketene 6-π electron cyclizations. The study also provides a method to make unsymmetric and functionalized spiro-ring structures catalytically. This work has revealed several opportunities to utilize isocyanide and cyclopropene for other potential applications.

## Methods

### General procedure for the [5 + 1] Benzannulation

To a catalyst mixture (0.05 mmol **L7**/NiBr_2_DME, 0.10 mmol NaBARF) stirred in toluene (1 mL) for 3 min at 80 ^o^C, an indicated amount of a premixed toluene solution of **1** and **2** (1 mL) was added in one-pot and stirred for an additional 12 h. After cooled down to r.t., it was diluted with 6 mL nhex/EA (10:1) and filtered through a short plug of silica gel. The solvent was then removed on rotavap. Conversion of **1** and selectivity of **3** to other possible isomers were determined by ^1^H NMR or GCMS (average of two runs). Product structures were confirmed by chromatography and isolation (5–10% EA/Hex).

## Supplementary information

Supplementary Information

## Data Availability

The compound characterization data generated in this study are provided in the Supplementary Information. The data sets generated during this study are available from Cambridge Crystallographic Data Centre via www.ccdc.cam.ac.uk/data_request/cif (X-Ray crystallographic data: CCDC **3aa** is 2036407 and [*trans*-IPr-NiBr_2_(CNDIPP)] is 2036409).
