# Supplementary material for: NHC-Ni(II)-catalyzed cyclopropene-isocyanide [5 + 1] benzannulation
Source: Nat Commun. 2022 Jul 16;13:4145. doi: 10.1038/s41467-022-31896-y (PMC9288548; doi:10.1038/s41467-022-31896-y)
Supplement: Supplementary file 1 — Supplementary Information [file 41467_2022_31896_MOESM1_ESM.pdf]

Supplementary Information

NHC-Ni(II) Catalyzed Cyclopropene-Isocyanide [5+1] Benzannulation

Jian–Qiang Huang,[1,]* Meng Yu,[1,3] Xuefeng Yong,[3] Chun–Yu Ho[1-4]**

[1]Guangdong Provincial Key Laboratory of Catalysis, Southern University of Science and Technology (SUSTech), Shenzhen, China

[2]Shenzhen Grubbs Institute, Southern University of Science and Technology (SUSTech), Shenzhen, 518055, China

[3]Department of Chemistry, Southern University of Science and Technology (SUSTech), Shenzhen, 518055, China

[4]Lead contact

content

1.Supplementary Notes S2

2.Supplementary Methods S3

2.1 Substrate synthesis S3

2.2 IPent^{An} synthesis S10

2.3 General procedure for NHC-Ni(II) catalyzed cyclopropene-isocyanide [5+1] benzannulation and diene synthesis S11

2.4 Control and additional experiments S14

2.4.1 D-Labelling Experiments S14

2.4.2 Attempts to trap the concerned NHC-Ni(vinylcarbenoid) S14

2.4.3 Isolation of [*trans*-IPr-Ni(CN-DIPP)Br_2] S15

2.4.4 Attempts to use NHC-Ni(0) as catalyst for the [5+1] benzannulation S16

2.4.5 Attempts to use an in situ generated styrenyl ketenimine as a substrate for a 6-π electron cyclization to naphthamine S18

2.4.6 Synthesis of the authentic dimer of **1a** (i.e. **4a**) by $PdCl_2$ for comparison. S20

2.4.7 [5+1] reaction in the presence of TEMPO S21

2.4.8 IPr-NiCl dimer reaction S21

2.4.9 Cyclopropylcarbonitrile rearrangement by alkylation S22

2.4.10 The bulky NHC **L7** steric effect on **Int.-2** conformation S23

2.5 Solvent-controlled regio-divergent bromination of [5+1] product S25

2.6 Product characterization data and GCMS trace S26

3.Supplementary Figures S49

3.1 Crystal structures of **3aa** and [*trans*-IPr-Ni(CN-DIPP)Br_2] S49

3.2 NMR spectra S51

4.Supplementary References S148

1. Supplementary Notes

Unless otherwise indicated, all reactions were performed under a nitrogen atmosphere from which oxygen and moisture were rigidly excluded from reagents and glassware. $NiBr_2DME$, NaBARF were purchased from ACROS, J&K or TCI, stored in a glovebox and used without further purification. Toluene was distilled over sodium and CaH_2 before use. IPr, SIPr, IMes, IPent*HCl were purchased from Aldrich, TCI or Acros. Both commercially available and synthesized cyclopropene and isocyanide were dried with CaH_2 or $CaCl_2$ before use. Unless otherwise indicated, they were synthesized according to literature procedures. Analytical thin layer chromatography (TLC) was performed using EM Science silica gel 60 F254 plates. The developed chromatogram was analyzed by UV lamp (254 nm), ethanolic phosphomolybdic acid (PMA) or potassium permanganate ($KMnO_4$). Purification of product was performed by using Silica Gel (230–400 mesh, 0.04-0.063 mm) coarse fritted glass column. Desired aromatic amine products can be isolated by column chromatography on silica gel. ^{1}H and ^{13}C NMR spectra were recorded on Bruker spectrometers in $CDCl_3$ (400 or 500 MHz for ^{1}H and 100 or 125 MHz for ^{13}C) Chemical shifts in ^{1}H NMR spectra are reported in ppm on the δ scale from an internal standard of TMS. Data are reported as follows: chemical shift, multiplicity (s = singlet, d = doublet, t = triplet, q = quartet, m = multiplet, br = broad), coupling constant in hertz (Hz), and integration. Chemical shifts of ^{13}C NMR spectra are reported in ppm from the central peak of $CDCl_3$ (77.16 ppm) or C_6D_6 (128.06 ppm) on the δ scale. Yield and selectivity were determined by integration of areas of selected peaks in crude ^{1}H NMR with relaxation ime d1 = 10 seconds and Mesitylene as standard. High resolution mass spectra (HRMS) were obtained on a Finnigan MAT 95XL GC Mass Spectrometer of the Southern University of Science and Technology, China.

2. Supplementary Methods

2.1 Substrate synthesis

a) cyclopropenes synthesis

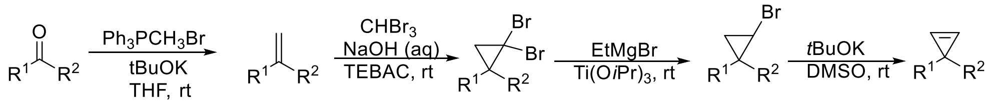

Supplementary Figure 1. Synthetic route for cyclopropene

All cyclopropane are prepared according the literature reports[1, 2], a typical procedure for preparation of cyclopropenes is described as follow:

A solution of methyl triphenylphosphonium bromide (10.7 g, 30.0 mmol) in anhydrous THF (40 mL) was cooled to 0 °C under argon, followed by addition of KOtBu (3.36 g, 30.0 mmol). The reaction mixture was stirred at 0 °C for 1 h, and then a solution of ketone (25.0 mmol) in anhydrous THF (20 mL) was added dropwise. The resulting mixture was warmed gradually to room temperature and kept stirring for 12 h. The resultant reaction solution was filtered over Celite®, and the filtrate was concentrated under reduced pressure to yield a residue which was further purified over silica gel flash column chromatography to afford olefin.

A mixture of olefin (20 mmol), TEBAC (2 mmol) and bromoform (30 mmol) was stirred vigorously with mechanical force at room temperature. A solution of concentrated NaOH in water (50 wt% NaOH) was added drop wise into the mixture. The solution turned into a dark brown emulsion while the stirring was continued for 24 h. The reaction was quenched by pouring the emulsion into 100 mL water and extracted with DCM (3x50 mL). The organic layer was separated, washed with water (3x50 mL) and brine, and concentrated under reduced pressure. The residue was purified through silica gel flash chromatography using hexanes: ethyl ether (30:1) as eluent to yield the dibromocyclopropanes.

A solution of dibromocyclopropane (15 mmol) in 60 mL anhydrous Et_2O containing 10 mol% $Ti(OiPr)_4$ was treated with 3M ethyl magnesium bromide solution in Et_2O drop wise (5.4 ml, 16.2 mmol) under argon at 0 °C, and the mixture was stirred continuously under argon at room temperature. The reaction was monitored by GCMS until the reaction was finished, and then the reaction was quenched with aqueous NH_4Cl, extracted with Et_2O, dried over anhydrous Na_2SO_4, and evaporated to dryness in vacuum. The residue was purified through flash chromatography to give the desired monobromocyclopropanes product.

t-BuOK (13 mol) was stirred in anhyd DMSO (15 mL) at room temperature under argon until the solution became homogenous, then solution of bromocyclopropane (10 mmol) in DMSO (10 ml) was added dropwise to the solution. The mixture was stirred for ~5 h at room temperature, then poured into ice-cold H_2O and extracted with Et_2O. The combined organic phases were washed with brine, dried and concentrated. The residue was purified through flash chromatography or distillation (**1t**, **7a**) to give the desired cyclopropene product. **1a**[1], **1b**[3], **1c**[4], **1e**[5], **1f**[5], **1g**[1], **1h**[1], **1i**[3], **1j**[1], **1l**[6], **1n**[3], **1q**[3], **1r**[6], **1w**[7] are known compounds and charactered in the literature.

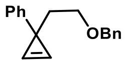 1d

Chemical Formula: $C_{18}H_{18}O$ Light yellow liquid, 30% yield over 3 steps.

^{1}H NMR (500 MHz, $CDCl_3$) δ: 7.40-7.16 (m, 12H), 4.49 (s, 2H), 3.47 (t, *J* = 7.0 Hz, 2H), 2.37 (t, *J* = 7.0 Hz, 2H); **^{13}C NMR** (125 MHz, $CDCl_3$) δ: 148.8, 138.7, 128.5, 128.1, 127.7, 127.6, 126.4, 125.4, 113.7, 73.1, 68.5, 35.8, 24.6; **HRMS(EI-MS)**: calculated $C_{18}H_{19}O$: 251.1430 (M+H), found: 251.1425.

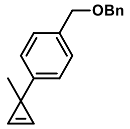 1K

Chemical Formula: $C_{18}H_{18}O$ Light yellow liquid, 40% yield over 3 steps.

^{1}H NMR (400 MHz, $CDCl_3$) δ: 7.45-7.20 (m, 11H), 4.59 (s, 2H), 4.59 (s, 2H), 1.68 (s, 3H); **^{13}C NMR** (100 MHz, $CDCl_3$) δ: 149.6, 138.5, 134.9, 128.5, 127.9, 127.9, 127.6, 126.2, 115.6, 72.0, 71.9, 25.6, 21.8; **HRMS(EI-MS)**: calculated $C_{18}H_{19}O$: 251.1430 (M+H), found: 251.1426.

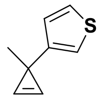 1m

Chemical Formula: C_8H_8S Light yellow liquid, 28% yield over 3 steps.

^{1}H NMR (400 MHz, $CDCl_3$) δ: 7.28 (s, 2H), 7.17 (dd, *J* = 2.4, 4.8 Hz, 1H), 6.88 (dd, *J* = 1.6, 3.2 Hz, 1H), 6.77 (dd, *J* = 1.2, 4.8 Hz, 1H), 1.59 (s, 3H); **^{13}C NMR** (100 MHz, $CDCl_3$) δ: 153.2, 126.9, 124.7, 118.6, 116.7, 25.9, 20.0; **HRMS(EI-MS)**: calculated C_8H_9S: 137.0420 (M+H), found: 137.0417.

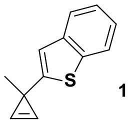 1o

Chemical Formula: $C_{12}H_{10}S$ Light yellow liquid, 18% yield over 3 steps.

^{1}H NMR (500 MHz, $CDCl_3$) δ: 7.71 (dd, *J* = 0.5, 7.5 Hz, 1H), 7.63 (dt, *J* = 0.5, 8.0 Hz, 1H), 7.35 (s, 2H), 7.31-7.26 (m, 1H), 7.23-7.18 (m, 1H), 7.00 (s, 1H), 1.69 (s, 3H); **^{13}C NMR** (125 MHz, $CDCl_3$) δ: 159.9, 141.4, 139.0, 124.2, 123.2, 122.6, 122.1, 118.8, 116.3, 25.2, 21.3; **HRMS(EI-MS)**: calculated $C_{12}H_{11}S$: 187.0576 (M+H), found: 187.0574.

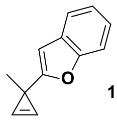

1p

Chemical Formula: $C_{12}H_{10}O$ Light yellow liquid, 30% yield over 3 steps.

^{1}H NMR (500 MHz, $CDCl_3$) δ: 7.47-7.42 (m, 1H), 7.39-7.33 (m, 1H), 7.30 (s, 2H), 7.17-7.12 (m, 2H), 6.35 (d, J = 1.0 Hz, 1H), 1.60 (s, 3H); **^{13}C NMR** (125 MHz, $CDCl_3$) δ: 165.4, 154.3, 129.7, 122.9, 122.5, 120.1, 114.2, 110.8, 101.0, 23.7, 18.4; **HRMS(EI-MS)**: calculated $C_{12}H_{11}O$: 171.0804 (M+H), found: 171.0802.

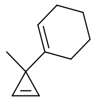

1s

Chemical Formula: $C_{10}H_{14}$ Colorless liquid, 18% yield over 3 steps.

^{1}H NMR (400 MHz, $CDCl_3$) δ: 7.38 (s, 2H), 5.56 (s, 1H), 2.10-2.03 (m, 2H), 1.66-1.63 (m, 2H), 1.60-1.50 (m, 4H), 1.33 (s, 3H); **^{13}C NMR** (100 MHz, $CDCl_3$) δ: 144.2, 120.6, 119.6, 27.8, 25.7, 25.5, 24.4, 23.5, 22.6; **HRMS(EI-MS)**: calculated $C_{10}H_{15}$: 135.1168 (M+H), found:135.1166.

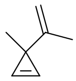

1t

Chemical Formula: C_7H_{10} Colorless liquid, 35% yield over 3 steps.

^{1}H NMR (400 MHz, $CDCl_3$) δ: 7.36 (s, 2H), 4.85 (s, 1H), 4.76 (s, 1H), 1.48 (s, 3H), 1.34 (s, 3H); **^{13}C NMR** (100 MHz, $CDCl_3$) δ: 153.0, 119.5, 109.1, 31.4, 25.4, 22.7; **HRMS(EI-MS)**: calculated C_7H_{11}: 95.0855 (M+H), found: 95.0859.

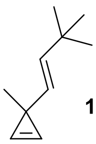

1u

Chemical Formula: $C_{10}H_{16}$ Colorless liquid, 18% yield over 3 steps.

^{1}H NMR (500 MHz, $CDCl_3$) δ: 7.34 (s, 2H), 5.31 (d, J = 16.0 Hz, 1H), 5.15 (d, J = 15.6 Hz, 1H), 1.28 (s, 3H), 0.99 (s, 9H); **^{13}C NMR** (125 MHz, $CDCl_3$) δ: 139.4, 135.9, 120.1, 32.8, 30.1, 25.1, 20.9; **HRMS(EI-MS)**: calculated $C_{10}H_{17}$: 137.1225 (M+H), found: 137.1323.

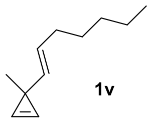

1v

Chemical Formula: $C_{11}H_{18}$ Colorless liquid, 41% yield over 3 steps.

^{1}H NMR (400 MHz, $CDCl_3$) δ: 7.33 (s, 2H), 5.31-5.25 (m, 2H), 2.03-1.97 (m, 2H), 1.38-1.25 (m,

9H), 0.88 (t, J = 6.8 Hz, 3H); **^{13}C NMR** (100 MHz, $CDCl_3$) δ: 141.3, 128.3, 120.0, 32.8, 31.7, 29.7, 25.0, 22.7, 21.0, 14.2; **HRMS(EI-MS)**: calculated $C_{11}H_{19}$: 151.1481 (M+H), found: 151.1483.

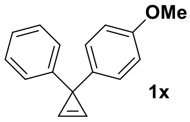

1x

Chemical Formula: $C_{16}H_{14}O$ Light yellow liquid, 35% yield over 3 steps.

^{1}H NMR (400 MHz, $CDCl_3$) δ: 7.48 (s, 2H), 7.30-7.23 (m, 2H), 7.20-7.15 (m, 3H), 7.13-7.07 (m, 2H), 6.85-6.78 (m, 2H), 3,77 (s, 3H); **^{13}C NMR** (100 MHz, $CDCl_3$) δ: 157.9, 147.6, 139.4, 129.2, 128.2, 128.1, 125.7, 113.7, 113.7, 55.4, 31.3; **HRMS(EI-MS)**: calculated $C_{16}H_{15}O$: 223.1117 (M+H), found: 223.1111.

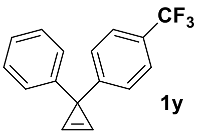

1y

Chemical Formula: $C_{16}H_{11}F_3$ Light yellow liquid, 42% yield over 3 steps.

^{1}H NMR (400 MHz, $CDCl_3$) δ: 7.55-7.46 (m, 4H), 7.34-7.12 (m, 7H); **^{13}C NMR** (100 MHz, $CDCl_3$) δ: 151.5, 146.0, 128.5, 128.4, 128.2, 126.3, 125.1, 125.1, 113.0, 31.9; **HRMS (EI-MS)**: calculated $C_{16}H_{12}F_3$: 261.0891 (M+H), found: 261.0890.

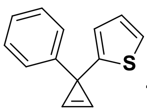

1z

Chemical Formula: $C_{13}H_{10}S$ Light yellow liquid, 32% yield over 3 steps.

^{1}H NMR (500 MHz, $CDCl_3$) δ: 7.48 (s, 2H), 7.35-7.29 (m, 4H), 7.25-7.20 (m, 1H), 7.11 (dd, J = 1.5, 5.0 Hz, 1H), 6.91 (dd, J = 3.5, 5.0 Hz, 1H), 6.70 (dd, J = 1.5, 3.5 Hz, 1H); **^{13}C NMR** (125 MHz, $CDCl_3$) δ: 155.3, 145.8, 128.4, 128.0, 127.2, 126.5, 124.5, 123.3, 113.6, 28.6; **HRMS(EI-MS)**: calculated $C_{13}H_{11}S$: 199.0576 (M+H), found: 199.0572.

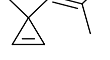

7a

Chemical Formula: C_8H_{12} Colorless liquid, 28% yield over 3 steps.

^{1}H NMR (400 MHz, $CDCl_3$) δ: 7.41 (s, 2H), 5.34 (t, J = 1.6 Hz, 1H), 1.66 (d, J = 1.6 Hz, 3H), 1.63 (d, J = 1.2 Hz, 3H), 1.23 (s, 3H); **^{13}C NMR** (100 MHz, $CDCl_3$) δ: 132.2, 131.4, 121.2, 31.4, 28.0, 26.0, 18.1; **HRMS(EI-MS)**: calculated C_8H_{13}: 109.1012 (M+H), found: 109.1014.

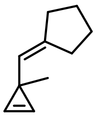

7b

Chemical Formula: $C_{10}H_{14}$ Colorless liquid, 22% yield over 3 steps.

^{1}H NMR (400 MHz, $CDCl_3$) δ: 6.23 (s, 2H), 5.51 (s, 1H), 2.80-2.70 (m, 4H), 2.45-2.38 (m, 4H), 2.14 (s, 3H); **^{13}C NMR** (100 MHz, $CDCl_3$) δ: 137.1, 128.8, 120.0, 36.3, 33.4, 29.4, 23.6; **HRMS(EI-MS)**: calculated $C_{10}H_{15}$: 135.1168 (M+H), found: 135.1167.

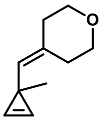

7c

Chemical Formula: $C_{10}H_{14}O$ Colorless liquid, 24% yield over 3 steps.

^{1}H NMR (500 MHz, $CDCl_3$) δ: 7.39 (s, 2H), 5.39 (s, 1H), 3.65 (q, J = 4.4 Hz, 4H), 2.37 (t, J = 4.0 Hz, 2H), 2.11 (t, J = 4.4 Hz, 2H), 1.25 (s, 3H); **^{13}C NMR** (125 MHz, $CDCl_3$) δ: 133.9, 131.3, 121.3, 69.8, 68.8, 36.7, 30.0, 28.4, 18.5; **HRMS(EI-MS)**: calculated $C_{10}H_{15}O$: 151.1117 (M+H), found: 151.1115

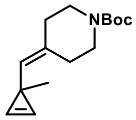

7d

Chemical Formula: $C_{15}H_{23}NO_2$ Colorless liquid, 31% yield over 3 steps.

^{1}H NMR (400 MHz, $CDCl_3$) δ: 7.40 (s, 2H), 5.42 (s, 1H), 3.38 (t, J = 6.0 Hz, 4H), 2.31 (t, J = 6.0 Hz, 2H), 2.05 (t, J = 6.0 Hz, 2H), 1.47 (s, 9H), 1.25 (s, 3H); **^{13}C NMR** (100 MHz, $CDCl_3$) δ: 155.0, 134.7, 131.9, 121.2, 79.5, 45.0, 35.6, 28.6, 28.4, 18.5; **HRMS(EI-MS)**: calculated $C_{15}H_{24}NO_2$: 250.1802 (M+H), found: 250.1794

b) isocyanide synthesis

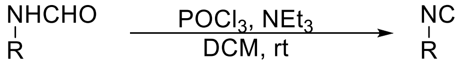

2a, **2b**, **2n**, **2o**, **2p**, **2q** are commercial available and used directly. Other isocyanide are prepared according the literature reports[8, 9], a typical procedure for preparation of isocyanides is described as follow:

A 100 mL round-bottom flask was charged with formamide (10 mmol, 1.0 equiv), triethylamine (3.00 equiv) and DCM (35 mL) and the solution was cooled to 0 ℃. Phosphorus oxychloride (1.30 equiv) was added to the reaction mixture dropwise over 15 min. The reaction mixture was stirred at 0 ℃ for 30 min and then quenched with saturated aqueous ammonium chloride (25 mL). The layers were separated and the aqueous layer was extracted with dichloromethane (2 x 60 mL). The combined organic layers were dried over sodium sulfate, filtered, and concentrated in vacuo. The crude oil was loaded onto a 1-inch silica gel plug, which was flushed with 10:1 hexanes/ethyl acetate (2 x 30 mL portions) and the collected solvent was concentrated in vacuo to yield isonitrile,

which was purified by column chromatography. **2c**[10], **2d**[11], **2e**[12], **2f**[13], **2g**[14], **2h**[15], **2i**[16], **2k**[17] are known compound and charactered in the literature.

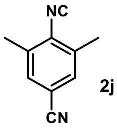

2j

Chemical Formula: $C_{10}H_8N_2$ White solid, 78% yield.

^{1}H NMR (400 MHz, $CDCl_3$) δ: 7.44 (s, 2H), 2.48 (s, 6H); **^{13}C NMR** (100 MHz, $CDCl_3$) δ: 172.2, 136.5, 131.5, 117.7, 112.6, 100.0, 18.9; **HRMS(EI-MS)**: calculated $C_{10}H_9N_2$: 157.0760 (M+H), found: 157.0757

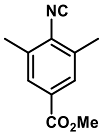

2l

Chemical Formula: $C_{11}H_{11}NO_2$ White solid, 65% yield.

^{1}H NMR (400 MHz, $CDCl_3$) δ: 7.77 (s, 2H), 3.91 (s, 3H), 2.45 (s, 6H); **^{13}C NMR** (100 MHz, $CDCl_3$) δ: 166.0, 135.2, 130.1, 130.0, 129.0, 52.5, 19.0; **HRMS(EI-MS)**: calculated $C_{11}H_{12}NO_2$: 190.0863 (M+H), found: 190.0859.

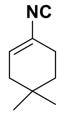

2r

Chemical Formula: $C_9H_{13}N$ Colorless liquid, 60% yield.

^{1}H NMR (400 MHz, $CDCl_3$) δ: 6.02-5.94 (m, 1H), 2.30-2.23 (m, 2H), 1.96-1.86 (m, 2H), 1.45 (t, *J* = 6.8 Hz, 2H), 0.95 (s, 6H); **^{13}C NMR** (100 MHz, $CDCl_3$) δ: 160.5, 128.1, 123.9, 38.0, 34.6, 28.3, 27.9, 26.4; **HRMS(EI-MS)**: calculated $C_9H_{14}N$: 136.1121 (M+H), found: 136.1119.

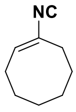

2s

Chemical Formula: $C_9H_{13}N$ Colorless liquid, 55% yield.

^{1}H NMR (400 MHz, $CDCl_3$) δ: 6.05-5.95 (m, 1H), 2.40-2.32 (m, 2H), 2.20-2.10 (m, 2H), 1.75-1.65 (m, 2H), 1.61-1.45 (m, 6H); **^{13}C NMR** (100 MHz, $CDCl_3$) δ: 159.4, 131.6, 127.1, 29.7, 29.5, 27.4, 26.0, 25.8, 25.6; **HRMS(EI-MS)**: calculated $C_9H_{14}N$: 136.1121 (M+H), found: 136.1119.

Isocyanide below was prepared according modified literature procedure[18].

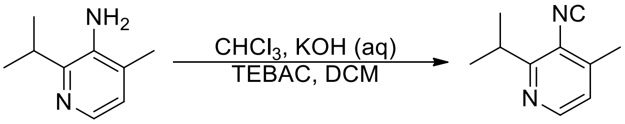

Procedure: amine (7.2 mmol) was dissolved in DCM (100 mL). Chloroform (1.5 ml) was added, followed by 50% aq. NaOH (50 mL) and TBAB (16 mol%). The biphasic mixture was vigorously stirred for 75 min., after which it was poured into water (300 mL) and extracted with AcOEt (3 x 300 mL). The collected organic phases were dried ($MgSO_4$), the volatiles were evaporated, and the product was purified by flash chromatography.

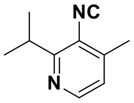

2m

Chemical Formula: $C_{10}H_{12}N_2$ Colorless liquid, 71% yield.

^{1}H NMR (400 MHz, $CDCl_3$) δ: 8.43 (d, *J* = 5.2 Hz, 1H), 7.07 (d, *J* = 4.8 Hz, 1H), 3.56 (hept, *J* = 6.8 Hz, 1H), 2.45 (s, 3H), 1.32 (dd, *J* = 0.8, 6.8 Hz, 6H); **^{13}C NMR** (100 MHz, $CDCl_3$) δ: 171.9, 162.9, 148.5, 144.1, 122.6, 31.6, 21.1, 18.6; **HRMS(EI-MS):** calculated $C_{10}H_{13}N_2$: 161.1073 (M+H), found: 161.1069.

2.2 IPent^{An} synthesis

The **IPent^{An}** HCl was prepared according to a modified literature procedure[19, 20]:

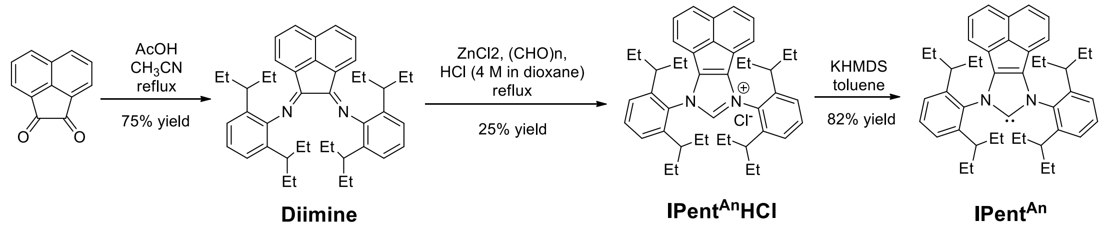

Supplementary Figure 2. Synthetic route for ligand **IPent^{An}**

Acenaphthenequinone (40 mmol) in CH_3CN (350 ml) was refluxed for 1 h before acetic acid (160 ml) was added at room temperature. After refluxed for an additional 1 hour, 2,6-di(pentan-3-yl)aniline[21] (85 mmol) was added slowly to the solution at this point, and the reaction was refluxed for another 5 hours. After the reaction was cooled down to room temperature, the yellow solid (diamine) was filtered, washed with hexane and dried under vacuum (75% yield).

A solution of above yellow solid (10 mmol, 1.0 equiv) in THF (250 mL) was treated with anhydrous $ZnCl_2$ (10 mmol, 1.0 equiv) at 70 °C and stirred for 5 min. p-Formaldehyde (11 mmol, 1.1 equiv) was subsequently added, followed by a dropwise addition of HCl (4.0 M in dioxane, 1.5 equiv, 15 mmol). The reaction was stirred at 70 °C for 3 hours and concentrated under vacuum. The residue was purified by column chromatography to afford the IPent^{An}HCl (25% yield, Hex/EA = 1:1).

In a glovebox, IPent^{An}HCl (1.0 mmol) and KHMDS (1.0 mmol) were dissolved in anhydrous toluene. The reaction mixture was stirred at room temperature for 1 hour. The reaction mixture was then filtered; the filtrate was concentrated in vacuo. The residue was washed with pentane and dried in vacuo affording the IPent^{An} (82% yield).

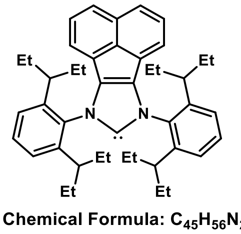

Chemical Formula: $C_{45}H_{56}N_2$

^{1}H NMR (400 MHz, C_6D_6) δ: 7.46-7.40 (m, 2H), 7.27-7.20 (m, 6H), 6.98-6.86 (m, 4H), 3.10-2.97 (m, 4H), 1.90-1.70 (m, 8H), 1.69-1.50 (m, 8H), 0.96 (t, J = 6.8 Hz, 12H), 0.66 (t, J = 6.8 Hz, 12H); **^{13}C NMR** (100 MHz, C_6D_6) δ: 232.7, 143.9, 140.9, 140.1, 131.6, 130.1, 128.7, 127.2, 127.2, 127.0, 124.9, 120.8, 42.4, 29.0, 28.4, 12.8, 11.8.

2.3 General procedure for NHC-Ni(II) catalyzed cyclopropene-isocyanide [5+1] benzannulation and diene synthesis

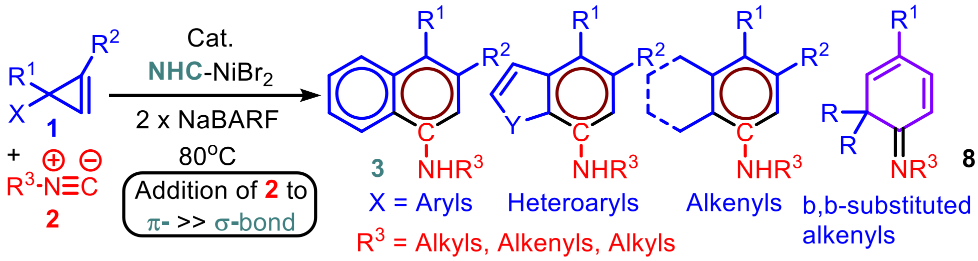

a) General Procedure for NHC-Ni(II) Catalyzed Cyclopropene-Isocyanide [5+1] Benzannulation (Figure 4-6) and Diene Synthesis (Figure 8b):

In a glovebox, an oven-dried test tube was charged with the catalyst mixture (0.05 mmol IPent^{An}/NiBr$_2$DME/NaBARF in 1:1:2 ratio) in 1 mL toluene and heated at 80°C for 3 mins. Cyclopropene **1** or **7** (0.5 mmol) and isocyanide **2** (1.0 mmol) or indicated amount of substrate were dissolved in 1 mL toluene at room tempertaure and was added in one-pot to the above in situ generated catalyst running at 80°C. The reaction was heated at 80°C for additional 12 hours except otherwise indicated.

b) General work up procedure:

Workup Method A (for product **3** and **8ab-8db**)*:* After the mixture was cooled down to room temperature, 6 mL of *n*hex/EA (10:1) was added and then the mixture was filtered through a short plug of silica gel. The solvent was removed in vacuo. Selectivity of **3** or **8** to other possible isomers were determined by ^{1}H NMR or GCMS (average of two runs). Product structures were confirmed by chromatography and isolation (5-10% EA/Hex).

Workup Method B: (for complete hydrolysis of enamine product **3vr** and **3vs** completely): The solvent was removed in vacuo after reaction was finished, and then 20 mL of MeOH/H$_2$O (20/1) was added and the mixture stirred at room temperature for overnight. After the hydrolysis was finished (monitored by GCMS), the solvent was removed in vacuo and the product structures were confirmed by chromatography and isolation (20-30% EA/Hex).

c) Example: Cyclopropene **1a** and isocyanide **2a** [5+1] reaction to give **3aa**:

In a glovebox, an oven-dried test tube was charged with the catalyst mixture (0.05 mmol IPent^{An}/NiBr$_2$DME/NaBARF in 1:1:2 ratio, 31.3 mg/15.4 mg/88.6 mg) in 1 mL toluene and heated at 80°C for 3 mins. Cyclopropene **1a** (0.5 mmol, 65 mg) and isocyanide **2a** (1.0 mmol, 131 mg) or indicated amount of substrate were dissolved in 1 mL toluene at room temperature and was added in one-pot to the above in situ generated catalyst running at 80°C. The reaction was heated at 80°C for additional 12 hours. After cooled it down to room temperature, it was diluted with 6 mL nhex/EA (10:1) and filtered through a short plug of silica gel. The solvent was then removed on rotavap and and selectivity of **3aa** to other possible isomers were determined by ^{1}H NMR or GCMS (average of two runs). Product structure was confirmed by chromatography and isolation (5-10% EA/Hex).

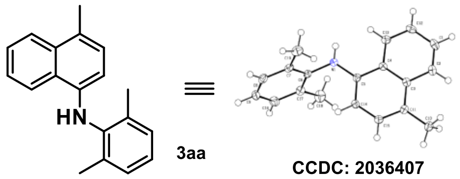

3aa   CCDC: 2036407

Chemical Formula: $C_{19}H_{19}N$

White solid, mp. 132-133°C. 68% yield, **3**/**4**: >95/5

^{1}H NMR (500 MHz, $CDCl_3$) δ: 8.15 (dd, *J* = 1.6, 7.5 Hz, 1H), 8.00 (dd, *J* = 2.0, 7.5 Hz, 1H), 7.60-7.52 (m, 2H), 7.17-7.13 (m, 2H), 7.13-7.07 (m, 1H), 7.04 (dd, *J* = 1.0, 7.5 Hz, 1H), 6.16 (d, *J* = 7.5 Hz, 1H), 5.59 (br, 1H), 2.59 (s, 3H), 2.19 (s, 6H); **^{13}C NMR** (125 MHz, $CDCl_3$) δ: 139.8, 139.3, 134.8, 133.5, 128.8, 127.0, 125.8, 125.2, 125.2, 125.0, 124.9, 124.8, 121.2, 107.8, 19.1, 18.3; **HRMS(EI-MS)**: calculated $C_{19}H_{20}N$: 262.1590 (M+H), found: 262.1603.

d) Example: Cyclopropene **7** and isocyanide **2b** [5+1] reaction to give **8ab**:

In a glovebox, an oven-dried test tube was charged with the catalyst mixture (0.05 mmol IPent^{An}/$NiBr_2$DME/NaBARF in 1:1:2 ratio, 31.3 mg/15.4 mg/88.6 mg) in 1 mL toluene and heated at 80 ^{o}C for 3 mins. Cyclopropene **7a** (0.5 mmol, 54.5 mg) and isocyanide **2b** (1.0 mmol, 187 mg) were dissolved in 1 mL toluene at room temperature and was added in one-pot to the above in situ generated catalyst running at 80 ^{o}C. The reaction was heated at 80 ^{o}C for additional 12 hours. After cooled it down to room temperature, it was diluted with 6 mL *n*hex/EA (10:1) and filtered through a short plug of silica gel. The solvent was then removed on rotavap and product **8ab** was confirmed by chromatography and isolation (5-10% EA/Hex).

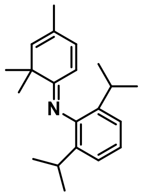

8ab

Chemical Formula: $C_{21}H_{29}N$

Colorless liquid, 65% yield.

^{1}H NMR (400 MHz, $CDCl_3$) δ: 7.13-7.06 (m, 2H), 7.05-6.98 (m, 1H), 6.19 (dd, *J* = 2.0, 9.6 Hz, 1H), 5.76 (br, 1H), 5.73 (d, *J* = 10.0 Hz, 1H), 2.75 (hept, *J* = 6.8 Hz, 2H), 1.85 (d, *J* = 1.6 Hz, 3H), 1.40 (s, 6H), 1.14 (d, *J* = 6.8 Hz, 6H), 1.06 (d, *J* = 6.8 Hz, 6H); **^{13}C NMR** (100 MHz, $CDCl_3$) δ: 172.2, 146.5, 139.9, 137.4, 137.0, 126.0, 123.1, 122.8, 118.1, 41.9, 28.9, 27.9, 23.4, 23.1, 21.1; **HRMS(EI-MS)**: calculated $C_{21}H_{30}N$: 296.2373 (M+H), found: 296.2367.

e) Gram scale experiment

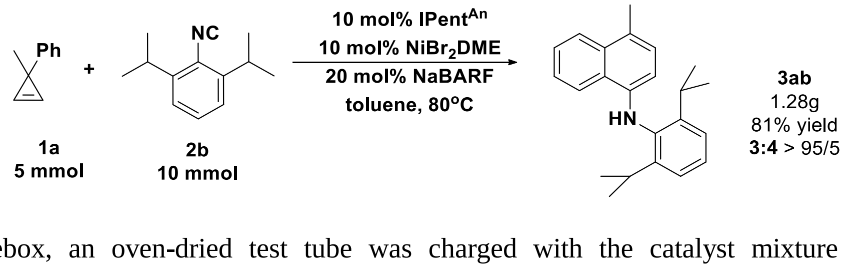

In a glovebox, an oven-dried test tube was charged with the catalyst mixture (0.5 mmol IPent^{An}/$NiBr_2$DME/NaBARF in 1:1:2 ratio) in 10 mL toluene and heated at 80°C for 3 mins. Cyclopropene **1a** (5.0 mmol) and isocyanide **2b** (10 mmol) were dissolved in 10 mL toluene at

room temperature. and was added in one-pot to the above in situ generated catalyst running at 80 °C. The reaction was heated at 80 °C for additional 12 hours. After cooled it down to room temperature, it was diluted with 60 mL *n*hex/EA (10:1) and filtered through a short plug of silica gel. The solvent was then removed on rotavap and product **3ab** was isolated by chromatography.

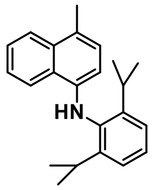

3ab

Chemical Formula: $C_{23}H_{27}N$ Dark brown liquid, 89% yield, **3**/**4**: >95/5

^{1}H NMR (400 MHz, $CDCl_3$) δ: 8.17-8.10 (m, 1H), 8.05-7.98 (m, 1H), 7.63-7.52 (m, 2H), 7.35-7.28 (m, 1H), 7.24-7.21 (m, 2H), 7.03 (dd, *J* = 0.8, 6.8 Hz, 1H), 6.10 (d, *J* = 7.6 Hz, 1H), 5.61 (br, 1H), 3.14 (hept, *J* = 6.8 Hz, 2H), 2.58 (s, 3H), 1.18 (d, *J* = 6.8 Hz, 6H), 1.09 (d, *J* = 6.8 Hz, 6H); **^{13}C NMR** (100 MHz, $CDCl_3$) δ: 146.9, 142.0, 136.1, 133.4, 127.2, 126.9, 125.8, 125.2, 124.8, 124.1, 124.0, 120.8, 107.2, 28.3, 24.9, 23.3, 19.1; **HRMS(EI-MS)**: calculated $C_{23}H_{28}N$: 318.2216 (M+H), found: 318.2216

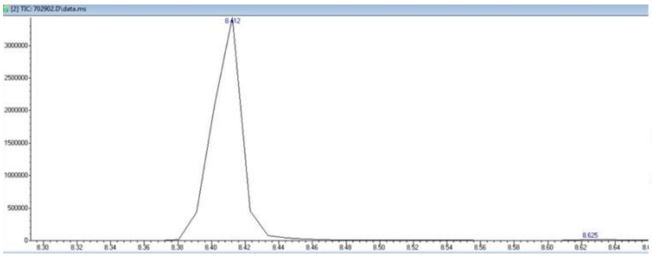

Data Path : D:\Data\hjq\7\
Data File : 702902.D
Acq On : 21 Apr 2020 09:46
Operator :
Sample : 702902

peak #	R.T. min	first scan	max scan	last scan	PK TY	peak height	corr. area	corr. % max.	% of total
4	8.412	448	454	469	rBV	3432812	4214571	100.00%	75.590%
5	8.625	472	474	480	rVB2	15860	31185	0.74%	0.559%

Supplementary Figure 3. GCMS trace of reaction crude mixture **3ab** (**3**/**4** = peak 4: peak 5 > 95/5)

2.4 Control and additional experiments

2.4.1 D-Labelling Experiments

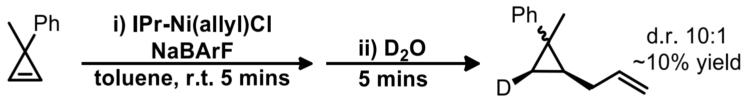

Supplementary Figure 4. D-Labelling Experiments

Trapping the isocyanide insertion intermediate (Int.-1) by D_2O was not successful at our standard condition (quenched the reaction) or at room temperature (no conversion). In order to provide a support for the NHC-Ni(II)-C bond insertion to cyclopropene step, NHC-Ni(II)-allyl insertion was carried out instead and was found to be facile at room temperature. by simply using the following procedure:

In a glove box, an oven-dried test tube was charged with [IPr-Ni(allyl)Cl] (0.3 mmol, 160.8 mg), NaBArF (0.3 mmol, 265.8 mg), cyclopropene **1a** (0.3 mmol, 39.0 mg) and toluene (3 ml). The mixture was stirred at room temperature. for 5 mins and then D_2O (0.5 ml) was added. After stirring for another 5 mins, the desired insertion product was obtained by chromatography. Colorless liquid, ~ 10% yield, d.r. 10/1.

^{1}H NMR (400 MHz, $CDCl_3$) δ: 7.35-7.25 (m, 4H), 7.21-7.15 (m, 1H), 6.07-5.95 (m, 1H), 5.15 (dd, J = 2.0, 17.2 Hz, 1H), 5.05 (dt, J = 1.6, 10.4 Hz, 1H), 2.35-2.20 (m, 2H), 1.44 (s, 3H), 1.15-1.08 (m, 2H).

2.4.2 Attempts to trap the concerned NHC-Ni(vinylcarbenoid)

Typical metal(vinylcarbenoids) could be trapped by alkene and a new cyclopropane could be obtained routinely. Yet, no such kinds of products were obtained under our standard condition (i.e. by cyclopropene itself, in the absence of **2**) and even ran under forcing conditions as follow (by large excess of additional alkenes). Only some **2**+**2** or cycloisomerization reactivity was observed, and this result suggested that the NHC-Ni(vinylcarbenoid) formation is inefficient.

Procedure in the attempts to trap the concerned NHC-Ni(vinylcarbenoid): In a glove box, an oven-dried test tube was charged with 10 mol% catalyst mixture (0.05 mmol IPent^{An}/$NiBr_2$DME/NaBARF in 1:1:2 ratio) in 1 mL toluene and stirred at 80°C for 3 mins. A solution of alkene (5 mmol) in toluene (2 ml) was added and then another solution of cyclopropene (0.5 mmol, 1 equiv.) in toluene (2 ml) was added dropwise in 10 mins. The mixture was stirred at 80°C until complete consumption of the cyclopropene **1a** or **1w**.

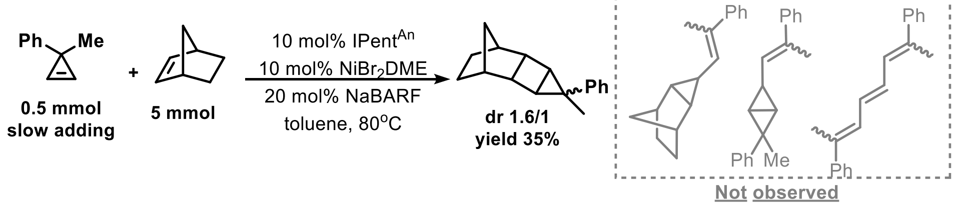

(Eq 1)

Supplementary Figure 5. attempts to trap the concerned NHC-Ni(vinylcarbenoid)

(Eq 1) Cyclopropene = **1a**; Alkene = Norbornene

A mixture of cross-products was isolated by chromatography at 35% yield and at 1.6/1 d.r.. Unfortunately, the relative configuration could not be confirmed rigorously due to significant peaks overlap. Remaining **1a** was converted non-selectively into gel /polymer with limited solubility in DMSO.

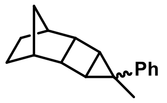

Chemical Formula: $C_{17}H_{20}$ Colorless liquid, 35% yield, 1.6/1 dr.

^{1}H NMR (400 MHz, $CDCl_3$) δ: 7.40-7.10 (m, 5H), 2.37-2.25 (m, 1H), 2.12 (s, 2H, minor) 2.03 (s, 2H, major), 1.85-1.50 (m, 3H), 1.45-1.32 (m, 3H), 1.31-1.05 (m, 5H), 0.85-0.75 (m, 1H); **^{13}C NMR** (100 MHz, $CDCl_3$) δ: 147.1 (142.2), 130.2 (128.3), 128.2 (127.0), 126.0 (125.6), 43.0, 42.6, 38.6, 38.5, 33.4, 33.2, 30.3, 30.0, 28.4, 28.1, 26.4, 14.4; **HRMS(EI-MS)**: calculated $C_{17}H_{21}$: 225.1638 (M+H), found: 225.1635.

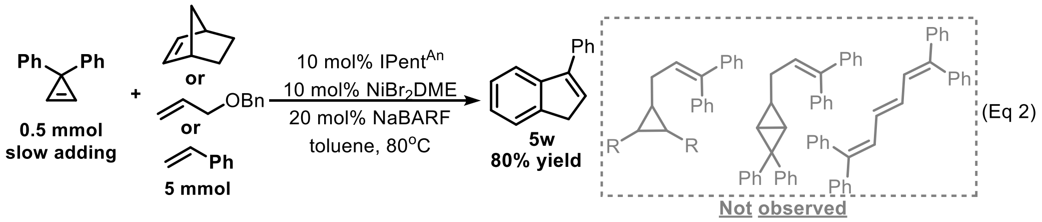

Supplementary Figure 6. attempts to trap the concerned NHC-Ni(vinylcarbenoid)

(Eq 2) Cyclopropene = **1w**; Alkene = Norbornene, styrene or allyl-OBn

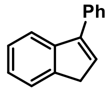

5w

Chemical Formula: $C_{15}H_{12}$ Colorless liquid, 80% yield.

5w was isolated by chromatography with ~80% yield. **^{1}H NMR** (500 MHz, $CDCl_3$) δ: 7.62-7.57 (m, 3H), 7.53 (d, J = 7.5 Hz, 1H), 7.44 (t, J = 7.5 Hz, 2H), 7.39-7.34 (m, 1H), 7.31 (t, J = 7.5 Hz, 1H), 7.25 (dt, J = 1.0, 7.5 Hz, 1H), 6.57 (t, J = 2.5 Hz, 1H), 3.49 (d, J = 2.0 Hz, 2H); **^{13}C NMR** (125 MHz, $CDCl_3$) δ: 145.3, 144.9, 144.0, 136.3, 131.1, 128.7, 127.8, 127.7, 126.3, 125.0, 124.2, 120.4, 38.3; **HRMS(EI-MS):** calculated $C_{15}H_{13}$: 193.1012 (M+H), found: 193.1008.

2.4.3 Isolation of [*trans*-IPr-Ni(CN-DIPP)Br_2]

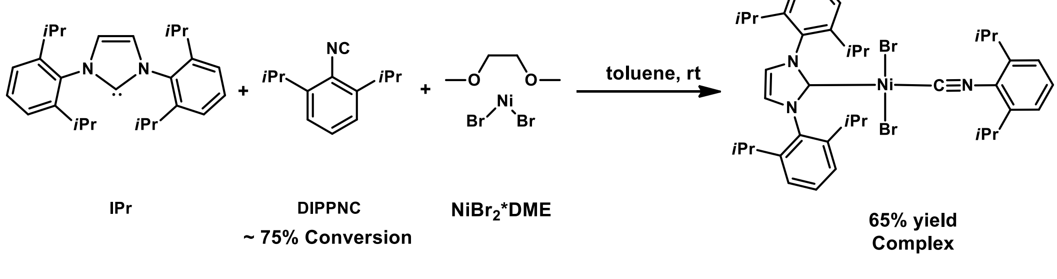

Preparation procedure: IPr/$NiBr_2$DME (0.4 mmol in 1:1 ratio) and (DIPP)NC (**2b**, 0.6 mmol, 113

mg) were suspended in 10 mL toluene and stirred for 6 hours at room temperature. Toluene was then removed in vacuum to yield a red solid, which was washed with 3 x 2 mL portions of hexanes and dried in vacuum to yield the desired complex (65% yield). Crystal suitable for single crystal X-ray crystallography was obtained by preparing a saturated solution in toluene and then leaves it alone for overnight.

^{1}H NMR (400 MHz, C_6D_6) δ: 7.40-7.15 (m, 6H), 6.85-6.45 (m, 5H), 3.60-3.38 (m, 4H), 3.36-3.14 (m, 2H), 1.85-1.50 (m, 12H), 1.15-0.75 (m, 24H); **^{13}C NMR** (100 MHz, C_6D_6) δ: 147.5, 146.8, 136.6, 131.0, 130.1, 125.8 (br), 124.8, 123.6, 29.6, 29.6, 26.9, 24.1, 23.0.

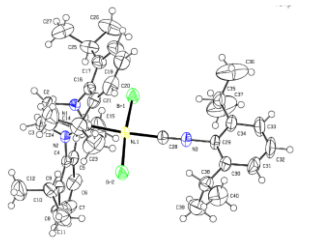

CCDC: 2036409

Supplementary Figure 7. X-ray crystallography of [*trans*-IPr-Ni(CN-DIPP)Br_2]

2.4.4 Attempts to use NHC-Ni(0) as catalyst for the [5+1] benzannulation

A) Synthesis of the complexes: the following IPr-Ni(0)L_n catalysts were prepared by the following literature procedure:

a) Ni(IPr)(methyl methacrylates)$_2$ complex[22]

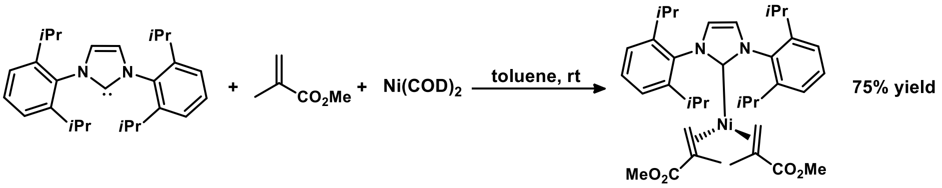

Ni(IPr)(methyl methacrylates)$_2$
Chemical Formula: $C_{37}H_{52}N_2NiO_4$

Ni(IPr)(methyl methacrylates)$_2$ complex was prepared according to the literature with slight modifications: A solution of methyl methacrylate (6.0 mmol) in 8 mL of toluene was added to a 50 ml Schlenk flask containing Ni(COD)$_2$ (1.5 mmol). After stirring for 20 min a solution of IPr (1.5 mmol) in 8 mL of toluene was added. The resulting solution was stirred for 2 hours and the bright yellow precipitate was formed. 20 mL of pentane was added to the reaction and it was stirred for 20 min. A bright yellow solid was isolated by filtration in 75 % yield, and the spectroscopic data is consistent with that reported in the literature. ^{1}H NMR contained potential isomers that were in thermal equilibrium with one another and peak numbers reflect NMR spectra at 25 ℃ which is included in this document.

^{1}H NMR (500 MHz, C_6D_6): δ 7.09-7.04, 7.02-6.98, 6.62-6.57, 3.44, 3.32, 3.14 -3.04, 3.02-2.86, 2.68-2.55, 1.40, 1.35, 1.26-1.20, 1.03- 0.90. **^{13}C NMR** (125 MHz, C_6D_6) δ: 200.94, 200.29, 173.47, 173.38, 146.16, 145.64, 137.12, 129.98, 124.68, 124.62, 124.26, 124.20, 124.10, 65.44, 58.43,

50.64, 50.22, 28.91, 28.79, 26.30, 26.28, 22.74, 22.42, 22.36, 22.27, 18.67, 17.91, 14.3.

b) (IPr)Ni(CNXyl)$_3$ complex[23]

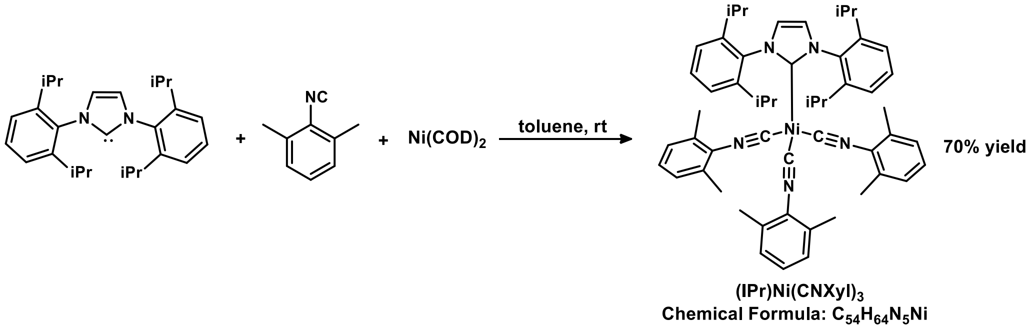

(IPr)Ni(CNXyl)$_3$ complex was prepared according to the literature with slight modifications: In a glovebox, Ni(COD)$_2$ (0.5 mmol) was weighed into a 20 mL vial. IPr (0.5 mmol) and CNXyl (1.5 mmol) were then weighed into two separate 5 mL vials. A stirring bar was placed in the 20 mL vial, and the contents were dissolved in toluene (8 mL). In both 5 mL vials was placed toluene (3 mL). The IPr and CNXyl solutions, in that order, were then added dropwise to the stirred Ni(COD)$_2$ solution at room temperature. After 5 min, the volatiles were removed in vacuo, and the residue was dissolved in minimal hexanes. The solution was filtered through Celite and cooled to -35 ℃ overnight. 70% yield of desired product was obtained, the spectroscopic data was consistent with that reported in the literature.

^{1}H NMR (400 MHz, C_6D_6) δ: 7.15-7.08 (m, 6H), 6.82-6.77 (m, 9H), 6.72-6.68 (m, 2H), 3.26 (hept, J = 6.8 Hz, 4H), 2.24 (s, 18H), 1.47 (d, J = 6.8 Hz, 12H), 1.17 (d, J = 6.8 Hz, 12H); **^{13}C NMR** (100 MHz, C_6D_6) δ: 206.26, 181.15, 146.48, 139.75, 133.19, 132.61, 128.85, 127.64, 124.53, 123.68, 122.57, 28.84, 25.31, 23.48, 19.59.

B) Attempts to use NHC-Ni(0) for the [5+1] benzannulation.

The following two cyclopropenes and isocyanide were employed as substrate pairs and examined by NHC-Ni(0) at a physical condition same as the standard [5+1] benzannulation condition.

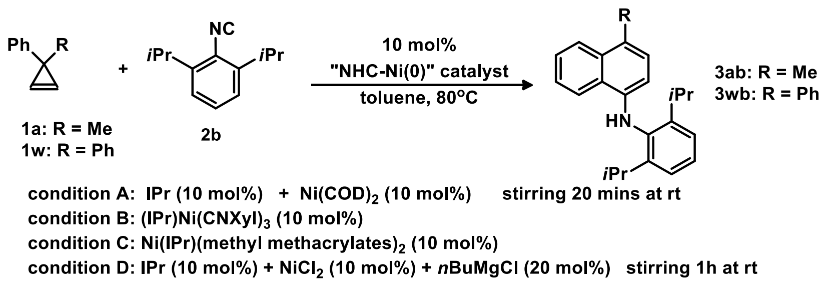

Supplementary Figure 8. Attempts to use NHC-Ni(0) for the [5+1] benzannulation

General procedure: Everything is the same as our standard [5+1] benzannulation condition, except the catalysts employed are different:

In a glove box, an oven-dried test tube was charged with 10 mol% catalyst mixture in 1 mL toluene and stirred at 80°C for 3 mins. A solution of cyclopropene and isocyanide in toluene was added directly to the catalyst and the mixture was stirred for further 12 hours at 80°C. The conversion of starting materials and crude yield of desired product were determined by ^{1}H NMR after removing solvent in vacuo.

Supplementary Table 1. NHC-Ni(0) catalyst for [5+1] benzannulation

**Entry**	**NHC-Ni(0) generation method**	**Cyclopropene** Conversion %	**Isocyanide** Conversion %	**Product** Yield %
1	Condition A<br>IPr + Ni(COD)$_2$<br>20 mins at room temperature	**1a**<br><10%	**2b**<br><10%	N.D
2		**1w**<br>~15%	**2b**<br><20%	N.D
3	Condition B<br>IPr-Ni(CNXyl)$_3$	**1a**<br>~20%	**2b**<br><10%	N.D
4		**1w**<br><20%	**2b**<br><20%	N.D
5	Condition C<br>IPr(methyl methacrylate)$_2$Ni	**1a**<br><10%	**2b**<br><10%	N.D
6		**1w**<br><10%	**2b**<br><20%	N.D
7[24, 25]	Condition D<br>IPr + NiCl$_2$ + nBuMgCl<br>1 hour at room temperature.	**1a**<br><10%	**2b**<br><10%	N.D
8[24, 25]		**1w**<br>~20%	**2b**<br><20%	N.D

The results of above control reactions suggest that the desired [5+1] reactivity was unlikely caused by NHC-Ni(0) species.

2.4.5 Attempts to use an in situ generated styrenyl ketenimine as a substrate for a 6-π electron cyclization to naphthamine

Styrenyl ketenimines were generated in situ based on a relevant literature[26] procedure for our 6-π electron cyclization study at 80°C:

In a glove box, an oven-dried test tube was charged with 10 mol% Pd(OAc)$_2$ (0.05 mmol), allyl carbonate and *t*BuNC (in 1:2 ratio) in 3 mL toluene and heated at 80°C for 12 hours (no allyl carbonate left). The reaction mixture was then allowed to cool to room temperature.

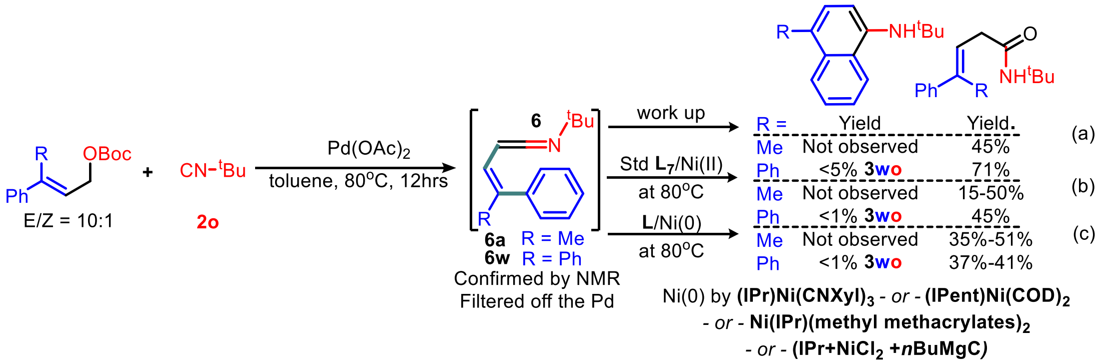

Supplementary Figure 9. Attempts to use styrenyl ketenimine as a substrate to prepare

naphthamine via 6-π electron cyclization

Procedure (Eq a): After reacted for 12 hours at 80°C, the reaction was cooled down to room temperature. Column chromatography with PE/EtOAc as eluent afforded the hydrolyzed product (amide) and only trace amount of aromatic amine was obtained.

Procedure (Eq b, NHC-Ni(II) catalysis): Same as (Eq a), except the in situ generated styrenyl ketenimine (after removing Pd catalyst via filtering) was subjected to a further 12 hours heating at 80°C in another test tube charged with our typical NHC-Ni(II) catalyst (0.05 mmol generated by standard procedure, with $IPent^{An}$ NHC or without NHC for a comparison). Work up procedure is same as the above (Eq a), the hydrolyzed product and only trace amount of aromatic amine was obtained.

Procedure (Eq c, NHC-Ni(0) catalysis): Same as (Eq b), except the another test tube was charged with NHC-Ni(0) catalyst (0.05 mmol) in toluene generated from the conditions below:

NHC-Ni(0) catalyst:
condition A: IPr (10 mol%)+ $Ni(COD)_2$ (10 mol%), stirring 20 mins at room temperature
condition B: $(IPr)Ni(CNXyl)_3$ (10 mol%)
condition C: $Ni(IPr)(methyl methacrylates)_2$ (10 mol%)
condition D: IPr (10 mol%)+$NiCl_2$ (10 mol%)+*n*BuMgCl (20 mol%), stirring 1 hour at room temperature

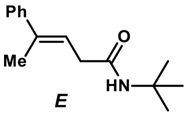

Chemical Formula: $C_{15}H_{21}NO$ Colorless liquid, 45% yield, E/Z = 6.4/1.

^{1}H NMR (400 MHz, $CDCl_3$) δ: 7.48-7.25 (m, 5 H), 5.94 (dt, *J* = 1.2, 7.6 Hz, 1H), 5.55 (br, 1H), 3.12 (dd, *J* = 0.8, 7.6 Hz, 2H), 2.09 (s, 3H), 1.37 (s, 9H); **^{13}C NMR** (100 MHz, $CDCl_3$) δ: 170.1, 143.0, 139.6, 128.4, 127.4, 125.8, 120.5, 51.3, 37.8, 28.9, 16.2; **HRMS(EI-MS)**: calculated $C_{15}H_{22}NO$: 232.1696 (M+H), found: 232.1692.

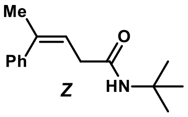

Chemical Formula: $C_{15}H_{21}NO$ Colorless liquid.

^{1}H NMR (500 MHz, $CDCl_3$) δ: 7.42-7.17 (m, 5H), 5.68 (dt, *J* = 1.5, 7.5 Hz, 1H), 5.72 (br, 1H), 2.84 (dd, *J* = 1.5, 7.5 Hz, 2H), 2.12 (s, 3H), 1.34 (s, 9H); **^{13}C NMR** (125 MHz, $CDCl_3$) δ: 170.8, 141.2, 140.9, 128.5, 127.9, 127.2, 120.0, 51.3, 38.1, 28.9, 25.9; **HRMS(EI-MS)**: calculated $C_{15}H_{22}NO$: 232.1696 (M+H), found: 232.1692.

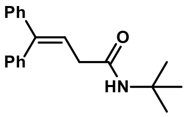

Chemical Formula: $C_{20}H_{23}NO$ Colorless liquid, 71% yield.

¹H NMR (400 MHz, $CDCl_3$) δ: 7.42-7.15 (m, 10H), 6.28 (t, *J* = 7.6 Hz, 1H), 5.33 (br, 1H), 2.96 (d, *J* = 7.6 Hz, 2H), 1.32 (s, 9H); **¹³C NMR** (100 MHz, $CDCl_3$) δ: 170.2, 145.1, 141.9, 139.4, 129.8, 128.5, 128.3, 127.5, 127.5, 121.8, 51.3, 38.7, 28.9; **HRMS(EI-MS)**: calculated $C_{20}H_{24}NO$: 294.1852 (M+H), found: 294.1848.

The in situ generated styrenyl ketenimines were characterized by crude ¹H and ¹³C NMR after filtration:

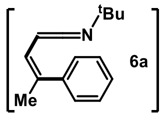

Chemical Formula: $C_{15}H_{19}N$

Characteristic peaks of the alkenyl CHs are shown below:

¹H NMR (500 MHz, C_6D_6) δ: 7.32-7.27 (m, 2H), 7.18-7.12 (m, 2H), 7.08-7.01 (m, 1H), 6.39 (dd, *J* = 1.5, **11.0 Hz**, 1H), 4.75 (d, *J* = **11.0 Hz**, 1H), 1.88 (s, 3H), 1.15 (s, 9H); The styrenyl ketenimine quaternary C: **¹³C NMR** (125 MHz, C_6D_6) δ: **184.8**, 143.5, 129.0, 128.2, 128.0, 126.0, 125.2, 119.2, 67.9, 58.7, 31.0, 29.7.

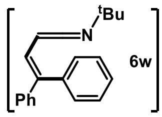

Chemical Formula: $C_{20}H_{21}N$

Characteristic peaks of the alkenyl CHs are shown below:

¹H NMR (500 MHz, C_6D_6) δ: 7.4-7.0 (m, 10H), 6.65 (d, *J* =**11.5** Hz, 1H), 4.84 (d, *J* = **11.5** Hz, 1H), 1.09 (s, 9H); The styrenyl ketenimine quaternary C: **¹³C NMR** (125 MHz, C_6D_6) δ: **184.1**, 143.2, 141.0, 130.8, 128.8, 128.7, 128.5, 127.4, 127.3, 127.2, 126.6, 121.2, 68.2, 59.3, 30.1.

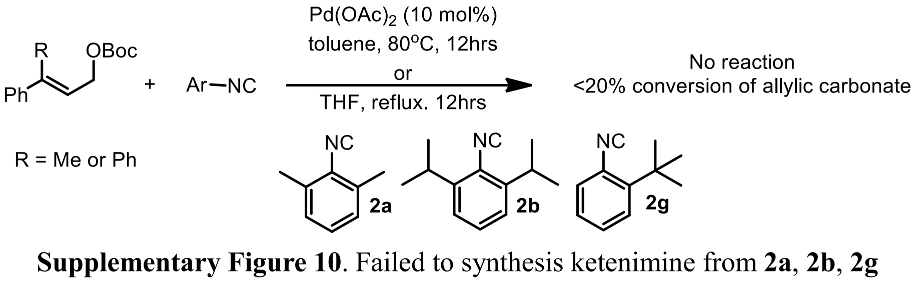

Supplementary Figure 10. Failed to synthesis ketenimine from **2a**, **2b**, **2g**

Attempts to synthesize the ketenimine from the above aryl substituted isocyanides under same condition were summarily failed in either THF or toluene. No styrenyl ketenimine, amide or aromatic amine was observed.

2.4.6 Synthesis of the authentic dimer of **1a** (i.e. **4a**) by $PdCl_2$ for comparison.

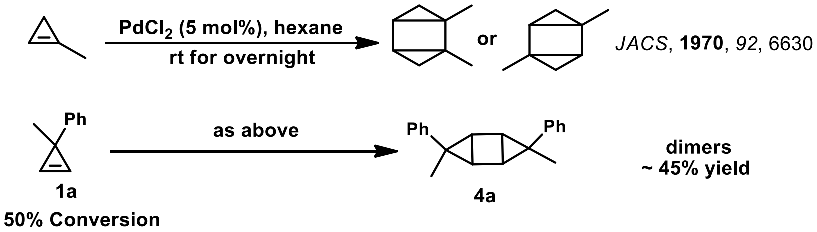

Supplementary Figure 11. Synthesis of dimers of **1a**

Procedure[27]: In a glove box, an oven-dried test tube was charged with 5 mol% $PdCl_2$ and cyclopropene **1a** (0.5 mmol). The mixture was stirred at room temperature for overnight. The conversion by NMR was determined after removal of solvent. The mixture of dimers was obtained by chromatography.

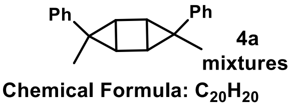

Colorless liquid, 45% yield.

^{1}H NMR (500 MHz, $CDCl_3$) δ: (mixtures)7.40-7.24 (m, 2H), 7.21-7.10 (m, 2H), 7.09-7.02 (m, 1H), 1.86-0.95 (m, 10H); **^{13}C NMR** (125 MHz, $CDCl_3$) δ: (mixtures)145.7, 145.6, 143.5, 143.3, 129.7, 129.6, 128.4, 128.3, 128.2, 127.9, 127.7, 126.2, 126.0, 126.0, 47.6, 47.4, 44.9, 44.8, 31.0, 30.1, 29.3, 28.8, 24.9, 24.5, 17.0, 16.7

The NMR obtained was found the same as those dimers isolated from our standard condition.

2.4.7 [5+1] reaction in the presence of TEMPO

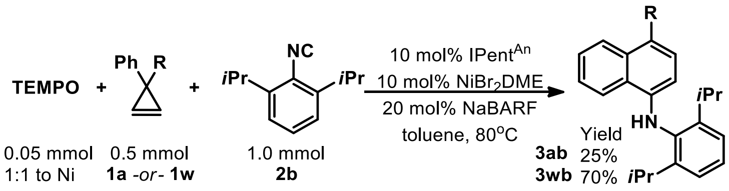

The reaction was carried out according to the standard [5+1] reaction procedure, except **10 mol% TEMPO** (Ni:TEMPO = 1:1) was added to the substrate solution and then was added to the catalyst together. The desired [5+1] reactivity and aromatic product were observed even ran in the presence of TEMPO, indicated that the chance of radical based catalysis is low.

2.4.8 IPr-NiCl dimer reaction

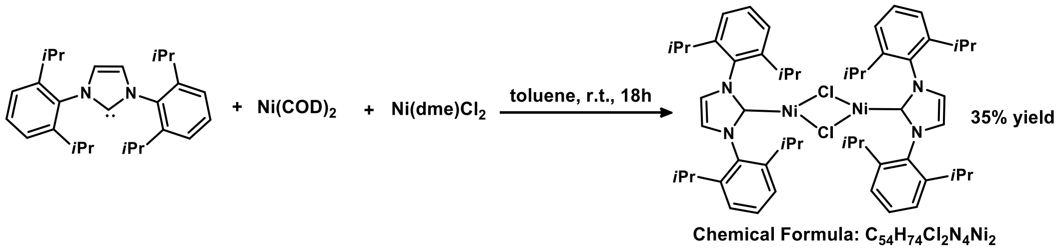

IPr-NiCl dimer was generated according to literature procedure[28]: Inside an Argon-filled glovebox, Ni(dme)Cl_2 (217.9 mg, 1.0 mmol, 1.0 equiv.), $Ni(COD)_2$ (275.0 mg, 1.0 mmol, 1.0 equiv.) and IPr (776.0 mg, 2.0 mmol, 2.0 equiv.) were suspended in toluene (15 mL). The resulting mixture was stirred at ambient temperature inside the glovebox for 18 h. The obtained mixture was filtered and the filtrate concentrated to 6 mL under reduced pressure. Pentane (20 mL) was added to and the mixture was cooled at -25 °C overnight. The formed crystals were collected by filtration and washed with cold pentane (3 x 2 mL) to afford Ni(I) dimer as greenish yellow crystals in 35% yield (340 mg, 0.35 mmol). The spectroscopic data was consistent with that reported in the literature.

^{1}H NMR (400 MHz, C_6D_6) δ: 7.16-7.05 (m, 12H), 6.67 (s, 4H), 3.10 (hept, J = 6.8 Hz, 8H), 2.50 (d, J = 6.8 Hz, 24H), 1.16 (d, J = 6.8 Hz, 24H); **^{13}C NMR** (100 MHz, C_6D_6) δ: 146.3, 139.3, 130.5, 129.6, 123.5, 29.7, 24.9, 24.4.

(IPr-Ni-Cl)$_2$ was examined as catalyst for the cyclopropene-isocyanide [5+1] reaction in chlorobenzene. Only trace amount desired product **3wb** was obtained from various conditions as follow:

Supplementary Table 2. (IPr-Ni-Cl)$_2$ as catalyst for [5+1] reaction

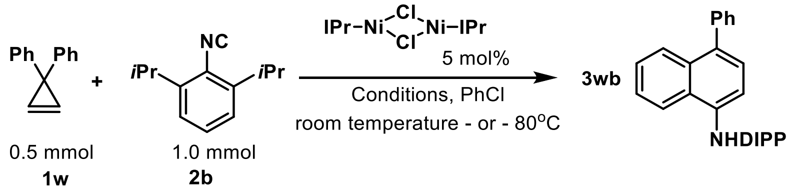

Entry	Temp (°C)	NaBArF	Conversion% (**1w**/**2b**)	Yield % **3wb**	**5w**
1	room temperature	nil	15/35	Not observed	
2	room temperature	10 mol%	60/80		17%
3	80°C	Nil	35/40	< 2 - 6%	
4	80°C	10 mol%	80/80		~24%

After workup, the yield of **3wb** and **5w** were determined by NMR. The above attempts showed that the desired [5+1] reaction is much less efficient than our standard condition. We believe the formation of **5w** and possibly also the **3wb** were mainly caused by a small amount of NHC-Ni(II) and/or the decomposed catalyst over time.

2.4.9 Cyclopropylcarbonitrile rearrangement by alkylation

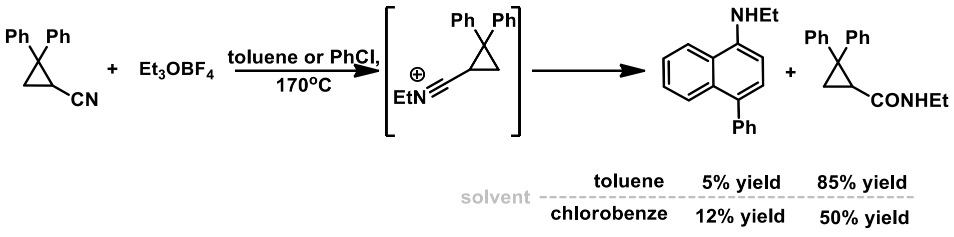

Supplementary Figure 12. Cyclopropylcarbonitrile rearrangement by alkylation

In a glove box, an oven-dried flask was charged with a cyclopropylcarbonitrile as indicated above (0.5 mmol, 110 mg), triethyloxonium tetrafluoroborate (0.75 mmol, 143 mg) and 5 mL toluene or chlorobenzene. The flask was sealed and stirred at 170°C for overnight. After the reaction mixture

was cooled to room temperature, the solvent was removed in vacuo and the products were isolated by column chromatography.

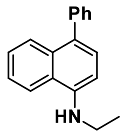

Chemical Formula: $C_{18}H_{17}N$ Colorless liquid

^{1}H NMR (400 MHz, $CDCl_3$) δ: 7.91 (dd, J = 1.6, 8.4 Hz, 1H), 7.87 (dd, J = 1.6, 8.4 Hz, 1H), 7.50-7.35 (m, 7H), 7.31 (d, J = 7.6 Hz, 1H), 6.68 (d, J = 8.0 Hz, 1H), 4.35 (br, 1H), 3.36 (q, J = 6.8 Hz, 2H), 1.43 (t, J = 6.8 Hz, 3H); **^{13}C NMR** (100 MHz, $CDCl_3$) δ: 143.2, 141.6, 132.4, 130.5, 129.7, 128.3, 127.9, 126.9, 126.6, 125.9, 124.7, 123.5, 120.2, 104.2, 38.9, 15.0; **HRMS(EI-MS)**: calculated $C_{18}H_{18}N$: 248.1434 (M+H), found: 248.1426.

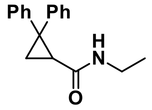

Chemical Formula: $C_{18}H_{19}NO$ Colorless liquid

^{1}H NMR (400 MHz, $CDCl_3$) δ: 7.35-7.10 (m, 10H), 5.94 (br, 1H), 3.15-2.98 (m, 2H), 2.28 (dd, J = 6.0, 8.3 Hz, 1H), 2.09 (dd, J = 4.8, 6.0 Hz, 1H), 1.50 (dd, J = 4.8, 8.0 Hz, 1H), 0.89 (t, J = 6.8 Hz, 3H); **^{13}C NMR** (100 MHz, $CDCl_3$) δ: 169.1, 145.4, 140.2, 129.9, 128.4, 128.3, 127.4, 126.9, 126.3, 38.4, 34.5, 31.2, 19.5, 14.8; **HRMS(EI-MS)**: calculated $C_{18}H_{20}NO$: 266.1539 (M+H), found: 266.1532.

2.4.10 The bulky NHC **L7** steric effect on **Int.-2** conformation

A conformational analysis suggested that the **Int.-2** conformations are restricted by the bulky substituent repulsions among the **L7** and the substrate. As a result, it affected the reactivity of the desired cyclization and the undesired styrenyl ketenimine formation.

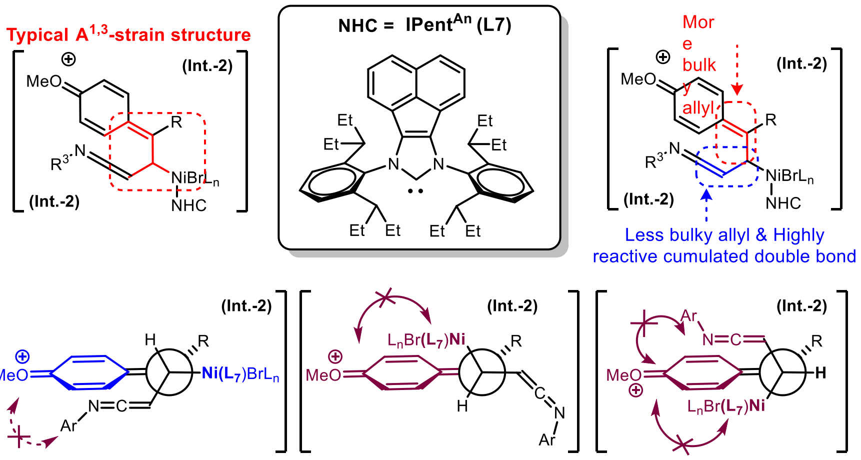

**Undesired Steric interactions related to NHC**	**Low**	High	**Very High**
**$A^{1,3}$-strain**	**Moderate**	Higher	**The highest**
**δ Ni-C overlap with pro-aromatic π-system**	**The Lowest**	Possible (but hard to access)	Possible (but hard to access)
**Possible allylic interaction site**	**Cumulated alkene (the ketenimine) > methallyl on cyclic tether**	Ketenimine > methallyl on cyclic tether	Ketenimine > methallyl on cyclic tether

Supplementary Figure 13. conformational analysis of **Int.-2**

In the stabilized conformation (A), most of the severe steric interactions are minimized. The two π-systems are aligned on the same side for the Path 1 (as shown below) accordingly. Conformations (B) and (C) offer a better δC-Ni overlap with the cyclic π-system tether for the styrenyl ketenimine formation (Path 2 as shown below). However, strong steric repulsions are involved, and it will increase severely as the R/Ar size increases. Hence, the styrenyl ketenimine formation from **Int.-2** is difficult with a bulky NHC and is unlikely a path for the desired product formation.

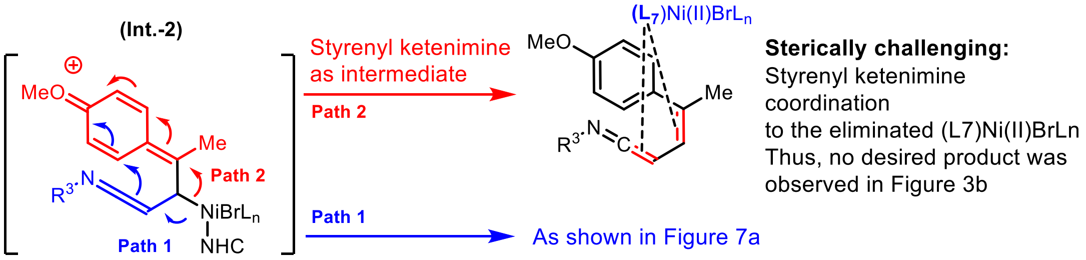

Supplementary Figure 14. Possible path of **Int.-2**

It should be noted that only trace amount of desired product was obtained from styrenyl ketenimine and a cationic Ni(II) catalyst (the control experiment in Figure 3b). This result suggested the **Int.-2** regeneration by a cationic Ni(II) catalyst re-addition to the styrenyl ketenimine is difficult, especially in the presence of the isocyanide and the Lewis basic N centers under the standard catalytic reaction condition.

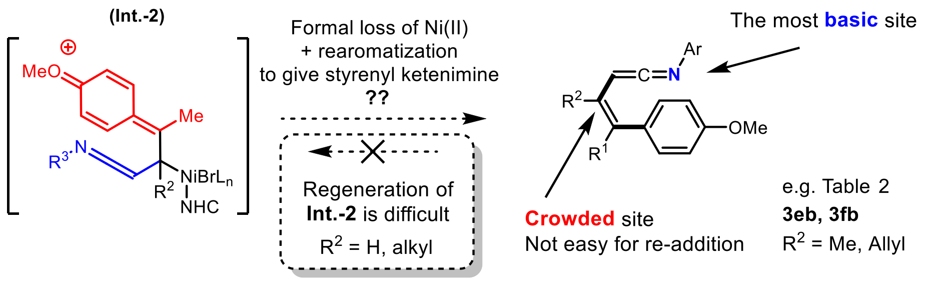

Supplementary Figure 15. **Int.-2** regeneration via Ni(II) catalyst re-addition to styrenyl ketenimine is difficult

2.5 Solvent-controlled regio-divergent bromination of [5+1] product

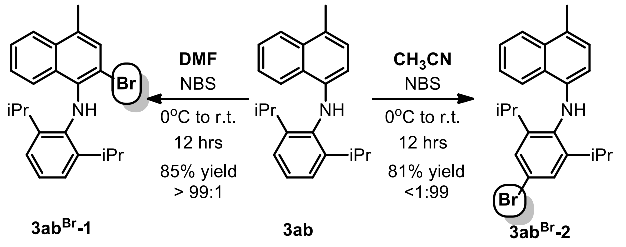

Supplementary Figure 16. regio-divergent bromination of [5+1] product

Bromination procedure: **3ab** [N-(2,6-diisopropylphenyl)-4-methylnaphthalen-1-amine] (160 mg, 0.5 mmol) was dissolved in 2 ml DMF or CH_3CN, and then NBS (98 mg, 0.55 mmol) was added to the reaction in one batch at 0°C. The reaction was allowed to reach room temperature. gradually, and the mixture was stirred for 12 hours. In the work up, 10 mL water, 15 mL hexane and 5 mL ethyl acetate were added sequentially. The organic layer was separated and washed with water, dried over anhydrous Na_2SO_4, concentrated in vacuum, subjected to GCMS analysis and column chromatography.

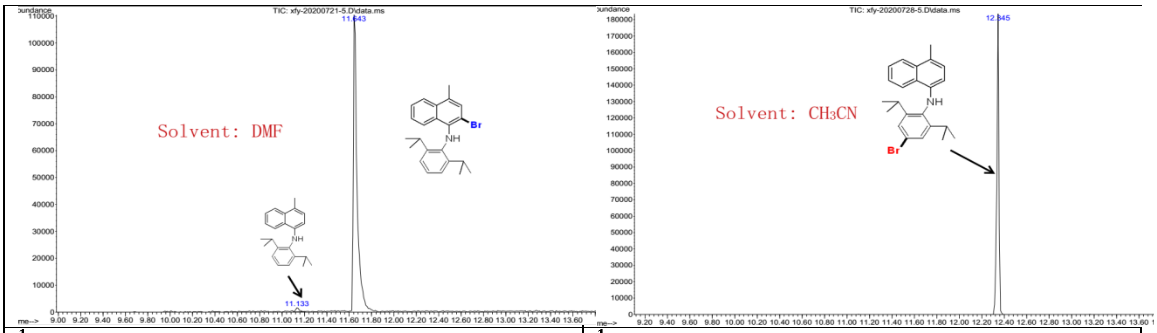

^{1}**H NMR** (500 MHz, $CDCl_3$) δ: 7.87-7.82 (m, 1H), 7.50-7.42 (m, 2H), 7.39-7.34 (m, 1H), 7.25-7.20 (m, 1H), 7.18-7.14 (m, 2H), 7.10-7.03 (m, 1H), 5.90 (s, 1H), 3.20 (hept, *J* = 6.8 Hz, 2H), 2.58 (s, 3H), 1.16 (d, *J* = 7.0 Hz, 6H), 0.88 (d, *J* = 6.5 Hz, 6H);<br>^{13}**C NMR** (100 MHz, $CDCl_3$) δ: 145.1, 138.9, 138.8, 133.3, 130.6, 126.5, 126.2, 126.0, 125.8, 124.8, 124.6, 124.1, 124.1, 107.6, 28.4, 24.6, 22.7, 18.9;<br>**HRMS(EI-MS)**: calculated $C_{23}H_{27}BrN$: 396.1327 (M+H), found: 396.1331.	^{1}**H NMR** (400 MHz, $CDCl_3$) δ: 8.15-8.08 (m, 1H), 8.05-7.96 (m, 1H), 7.61-7.50 (m, 2H), 7.40-7.30 (m, 2H), 7.03 (d, *J* = 7.6 Hz, 1H), 6.09 (dd, *J* = 2.1, 7.6 Hz, 1H), 5.50 (br, 1H), 3.09 (hept, *J* = 6.8 Hz, 2H), 2.58 (s, 3H), 1.16 (dd, *J* = 2.0, 6.8 Hz, 6H), 1.07 (d, *J* = 6.8 Hz, 6H);<br>^{13}**C NMR** (125 MHz, $CDCl_3$) δ: 149.2, 141.5, 135.3, 133.4, 127.5, 127.1, 126.0, 125.3, 125.0, 124.7, 124.0, 120.8, 120.7, 107.4, 28.4, 24.7, 23.1, 19.1;<br>**HRMS(EI-MS)**: calculated $C_{23}H_{27}BrN$: 396.1327 (M+H), found: 396.1329.

Supplementary Figure 17. GCMS traces and NMR data of **3ab^{Br}-1** and **3ab^{Br}-2**

2.6 Product characterization data and GCMS trace

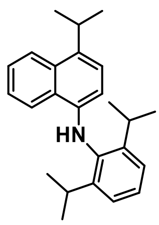

3bb

Chemical Formula: $C_{25}H_{31}N$ Dark brown liquid, 85% yield, **3**/**4**: >95/5

^{1}H NMR (500 MHz, $CDCl_3$) δ: 8.20-8.12 (m, 2H), 7.60-7.52 (m, 2H), 7.34-7.29 (m, 1H), 7.27-7.24 (m, 2H), 7.13-7.09 (m, 1H), 6.17 (d, *J* = 8.0 Hz, 1H), 5.60 (br, 1H), 3.64 (hept, *J* = 6.8 Hz, 1H), 3.14 (hept, *J* = 6.8 Hz, 2H), 1.35 (d, *J* = 7.0 Hz, 6H), 1.18 (d, *J* = 7.0 Hz, 6H), 1.10 (d, *J* = 7.0 Hz, 6H); **^{13}C NMR** (125 MHz, $CDCl_3$) δ: 146.8, 141.6, 136.2, 134.5, 132.2, 126.9, 125.7, 124.6, 124.4, 124.1, 124.0, 122.4, 121.0, 107.5, 28.3, 28.2, 25.0, 23.8, 23.3; **HRMS(EI-MS)**: calculated $C_{25}H_{32}N$: 346.2529 (M+H), found: 346.2529.

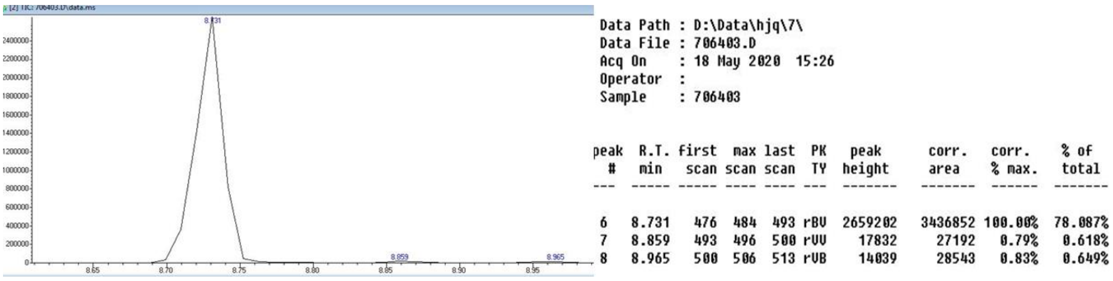

Data Path : D:\Data\hjq\7\
Data File : 706403.D
Acq On : 18 May 2020 15:26
Operator :
Sample : 706403

peak #	R.T. min	first scan	max scan	last scan	PK TY	peak height	corr. area	corr. % max.	% of total
6	8.731	476	484	493	rBV	2659202	3436852	100.00%	78.087%
7	8.859	493	496	500	rVV	17832	27192	0.79%	0.618%
8	8.965	500	506	513	rVB	14039	28543	0.83%	0.649%

Supplementary Figure 18. GCMS trace of reaction crude mixture **3bb** (**3**/**4** = peak 6: peak 7+8 > 95/5)

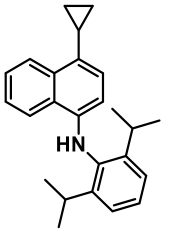

3cb

Chemical Formula: $C_{25}H_{29}N$ Dark brown liquid, 71% yield, **3**/**4**: >95/5

^{1}H NMR (400 MHz, $CDCl_3$) δ: 8.47 (dd, *J* = 1.2, 8.0 Hz, 1H), 8.12 (dd, *J* = 1.2, 8.0 Hz, 1H), 7.65-7.53 (m, 2H), 7.35-7.20 (m, 3H), 7.00 (d, *J* = 7.6 Hz, 1H), 6.09 (d, *J* = 8.0 Hz, 1H), 5.64 (br, 1H), 3.13 (hept, *J* = 6.8 Hz, 2H), 2.25-2.10 (m, 1H), 1.17 (d, *J* = 6.8 Hz, 6H), 1.08 (d, *J* = 6.8 Hz, 6H), 1.00-0.92 (m, 2H), 0.70-0.62 (m, 2H); **^{13}C NMR** (100 MHz, $CDCl_3$) δ: 146.9, 142.1, 136.0, 134.4, 128.9, 127.0, 125.8, 125.6, 125.2, 124.9, 124.1, 123.8, 120.6, 106.9, 28.3, 24.9, 23.3, 13.1, 6.1; **HRMS (EI-MS)**: calculated $C_{25}H_{30}N$: 344.2373 (M+H), found: 344.2367.

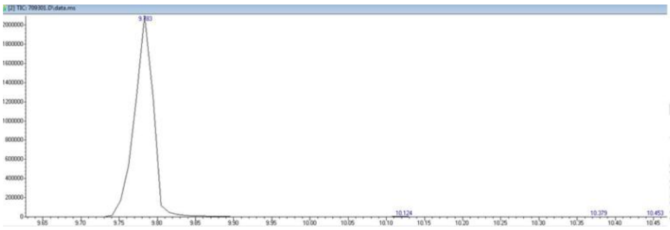

Data Path : D:\Data\hjq\7\
Data File : 709301.D
Acq On : 18 Jun 2020 09:20
Operator :
Sample : 709301

peak #	R.T. min	first scan	max scan	last scan	PK TY	peak height	corr. area	corr. % max.	% of total
26	9.783	574	583	595	rBV	2098007	3551875	100.00%	51.348%
27	10.124	612	615	623	rBV5	2855	9716	0.27%	0.140%
28	10.379	637	639	641	rVB2	1152	1527	0.04%	0.022%
29	10.453	644	646	650	rVV2	916	1595	0.04%	0.023%

Supplementary Figure 19. GCMS trace of reaction crude mixture **3cb** (**3**/**4** = peak 26: peak 27-29 > 95/5)

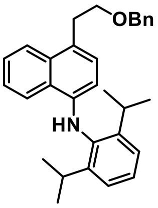

3db

Chemical Formula: $C_{31}H_{35}NO$

Dark brown liquid, 65% yield, **3**/**4**: >95/5 (determined by HNMR)

^{1}H NMR (400 MHz, $CDCl_3$) δ: 8.20-8.15 (m, 1H), 8.15-8.05 (m, 1H), 7.65-7.55 (m, 2H), 7.40-7.30 (m, 8H), 7.15-7.10 (m, 1H), 6.17 (d, *J* = 8.0 Hz, 1H), 5.69 (br, 1H), 4.59 (s, 2H), 3.83 (t, *J* = 7.6 Hz, 2H), 3.35 (t, *J* = 7.6 Hz, 2H), 3.18 (hept, *J* = 6.8 Hz, 2H), 1.22 (d, *J* = 6.8 Hz, 6H), 1.14 (d, *J* = 6.8 Hz, 6H); **^{13}C NMR** (125 MHz, $CDCl_3$) δ: 146.9, 142.4, 138.6, 135.9, 133.0, 128.5, 127.8, 127.7, 127.6, 127.1, 126.0, 124.8, 124.8, 124.4, 124.1, 123.9, 120.8, 107.1, 73.1, 71.1, 33.3, 28.3, 24.9, 23.3; **HRMS (EI-MS)**: calculated $C_{31}H_{36}NO$: 438.2791 (M+H), found: 438.2784.

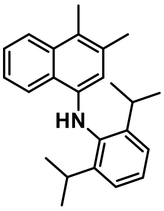

3eb

Chemical Formula: $C_{24}H_{29}N$

Dark brown liquid, 70% yield, **3**/**4**: >95/5

^{1}H NMR (400 MHz, $CDCl_3$) δ: 8.12-8.01 (m, 2H), 7.60-7.45 (m, 2H), 7.35-7.21 (m, 3H), 6.06 (s, 1H), 5.60 (br, 1H), 3.14 (hept, *J* = 6.8 Hz, 2H), 2.49 (s, 3H), 2.26 (s, 3H), 1.17 (d, *J* =6.8 Hz, 6H), 1.10 (d, *J* = 6.8 Hz, 6H); **^{13}C NMR** (100 MHz, $CDCl_3$) δ: 146.7, 141.1, 136.0, 133.9, 133.8, 126.8, 125.9, 124.8, 124.1, 123.8, 122.6, 121.1, 120.5, 110.6, 28.3, 24.9, 23.3, 21.1, 14.1; **HRMS(EI-MS)**: calculated $C_{24}H_{30}N$: 332.2373 (M+H), found: 332.2365.

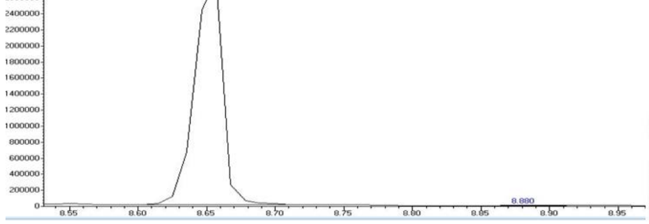

Data Path : D:\Data\hjq\7\
Data File : 708903.D
Acq On : 20 Jun 2020 12:36
Operator :
Sample : 708903

peak #	R.T. min	first scan	max scan	last scan	PK TY	peak height	corr. area	corr. % max.	% of total
20	8.657	472	477	483	rVV	2810420	4060572	100.00%	51.617%
21	8.880	495	498	501	rBV4	11051	24210	0.60%	0.308%

Supplementary Figure 20. GCMS trace of reaction crude mixture **3eb** (**3**/**4** = peak 20: peak 21 >

95/5)

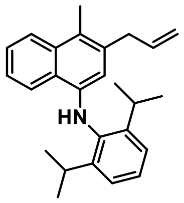

3fb

Chemical Formula: $C_{26}H_{31}N$ Dark brown liquid, 81% yield, **3**/**4**: >95/5

^{1}H NMR (500 MHz, $CDCl_3$) δ: 8.20-8.10 (m, 2H), 7.67-7.52 (m, 2H), 7.42-7.36 (m, 1H), 7.35-7.30 (m, 2H), 6.15-6.08 (m, 1H), 5.98-5.82 (m, 1H), 5.72-5.66 (m, 1H), 5.02-4.94 (m, 1H), 4.94-4.84 (m, 1H), 3.45-3.40 (m, 2H), 3.29-3.18 (m, 2H), 2.60-2.55 (m, 3H), 1.25 (d, *J* = 6.8 Hz, 6H), 1.16 (d, *J* = 6.8 Hz, 6H); **^{13}C NMR** (125 MHz, $CDCl_3$) δ:146.9, 141.5, 136.9, 135.9, 135.5, 134.0, 126.9, 126.0, 125.0, 124.2, 124.0, 122.9, 121.4, 120.5, 115.1, 110.1, 39.0, 28.3, 24.9, 23.4, 13.9; **HRMS(EI-MS)**: calculated $C_{26}H_{32}N$: 358.2529 (M+H), found: 358.2521.

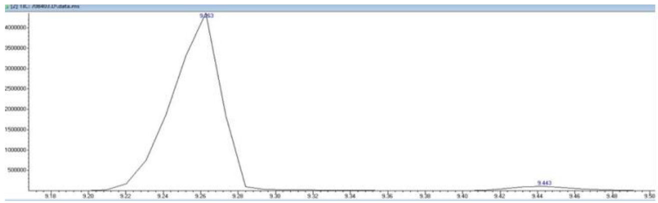

Data Path : D:\Data\hjq\7\
Data File : 708403.D
Acq On : 07 Jun 2020 17:04
Operator :
Sample : 708403

peak #	R.T. min	first scan	max scan	last scan	PK TY	peak height	corr. area	corr. % max.	% of total
35	9.263	527	534	547	rVV	4364025	8001013	100.00%	76.215%
36	9.443	547	551	562	rVB2	108771	263832	3.30%	2.513%

Supplementary Figure 21. GCMS trace of reaction crude mixture **3fb** (**3**/**4** = peak 35: peak 36 > 95/5)

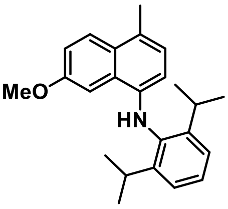

3gb

Chemical Formula: $C_{24}H_{29}NO$ Dark brown liquid, 91% yield, **3**/**4**: >95/5

^{1}H NMR (400 MHz, $CDCl_3$) δ: 7.94 (d, *J* = 9.2 Hz, 1H), 7.43 (d, *J* = 2.8 Hz, 1H), 7.32-7.21 (m, 4H), 6.90 (dd, *J* = 0.8, 7.6 Hz, 1H), 6.13 (d, *J* = 7.6 Hz, 1H), 5.36 (br, 1H), 4.00 (s, 4H), 3.12 (hept, *J* = 6.8 Hz, 2H), 2.55 (s, 3H), 1.19 (d, *J* = 6.8 Hz, 6H), 1.09 (d, *J* = 6.8 Hz, 6H); **^{13}C NMR** (100 MHz, $CDCl_3$) δ: 157.3, 146.4, 141.1, 136.5, 128.8, 127.0, 126.7, 125.4, 124.8, 124.7, 124.0, 117.1, 108.7, 100.8, 55.7, 28.3, 24.9, 23.4, 19.1; **HRMS (EI-MS)**: calculated $C_{24}H_{30}NO$: 348.2322 (M+H), found: 348.2316.

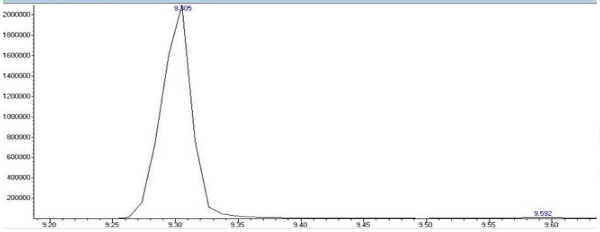

Data Path : D:\Data\hjq\7\
Data File : 7080021.D
Acq On : 02 Jun 2020 20:47
Operator :
Sample : 7080021

peak #	R.T. min	first scan	max scan	last scan	PK TY	peak height	corr. area	corr. % max.	% of total
2	9.305	531	538	551	rBV	2097273	3561653	100.00%	94.559%
3	9.592	557	565	576	rVB7	8027	28876	0.81%	0.767%

Supplementary Figure 22. GCMS trace of reaction crude mixture **3gb** (**3**/**4** = peak 2: peak 3>

95/5)

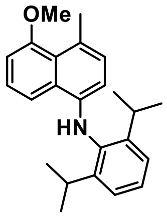

3hb

Chemical Formula: $C_{24}H_{29}NO$

Dark brown liquid, 45% yield, **3**/**4**: 80/20 (determined by HNMR)

^{1}H NMR (500 MHz, $CDCl_3$) δ: 7.69 (dd, *J* = 1.0, 8.5 Hz, 1H), 7.43 (t, *J* = 8.0 Hz, 1H), 7.32-7.27 (m, 1H), 7.26-7.22 (m, 2H), 6.90 (t, *J* = 8.0 Hz, 2H), 6.10 (d, *J* = 8.0 Hz, 1H), 5.48 (br, 1H), 3.93 (s, 3H), 3.11 (hept, *J* = 6.8 Hz, 2H), 2.78 (s, 3H), 1.17 (d, *J* = 6.8 Hz, 6H), 1.08 (d, *J* = 6.8 Hz, 6H); **^{13}C NMR** (125 MHz, $CDCl_3$) δ: 159.0, 146.7, 141.4, 136.3, 128.8, 126.8, 126.3, 126.0, 125.1, 125.1, 124.0, 113.1, 108.4, 105.8, 55.5, 28.3, 25.0, 24.9, 23.3; **HRMS(EI-MS)**: calculated $C_{24}H_{30}ON$: 348.2322 (M+H), found: 348.2321.

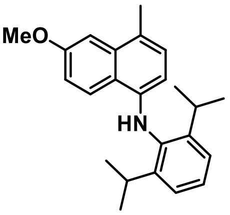

3ib

Chemical Formula: $C_{24}H_{29}NO$

Dark brown liquid, 62% yield, r.r = 2.1/1, **3**/**4**: >95/5

^{1}H NMR (400 MHz, $CDCl_3$) δ: 8.06 (d, *J* = 7.2 Hz, 1H), 7.37-7.18 (m, 5H), 7.00 (d, *J* = 8.0 Hz, 1H), 5.98 (d, *J* = 7.6 Hz, 1H), 5.56 (br, 1H), 3.98 (s, 3H), 3.14 (hept, *J* = 6.8 Hz, 2H), 2.53 (s, 3H), 1.17 (d, *J* = 6.8 Hz, 6H), 1.09 (d, *J* = 6.8 Hz, 6H); **^{13}C NMR** (100 MHz, $CDCl_3$) δ:157.7, 146.8, 142.2, 136.1, 134.8, 127.9, 126.9, 124.0, 122.9, 122.5, 119.1, 116.6, 105.6, 104.3, 55.5, 28.3, 24.9, 23.3, 19.3; **HRMS(EI-MS)**: calculated $C_{24}H_{30}NO$: 348.2322 (M+H), found: 348.2315.

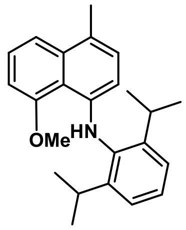

3'ib

Chemical Formula: $C_{24}H_{29}NO$

Dark brown liquid, 62% yield, r.r = 2.1/1, **3**/**4**: >95/5

^{1}H NMR (400 MHz, $CDCl_3$) δ: 8.27 (br, 1H), 7.52 (dd, *J* = 1.2, 8.4 Hz, 1H), 7.39 (t, *J* = 8.0 Hz, 1H), 7.34-7.23 (m, 3H), 6.97 (dd, *J* = 1.2, 8.0 Hz, 1H), 6.85 (dd, *J* = 1.2, 8.0 Hz, 1H), 5.94 (d, *J* = 7.6 Hz, 1H), 4.03 (s, 3H), 3.24 (hept, *J* = 6.8 Hz, 2H), 2.49 (s, 3H), 1.17 (d, *J* = 6.8 Hz, 6H), 1.13 (d, *J* = 6.8 Hz, 6H); **^{13}C NMR** (100 MHz, $CDCl_3$) δ: 158.6, 147.9, 145.0, 136.3, 136.0, 128.4,

127.1, 125.4, 124.0, 120.8, 118.1, 114.7, 105.2, 104.4, 56.3, 28.5, 25.0, 23.1, 19.8; **HRMS(EI-MS)**: calculated $C_{24}H_{30}NO$: 348.2322 (M+H), found: 348.2315.

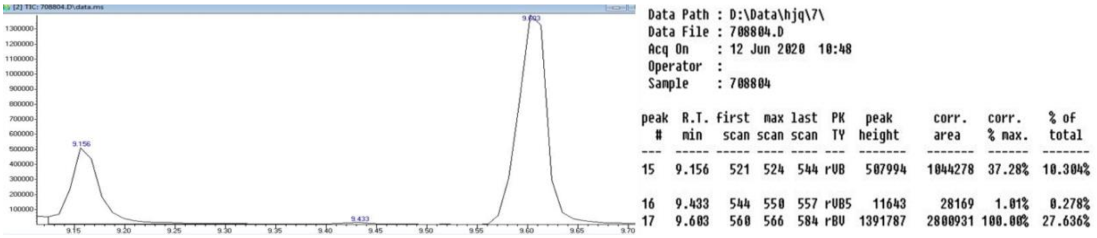

Supplementary Figure 23. GCMS trace of reaction crude mixture **3ib** and **3'ib** (**3**/**4** = peak 15+17: peak 16 > 95/5)

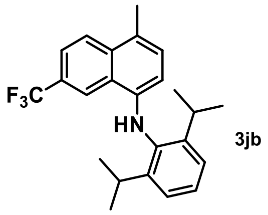

Chemical Formula: $C_{24}H_{26}F_3N$ Dark brown liquid, 57% yield, **3**/**4**: 89/11

^{1}H NMR (400 MHz, $CDCl_3$) δ: 8.41 (s, 1H), 8.10 (d, *J* = 8.8 Hz, 1H), 7.73 (dd, *J* = 0.8, 8.8 Hz, 1H), 7.36-7.30 (m, 1H), 7.29-7.24 (m, 2H), 7.14 (d, *J* = 8.0 Hz, 1H), 6.19 (d, *J* = 8.0 Hz, 1H), 5.63 (br, 1H), 3.11 (hept, *J* = 6.8 Hz, 2H), 2.59 (s, 3H), 1.19 (d, *J* = 6.8 Hz, 6H), 1.08 (d, *J* = 6.8 Hz, 6H); **^{13}C NMR** (100 MHz, $CDCl_3$) δ: 146.8, 142.9, 135.4, 134.8, 129.5, 127.3, 126.3, 124.2, 124.1, 123.5, 122.9, 121.5, 121.4, 118.6, 118.6, 108.7, 28.3, 24.8, 23.4, 19.0; **^{19}F NMR** (376 MHz, $CDCl_3$) δ: -61.7; **HRMS (EI-MS)**: calculated $C_{24}H_{27}F_3N$: 386.2090 (M+H), found: 386.2081.

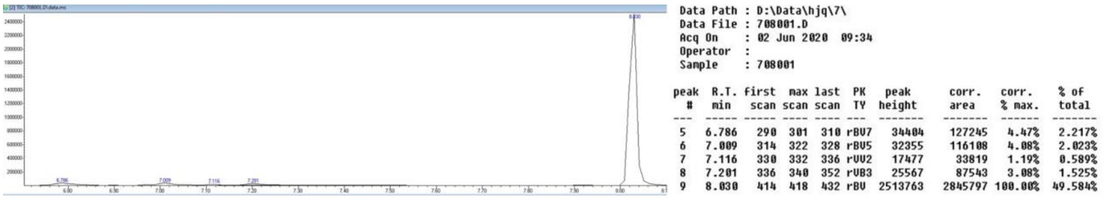

Supplementary Figure 24. GCMS trace of reaction crude mixture **3jb** (**3**/**4** = peak 9: peak (5-8) = 89/11)

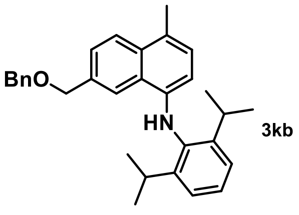

Chemical Formula: $C_{31}H_{35}NO$ Dark brown liquid, 71% yield, **3**/**4**: >95/5 (determined by HNMR)

^{1}H NMR (400 MHz, $CDCl_3$) δ: 8.18 (s, 1H), 8.08 (d, J = 8.8 Hz, 1H), 7.66 (dd, J = 1.6, 8.8 Hz, 1H), 7.55-7.30 (m, 8H), 7.09 (d, J = 7.6 Hz, 1H), 6.19 (d, J = 7.6 Hz, 1H), 5.69 (br, 1H), 4.90 (s, 2H), 4.74 (s, 2H), 3.23 (hept, J = 6.8 Hz, 2H), 2.65 (s, 2H), 1.26 (d, J = 6.8 Hz, 6H), 1.17 (d, J =

6.8 Hz, 6H); **^{13}C NMR** (100 MHz, $CDCl_3$) δ: 146.9, 142.1, 138.3, 136.0, 134.6, 133.0, 128.6, 128.0, 127.9, 127.2, 126.9, 125.8, 125.6, 124.0, 124.0, 123.8, 119.6, 107.5, 72.6, 72.3, 28.3, 24.9, 23.4, 19.1; **HRMS (EI-MS)**: calculated $C_{31}H_{36}NO$: 438.2791 (M+H), found: 438.2785.

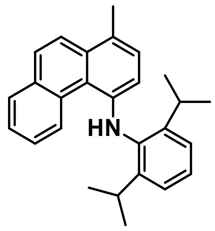

3lb

Chemical Formula: $C_{27}H_{29}N$ Dark brown liquid, 63% yield, **3**/**4**: 85/15

^{1}H NMR (400 MHz, $CDCl_3$) δ: 9.80-9.72 (m, 1H), 8.00-7.89 (m, 2H), 7.77 (d, J = 9.2 Hz, 1H), 7.61-7.55 (m, 2H), 7.33-7.25 (m, 3H), 7.15 (d, J = 8.0 Hz, 1H), 6.48 (d, J = 8.0 Hz, 1H), 5.92 (br, 1H), 3.17 (hept, J = 6.8 Hz, 2H), 2.63 (s, 3H), 1.20 (d, J = 6.8 Hz, 6H), 1.09 (d, J = 6.8 Hz, 6H); **^{13}C NMR** (100 MHz, $CDCl_3$) δ: 145.6, 144.8, 136.5, 132.8, 132.8, 131.3, 129.0, 128.2, 127.1, 126.4, 126.0, 125.7, 125.5, 125.5, 124.2, 123.9, 120.0, 112.4, 28.4, 25.0, 23.3, 19.8; **HRMS(EI-MS)**: calculated $C_{27}H_{30}N$: 368.2373 (M+H), found: 368.2373.

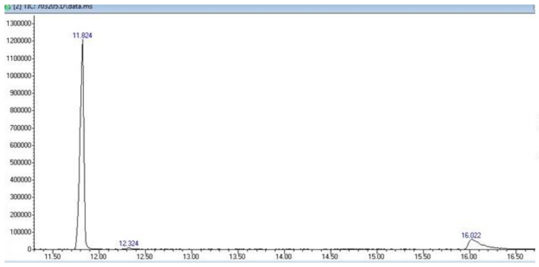

Data Path : D:\Data\hjq\7\
Data File : 703205.D
Acq On : 22 Apr 2020 12:13
Operator :
Sample : 703205

peak #	R.T. min	first scan	max scan	last scan	PK TY	peak height	corr. area	corr. % max.	% of total
5	11.824	765	775	785	rBV	1208871	3519323	100.00%	49.088%
6	12.324	811	822	830	rBV4	9325	29529	0.84%	0.412%
7	16.022	1161	1170	1199	rBV4	57709	592760	16.84%	8.268%

Supplementary Figure 25. GCMS trace of reaction crude mixture **3lb** (**3**/**4** = peak 5: peak 7 = 85/15)

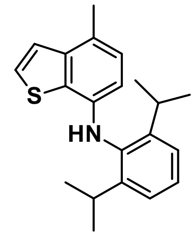

3mb

Chemical Formula: $C_{21}H_{25}NS$ Dark brown liquid, 77% yield, **3**/**4**: >95/5

^{1}H NMR (400 MHz, $CDCl_3$) δ: 7.46-7.40 (m, 2H), 7.34-7.27 (m, 1H), 7.26-7.20 (m, 2H), 6.91 (d, J = 7.6 Hz, 1H), 6.08 (d, J = 8.0 Hz, 1H), 5.05 (br, 1H), 3.16 (hept, J = 6.8 Hz, 2H), 2.52 (s, 3H), 1.13 (d, J = 6.8 Hz, 12H); **^{13}C NMR** (100 MHz, $CDCl_3$) δ: 147.2, 140.8, 140.2, 135.4, 127.3, 126.4, 126.0, 124.4, 123.9, 123.7, 123.1, 107.1, 28.4, 22.8 (br), 19.0; **HRMS(EI-MS)**: calculated $C_{21}H_{26}NS$: 324.1771 (M+H), found: 324.1781.

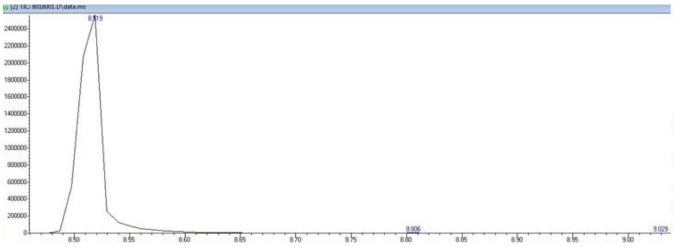

Data Path : D:\Data\hjq\
Data File : 8018001.D
Acq On : 18 Jul 2020 08:41
Operator :
Sample : 8018001

peak #	R.T. min	first scan	max scan	last scan	PK TY	peak height	corr. area	corr. % max.	% of total
11	8.519	459	464	482	rBV	2563610	3743495	100.00%	69.070%
12	8.806	487	491	497	rBV3	3701	8575	0.23%	0.158%
13	9.029	508	512	513	rBB3	268	476	0.01%	0.009%

Supplementary Figure 26. GCMS trace of reaction crude mixture **3mb** (**3**/**4** = peak 11: peak 12-13 > 95/5)

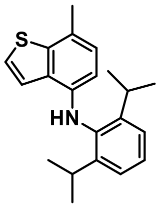

3nb

Chemical Formula: $C_{21}H_{25}NS$ Dark brown liquid, 75% yield, **3**/**4**: >95/5

^{1}H NMR (400 MHz, $CDCl_3$) δ: 7.49 (d, *J* = 5.6 Hz, 1H), 7.41 (d, *J* =5.6 Hz, 1H), 7.34-7.28 (m, 1H), 7.27-7.20 (m, 2H), 6.86 (d, *J* = 8.0 Hz, 1H), 6.03 (d, *J* = 8.0 Hz, 1H), 5.42 (br, 1H), 3.18 (hept, *J* = 6.8 Hz, 2H), 2.45 (s, 3H), 1.24-1.04 (m, 12H); **^{13}C NMR** (100 MHz, $CDCl_3$) δ: 147.3, 141.5, 141.2, 135.5, 127.4, 127.3, 125.6, 124.3, 124.0, 121.4, 119.9, 106.7, 28.3, 24.8, 23.4, 22.7, 19.7; **HRMS(EI-MS)**: calculated $C_{21}H_{26}NS$: 324.1781 (M+H), found: 324.1776.

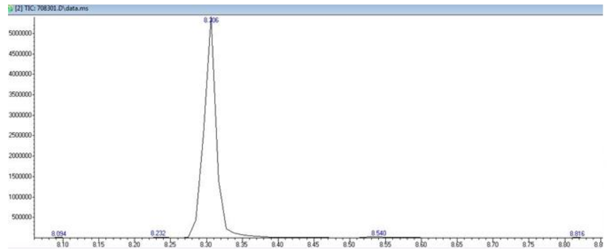

Data Path : D:\Data\hjq\7\
Data File : 708301.D
Acq On : 04 Jun 2020 22:53
Operator :
Sample : 708301

peak #	R.T. min	first scan	max scan	last scan	PK TY	peak height	corr. area	corr. % max.	% of total
10	8.094	421	424	428	rBV4	6999	10918	0.16%	0.090%
11	8.232	434	437	440	rBV3	12451	17738	0.27%	0.146%
12	8.306	440	444	462	rVV	5407417	6627710	100.00%	54.719%
13	8.540	462	466	480	rVB8	17522	71777	1.08%	0.593%
14	8.816	488	492	498	rBV6	5342	9389	0.14%	0.078%

Supplementary Figure 27. GCMS trace of reaction crude mixture **3nb** (**3**/**4** = peak 12: peak (10-11) > 95/5)

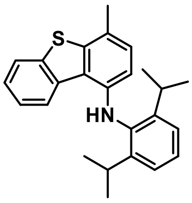

3ob

Chemical Formula: $C_{25}H_{27}NS$ Dark brown liquid, 80% yield, **3**/**4**: >95/5

^{1}H NMR (400 MHz, $CDCl_3$) δ: 8.49 (dd, *J* = 1.2, 8.0 Hz, 1H), 7.93 (dd, *J* = 1.2, 7.6 Hz, 1H), 7.52-7.40 (m, 2H), 7.34-7.24 (m, 3H), 6.98 (d, *J* = 8.0 Hz, 1H), 6.27 (d, *J* = 8.0 Hz, 1H), 5.79 (br, 1H), 3.17 (hept, *J* = 6.8 Hz, 2H), 2.46 (s, 3H), 1.20 (d, *J* = 6.8 Hz, 6H), 1.10 (d, *J* =6.8 Hz, 6H); **^{13}C NMR** (100 MHz, $CDCl_3$) δ: 146.1, 144.1, 140.8, 139.1, 136.7, 135.9, 127.6, 126.9, 125.2, 124.6, 124.1, 123.2, 123.1, 122.4, 122.3, 110.4, 28.4, 24.9, 23.2, 19.9; **HRMS(EI-MS)**: calculated $C_{25}H_{28}NS$: 374.1937 (M+H), found: 374.1930.

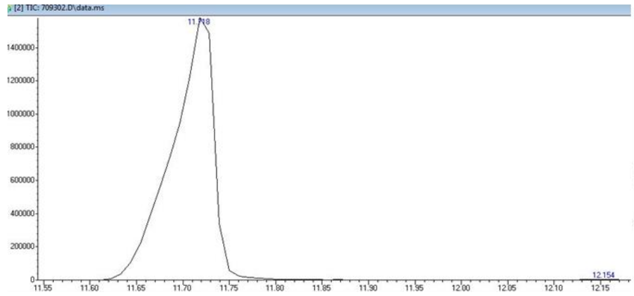

Data Path : D:\Data\hjq\7\
Data File : 709302.D
Acq On : 18 Jun 2020 09:53
Operator :
Sample : 709302

peak #	R.T. min	first scan	max scan	last scan	PK TY	peak height	corr. area	corr. % max.	% of total
8	11.718	752	765	784	rVV	1579909	4961599	100.00%	87.605%
9	12.154	799	806	812	rBV3	3382	12561	0.25%	0.222%

Supplementary Figure 28. GCMS trace of reaction crude mixture **3ob** (**3**/**4** = peak 8: peak 9 > 95/5)

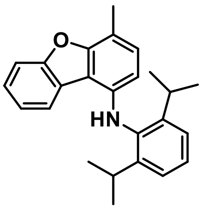

3pb

Chemical Formula: $C_{25}H_{27}NO$ Dark brown liquid, 81% yield, **3**/**4**: >95/5

^{1}H NMR (400 MHz, $CDCl_3$) δ:7.95 (d, *J* = 8.0 Hz, 1H), 7.63 (d, *J* = 8.0 Hz, 1H), 7.44 (dt, *J* = 1.2, 8.0 Hz, 1H), 7.37 (dt, *J* = 1.2, 7.2 Hz, 1H), 7.35-7.30 (m, 1H), 7.29-7.22 (m, 2H), 6.97 (d, *J* = 8.0 Hz, 1H), 6.04 (dd, *J* = 1.2, 8.0 Hz, 1H), 5.70-5.60 (br, 1H), 3.23 (hept, *J* = 6.8 Hz, 2H), 2.48 (s, 3H), 1.20 (d, *J* = 6.8 Hz, 6H), 1.13 (d, *J* = 6.8 Hz, 6H); **^{13}C NMR** (100 MHz, $CDCl_3$) δ: 156.0, 155.6, 147.0, 142.5, 135.3, 129.1, 127.4, 125.6, 124.7, 124.1, 122.7, 120.5, 111.6, 111.3, 110.3, 106.1, 28.5, 24.8, 23.3, 14.7; **HRMS(EI-MS)**: calculated $C_{25}H_{28}NO$: 358.2165 (M+H), found: 358.2158.

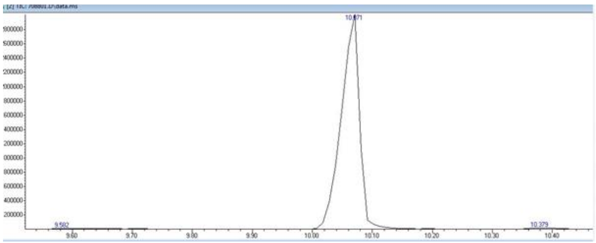

Data Path : D:\Data\hjq\7\
Data File : 708801.D
Acq On : 12 Jun 2020 09:09
Operator :
Sample : 708801

peak #	R.T. min	first scan	max scan	last scan	PK TY	peak height	corr. area	corr. % max.	% of total
13	9.582	554	564	571	rBV6	4450	26733	0.42%	0.289%
14	10.071	602	610	624	rBV	3015751	6372937	100.00%	68.910%
15	10.379	634	639	648	rVB7	14208	51971	0.82%	0.562%

Supplementary Figure 29. GCMS trace of reaction crude mixture **3pb** (**3**/**4** = peak 8: peak (5-7) > 95/5)

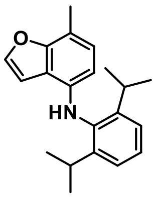

3qb

Chemical Formula: $C_{21}H_{25}NO$ Dark brown liquid, 82% yield, **3**/**4**: >95/5

^{1}H NMR (400 MHz, $CDCl_3$) δ: 7.55-7.50 (m, 1H), 7.35-7.27 (m, 1H), 7.26-7.19 (m, 2H), 6.85-6.79 (m, 1H), 6.56-6.52 (m, 1H), 6.05-5.97 (m, 1H), 5.22 (br, 1H), 3.22 (hept, *J* = 6.8 Hz, 2H), 2.40 (s, 3H), 1.13 (t, *J* = 6.8 Hz, 12H); **^{13}C NMR** (100 MHz, $CDCl_3$) δ: 155.1, 147.5, 142.9, 139.9,

135.6, 127.3, 125.9, 123.9, 114.7, 111.4, 105.1, 103.8, 28.3, 24.0, 14.6; **HRMS(EI-MS)**: calculated $C_{21}H_{26}NO$: 308.2009 (M+H), found: 308.2003.

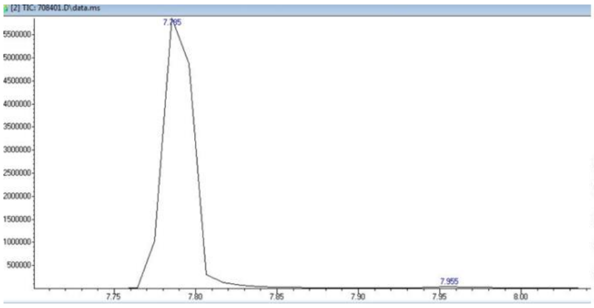

Data Path : D:\Data\hjq\7\
Data File : 708401.D
Acq On : 07 Jun 2020 15:58
Operator :
Sample : 708401

peak #	R.T. min	first scan	max scan	last scan	PK TY	peak height	corr. area	corr. % max.	% of total
12	7.785	386	395	408	rBV	5858723	7926554	100.00%	65.422%
13	7.955	408	411	424	rVB2	41631	120931	1.53%	0.998%

Supplementary Figure 30. GCMS trace of reaction crude mixture **3qb** (**3**/**4** = peak 12: peak 13 > 95/5)

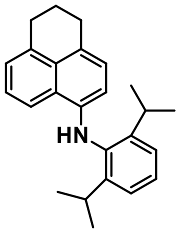

3rb

Chemical Formula: $C_{25}H_{29}N$ Dark brown liquid, 62% yield, **3**/**4**: >95/5

^{1}H NMR (500 MHz, $CDCl_3$) δ: 7.93 (dd, J = 1.0, 8.5 Hz, 1H), 7.44 (dd, J = 7.0, 8.5 Hz, 1H), 7.35-7.27 (m, 2H), 7.27-7.23 (m, 2H), 6.94 (d, J = 8.0 Hz, 1H), 6.12 (d, J = 7.5 Hz, 1H), 5.57 (br, 1H), 3.20-3.08 (m, 4H), 3.00 (t, J = 6.5 Hz, 2H), 2.05 (hept, J = 6.8 Hz, 2H), 1.18 (d, J = 6.8 Hz, 6H), 1.09 (d, J = 6.8 Hz, 6H); **^{13}C NMR** (125 MHz, $CDCl_3$) δ: 130.8, 126.9, 126.2, 124.7, 124.4, 124.3, 124.0, 123.7, 118.2, 107.2, 31.9, 31.0, 28.3, 24.9, 23.5, 23.3; **HRMS(EI-MS)**: calculated $C_{25}H_{30}N$: 344.2373 (M+H), found: 344.2368.

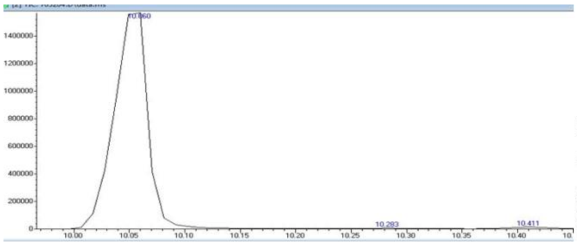

Data Path : D:\Data\hjq\7\
Data File : 703204.D
Acq On : 22 Apr 2020 11:40
Operator :
Sample : 703204

peak #	R.T. min	first scan	max scan	last scan	PK TY	peak height	corr. area	corr. % max.	% of total
29	10.060	601	609	623	rBV	1569670	3328246	100.00%	45.776%
30	10.283	627	630	637	rVB3	4459	8854	0.27%	0.122%
31	10.411	637	642	652	rBV4	12817	35748	1.07%	0.492%

Supplementary Figure 31. GCMS trace of reaction crude mixture **3rb** (**3**/**4** = peak 29: peak 30-31 > 95/5)

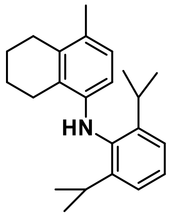

3sb

Chemical Formula: $C_{23}H_{31}N$ Dark brown liquid, 65% yield, **3**/**4**: >95/5

^{1}H NMR (400 MHz, $CDCl_3$) δ: 7.30-7.16 (m, 3H), 6.73 (d, *J* = 8.0 Hz, 1H), 5.92 (d, *J* = 8.4 Hz, 1H), 4.81 (br, 1H), 3.07 (hept, *J* = 6.8 Hz, 2H), 2.65 (t, *J* = 6.4 Hz, 4H), 2.13 (s, 3H), 1.98-1.80 (m, 4H), 1.14 (t, *J* = 6.8 Hz, 6H), 1.13 (t, *J* = 6.8 Hz, 6H); **^{13}C NMR** (100 MHz, $CDCl_3$) δ: 147.1, 143.8, 136.4, 136.0, 127.2, 126.8, 125.5, 123.8, 121.2, 108.7, 28.3, 27.6, 25.0, 24.8, 23.1, **22.91**, **22.87**, 19.1; **HRMS(EI-MS)**: calculated $C_{23}H_{32}N$: 322.2529 (M+H), found: 322.2520.

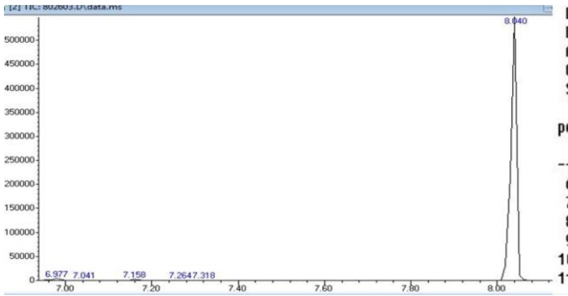

Data Path : D:\Data\hjq\
Data File : 802603.D
Acq On : 30 Jul 2020 09:55
Operator :
Sample : 802603

peak #	R.T. min	first scan	max scan	last scan	PK TY	peak height	corr. area	corr. % max.	% of total
6	6.977	314	319	323	rBB	4008	6898	1.33%	1.213%
7	7.041	324	325	328	rBB	626	689	0.13%	0.121%
8	7.158	333	336	341	rBV	2589	3715	0.72%	0.653%
9	7.264	341	346	348	rVB	354	775	0.15%	0.136%
10	7.318	349	351	354	rBB	402	578	0.11%	0.102%
11	8.040	414	419	424	rBB	548619	516762	100.00%	90.892%

Supplementary Figure 32. GCMS trace of reaction crude mixture **3sb** (**3**/**4** = peak 11: peak 6-10 > 95/5)

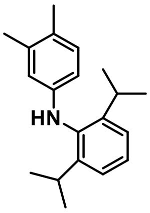

3tb

Chemical Formula: $C_{20}H_{27}N$

Colorless liquid, 62% yield, **3**/**4**: >95/5

^{1}H NMR (500 MHz, $CDCl_3$) δ: 7.30-7.24 (m, 1H), 7.22-7.18 (m, 2H), 6.88 (d, *J* = 8.0 Hz, 1H), 6.35 (s, 1H), 6.20 (dd, *J* = 2.5, 8.0 Hz, 1H), 4.99 (br, 1H), 3.19 (hept, *J* = 6.8 Hz, 2H), 2.15 (s, 3H), 2.14 (s, 3H), 1.14 (d, *J* = 6.8 Hz, 12H); **^{13}C NMR** (125 MHz, $CDCl_3$) δ: 147.4, 146.2, 137.4, 135.7, 130.3, 127.0, 125.7, 123.9, 114.8, 110.5, 28.3, 24.0, 20.1, 18.9; **HRMS(EI-MS)**: calculated $C_{20}H_{28}N$: 282.2216 (M+H), found: 282.2211.

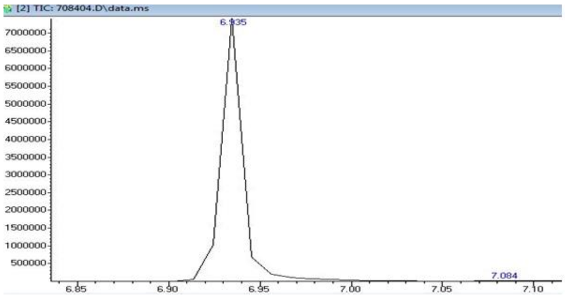

Data Path : D:\Data\hjq\7\
Data File : 708404.D
Acq On : 07 Jun 2020 17:57
Operator :
Sample : 708404

peak #	R.T. min	first scan	max scan	last scan	PK TY	peak height	corr. area	corr. % max.	% of total
11	6.935	310	315	327	rBV	7415212	6208971	100.00%	61.431%
12	7.084	327	329	338	rVB5	23821	62612	1.01%	0.619%

Supplementary Figure 33. GCMS trace of reaction crude mixture **3tb** (**3**/**4** = peak 11: peak 12 > 95/5)

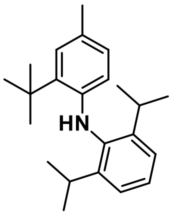

3ub

Chemical Formula: $C_{23}H_{33}N$

Colorless liquid, 55% yield, **3**/**4**: >95/5

^{1}H NMR (400 MHz, $CDCl_3$) δ: 7.30-7.20 (m, 3H), 7.16-7.10 (m, 1H), 6.74 (dt, J = 1.2, 8.4 Hz, 1H), 6.10 (dd, J = 1.6, 8.0 Hz, 1H), 5.16 (br, 1H), 3.07 (hept, J = 6.8 Hz, 2H), 2.24 (s, 3H), 1.55 (s, 9H), 1.16 (d, J = 6.8 Hz, 6H), 1.11 (d, J = 6.8 Hz, 6H); **^{13}C NMR** (100 MHz, $CDCl_3$) δ: 146.8, 143.7, 136.5, 133.2, 127.5, 127.4, 126.8, 126.6, 124.0, 114.0, 34.4, 30.0, 28.4, 25.1, 22.9, 20.9; **HRMS(EI-MS)**: calculated $C_{23}H_{34}N$: 324.2686 (M+H), found: 324.2674.

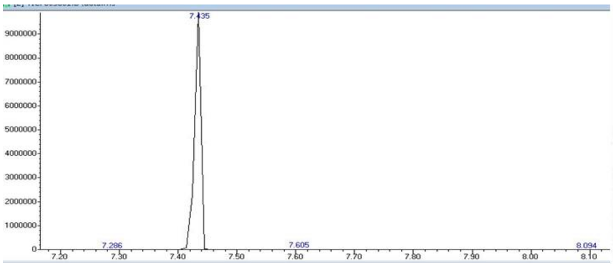

Data Path : D:\Data\hjq\
Data File : 803801.D
Acq On : 17 Aug 2020 13:14
Operator :
Sample : 803801

peak #	R.T. min	first scan	max scan	last scan	PK TY	peak height	corr. area	corr. % max.	% of total
21	7.286	346	348	350	rBB	192	122	0.00%	0.002%
22	7.435	357	362	372	rBB	9905614	7840643	100.00%	98.778%
23	7.605	372	378	383	rBB	2551	4668	0.06%	0.059%
24	8.094	422	424	428	rBB	226	358	0.00%	0.005%

Supplementary Figure 34. GCMS trace of reaction crude mixture **3ub** (**3**/**4** = peak 22: peak 21, 23-24 > 95/5)

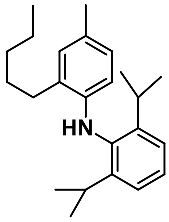

3vb

Chemical Formula: $C_{24}H_{35}N$

Colorless liquid, 85% yield, **3**/**4**: >95/5

^{1}H NMR (500 MHz, $CDCl_3$) δ: 7.32-7.18 (m, 3H), 6.98-6.91 (m, 1H), 6.80-6.70 (m, 1H), 6.10-6.00 (m, 1H), 4.92 (s, 1H), 3.15-3.04 (m, 2H), 2.70-2.60 (m, 2H), 2.28-2.20 (m, 3H), 1.80-1.70 (m, 2H), 1.50-1.40 (m, 4H), 1.20-1.08 (m, 12H), 0.99-0.89 (m, 3H); **^{13}C NMR** (125 MHz, $CDCl_3$) δ:147.1, 143.2, 136.2, 130.3, 127.3, 126.8, 126.3, 123.9, 112.1, 32.2, 31.8, 29.0, 28.4, 24.9, 23.1, 22.9, 20.6, 14.2; **HRMS(EI-MS)**: calculated $C_{24}H_{36}N$: 338.2842 (M+H), found: 338.2839.

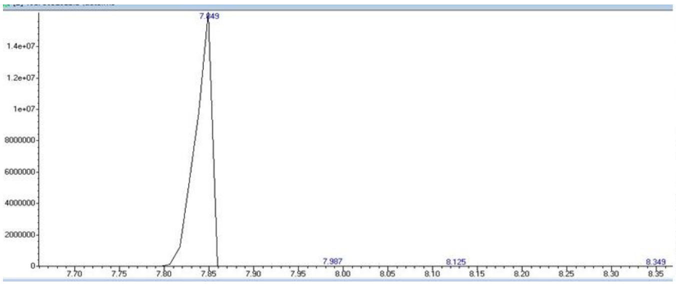

Data Path : D:\Data\hjq\
Data File : 8052011.D
Acq On : 15 Sep 2020 18:49
Operator :
Sample : 8052011

peak #	R.T. min	first scan	max scan	last scan	PK TY	peak height	corr. area	corr. % max.	% of total
20	7.849	393	401	410	rBB	16195792	20986291	100.00%	99.520%
21	7.987	410	414	422	rBB	2977	5508	0.03%	0.026%
22	8.125	425	427	429	rBB	166	106	0.00%	0.001%
23	8.349	445	448	454	rBB	371	543	0.00%	0.003%

Supplementary Figure 35. GCMS trace of reaction crude mixture **3vb** (**3**/**4** = peak 20: peak 21-23 > 95/5)

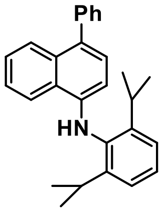

3wb

Chemical Formula: $C_{28}H_{29}N$ Dark brown liquid, 95% yield, **3**/**5**: >95/5 (determined by HNMR)

^{1}H NMR (400 MHz, $CDCl_3$) δ: 8.14 (dd, *J* = 1.2, 8.4 Hz, 1H), 8.00 (dd, *J* = 1.2, 8.4 Hz, 1H), 7.59-7.53 (m, 1H), 7.50-7.40 (m, 5H), 7.38-7.31 (m, 2H), 7.30-7.24 (m, 2H), 7.16-7.12 (m, 1H), 6.24 (d, *J* = 7.6 Hz, 1H), 5.77 (br, 1H), 3.19 (hept, *J* = 6.8 Hz, 2H), 1.21 (d, *J* = 6.8 Hz, 6H), 1.13 (d, *J* = 6.8 Hz, 6H); **^{13}C NMR** (100 MHz, $CDCl_3$) δ: 147.1, 143.0, 141.5, 135.6, 132.5, 130.6, 130.5, 128.3, 127.9, 127.3, 127.1, 126.7, 126.1, 125.0, 124.2, 123.4, 120.4, 106.9, 28.4, 25.0,23.4; **HRMS (EI-MS)**: calculated $C_{28}H_{30}N$: 380.2373 (M+H), found: 380.2376.

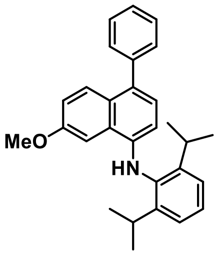

3xb

Chemical Formula: $C_{29}H_{31}NO$ Dark brown liquid, 72% yield, **3**/**5**: >95/5, **3xb**/**3'xb**: 3.2/1

^{1}H NMR (400 MHz, $CDCl_3$) δ: 7.93 (d, *J* = 9.2 Hz, 1H), 7.50-7.39 (m, 5H), 7.36-7.30 (m, 2H), 7.30-7.23 (m, 2H), 7.20-7.14 (m, 1H), 7.02 (d, *J* = 7.6 Hz, 1H), 6.27 (d, *J* = 7.6 Hz, 1H), 5.52 (br, 1H), 4.00 (s, 3H), 3.18 (hept, *J* = 6.8 Hz, 2H), 1.22 (d, *J* = 6.8 Hz, 6H), 1.13 (d, *J* = 6.8 Hz, 6H); **^{13}C NMR** (100 MHz, $CDCl_3$) δ: 157.3, 146.6, 141.9, 141.4, 135.9, 131.0, 130.3, 128.8, 128.2, 127.7, 126.9, 126.6, 125.4, 124.7, 124.0, 117.3, 108.1, 100.2, 55.6, 28.2, 24.8, 23.3; **HRMS(EI-MS)**: calculated $C_{29}H_{32}NO$: 410.2478 (M+H), found: 410.2466.

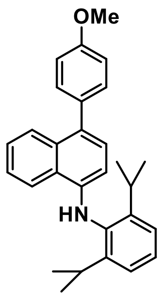

3'xb

Chemical Formula: $C_{29}H_{31}NO$ Dark brown liquid, 72% yield, **3**/**5**: >95/5

^{1}H NMR (400 MHz, $CDCl_3$) δ: 8.14 (d, *J* = 8.4 Hz, 1H), 8.00 (d, *J* = 8.8 Hz, 1H), 7.54 (t, *J* = 8.0 Hz, 1H), 7.50-7.25 (m, 6H), 7.12 (dd, *J* = 7.6, 2.0 Hz, 1H), 6.99 (dd, *J* = 8.4, 2.0 Hz, 2H), 6.24

(dd, J = 8.0, 2.0 Hz, 1H), 5.75 (br, 1H), 3.85 (s, 3H), 3.18 (hept, J = 6.8 Hz, 2H), 1.22 (d, J = 6.8 Hz, 6H), 1.13 (d, J = 6.8 Hz, 6H); ^{13}**C NMR** (100 MHz, $CDCl_3$) δ: 158.5, 147.0, 142.6, 135.6, 133.7, 132.7, 131.4, 130.1, 127.7, 127.7, 127.2, 127.0, 125.9, 124.9, 123.4, 120.3, 113.7, 106.8, 55.3, 28.2, 24.9, 23.3; **HRMS(EI-MS)**: calculated $C_{29}H_{32}NO$: 410.2478 (M+H), found: 410.2466.

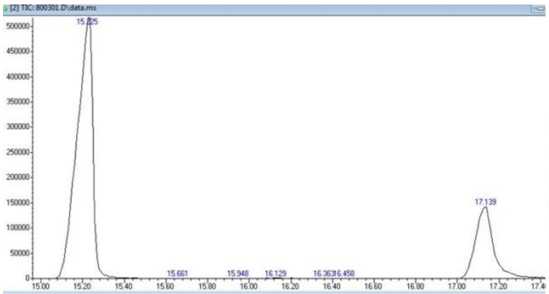

Data Path : D:\Data\hjq\
Data File : 800301.D
Acq On : 03 Jul 2020 10:26
Operator :
Sample : 800301
Misc :

peak #	R.T. min	first scan	max scan	last scan	PK TY	peak height	corr. area	corr. % max.	% of total
47	15.225	1079	1095	1114	rBV	517143	2704625	100.00%	54.163%
48	15.661	1129	1136	1140	rBB	480	1834	0.07%	0.037%
49	15.948	1162	1163	1169	rBB	500	1138	0.04%	0.023%
50	16.129	1175	1180	1190	rBV2	1235	5961	0.22%	0.119%
51	16.363	1199	1202	1206	rBV2	531	1525	0.06%	0.031%
52	16.458	1209	1211	1216	rBV	521	1682	0.06%	0.034%
53	17.139	1262	1275	1297	rBV2	141769	849444	31.41%	17.011%

Supplementary Figure 36. GCMS trace of reaction crude mixture **3xb** and **3’xb** (**3**/**5** = peak 47+53:peak 48-52 >95/5; **3/3’** = peak47:peak 53 = 3.2/1)

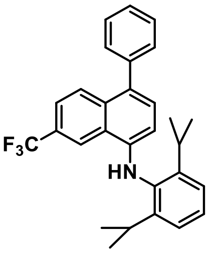

3yb

Chemical Formula: $C_{29}H_{28}F_3N$ Dark brown liquid, 82% yield, **3**/**5**: >95/5, **3yb**/**3’yb**: 1.1/1

^{1}**H NMR** (400 MHz, $CDCl_3$) δ: 8.15 (dd, J = 1.2, 8.4 Hz, 1H), 7.93 (dd, J = 1.2, 8.4 Hz, 1H), 7.70 (d, J = 8.0 Hz, 2H), 7.62-7.55 (m, 3H), 7.54-7.47 (m, 1H), 7.39-7.32 (m, 1H), 7.32-7.25 (m, 2H), 7.14 (d, J = 7.6 Hz, 1H), 6.24 (d, J = 8.0 Hz, 1H), 5.83 (br, 1H), 3.18 (hept, J = 6.8 Hz, 2H), 1.21 (d, J = 6.8 Hz, 6H), 1.13 (d, J = 6.8 Hz, 6H); ^{13}**C NMR** (100 MHz, $CDCl_3$) δ: 147.2, 145.3, 143.6, 135.3, 132.2, 130.7, 128.8, 128.2, 127.5, 126.5, 126.2, 125.3, 125.2, 124.2, 123.3, 120.5, 106.6, 28.4, 24.9, 23.4; ^{19}**F NMR** (376 MHz, $CDCl_3$) δ: -62.3; **HRMS(EI-MS)**: calculated $C_{29}H_{29}F_3N$: 448.2247 (M+H), found: 448.2235.

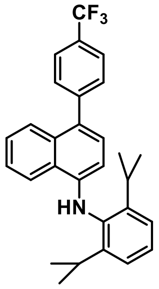

3'yb

Chemical Formula: $C_{29}H_{28}F_3N$ Dark brown liquid, 82% yield, **3**/**5**: >95/5

^{1}**H NMR** (400 MHz, $CDCl_3$) δ: 8.43 (t, J = 1.2 Hz, 1H), 8.09 (d, J = 8.8 Hz, 1H), 7.62 (dd, J = 2.0, 8.8 Hz, 1H), 7.50-7.43 (m, 4H), 7.40-7.33 (m, 2H), 7.30-7.25 (m, 3H), 6.33 (d, J = 8.0 Hz, 1H), 5.81 (br, 1H), 3.17 (hept, J = 6.8 Hz, 2H), 1.22 (d, J = 6.8 Hz, 6H), 1.13 (d, J = 6.8 Hz, 6H); ^{13}**C NMR** (100 MHz, $CDCl_3$) δ: 147.1, 143.9, 140.7, 134.9, 133.9, 130.5, 130.4, 130.1, 128.5,

128.5, 128.4, 128.2, 127.7, 127.1, 126.3, 124.3, 121.7, 113.0, 108.2, 28.4, 24.9, 23.5; ^{19}**F NMR** (376 MHz, $CDCl_3$) δ: -61.8; **HRMS(EI-MS)**: calculated $C_{29}H_{29}F_3N$: 448.2247 (M+H), found: 448.2235.

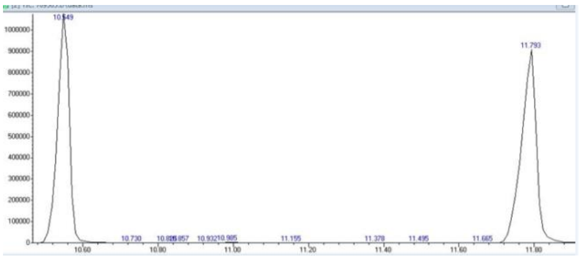

Data Path : D:\Data\hjq\7\
Data File : 709303.D
Acq On : 18 Jun 2020 10:27
Operator :
Sample : 709303

peak #	R.T. min	first scan	max scan	last scan	PK TY	peak height	corr. area	corr. % max.	% of total
23	10.549	648	655	670	rVV	1077064	2333662	93.39%	38.357%
24	10.730	670	672	674	rVB	667	1035	0.04%	0.017%
25	10.825	677	681	682	rBV	533	1035	0.04%	0.017%
26	10.857	682	684	689	rVB	1048	2421	0.10%	0.040%
27	10.932	689	691	693	rBV	643	1199	0.05%	0.020%
28	10.985	693	696	704	rVB3	3450	9000	0.36%	0.148%
29	11.155	706	712	714	rBV	701	1609	0.06%	0.026%
30	11.378	732	733	739	rBV	557	1339	0.05%	0.022%
31	11.495	742	744	749	rVB	619	1444	0.06%	0.024%
32	11.665	753	760	762	rBV2	1109	3740	0.15%	0.061%
33	11.793	762	772	787	rVV	908196	2498740	100.00%	41.070%

Supplementary Figure 37. GCMS trace of reaction crude mixture **3yb** and **3'yb** (**3**/**5** = peak 23+33: peak 24-32 > 95/5; **3/3'** = peak 33: peak 23 = 1.1/1)

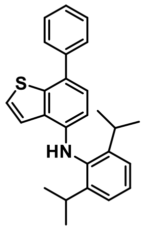

3zb

Chemical Formula: $C_{26}H_{27}NS$ Dark brown liquid, 75% yield, **3**/**5**: >95/5, **3zb**/**3'zb**: 3.4/1

^{1}**H NMR** (500 MHz, $CDCl_3$) δ: 7.72-7.67 (m, 2H), 7.51 (d, J = 5.5 Hz, 1H), 7.45-7.37 (m, 3H), 7.36-7.28 (m, 2H), 7.28-7.23 (m, 2H), 7.13 (d, J = 7.5 Hz, 1H), 6.17 (d, J = 8.0 Hz, 1H), 5.57 (br, 1H), 3.22 (hept, J = 6.8 Hz, 2H), 1.25-1.09 (m, 12H); ^{13}**C NMR** (100 MHz, $CDCl_3$) δ: 147.5, 142.6, 141.1, 140.0, 135.0, 128.8, 128.1, 127.9, 127.6, 127.1, 126.6, 126.0, 124.7, 124.1, 119.4, 107.0, 28.5, 24.9, 23.5; **HRMS(EI-MS)**: calculated $C_{26}H_{28}NS$: 386.1937 (M+H), found: 386.1930.

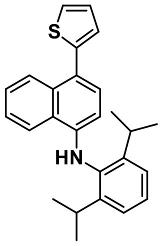

3'zb

Chemical Formula: $C_{26}H_{27}NS$ Dark brown liquid, 75% yield, **3**/**5**: >95/5

^{1}**H NMR** (400 MHz, $CDCl_3$) δ: 8.26 (dd, J = 2.0, 7.6 Hz, 1H), 8.12 (dd, J = 2.0, 8.0 Hz, 1H), 7.60-7.50 (m, 2H), 7.37-7.26 (m, 5H), 7.17-7.11 (m, 2H), 6.20 (d, J = 8.0 Hz, 1H), 5.82 (br, 1H), 3.17 (hept, J = 6.8 Hz, 2H), 1.20 (d, J = 6.8 Hz, 6H), 1.12 (d, J = 6.8 Hz, 6H); ^{13}**C NMR** (100 MHz, $CDCl_3$) δ: 147.2, 143.7, 143.0, 135.3, 133.0, 129.3, 127.5, 127.2, 126.9, 126.5, 125.3, 124.9, 124.2, 123.4, 123.3, 122.3, 120.3, 106.4, 28.4, 24.9, 23.4; **HRMS(EI-MS)**: calculated $C_{26}H_{28}NS$: 386.1937 (M+H), found: 386.1930.

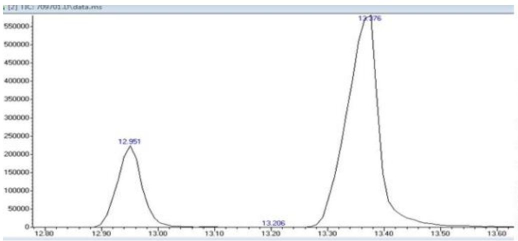

Data Path : D:\Data\hjq\7\
Data File : 709701.D
Acq On : 28 Jun 2020 17:03
Operator :
Sample : 709701

peak #	R.T. min	first scan	max scan	last scan	PK TY	peak height	corr. area	corr. % max.	% of total
30	12.951	874	881	892	rBV	224261	677191	29.16%	16.932%
31	13.206	902	905	908	rBV	613	1264	0.05%	0.032%
32	13.376	909	921	940	rBV	581317	2322335	100.00%	58.066%

Supplementary Figure 38. GCMS trace of reaction crude mixture **3zb** and **3'zb** (**3**/**5** = peak 30+32: peak 31 > 95/5; **3**/**3'** = peak32:peak 30 = 3.4/1)

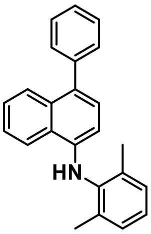

3wa

Chemical Formula: $C_{24}H_{21}N$ Dark brown liquid, 90% yield, **3**/**5w**: >95/5 (determined by HNMR)

^{1}H NMR (400 MHz, $CDCl_3$) δ: 8.14 (dd, J = 1.2, 8.0 Hz, 1H), 7.98 (dd, J = 1.2, 8.0 Hz, 1H), 7.57-7.49 (m, 1H), 7.48-7.40 (m, 5H), 7.37-7.30 (m, 1H), 7.20-7.07 (m, 4H), 6.28 (d, J = 8.0 Hz, 1H), 5.73 (br, 1H), 2.23 (s, 6H); **^{13}C NMR** (100 MHz, $CDCl_3$) δ: 141.4, 140.9, 138.8, 135.2, 132.6, 131.3, 130.5, 128.9, 128.3, 127.8, 127.0, 126.7, 126.1, 125.7, 125.1, 124.2, 120.7, 107.2, 18.4; **HRMS(EI-MS)**: calculated $C_{24}H_{22}N$: 324.1747 (M+H), found: 324.1743.

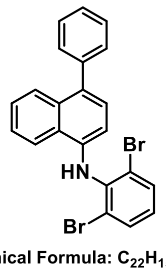

3wc

Chemical Formula: $C_{22}H_{15}Br_2N$ Dark brown liquid, 30% yield, **3**/**5w**: 80/20 (determined by HNMR)

^{1}H NMR (500 MHz, $CDCl_3$) δ: 8.27 (d, J = 8.5 Hz, 1H), 7.97 (d, J = 8.5 Hz, 1H), 7.63 (d, J = 8.5 Hz, 2H), 7.62-7.56 (m, 1H), 7.52-7.43 (m, 5H), 7.41-7.36 (m, 1H), 7.25-7.21 (m, 1H); 6.94 (t, J =

8.0 Hz, 1H), 6.56 (d, J = 8.0 Hz, 1H), 6.20 (br, 1H); **^{13}C NMR** (125 MHz, $CDCl_3$) δ: 141.0, 140.0, 138.6, 134.3, 133.1, 132.6, 130.4, 128.3, 127.0, 127.0, 126.9, 126.4, 126.3, 126.2, 125.7, 121.6, 121.0, 111.5; **HRMS(EI-MS)**: calculated $C_{22}H_{16}Br_2N$: 451.9644 (M+H), found: 451.9634.

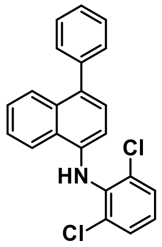

3wd

Chemical Formula: $C_{22}H_{15}Cl_2N$ Dark brown liquid, 65% yield, **3**/**5w**: 72/28 (determined by HNMR)

^{1}H NMR (400 MHz, $CDCl_3$) δ: 8.27 (dd, J = 2.0, 8.0 Hz, 1H), 7.96 (dd, J = 2.0, 8.0 Hz, 1H), 7.62-7.53 (m, 1H), 7.52-7.43 (m, 5H), 7.40-7.35 (m, 3H), 7.27-7.22 (m, 1H), 7.05 (t, J = 8.0 Hz, 1H), 6.63-6.56 (m, 1H), 6.22 (br, 1H); **^{13}C NMR** (100 MHz, $CDCl_3$) δ: 141.0, 138.4, 137.7, 134.5, 132.6, 130.4, 130.3, 129.1, 128.3, 127.0, 126.9, 126.9, 126.3, 126.2, 125.8, 124.9, 121.6, 111.7; **HRMS(EI-MS)**: calculated $C_{22}H_{16}NCl_2$: 364.0654 (M+H), found: 364.0647.

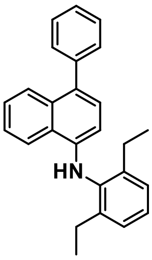

3we

Chemical Formula: $C_{26}H_{25}N$ Dark brown liquid, 95% yield, **3**/**5w**: >95/5 (determined by HNMR)

^{1}H NMR (500 MHz, $CDCl_3$) δ: 8.14 (dd, J = 1.0, 8.5 Hz, 1H), 7.99 (dd, J = 1.0, 8.5 Hz, 1H), 7.57-7.50 (m, 1H), 7.50-7.40 (m, 5H), 7.38-7.31 (m, 1H), 7.27-7.20 (m, 3H), 7.14 (d, J = 8.0 Hz, 1H), 6.26 (d, J = 7.5 Hz, 1H), 5.78 (br, 1H), 2.73-2.60 (m, 2H), 2.60-2.50 (m, 2H), 1.17 (t, J = 7.5 Hz, 6H); **^{13}C NMR** (125 MHz, $CDCl_3$) δ: 142.0, 141.7, 141.4, 137.4, 132.6, 130.9, 130.5, 128.3, 127.8, 127.0, 126.9, 126.7, 126.5, 126.0, 125.1, 123.9, 120.5, 107.0, 24.7, 14.9; **HRMS(EI-MS)**: calculated $C_{26}H_{26}N$: 352.2060 (M+H), found: 352.2062.

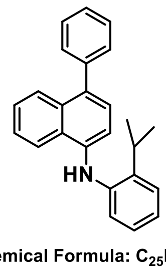

3wf

Chemical Formula: $C_{25}H_{23}N$ Dark brown liquid, 22% yield, **3**/**5w**: 28/72 (determined by HNMR)

^{1}H NMR (400 MHz, $CDCl_3$) δ: 8.08 (dd, J = 1.6, 9.2 Hz, 1H), 7.96 (dd, J = 1.2, 8.4 Hz, 1H), 7.52-7.45 (m, 5H), 7.42-7.35 (m, 2H), 7.30-7.25 (m, 2H), 7.15-7.12 (m, 1H), 7.10-7.07 (m, 2H),

7.04-7.00 (m, 1H), 5.90 (br, 1H), 3.24 (hept, *J* = 6.8 Hz, 1H), 1.32 (d, *J* = 6.8 Hz, 6H); **^{13}C NMR** (125 MHz, $CDCl_3$) δ: 141.2, 140.9, 140.5, 139.7, 133.8, 132.7, 130.4, 128.4, 127.5, 127.0, 126.9, 126.8, 126.6, 126.3, 126.2, 125.5, 123.1, 121.6, 121.5, 112.9, 28.0, 23.2; **HRMS(EI-MS)**: calculated $C_{25}H_{24}N$: 338.1903 (M+H), found: 338.1903.

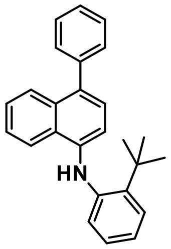

3wg

Chemical Formula: $C_{26}H_{25}N$ Dark brown liquid, 60% yield, **3**/**5w**: 92/8 (determined by HNMR)

^{1}H NMR (400 MHz, $CDCl_3$) δ: 8.05 (dd, *J* = 1.6, 8.4 Hz, 1H), 7.97 (dd, *J* = 1.2, 8.0 Hz, 1H), 7.54-7.41 (m, 7H), 7.41-7.35 (m, 1H), 7.27 (d, *J* = 8.0 Hz, 1H), 7.22-7.12 (m, 2H), 7.08-7.02 (m, 1H), 7.00 (d, *J* = 7.6 Hz, 1H), 6.06 (br, 1H), 1.52 (s, 9H); **^{13}C NMR** (100 MHz, $CDCl_3$) δ: 142.2, 141.7, 141.2, 140.9, 133.0, 132.7, 130.4, 128.4, 127.6, 127.2, 127.1, 127.0, 126.9, 126.3, 126.2, 125.5, 124.7, 123.2, 121.3, 112.1, 35.0, 30.8; **HRMS(EI-MS)**: calculated $C_{26}H_{26}N$: 352.2060 (M+H), found: 352.2058.

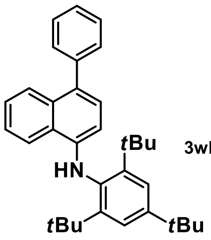

3wh

Chemical Formula: $C_{34}H_{41}N$ Dark brown liquid, 80% yield, **3**/**5w**: 94/6 (determined by HNMR)

^{1}H NMR (400 MHz, $CDCl_3$) δ: 8.02 (d, *J* = 8.4 Hz, 1H), 7.99 (dd, *J* = 1.2, 8.8 Hz, 1H), 7.55-7.48 (m, 3H), 7.47-7.40 (m, 5H), 7.36-7.30 (m, 1H), 7.10 (d, *J* = 7.6 Hz, 1H), 5.93 (br, 1H), 5.86 (d, *J* = 8.0 Hz, 1H), 1.39 (s, 9H), 1.34 (s, 18H); **^{13}C NMR** (100 MHz, $CDCl_3$) δ: 149.2, 148.4, 145.1, 141.7, 135.5, 132.4, 130.6, 129.1, 128.2, 127.8, 126.9, 126.4, 125.8, 124.8, 123.9, 122.9, 120.3, 107.8, 36.9, 35.1, 32.6, 31.7; **HRMS(EI-MS)**: calculated $C_{34}H_{42}N$: 464.3312 (M+H), found: 464.3307.

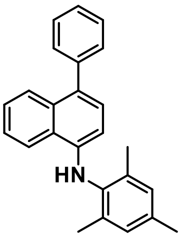

3wi

Chemical Formula: $C_{25}H_{23}N$ Dark brown liquid, 84% yield, **3**/**5w**: >95/5 (determined by HNMR)

^{1}H NMR (400 MHz, $CDCl_3$) δ: 8.16-8.07 (m, 1H), 8.01-7.90 (m, 1H), 7.58-7.30 (m, 7H), 7.19-7.10 (m, 1H), 6.99 (s, 2H), 6.30-6.20 (m, 1H), 5.71-5.65 (br, 1H), 2.33 (s, 3H), 2.20 (s, 6H); **^{13}C NMR** (100 MHz, $CDCl_3$) δ: 141.5, 141.2, 136.0, 135.4, 132.6, 130.9, 130.5, 129.5, 128.3, 127.9, 127.0, 126.7, 126.0, 125.0, 124.0, 120.7, 106.7, 21.1, 18.2; **HRMS(EI-MS)**: calculated $C_{25}H_{24}N$: 338.1903 (M+H), found: 338.1905.

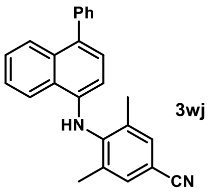

3wj

Chemical Formula: $C_{25}H_{20}N_2$ Dark brown liquid, 81% yield, **3**/**5w**: >95/5 (determined by HNMR)

^{1}H NMR (400 MHz, $CDCl_3$) δ: 8.26 (dd, *J* = 1.2, 8.0 Hz, 1H), 8.02 (dd, *J* = 1.6, 8.4 Hz, 1H), 7.68-7.58 (m, 1H), 7.58-7.40 (m, 8H), 7.25 (d, *J* = 7.6 Hz, 1H), 6.46 (d, *J* = 7.6 Hz, 1H), 5.87 (s, 1H), 2.27 (s, 6H); **^{13}C NMR** (100 MHz, $CDCl_3$) δ: 144.4, 140.9, 139.0, 133.90, 133.87, 132.70, 132.68, 130.3, 128.4, 127.09, 127.05, 126.5, 125.8, 125.7, 121.2, 119.5, 110.5, 107.3, 18.6; **HRMS(EI-MS)**: calculated $C_{25}H_{21}N_2$: 349.1699 (M+H), found: 349.1691

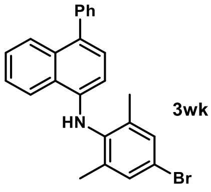

3wk

Chemical Formula: $C_{24}H_{20}BrN$ Dark brown liquid, 75% yield, **3**/**5w**: 90/10 (determined by HNMR)

^{1}H NMR (400 MHz, $CDCl_3$) δ: 8.20 (dd, *J* = 1.2, 8.4 Hz, 1H), 8.04 (dd, *J* = 1.2, 8.4 Hz, 1H), 7.65-7.57 (m, 1H), 7.58-7.48 (m, 5H), 7.47-7.40 (m, 1H), 7.38 (s, 2H), 7.23 (d, *J* = 8.0 Hz, 1H), 6.34 (d, *J* = 7.6 Hz, 1H), 5.71 (s, 1H), 2.27 (s, 6H); **^{13}C NMR** (100 MHz, $CDCl_3$) δ: 141.2, 140.3, 138.0, 137.3, 132.6, 131.8, 131.6, 130.4, 128.3, 127.6, 127.0, 126.8, 126.2, 125.3, 124.3, 120.7, 118.6, 107.5, 18.2; **HRMS(EI-MS)**: calculated $C_{24}H_{21}NBr$: 402.0852 (M+H), found: 402.0837

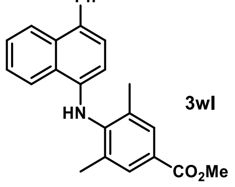

3wl

Chemical Formula: $C_{26}H_{23}NO_2$ Dark brown liquid, 65% yield, **3**/**5w**: 72/28 (determined by HNMR)

^{1}H NMR (400 MHz, $CDCl_3$) δ: 8.26 (d, *J* = 8.4 Hz, 1H), 8.03 (d, *J* = 8.0 Hz, 1H), 7.91 (s, 2H), 7.65-7.57 (m, 1H), 7.57-7.45 (m, 5H), 7.45-7.38 (m, 1H), 7.24 (d, *J* = 7.6 Hz, 1H), 6.44 (d, *J* = 7.6 Hz, 1H), 5.90 (s, 1H), 3.97 (s, 3H), 2.31 (6H); **^{13}C NMR** (100 MHz, $CDCl_3$) δ: 167.4, 144.1, 141.0, 139.5, 133.2, 133.0, 132.6, 130.4, 128.3, 127.3, 126.98, 126.94, 126.3, 125.9, 125.5, 125.3, 121.2, 109.7, 52.1, 18.6;**HRMS(EI-MS)**: calculated $C_{26}H_{24}NO_2$: 382.1802 (M+H), found:

382.1793

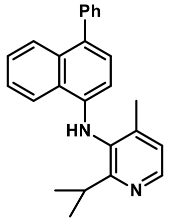

3wm

Chemical Formula: $C_{25}H_{24}N_2$ Dark brown liquid, 71% yield, **3**/**5w**: >95/5 (determined by HNMR)

^{1}H NMR (400 MHz, $CDCl_3$) δ: 8.43 (d, J = 4.8 Hz, 1H), 8.15 (dd, J = 1.2, 8.8 Hz, 1H), 8.00 (d, J = 1.2, 8.4 Hz, 1H), 7.60-7.30 (m, 7H), 7.17 (d, J = 7.6 Hz, 1H), 7.10 (d, J = 4.8 Hz, 1H), 6.23 (d, J = 7.6 Hz, 1H), 5.77 (br, 1H), 3.36 (hept, J = 6.8 Hz, 1H), 2.22 (s, 3H), 1.35-1.11 (m, 6H); **^{13}C NMR** (100 MHz, $CDCl_3$) δ: 164.1, 146.5, 145.1, 141.0, 140.9, 133.7, 132.6, 131.8, 130.3, 128.2, 127.5, 127.0, 126.8, 126.2, 125.3, 124.0, 123.8, 120.4, 107.3, 30.0, 22.4 (br), 18.0; **HRMS(EI-MS)**: calculated $C_{25}H_{25}N_2$: 353.2012 (M+H), found: 353.2001.

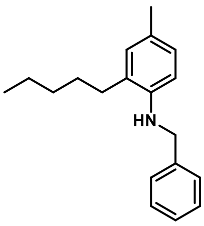

3vn

Chemical Formula: $C_{19}H_{25}N$ Colorless liquid, 29% yield, **3**/**4**: >95/5

^{1}H NMR (400 MHz, $CDCl_3$) δ: 7.45-7.22 (m, 5H), 6.93-6.85 (m, 2H), 6.58-6.49 (m, 1H), 4.35 (s, 2H), 4.05-3.60 (br, 1H), 2.45 (t, J = 6.8 Hz, 2H), 2.23 (s, 3H), 1.68-1.55 (m, 2H), 1.40-1.30 (m, 4H), 0.90 (t, J = 6.8 Hz, 3H); **^{13}C NMR** (100 MHz, $CDCl_3$) δ: 143.4, 140.0, 130.0, 128.7, 127.6, 127.4, 127.2, 126.8, 126.5, 110.8, 48.8, 32.1, 31.3, 28.6, 22.7, 20.6, 14.2; **HRMS(EI-MS)**: calculated $C_{19}H_{26}N$: 268.2060 (M+H), found: 268.2058.

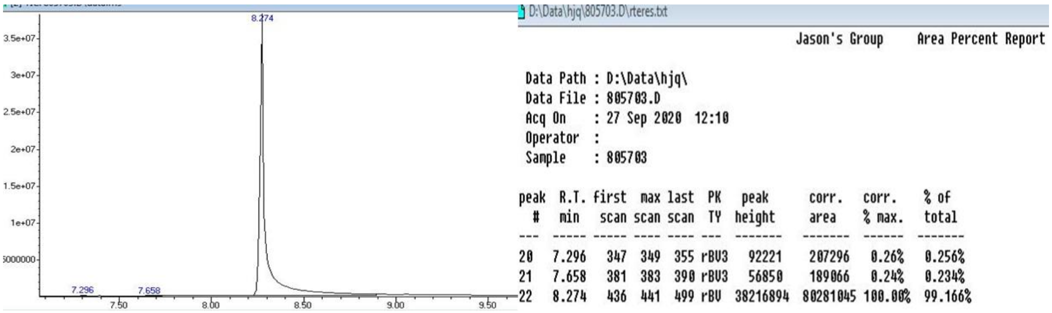

peak #	R.T. min	first scan	max scan	last scan	PK TY	peak height	corr. area	corr. % max.	% of total
20	7.296	347	349	355	rBV3	92221	207296	0.26%	0.256%
21	7.658	381	383	390	rBV3	56850	189066	0.24%	0.234%
22	8.274	436	441	499	rBV	38216894	80281045	100.00%	99.166%

Supplementary Figure 39. GCMS trace of reaction crude mixture **3vn** (**3**/**4** = peak 22: peak 20-21 > 95/5)

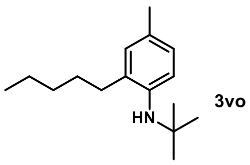

Chemical Formula: $C_{16}H_{27}N$ Colorless liquid, 81% yield, **3**/**4**: >95/5

^{1}H NMR (500 MHz, $CDCl_3$) δ: 6.91-6.80 (m, 3H), 3.60-2.75 (br, 1H), 2.45-2.38 (m, 2H), 2.22 (s, 3H), 1.60-1.50 (m, 2H), 1.40-1.30 (m, 13H), 0.90 (t, *J* = 6.8 Hz, 3H); **^{13}C NMR** (125 MHz, $CDCl_3$) δ: 142.1, 130.3, 129.6, 127.2, 126.9, 116.6, 51.6, 32.1, 31.9, 30.5, 29.2, 22.7, 20.6, 14.2; **HRMS(EI-MS)**: calculated $C_{16}H_{28}N$: 234.2216 (M+H), found: 234.2214.

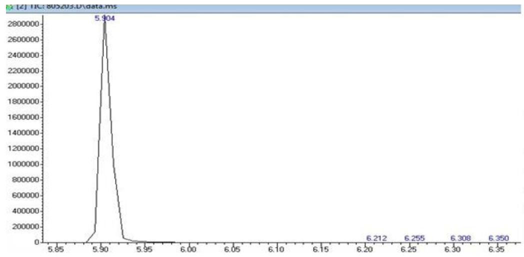

Data Path : D:\Data\hjq\
Data File : 805203.D
Acq On : 15 Sep 2020 19:55
Operator :
Sample : 805203

peak #	R.T. min	first scan	max scan	last scan	PK TY	peak height	corr. area	corr. % max.	% of total
12	5.904	215	218	233	rBV	2919291	2664219	100.00%	99.566%
13	6.212	246	247	248	rBB	204	130	0.00%	0.005%
14	6.255	249	251	253	rBB	195	228	0.01%	0.009%
15	6.308	254	256	258	rBB	159	101	0.00%	0.004%
16	6.350	259	260	262	rBB	171	109	0.00%	0.004%

Supplementary Figure 40. GCMS trace of reaction crude mixture **3vo** (**3**/**4** = peak 12: peak 13-16 > 95/5)

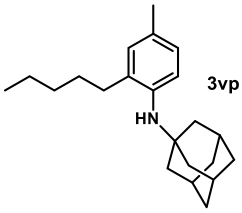

Chemical Formula: $C_{22}H_{33}N$ Colorless liquid, 71% yield, **3**/**4**: >95/5

^{1}H NMR (500 MHz, $CDCl_3$) δ: 6.94-6.82 (m, 3H), 3.25-2.90 (br, 1H), 2.46 (t, *J* = 6.8 Hz, 2H), 2.23 (s, 3H), 2.14-2.04 (m, 3H), 1.92-1.85 (m, 5H), 1.74-1.60 (m, 6H), 1.59-1.50 (m, 2H), 1.40-1.34 (m, 4H), 0.91 (t, *J* = 6.8 Hz, 3H); **^{13}C NMR** (125 MHz, $CDCl_3$) δ: 141.2, 130.6, 130.3, 128.1, 126.7, 119.1, 52.5, 43.9, 36.6, 32.1, 32.0, 29.9, 29.5, 22.7, 20.7, 14.2; **HRMS(EI-MS)**: calculated $C_{22}H_{34}N$: 312.2686 (M+H), found: 312.2684.

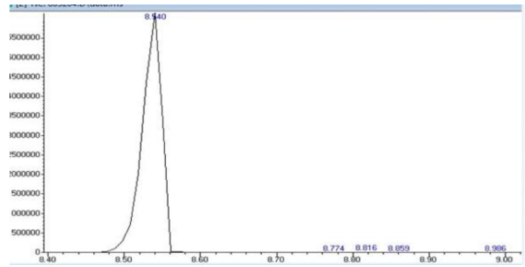

Data Path : D:\Data\hjq\
Data File : 805204.D
Acq On : 15 Sep 2020 20:28
Operator :
Sample : 805204

peak #	R.T. min	first scan	max scan	last scan	PK TY	peak height	corr. area	corr. % max.	% of total
29	8.540	457	466	479	rBV	6139594	10865227	100.00%	98.257%
30	8.774	486	488	489	rBB	160	198	0.00%	0.002%
31	8.816	489	492	494	rBB	382	527	0.00%	0.005%
32	8.859	495	496	498	rBB	163	104	0.00%	0.001%
33	8.986	506	508	510	rBB	193	123	0.00%	0.001%

Supplementary Figure 41. GCMS trace of reaction crude mixture **3vp** (**3**/**4** = peak 29: peak 30-33 > 95/5)

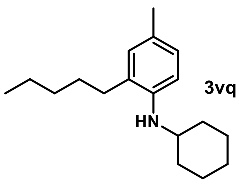

3vq

Chemical Formula: $C_{18}H_{29}N$ Colorless liquid, 78% yield, **3**/**4**: >95/5

^{1}H NMR (500 MHz, $CDCl_3$) δ: 6.88 (d, *J* = 8.5 Hz, 1H), 6.84 (s, 1H), 6.56 (d, *J* = 8.5 Hz, 1H), 3.33-3.22 (m, 1H), 3.55-2.95 (br, 1H), 2.39 (t, *J* = 6.8 Hz, 2H), 2.22 (s, 3H), 2.10-2.00 (m, 2H), 1.80-1.70 (m, 2H), 1.68-1.53 (m, 3H), 1.40-1.33 (m, 6H), 1.30-1.12 (m, 3H), 0.91 (t, *J* = 6.8 Hz, 3H); **^{13}C NMR** (100 MHz, $CDCl_3$) δ: 142.5, 130.1, 127.3, 126.6, 125.6, 111.3, 51.9, 33.8, 32.1, 31.4, 28.6, 26.2, 25.1, 22.7, 20.5, 14.2; **HRMS(EI-MS)**: calculated $C_{18}H_{30}N$: 260.2373 (M+H), found: 260.2370.

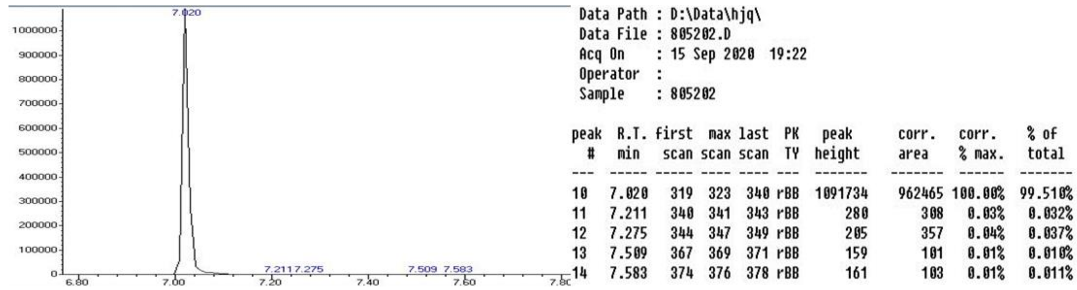

Data Path : D:\Data\hjq\
Data File : 805202.D
Acq On : 15 Sep 2020 19:22
Operator :
Sample : 805202

peak #	R.T. min	first scan	max scan	last scan	PK TY	peak height	corr. area	corr. % max.	% of total
10	7.020	319	323	340	rBB	1091734	962465	100.00%	99.510%
11	7.211	340	341	343	rBB	280	308	0.03%	0.032%
12	7.275	344	347	349	rBB	205	357	0.04%	0.037%
13	7.509	367	369	371	rBB	159	101	0.01%	0.010%
14	7.583	374	376	378	rBB	161	103	0.01%	0.011%

Supplementary Figure 42. GCMS trace of reaction crude mixture **3vq** (**3**/**4** = peak 10: peak 11-14 > 95/5)

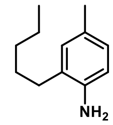

3'vr & 3'vs

Chemical Formula: $C_{12}H_{19}N$ Colorless liquid, 71% yield for **3vr** & 80% yield for **3vs**. **3**/**4**: >95/5

^{1}H NMR (500 MHz, $CDCl_3$) δ: 6.85 (s, 1H), 6.83 (d, *J* = 8.0 Hz, 1H), 6.58 (d, *J* = 8.0 Hz, 1H), 3.48 (br, 2H), 2.45 (t, *J* = 6.8 Hz, 2H), 2.23 (s, 3H), 1.65-1.55 (m, 2H), 1.42-1.30 (m, 4H), 0.95-0.88 (m, 3H); **^{13}C NMR** (125 MHz, $CDCl_3$) δ: 141.6, 130.2, 128.0, 127.3, 127.3, 115.8, 32.1, 31.5, 28.8, 22.7, 20.6, 14.2; **HRMS(EI-MS)**: calculated $C_{12}H_{20}N$: 178.1590 (M+H), found: 178.1587.

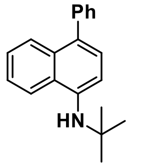

3wo

Chemical Formula: $C_{20}H_{21}N$ Dark brown liquid, 27% yield.

^{1}H NMR (500 MHz, $CDCl_3$) δ: 7.98-7.88 (m, 2H), 7.55-7.37 (m, 7H), 7.35-7.30 (m, 1H), 7.03 (dd, *J* = 2.0, 7.5 Hz, 1H), 1.55 (s, 9H); **^{13}C NMR** (100 MHz, $CDCl_3$) δ: 141.5, 141.4, 132.7, 130.5, 130.1, 128.3, 127.4, 126.9, 126.6, 125.7, 125.4, 124.7, 120.7, 109.4, 51.9, 30.1; **HRMS(EI-MS)**: calculated $C_{20}H_{22}N$: 276.1747 (M+H), found: 276.1740.

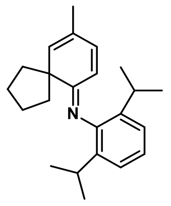

8bb

Chemical Formula: $C_{23}H_{31}N$ Colorless liquid, 81% yield.

^{1}H NMR (400 MHz, $CDCl_3$) δ: 7.09-6.91 (m, 3H), 6.16-6.08 (m, 1H), 5.86-5.80 (m, 1H), 5.72-5.66 (m, 1H), 2.65 (hept, *J* = 6.8 Hz, 2H), 2.28-2.12 (m, 2H), 2.02-1.90 (m, 2H), 1.85-1.71 (m, 7H), 1.07 (d, *J* = 6.8 Hz, 6H), 1.00 (d, *J* = 6.8 Hz, 6H); **^{13}C NMR** (100 MHz, $CDCl_3$) δ: 172.7, 146.8, 138.9, 137.1, 137.0, 125.0, 123.0, 122.8, 118.5, 53.0, 42.5, 28.0, 25.8, 23.3, 23.2, 21.1; **HRMS(EI-MS)**: calculated $C_{23}H_{32}N$: 322.2529 (M+H), found: 322.2520.

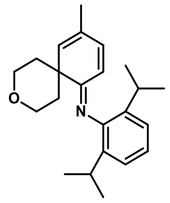

8cb

Chemical Formula: $C_{23}H_{31}NO$ Colorless liquid, 73% yield.

^{1}H NMR (500 MHz, $CDCl_3$) δ: 7.14-7.06 (m, 2H), 7.05-7.00 (m, 1H), 6.41 (s, 1H), 6.15 (dd, *J* = 2.0, 8.0 Hz, 1H), 5.77 (d, *J* = 8.0 Hz, 1H), 4.05-3.95 (m, 2H), 3.85 (dt, *J* = 2.5, 11.5 Hz, 2H), 2.70 (hept, *J* = 6.8 Hz, 2H), 2.44 (dt, *J* = 4.5 Hz, 11.5 Hz, 2H), 1.91 (s, 3H), 1.61 (dd, *J* = 2.5, 13.5 Hz, 2H), 1.14 (d, *J* = 7.0 Hz, 6H), 1.06 (d, *J* = 7.0 Hz, 6H); **^{13}C NMR** (125 MHz, $CDCl_3$) δ: 171.1, 146.4, 136.6, 135.9, 133.7, 128.2, 123.1, 122.8, 118.7, 63.6, 42.3, 36.8, 28.0, 23.20, 23.15, 21.7; **HRMS(EI-MS)**: calculated $C_{23}H_{32}NO$: 338.2478 (M+H), found: 338.2470

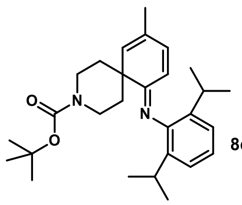

8db

Chemical Formula: $C_{28}H_{40}N_2O_2$ Colorless liquid, 85% yield.

^{1}H NMR (400 MHz, $CDCl_3$) δ: 7.15-7.09 (m, 2H), 7.07-7.00 (m, 1H), 6.30 (s, 1H), 6.18 (dd, *J* = 1.6, 9.6 Hz, 1H), 5.79 (d, *J* = 10.0 Hz, 1H), 4.25-4.00 (m, 2H), 3.32-3.10 (m, 2H), 2.71 (hept, *J* = 6.8 Hz, 2H), 2.35-2.15 (m, 2H), 1.93 (s, 3H), 1.80-1.62 (m, 2H), 1.51 (s, 9H), 1.16 (d, *J* = 6.8 Hz, 6H), 1.07 (d, *J* = 6.8 Hz, 6H); **^{13}C NMR** (100 MHz, $CDCl_3$) δ: 171.2, 155.1, 146.2, 136.6, 136.0, 133.1, 128.4, 123.2, 122.8, 118.7, 79.6, 43.0, 39.9, 39.1, 36.8, 35.8, 28.6, 27.9, 23.24, 23.19, 21.7; **HRMS(EI-MS)**: calculated $C_{28}H_{41}N_2O_2$: 437.3168 (M+H), found: 437.3153

Isolated indene byproduct from general procedures.

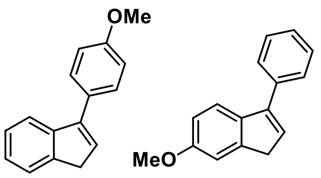

1/1

5x and 5'x

Chemical Formula: $C_{16}H_{14}O$

Colorless liquid, isolated as byproduct

^{1}H NMR (400 MHz, $CDCl_3$) δ: 7.62-7.40 (m, 4H), 7.39-7.28 (m, 1H), 7.20-6.78 (m, 3H), 6.53-6.41 (m, 1H), 3.85 (s, 3H), 3.84 (s, 3H), 3.51-3.44 (m, 2H); **^{13}C NMR** (100 MHz, $CDCl_3$) δ: 159.3, 158.1, 144.9, 144.8, 144.7, 144.3, 137.2, 136.4, 130.0, 130.0, 128.9, 128.8, 128.7, 127.7, 127.6, 126.2, 124.9, 124.2, 120.8, 120.4, 114.1, 113.8, 111.9, 110.7, 55.7, 55.5, 38.3, 38.2; **HRMS(EI-MS)**: calculated $C_{16}H_{15}O$: 223.1117 (M+H), found: 223.1112.

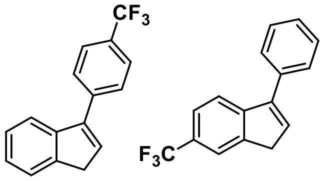

1/1

5y and 5'y

Chemical Formula: $C_{16}H_{11}F_3$

Colorless liquid, isolated as byproduct

^{1}H NMR (400 MHz, $CDCl_3$) δ (mixtures) 7.80-7.25 (m, 8H), 6.75-6.60 (m, 1H), 3.58-3.50 (m, 2H); **^{13}C NMR** (100 MHz, $CDCl_3$) δ: (mixtures) 147.4, 145.1, 144.9, 144.8, 144.2, 143.4, 139.9, 135.5, 133.8, 132.6, 128.9, 128.1, 127.8, 127.3, 127.0, 126.5, 125.7, 125.4, 124.4, 123.7, 121.0, 120.4, 120.2, 38.5, 38.4; **^{19}F NMR** (376 MHz, $CDCl_3$) δ: (mixtures) -61.4, -62.4; **HRMS(EI-MS)**: calculated $C_{16}H_{12}F_3$: 261.0886 (M+H), found: 261.0879.

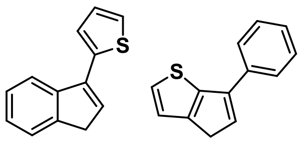

3.9/1

5z and 5'z

Chemical Formula: $C_{13}H_{10}S$

Colorless liquid, isolated as byproduct

^{1}H NMR (400 MHz, $CDCl_3$) δ: 7.85-7.72 (m, 1H), 7.55-7.25 (m, 5H), 7.16-7.10 (m, 1H), 6.79-6.66 (m, 1H), 3.55-3.37 (m, 2H); **^{13}C NMR** (125 MHz, $CDCl_3$) δ: (major) 144.7, 143.2, 138.1, 138.0, 131.1, 127.6, 126.5, 125.3, 124.6, 124.5, 124.2, 120.6, 38.3; (minor) 148.3, 144.3, 141.3, 134.6, 129.1, 128.9, 128.0, 126.2, 125.7, 122.4; **HRMS(EI-MS)**: calculated $C_{13}H_{11}S$: 199.0576 (M+H), found: 199.0571.

3. Supplementary Figures

3.1 Crystal structures of **3aa** and [*trans*-IPr-Ni(CN-DIPP)Br_2]

Supplementary Table 3. Crystal data and structure refinement for **3aa**.

Identification code	cxy1036_0m
Empirical formula	$C_{19}H_{19}N$
Formula weight	261.35
Temperature/K	100(2)
Crystal system	monoclinic
Space group	$P2_1/c$
a/Å	14.2640(6)
b/Å	7.6286(3)
c/Å	14.9302(6)
α/°	90
β/°	116.1840(10)
γ/°	90
Volume/Å^3	1457.90(10)
Z	4
ρ_{calc}g/cm^3	1.191
μ/mm^{-1}	0.069
F(000)	560.0
Crystal size/mm^3	0.41 × 0.36 × 0.35
Radiation	MoKα (λ = 0.71073)
2Θ range for data collection/°	6.082 to 56.748
Index ranges	$-19 \le h \le 19$, $-10 \le k \le 10$, $-17 \le l \le 19$
Reflections collected	21338
Independent reflections	3621 [R_{int} = 0.0496, R_{sigma} = 0.0341]
Data/restraints/parameters	3621/0/187
Goodness-of-fit on F^2	1.056
Final R indexes [I>=2σ (I)]	R_1 = 0.0461, wR_2 = 0.1136
Final R indexes [all data]	R_1 = 0.0633, wR_2 = 0.1232
Largest diff. peak/hole / e Å^{-3}	0.30/-0.24

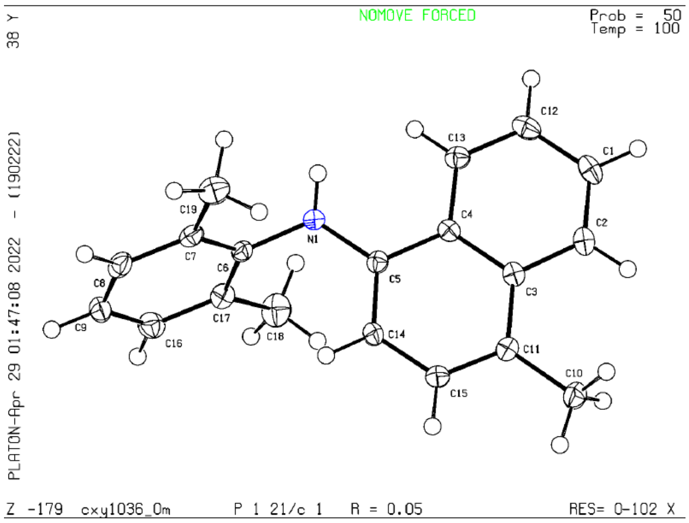

Supplementary Figure 43. ORTEP drawing of **3aa**. **CCDC2036407**

Supplementary Table 4. Crystal data and structure refinement for [*trans*-IPr-Ni(CN-DIPP)Br_2].

Identification code	cxy1295_0m
Empirical formula	$C_{40}H_{53}Br_2N_3Ni$
Formula weight	794.38
Temperature/K	298(2)
Crystal system	triclinic
Space group	P-1
a/Å	10.556(4)
b/Å	14.070(6)
c/Å	15.988(6)
α/°	100.724(13)
β/°	107.806(14)
γ/°	109.977(13)
Volume/Å^3	2009.7(14)
Z	2
ρ_{calc}g/cm^3	1.313
μ/mm^{-1}	2.500
F(000)	824.0
Crystal size/mm^3	0.41 × 0.38 × 0.34
Radiation	MoKα (λ = 0.71073)
2Θ range for data collection/ °	4.48 to 56.702
Index ranges	$-14 \leq h \leq 14$, $-18 \leq k \leq 18$, $-21 \leq l \leq 21$
Reflections collected	40062
Independent reflections	9978 [R_{int} = 0.0443, R_{sigma} = 0.0408]
Data/restraints/parameters	9978/0/428
Goodness-of-fit on F^2	1.022
Final R indexes [I>=2σ (I)]	R_1 = 0.0378, wR_2 = 0.0817
Final R indexes [all data]	R_1 = 0.0668, wR_2 = 0.0940
Largest diff. peak/hole / e Å^{-3}	0.68/-0.65

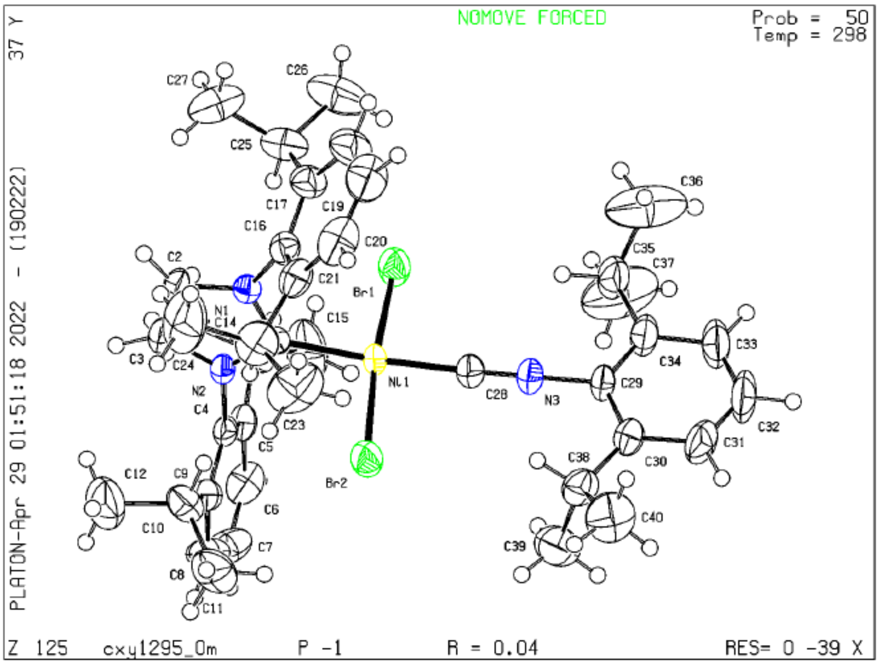

Supplementary Figure 44. ORTEP drawing of [*trans*-IPr-Ni(CN-DIPP)Br_2]. **CCDC: 2036409**

3.2 NMR spectra

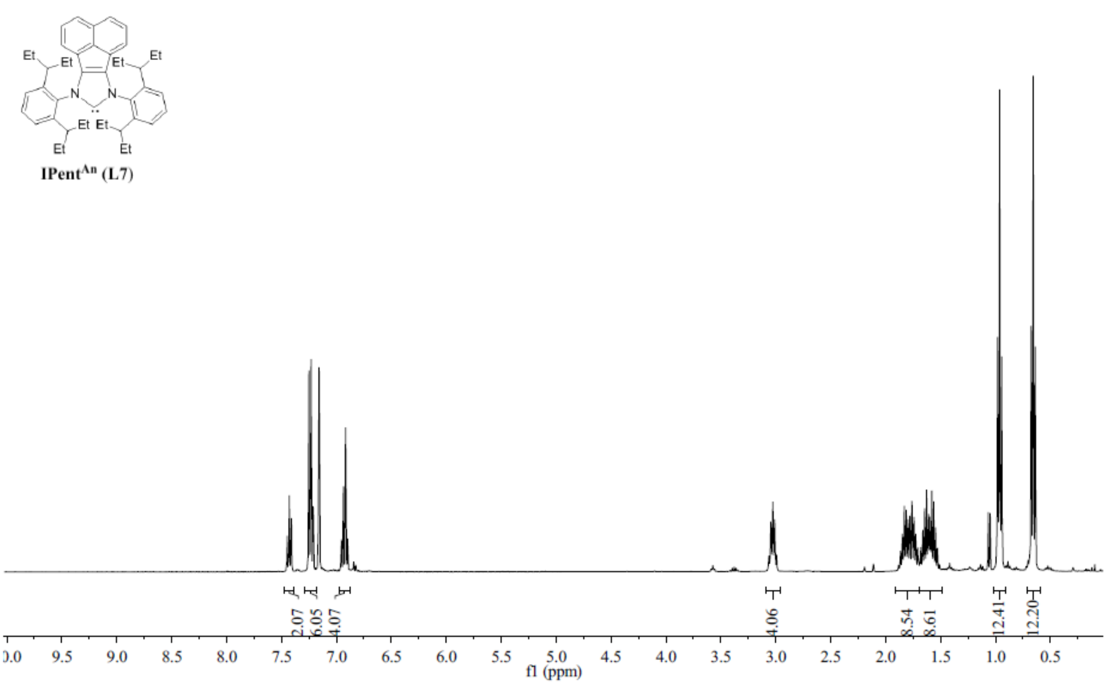

Supplementary Figure 45. ^{1}H-NMR of compound **L7**, recorded at 400 MHz and 25 °C in C_6D_6.

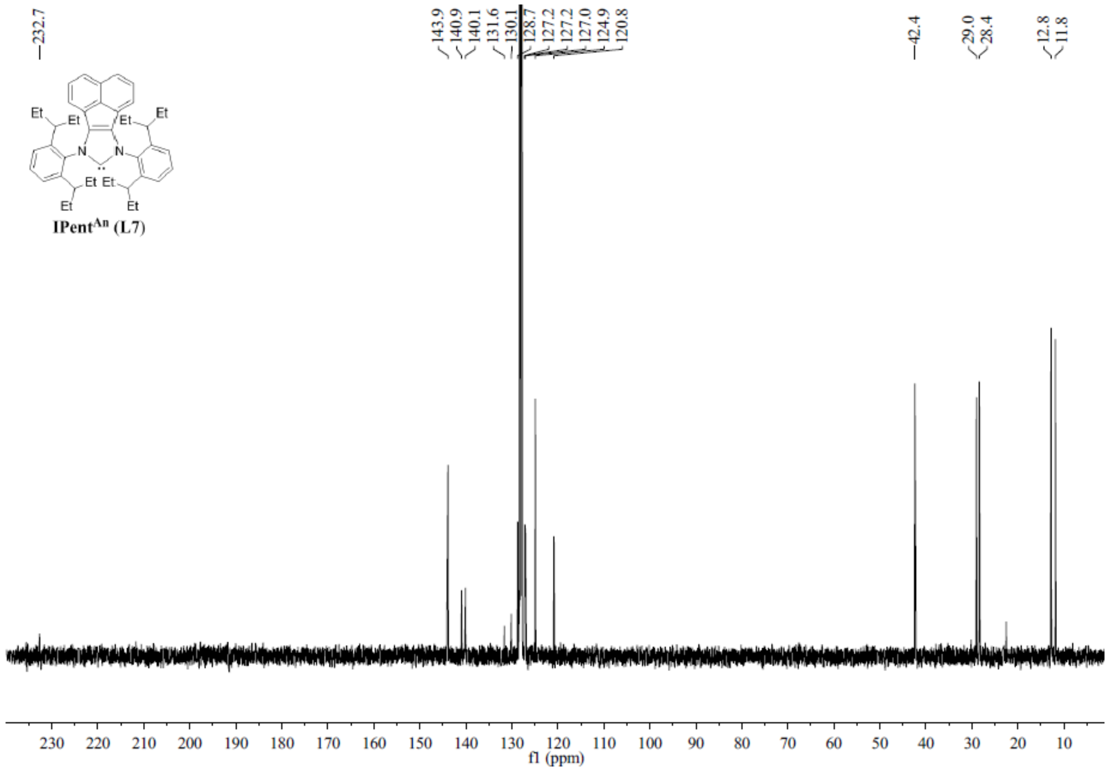

Supplementary Figure 46. ^{13}C-NMR of compound **L7**, recorded at 100 MHz and 25 °C in C_6D_6.

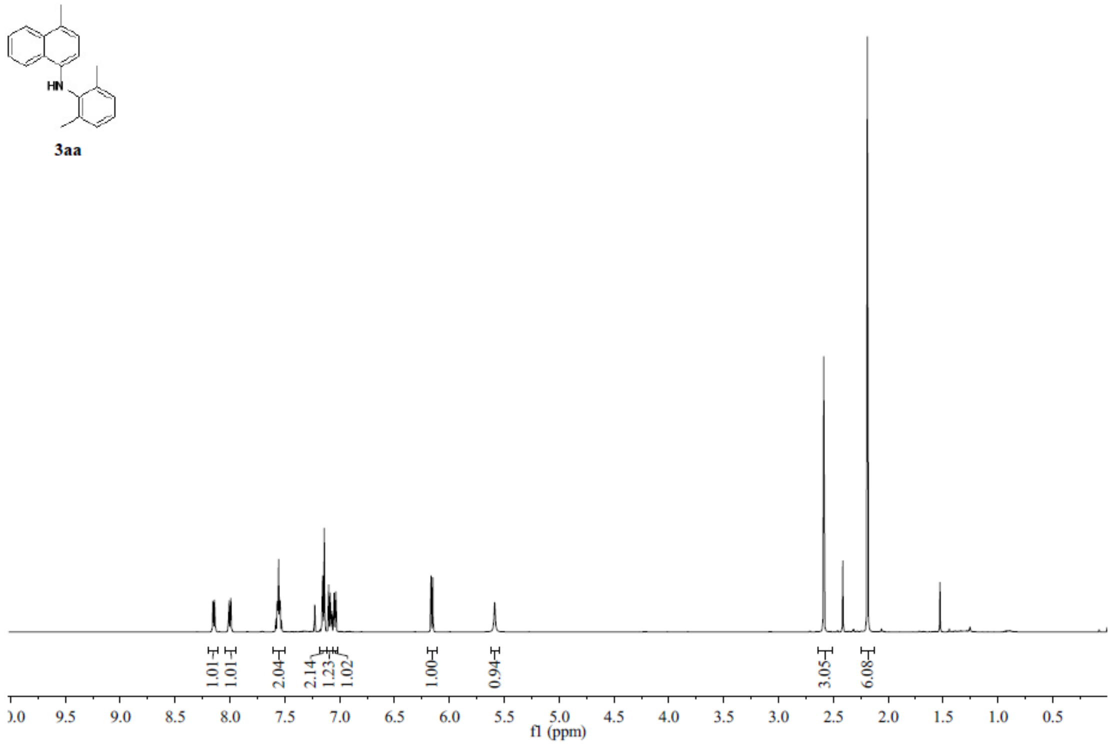

Supplementary Figure 47. ^{1}H-NMR of compound **3aa**, recorded at 500 MHz and 25 °C in $CDCl_3$.

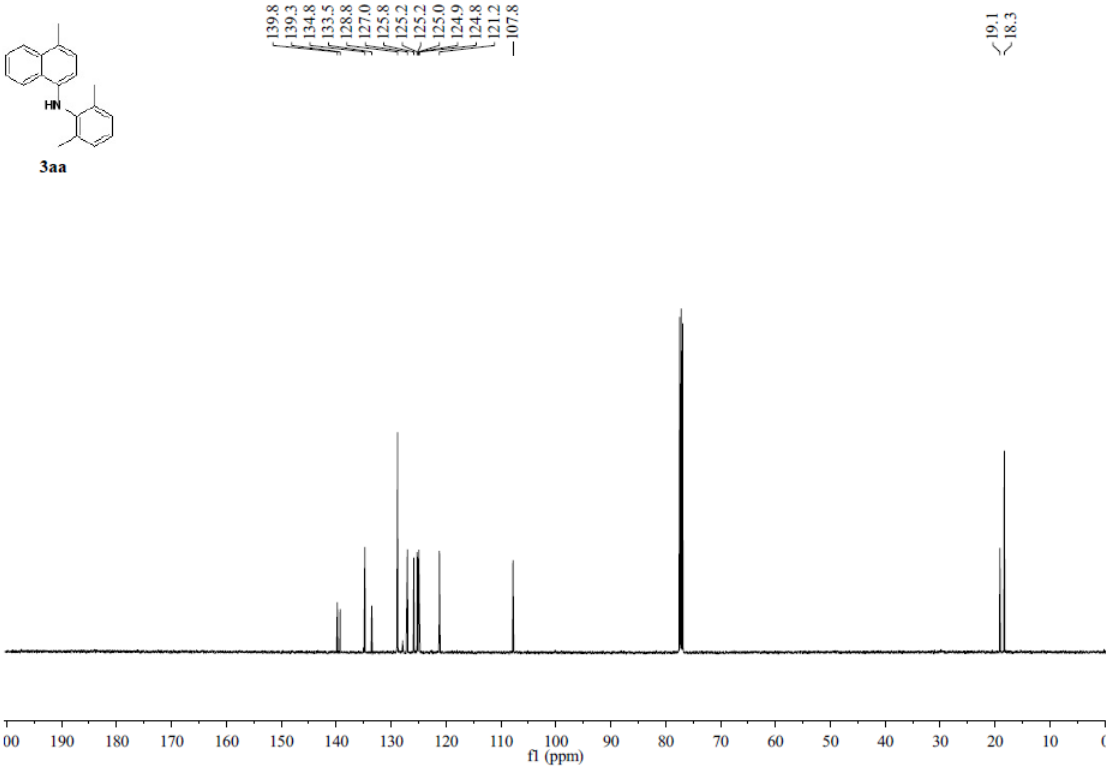

Supplementary Figure 48. ^{13}C-NMR of compound **3aa**, recorded at 125 MHz and 25 °C in $CDCl_3$.

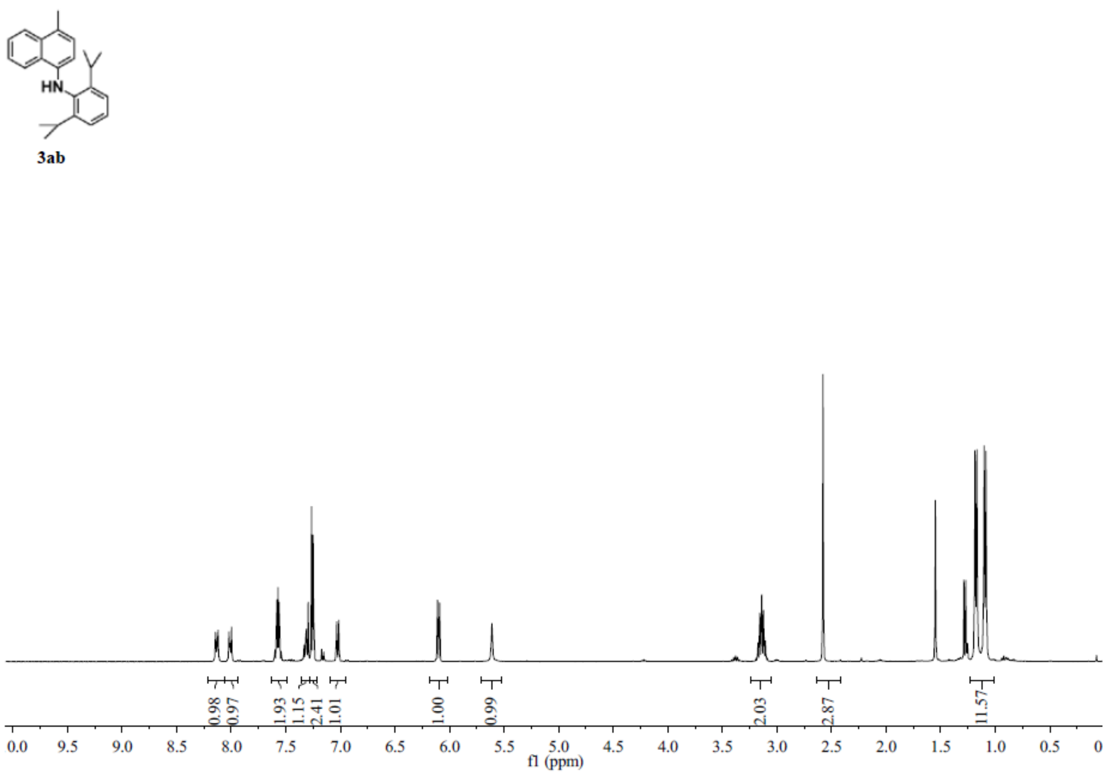

Supplementary Figure 49. ^{1}H-NMR of compound **3ab**, recorded at 400 MHz and 25 °C in $CDCl_3$.

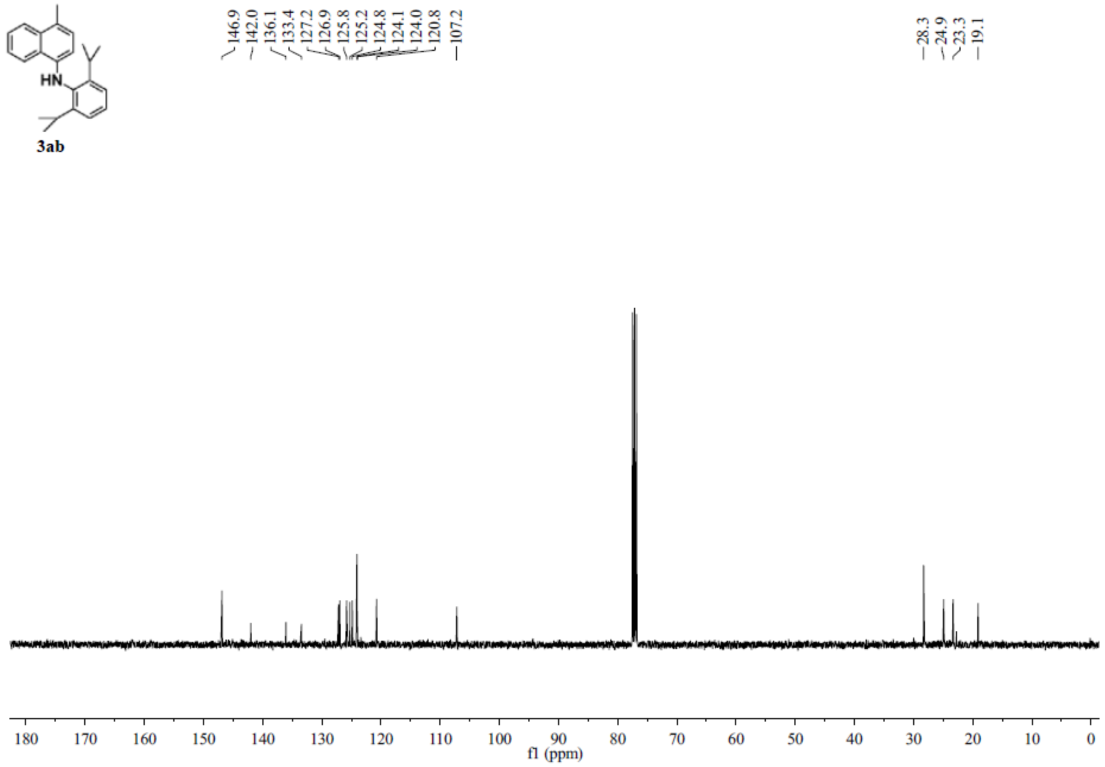

Supplementary Figure 50. ^{13}C-NMR of compound **3ab**, recorded at 100 MHz and 25 °C in $CDCl_3$.

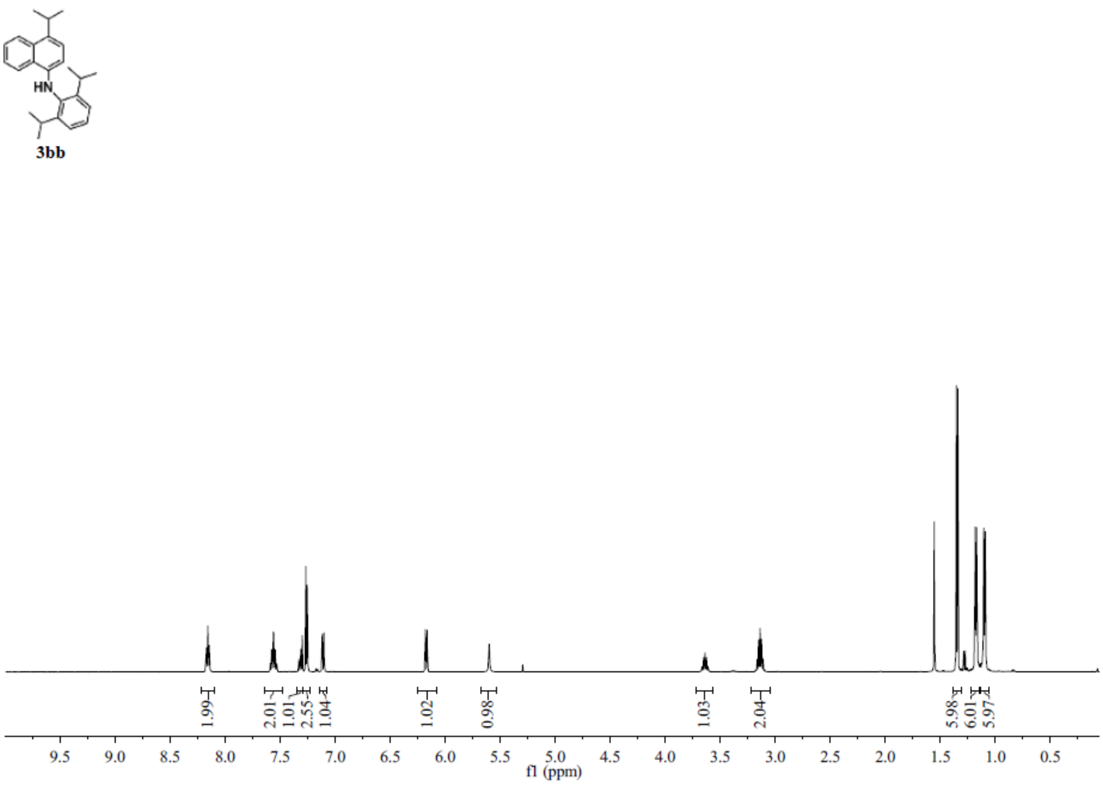

Supplementary Figure 51. ^{1}H-NMR of compound **3bb**, recorded at 500 MHz and 25 °C in $CDCl_3$.

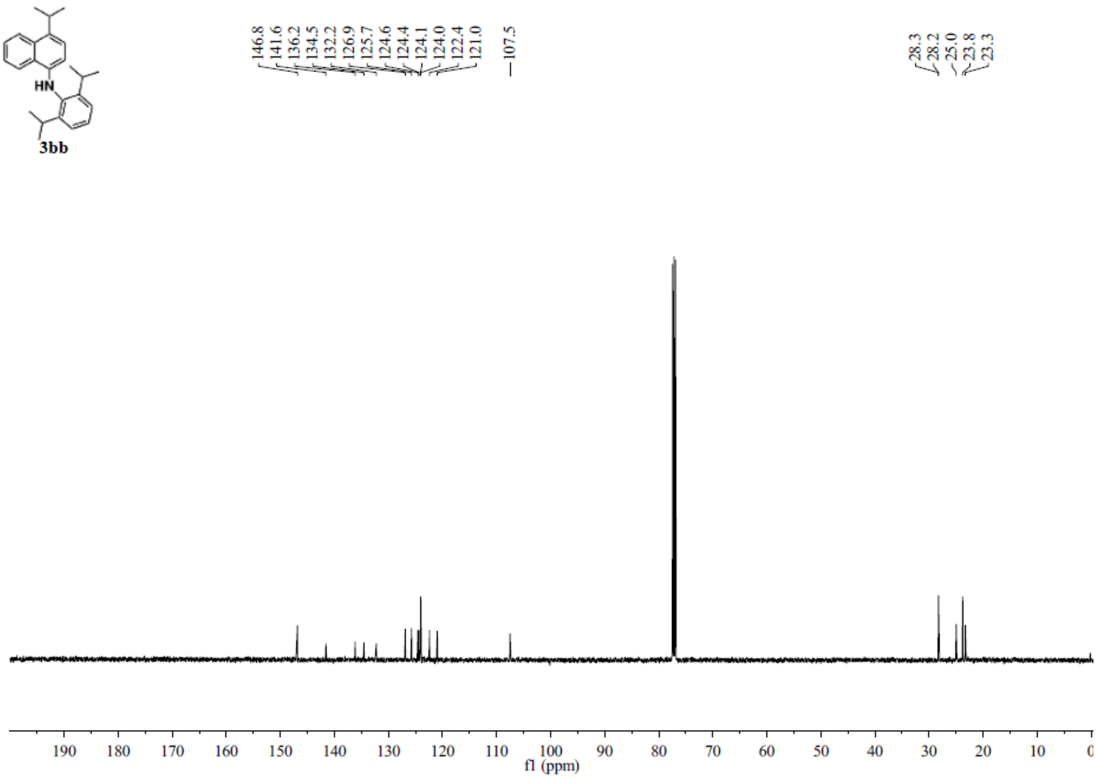

Supplementary Figure 52. ^{13}C-NMR of compound **3bb**, recorded at 125 MHz and 25 °C in $CDCl_3$.

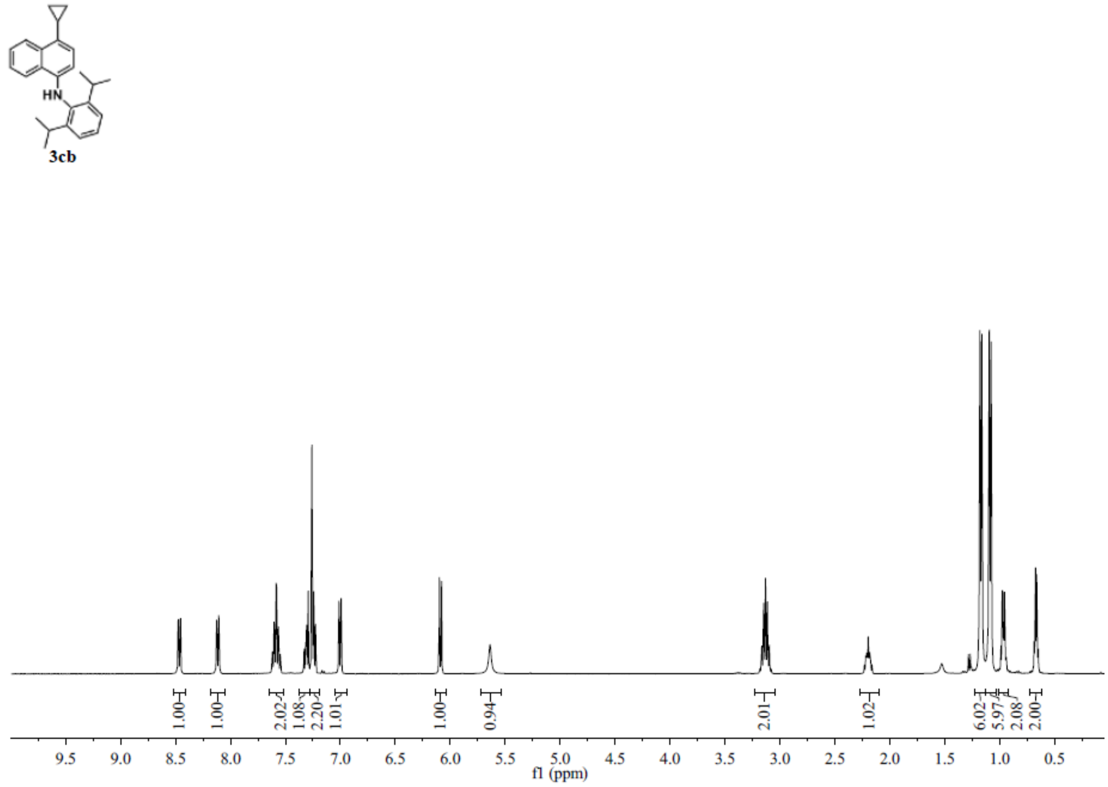

Supplementary Figure 53. ^{1}H-NMR of compound **3cb**, recorded at 400 MHz and 25 °C in $CDCl_3$.

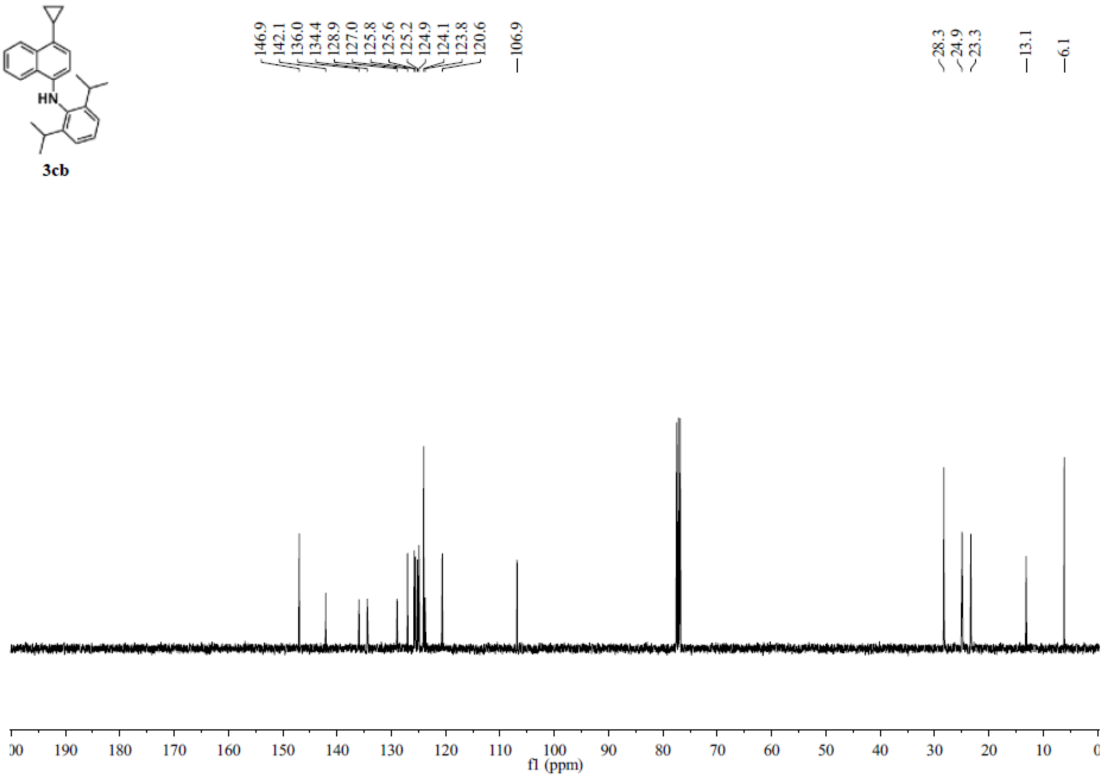

Supplementary Figure 54. ^{13}C-NMR of compound **3cb**, recorded at 100 MHz and 25 °C in $CDCl_3$.

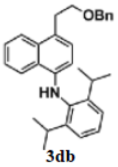

3db

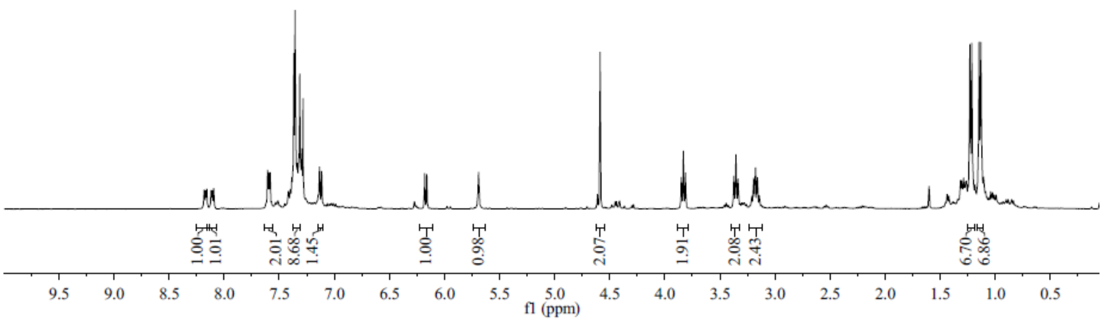

Supplementary Figure 55. ^{1}H-NMR of compound **3db**, recorded at 400 MHz and 25 °C in $CDCl_3$.

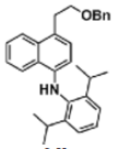

3db

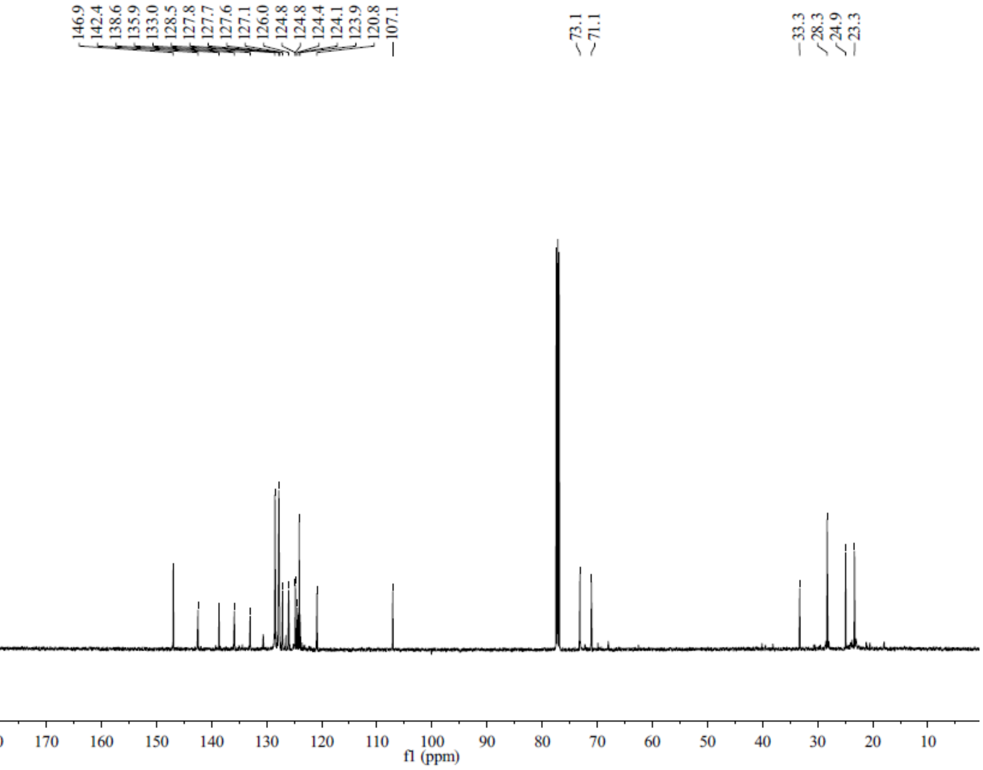

Supplementary Figure 56. ^{13}C-NMR of compound **3db**, recorded at 125 MHz and 25 °C in $CDCl_3$.

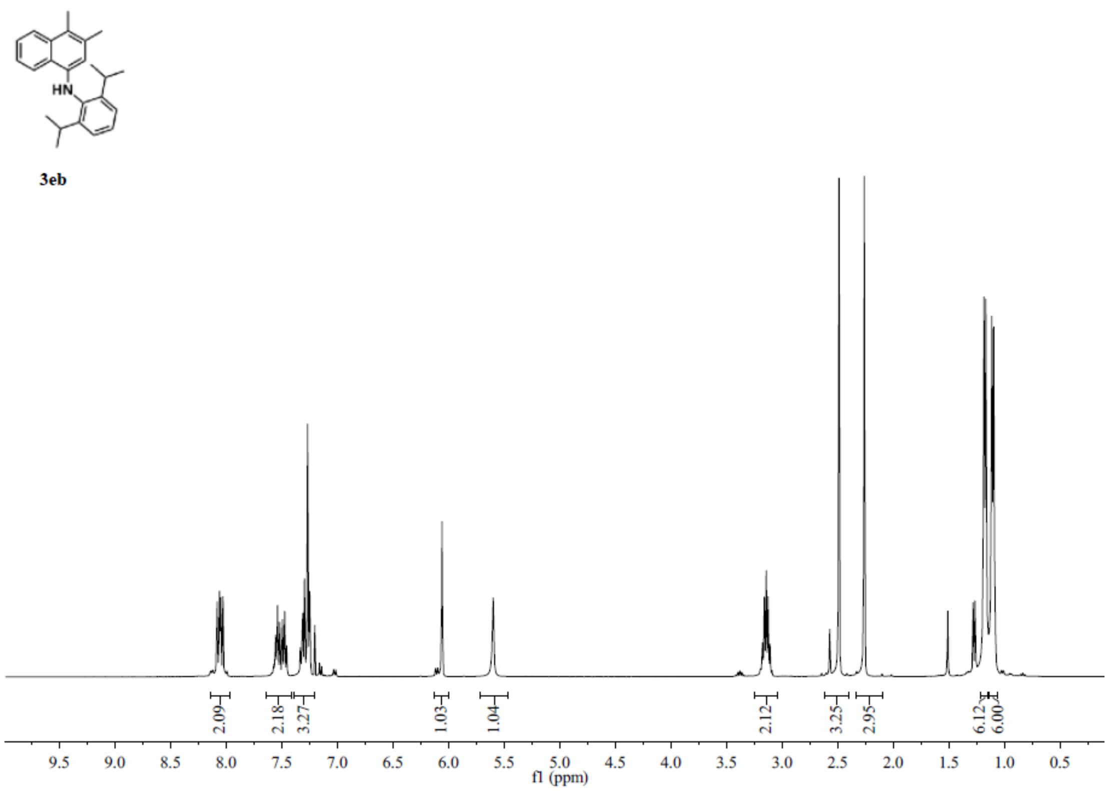

Supplementary Figure 57. ^{1}H-NMR of compound **3eb**, recorded at 400 MHz and 25 °C in $CDCl_3$.

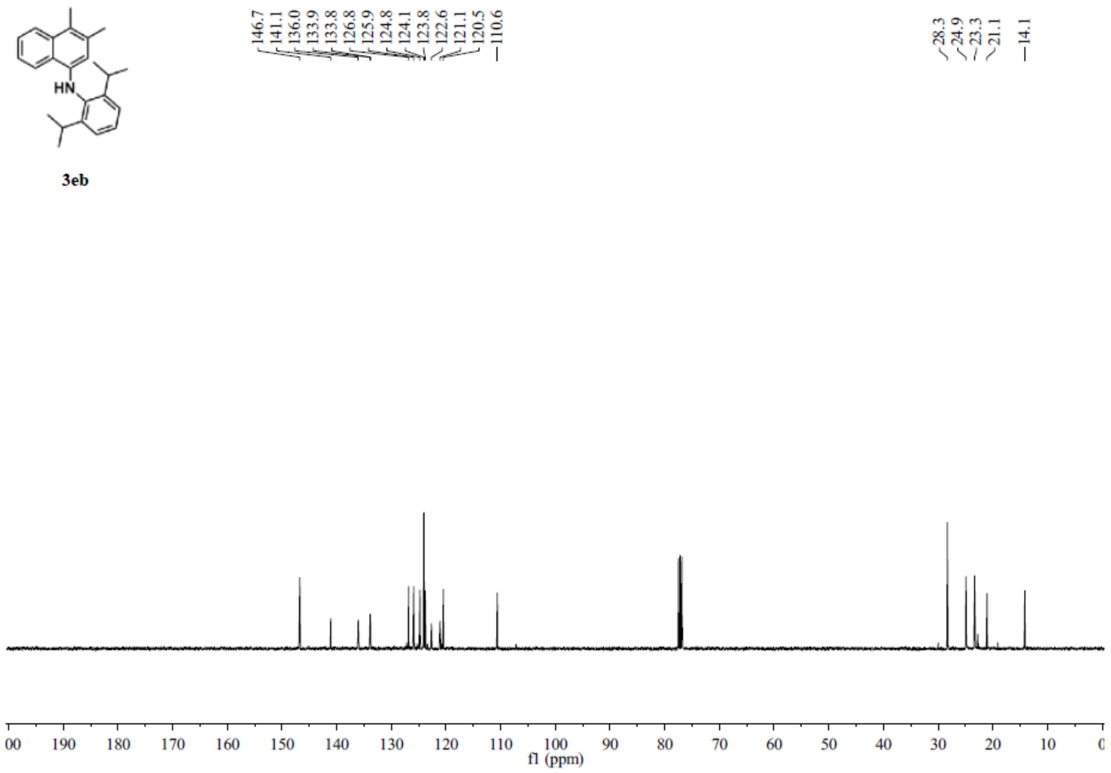

Supplementary Figure 58. ^{13}C-NMR of compound **3eb**, recorded at 100 MHz and 25 °C in $CDCl_3$.

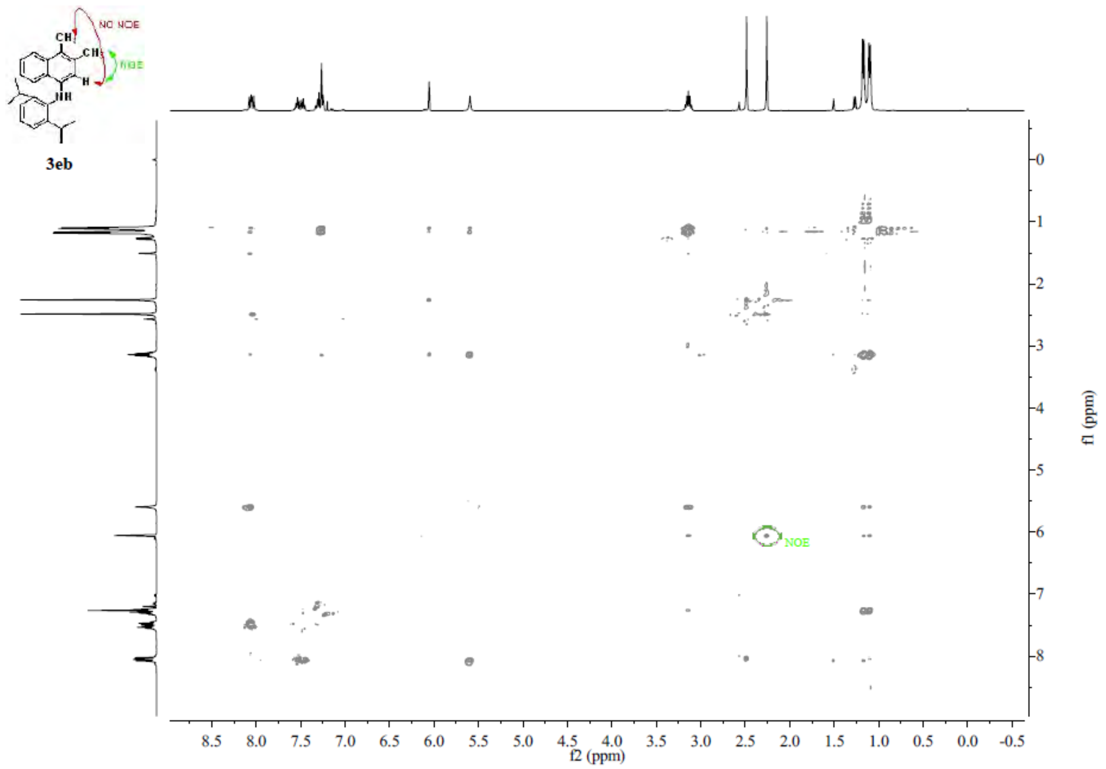

Supplementary Figure 59. NOESY of compound **3eb**, recorded at 400 MHz and 25 °C in $CDCl_3$.

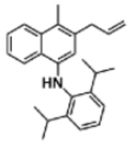

3fb

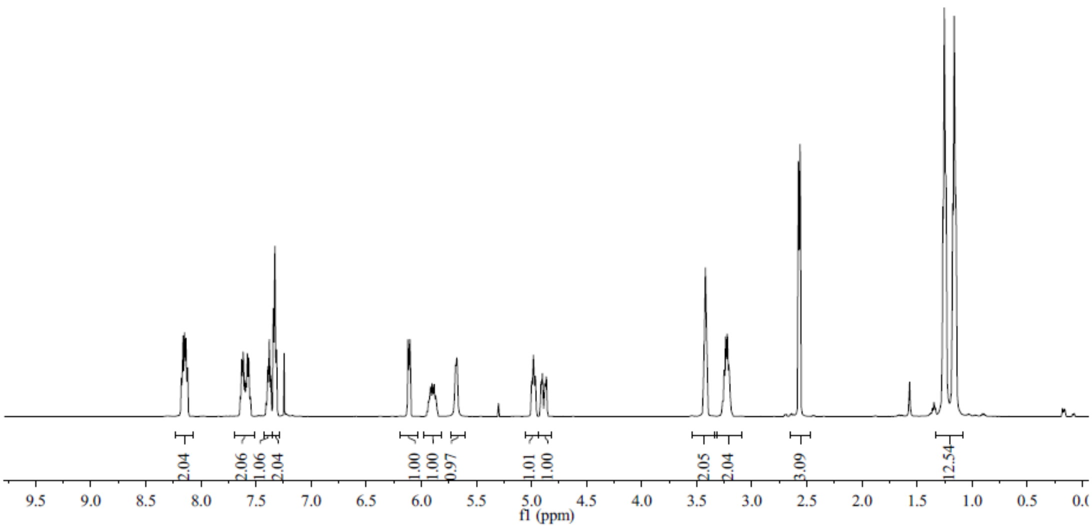

Supplementary Figure 60. ^{1}H-NMR of compound **3fb**, recorded at 500 MHz and 25 °C in $CDCl_3$.

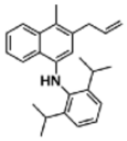

3fb

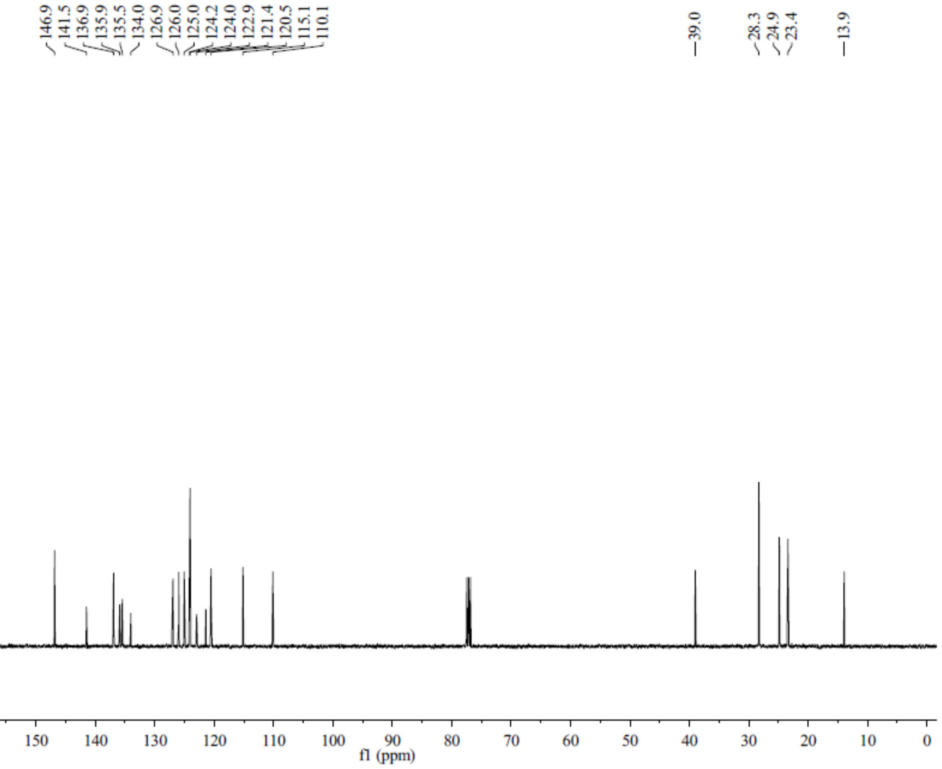

Supplementary Figure 61. ^{13}C-NMR of compound **3fb**, recorded at 125 MHz and 25 °C in $CDCl_3$.

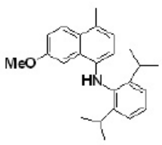

3gb

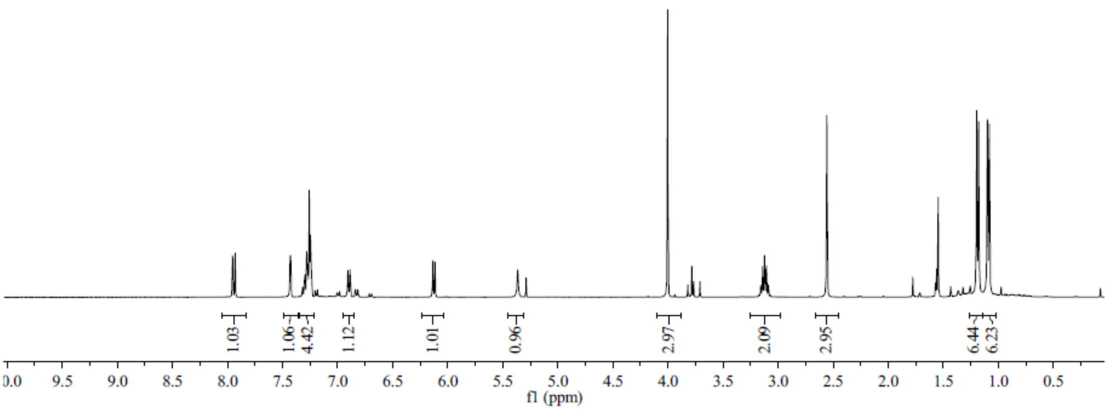

Supplementary Figure 62. ^{1}H-NMR of compound **3gb**, recorded at 400 MHz and 25 °C in $CDCl_3$.

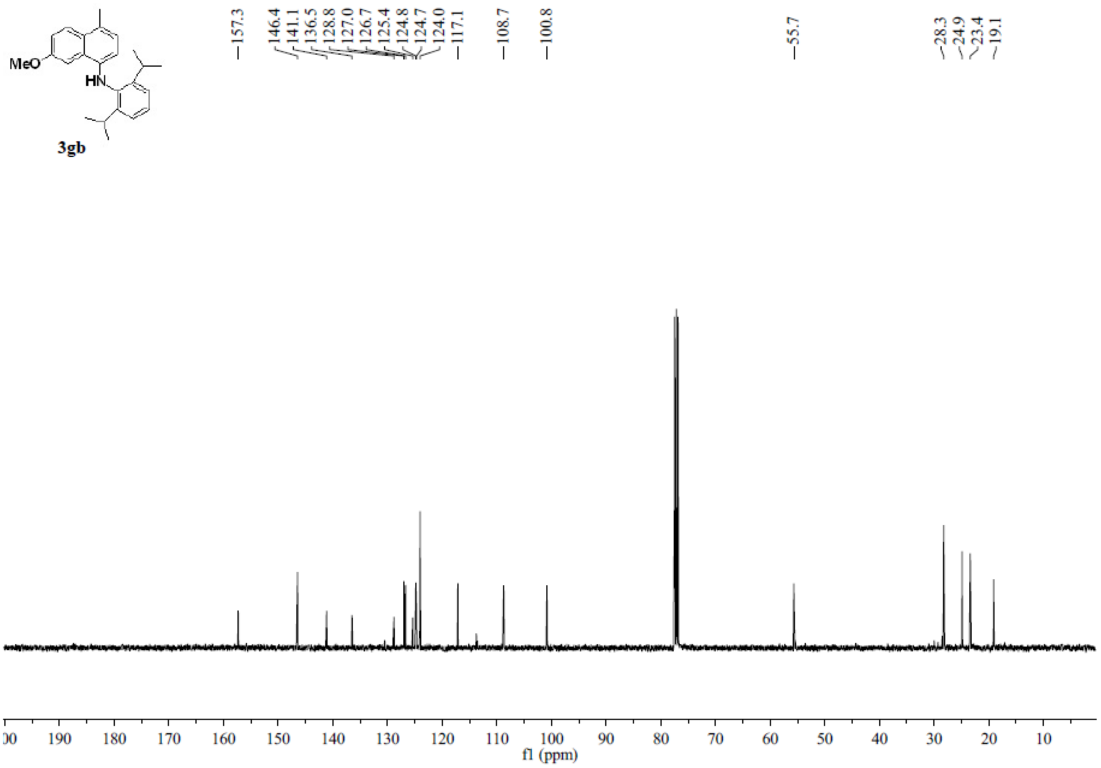

Supplementary Figure 63. ^{13}C-NMR of compound **3gb**, recorded at 100 MHz and 25 °C in $CDCl_3$.

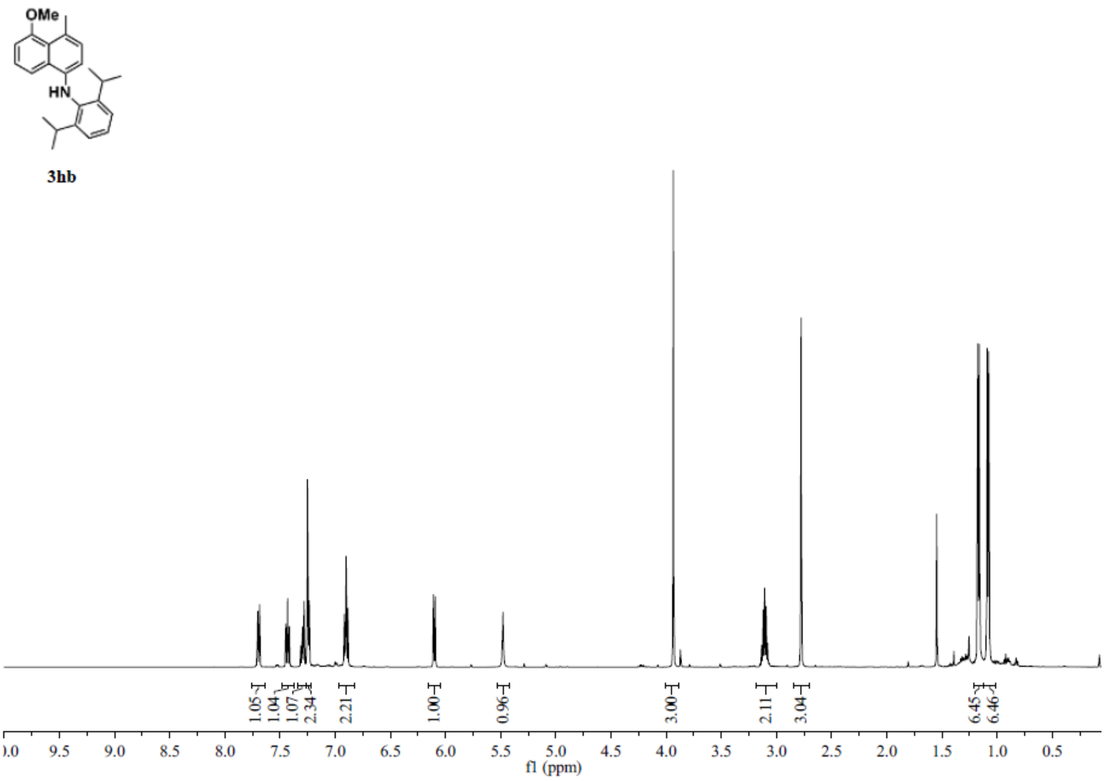

Supplementary Figure 64. ^{1}H-NMR of compound **3hb**, recorded at 500 MHz and 25 °C in $CDCl_3$.

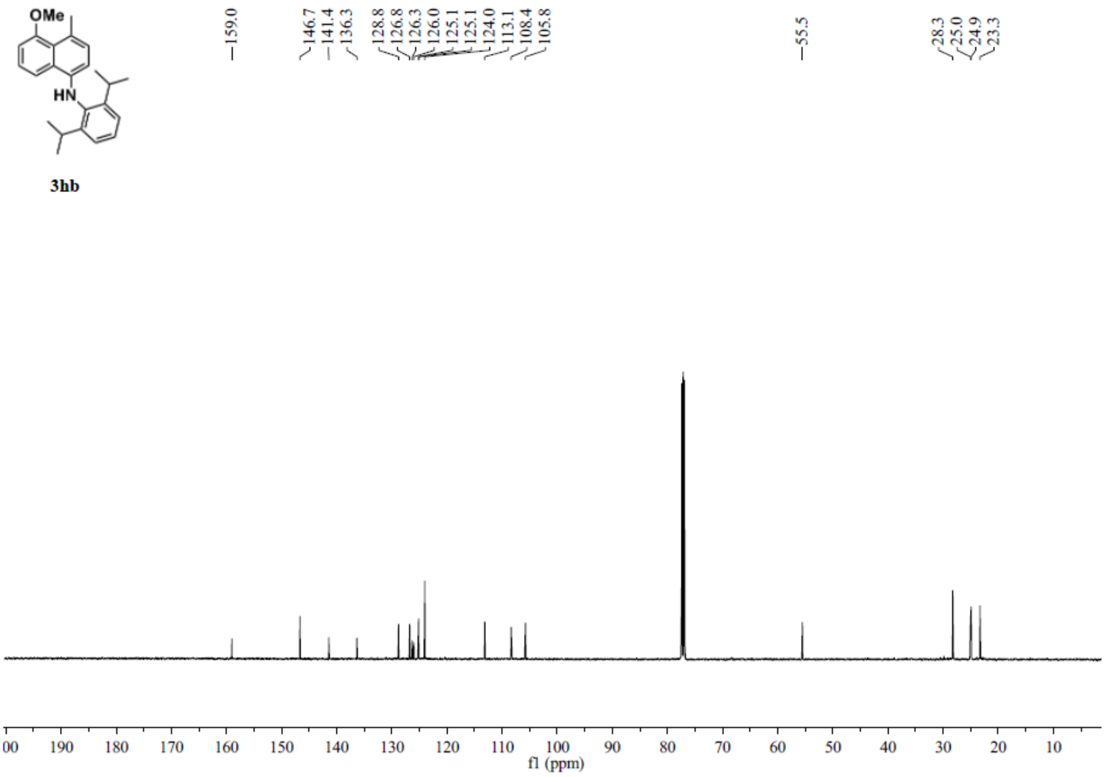

Supplementary Figure 65. ^{13}C-NMR of compound **3hb**, recorded at 125 MHz and 25 °C in $CDCl_3$.

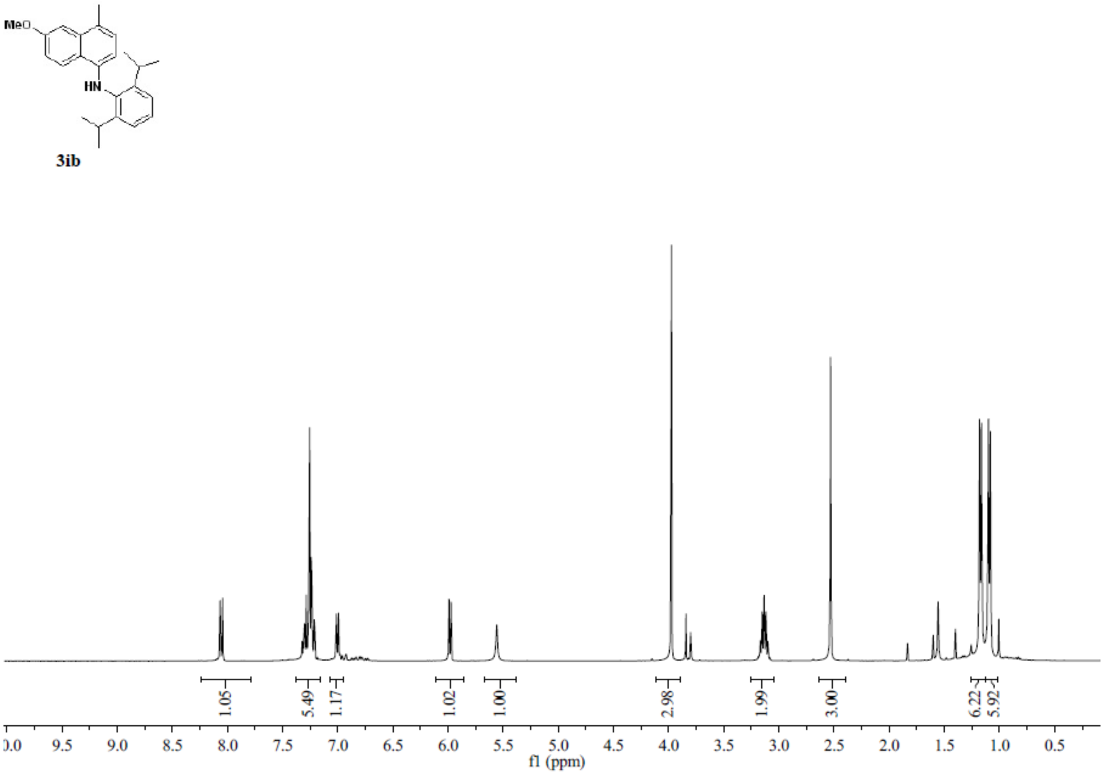

Supplementary Figure 66. ^{1}H-NMR of compound **3ib**, recorded at 400 MHz and 25 °C in $CDCl_3$.

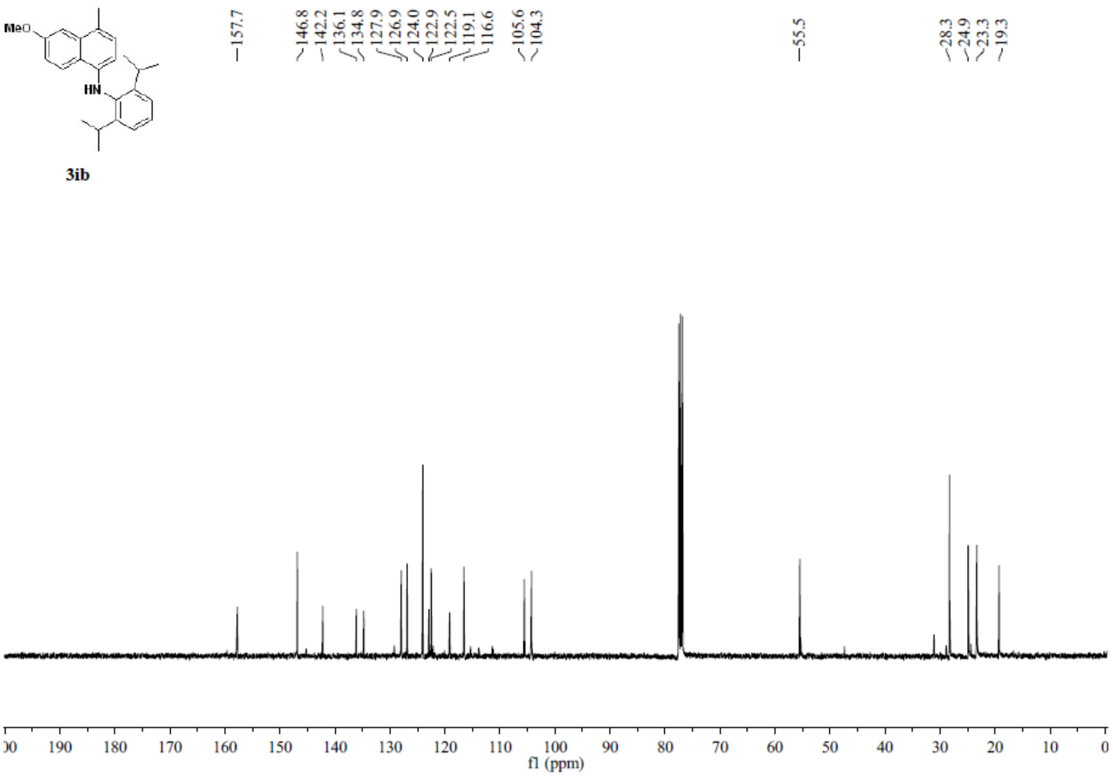

Supplementary Figure 67. ^{13}C-NMR of compound **3ib**, recorded at 100 MHz and 25 °C in $CDCl_3$.

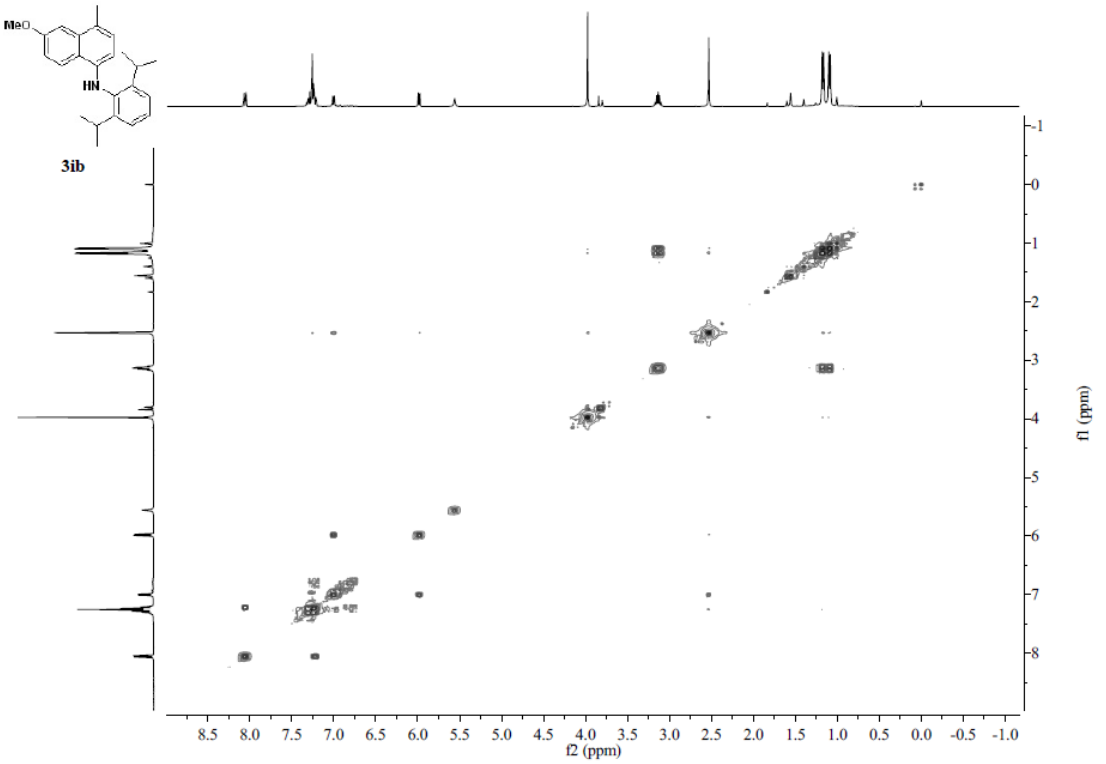

Supplementary Figure 68. COSY of compound **3ib**, recorded at 400 MHz and 25 °C in $CDCl_3$.

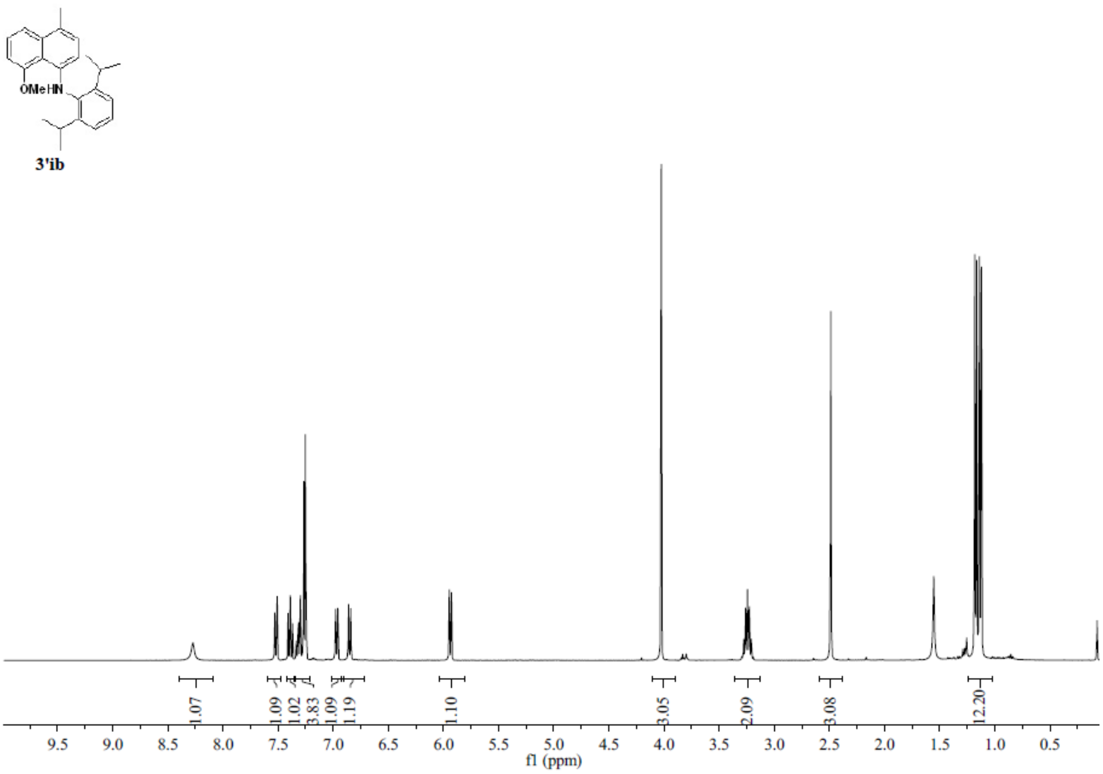

Supplementary Figure 69. ^{1}H-NMR of compound **3’ib**, recorded at 400 MHz and 25 °C in $CDCl_3$.

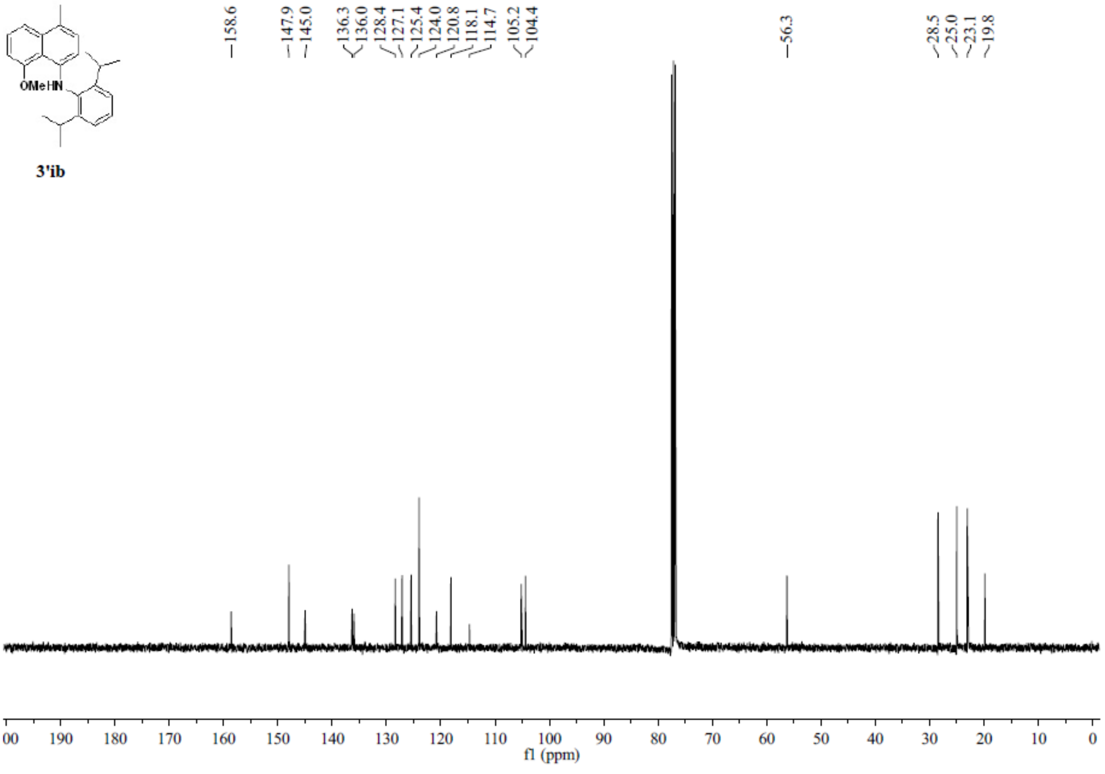

Supplementary Figure 70. ^{13}C-NMR of compound **3’ib**, recorded at 100 MHz and 25 °C in $CDCl_3$.

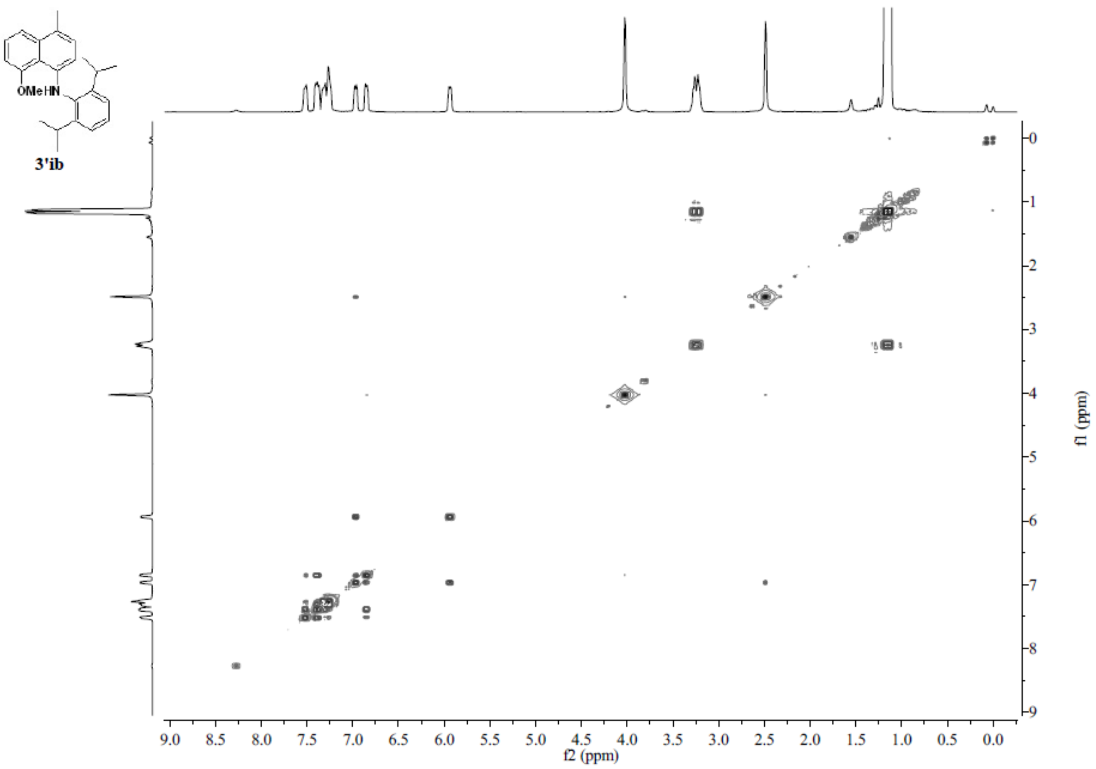

Supplementary Figure 71. COSY of compound **3‘ib**, recorded at 400 MHz and 25 °C in $CDCl_3$

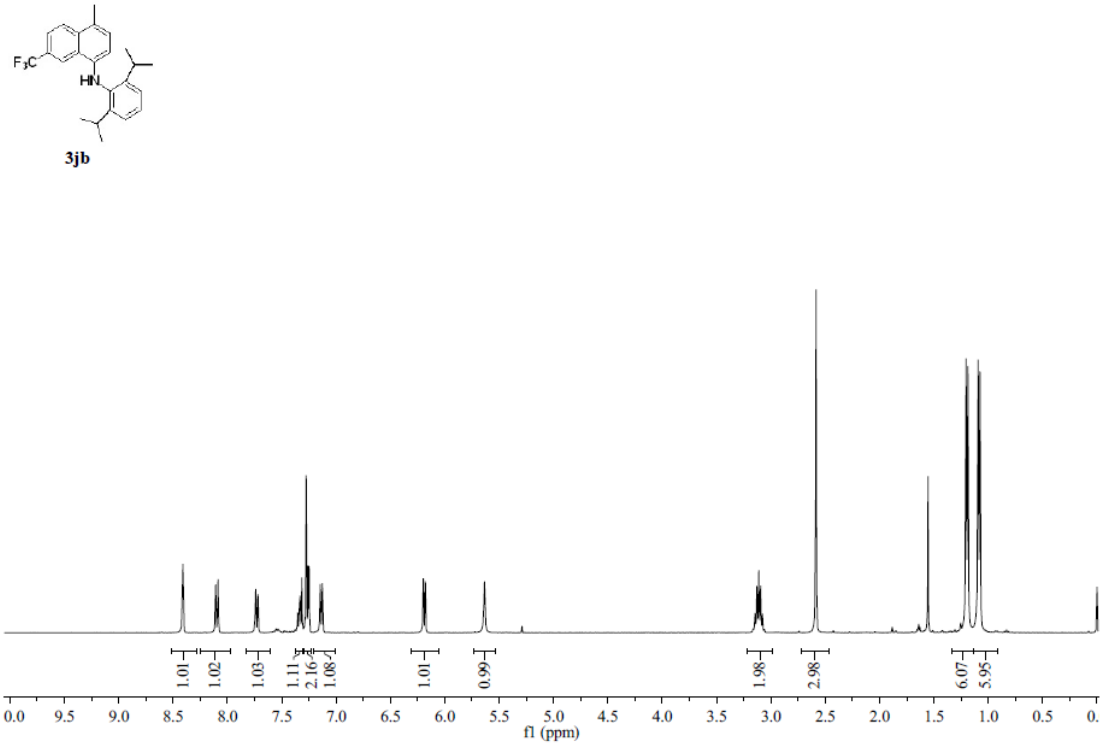

Supplementary Figure 72. ^{1}H-NMR of compound **3jb**, recorded at 400 MHz and 25 °C in $CDCl_3$.

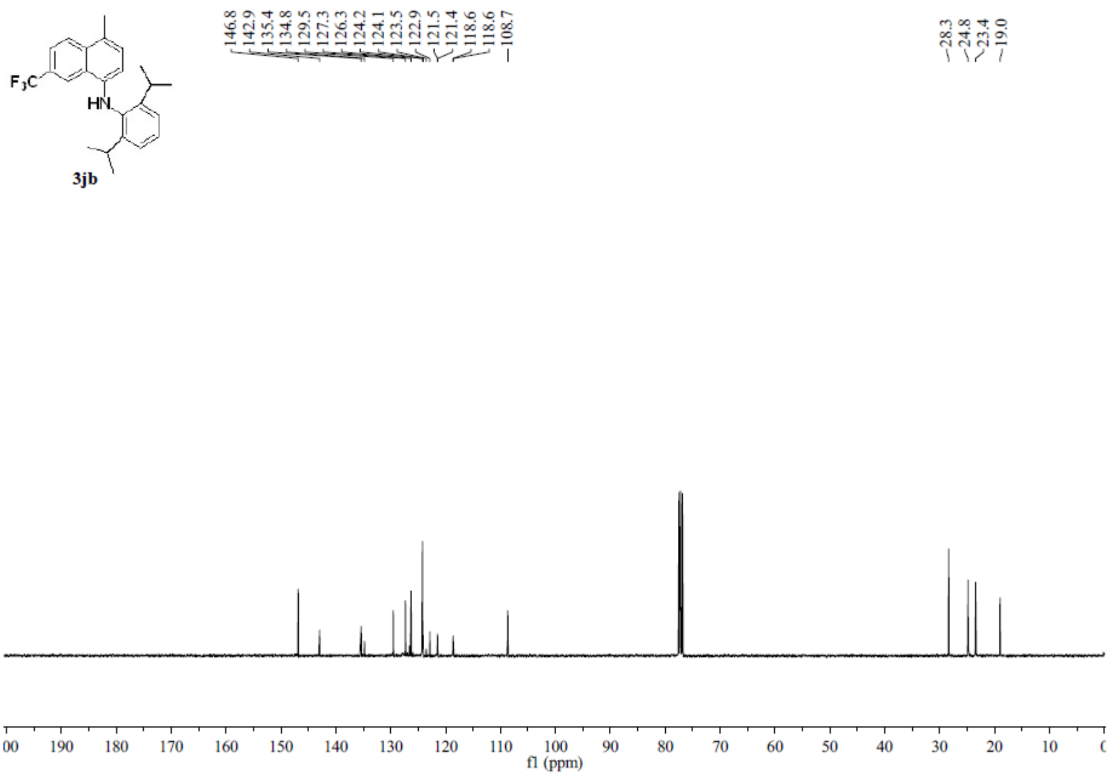

Supplementary Figure 73. ^{13}C-NMR of compound **3jb**, recorded at 100 MHz and 25 °C in $CDCl_3$.

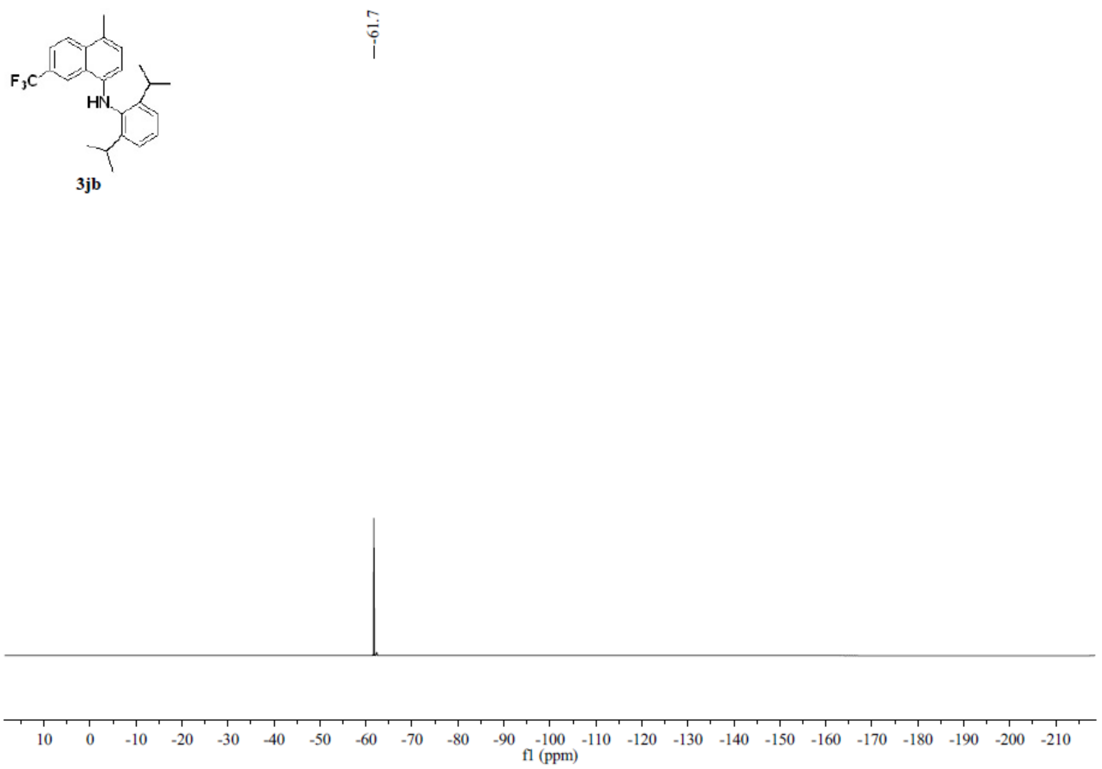

Supplementary Figure 74. ^{19}F-NMR of compound **3jb**, recorded at 376 MHz and 25 °C in $CDCl_3$.

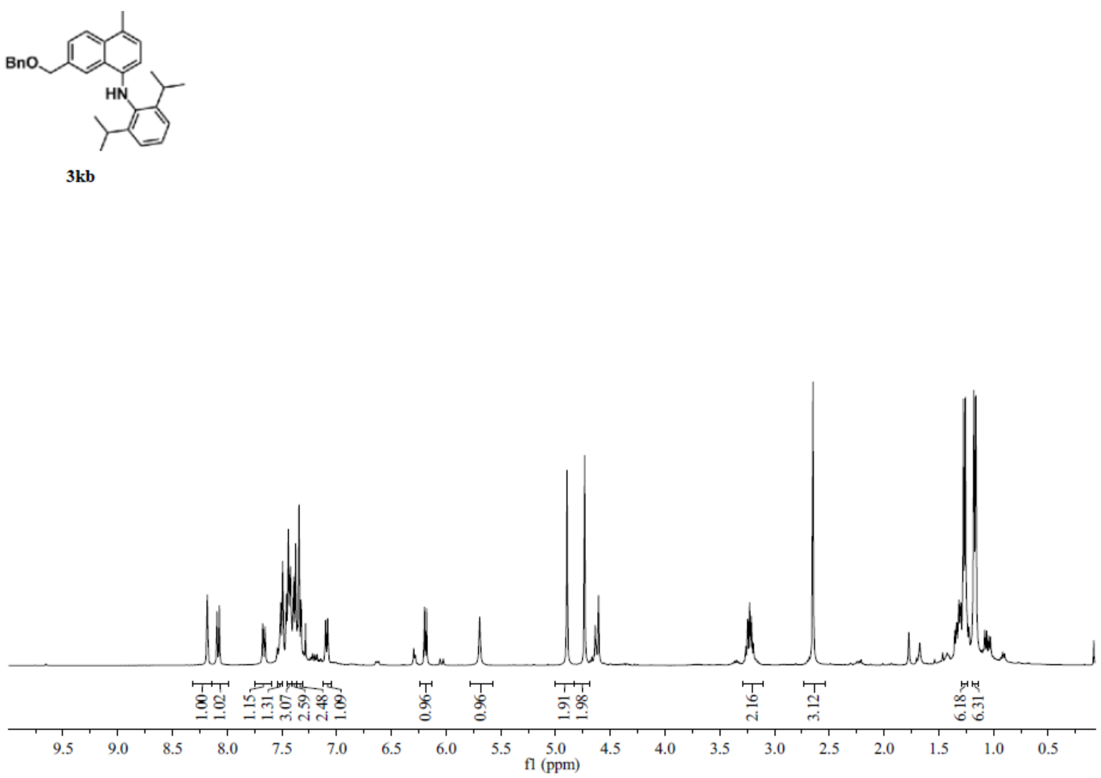

Supplementary Figure 75. ^{1}H-NMR of compound **3kb**, recorded at 400 MHz and 25 °C in $CDCl_3$.

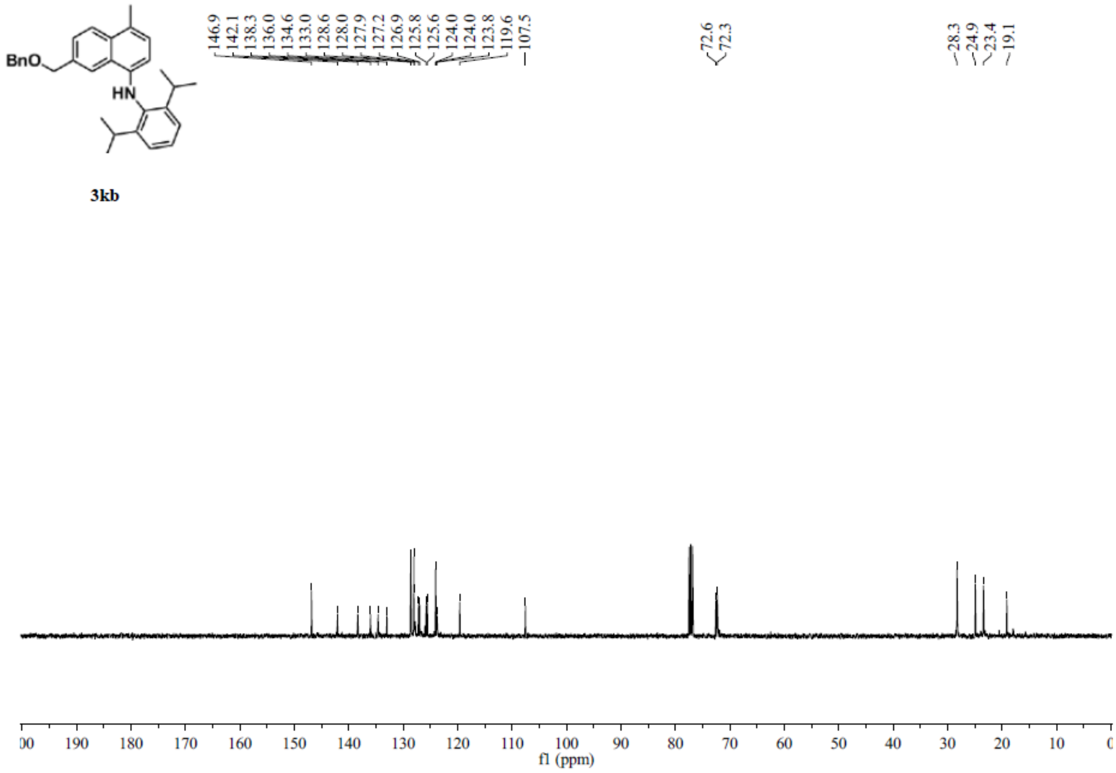

Supplementary Figure 76. ^{13}C-NMR of compound **3kb**, recorded at 100 MHz and 25 °C in $CDCl_3$.

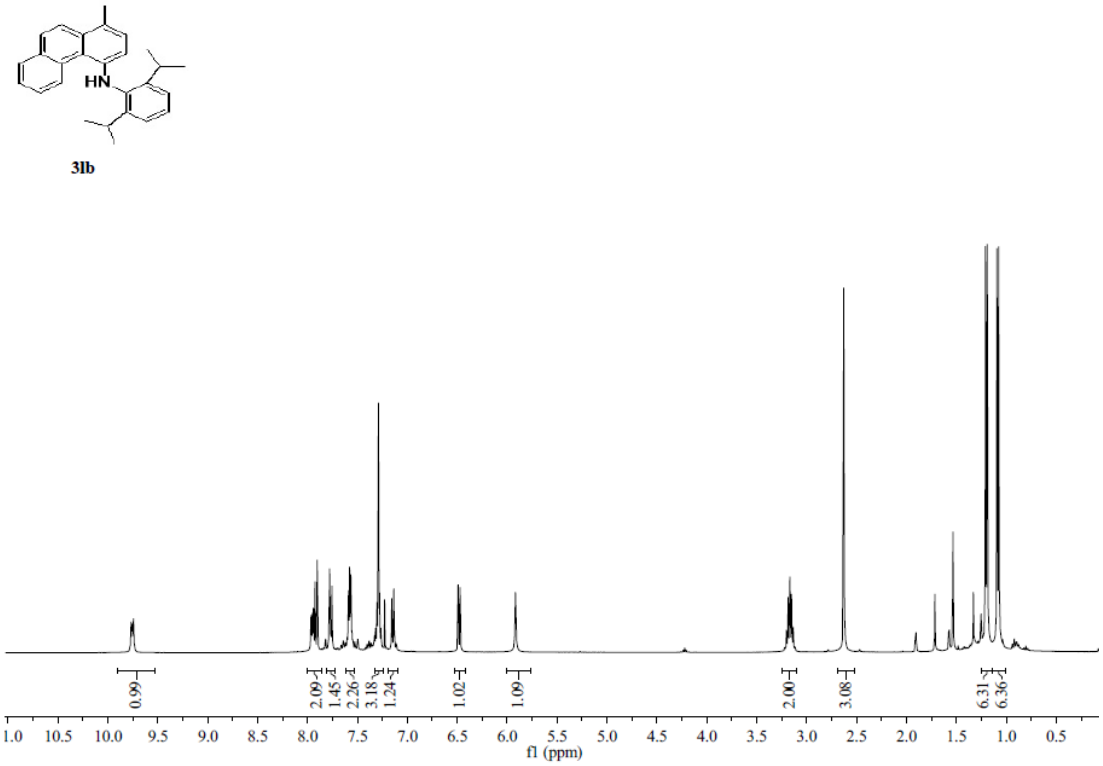

Supplementary Figure 77. ^{1}H-NMR of compound **3lb**, recorded at 400 MHz and 25 °C in $CDCl_3$.

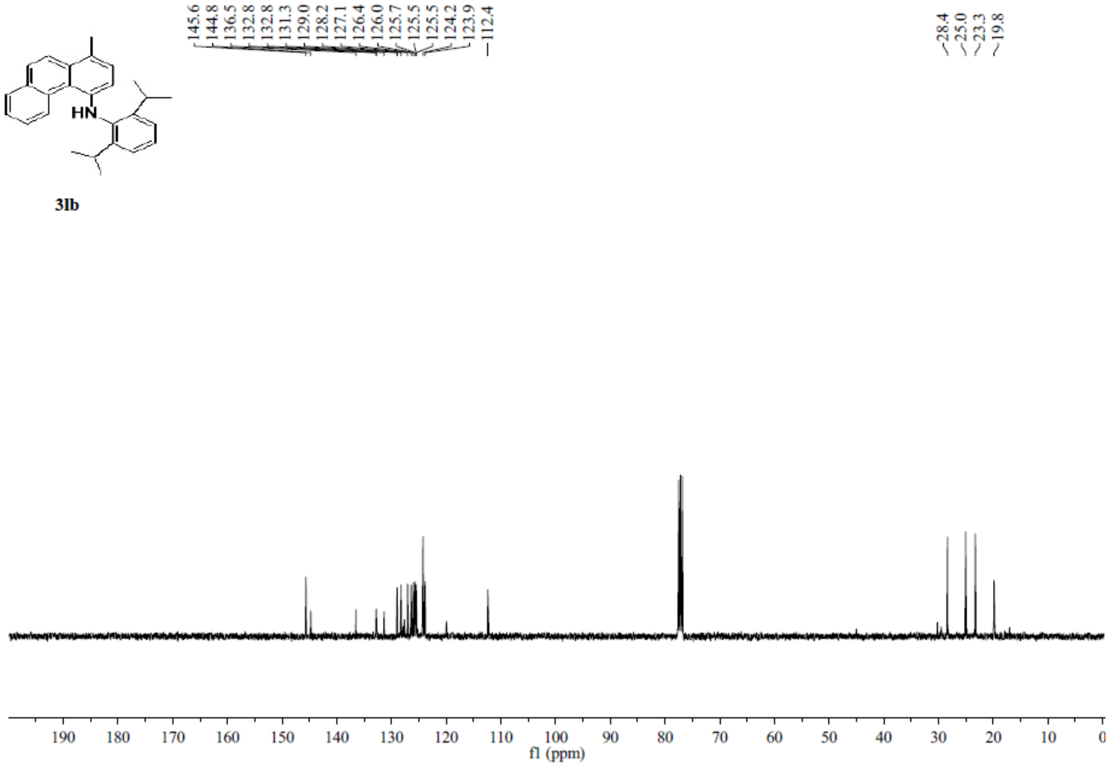

Supplementary Figure 78. ^{13}C-NMR of compound **3lb**, recorded at 100 MHz and 25 °C in $CDCl_3$.

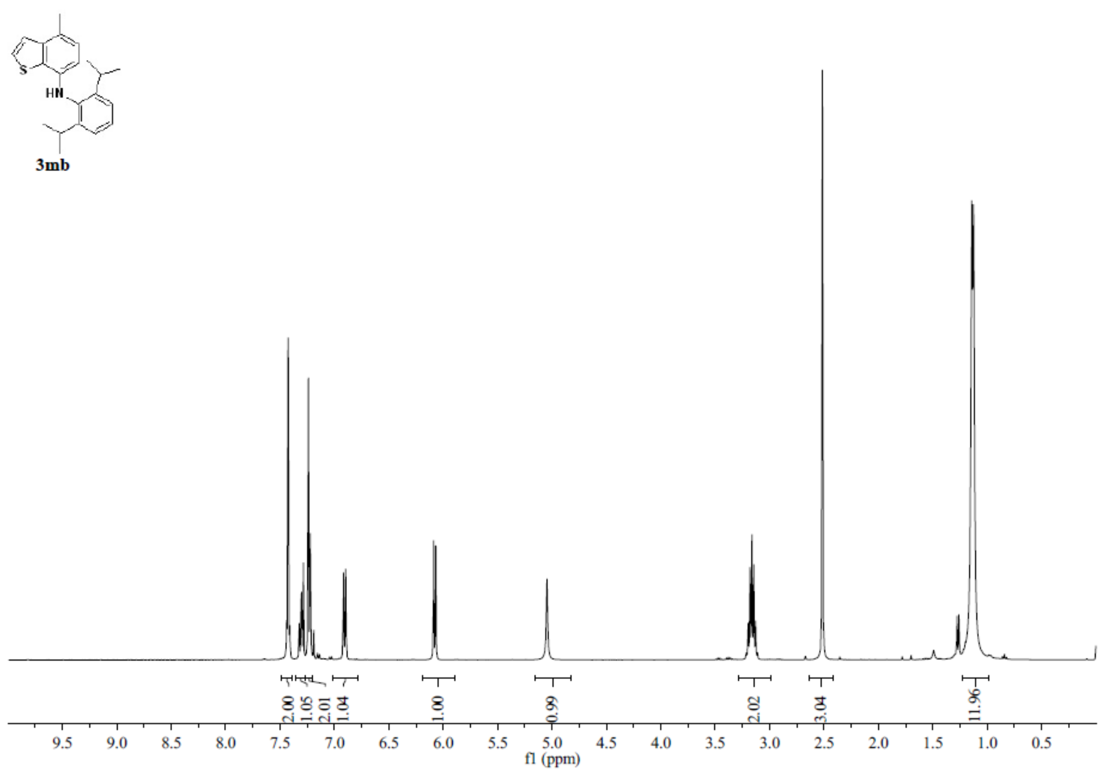

Supplementary Figure 79. ^{1}H-NMR of compound **3mb**, recorded at 400 MHz and 25 °C in $CDCl_3$.

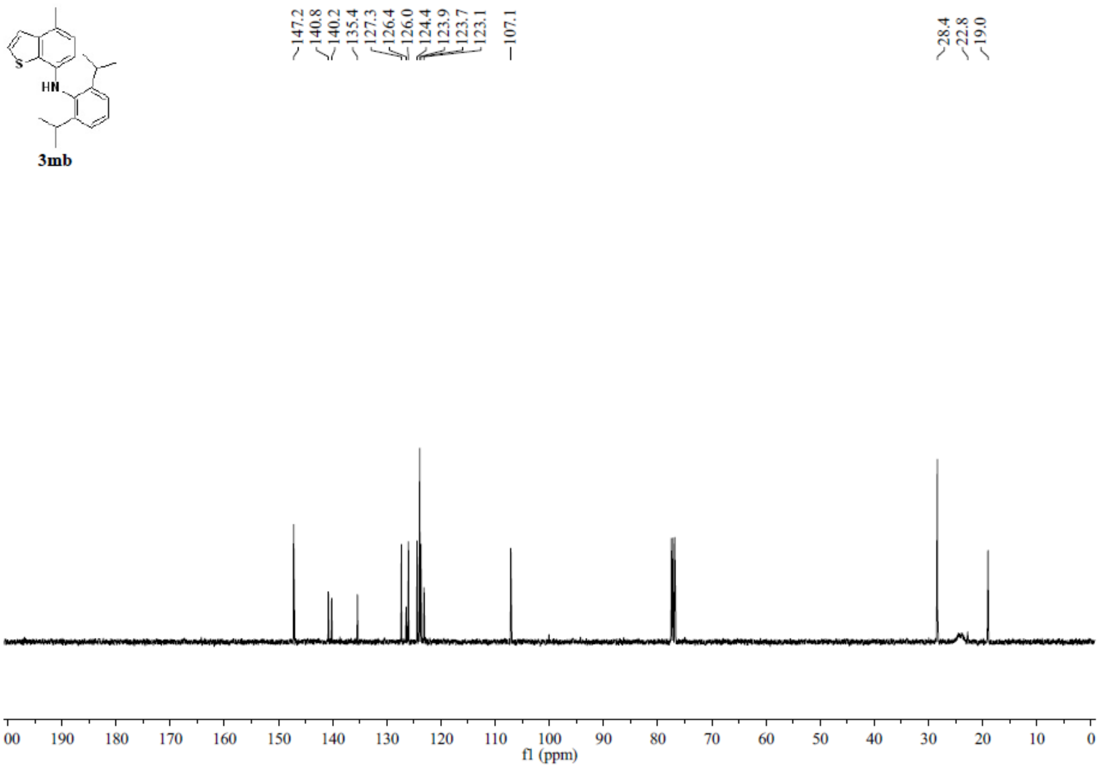

Supplementary Figure 80. ^{13}C-NMR of compound **3mb**, recorded at 100MHz and 25 °C in $CDCl_3$.

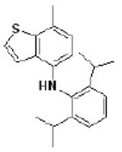

3nb

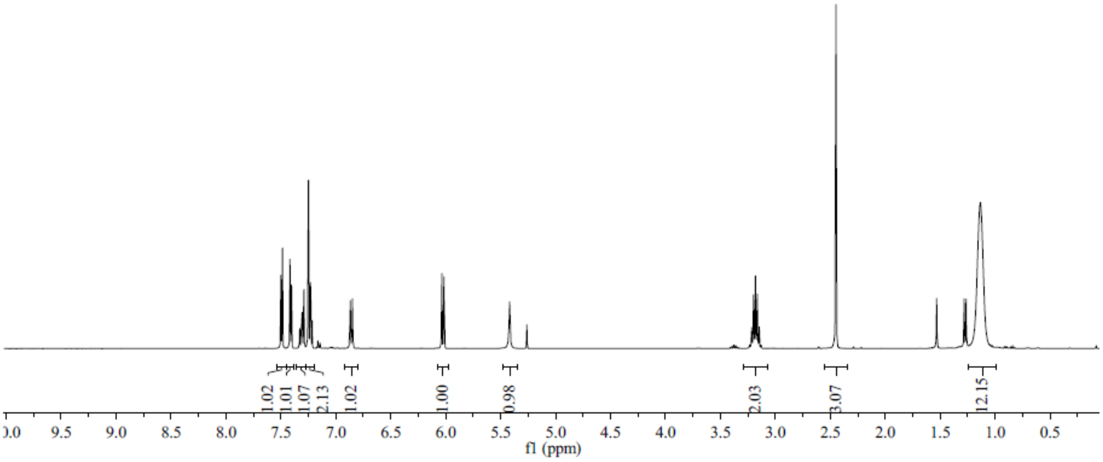

Supplementary Figure 81. ^{1}H-NMR of compound **3nb**, recorded at 400 MHz and 25 °C in $CDCl_3$.

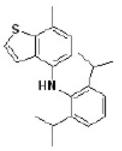

3nb

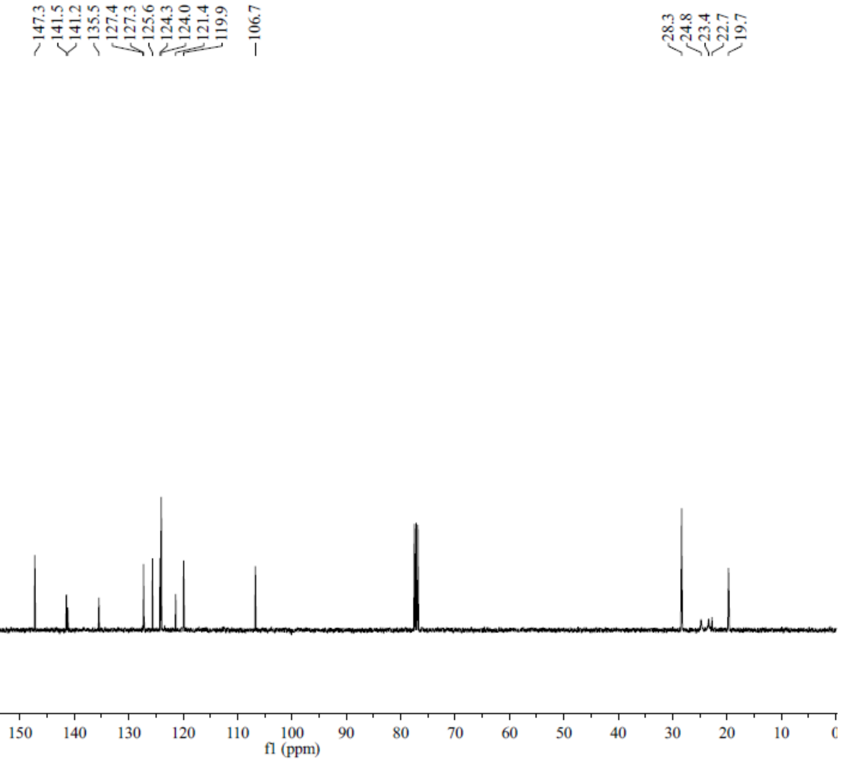

Supplementary Figure 82. ^{13}C-NMR of compound **3nb**, recorded at 100 MHz and 25 °C in $CDCl_3$.

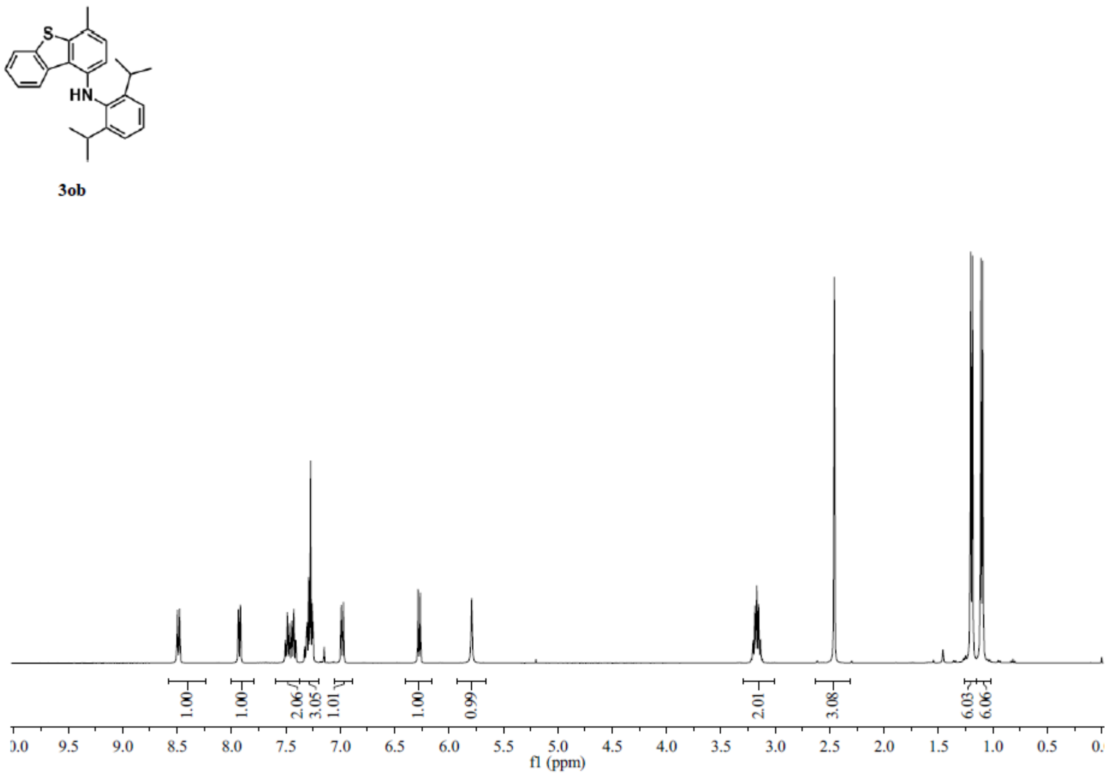

Supplementary Figure 83. ^{1}H-NMR of compound **3ob**, recorded at 400 MHz and 25 °C in $CDCl_3$.

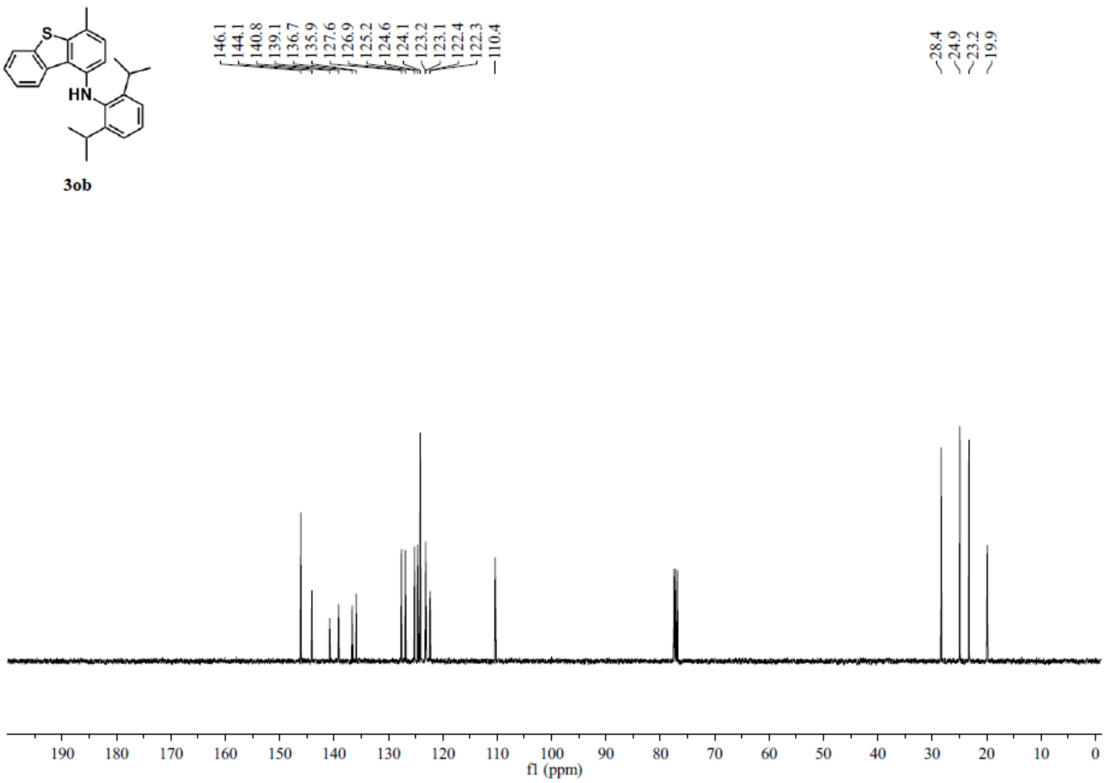

Supplementary Figure 84. ^{13}C-NMR of compound **3ob**, recorded at 100 MHz and 25 °C in $CDCl_3$.

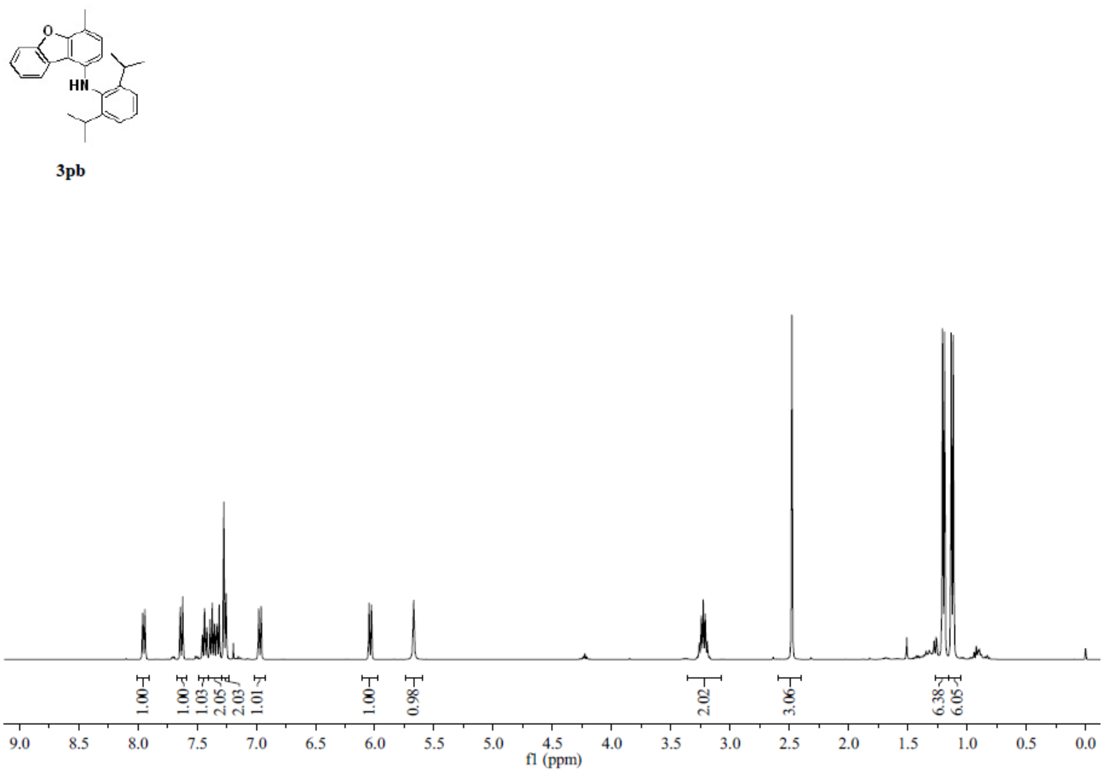

Supplementary Figure 85. ^{1}H-NMR of compound **3pb**, recorded at 400 MHz and 25 °C in $CDCl_3$.

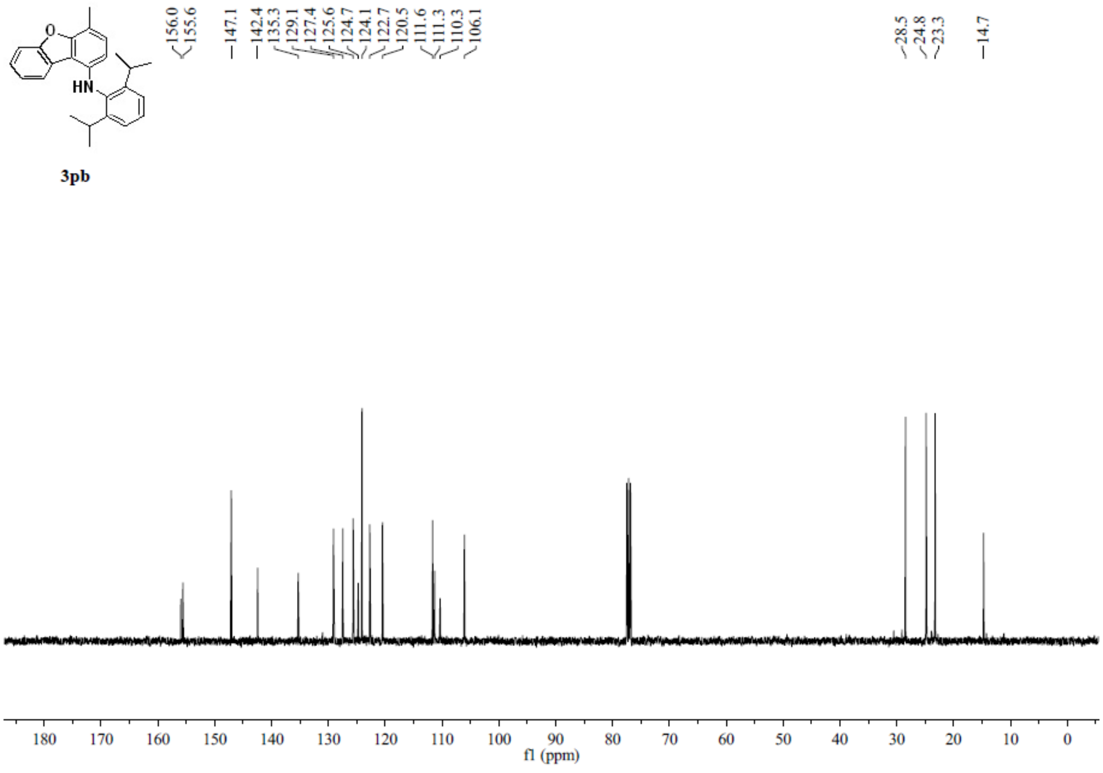

Supplementary Figure 86. ^{13}C-NMR of compound **3pb**, recorded at 100 MHz and 25 °C in $CDCl_3$.

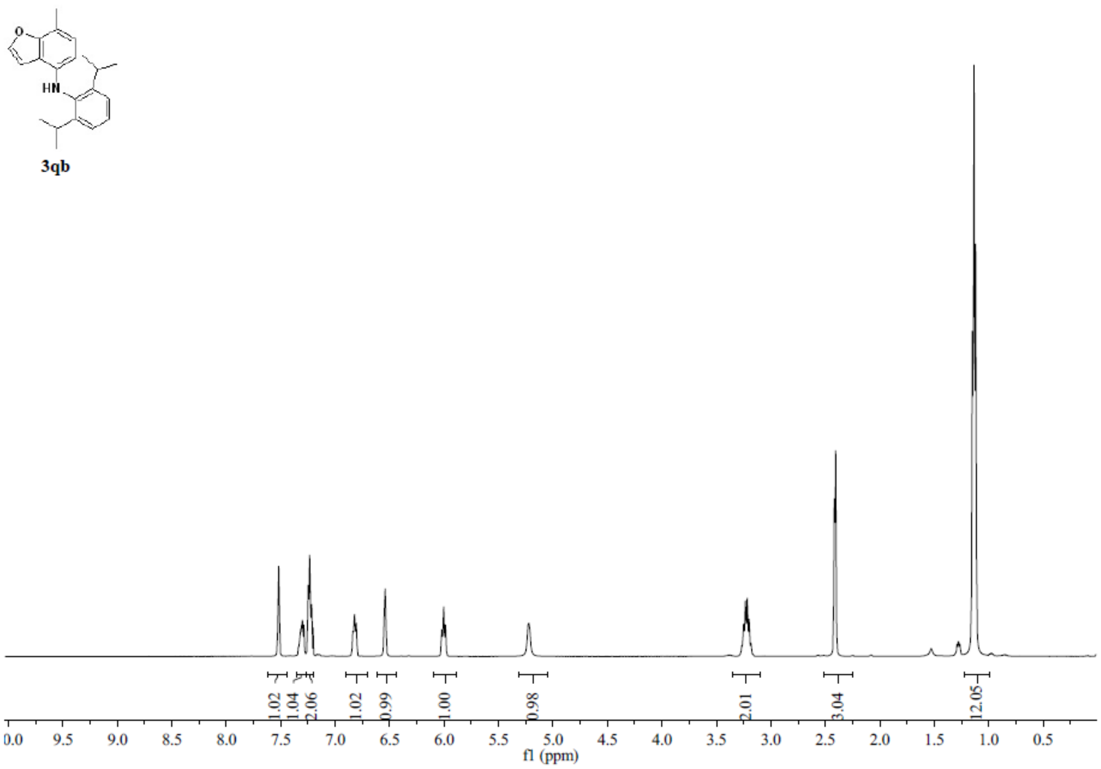

Supplementary Figure 87. ^{1}H-NMR of compound **3qb**, recorded at 400 MHz and 25 °C in $CDCl_3$.

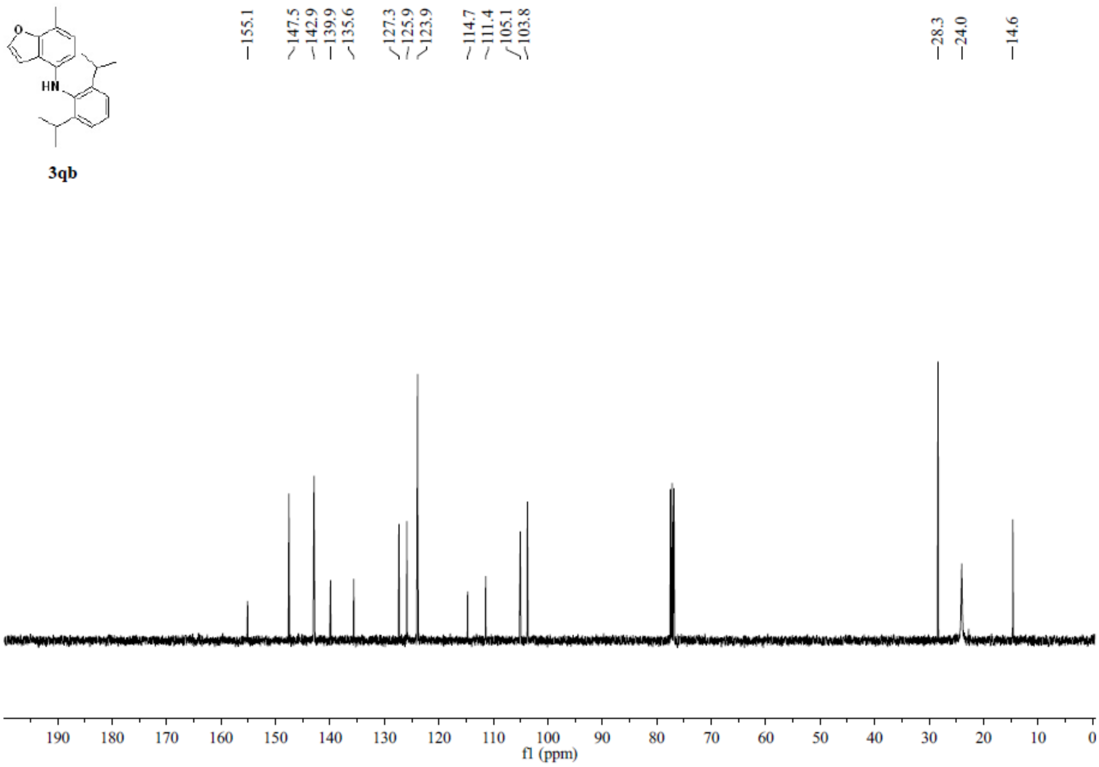

Supplementary Figure 88. ^{13}C-NMR of compound **3qb**, recorded at 100 MHz and 25 °C in $CDCl_3$.

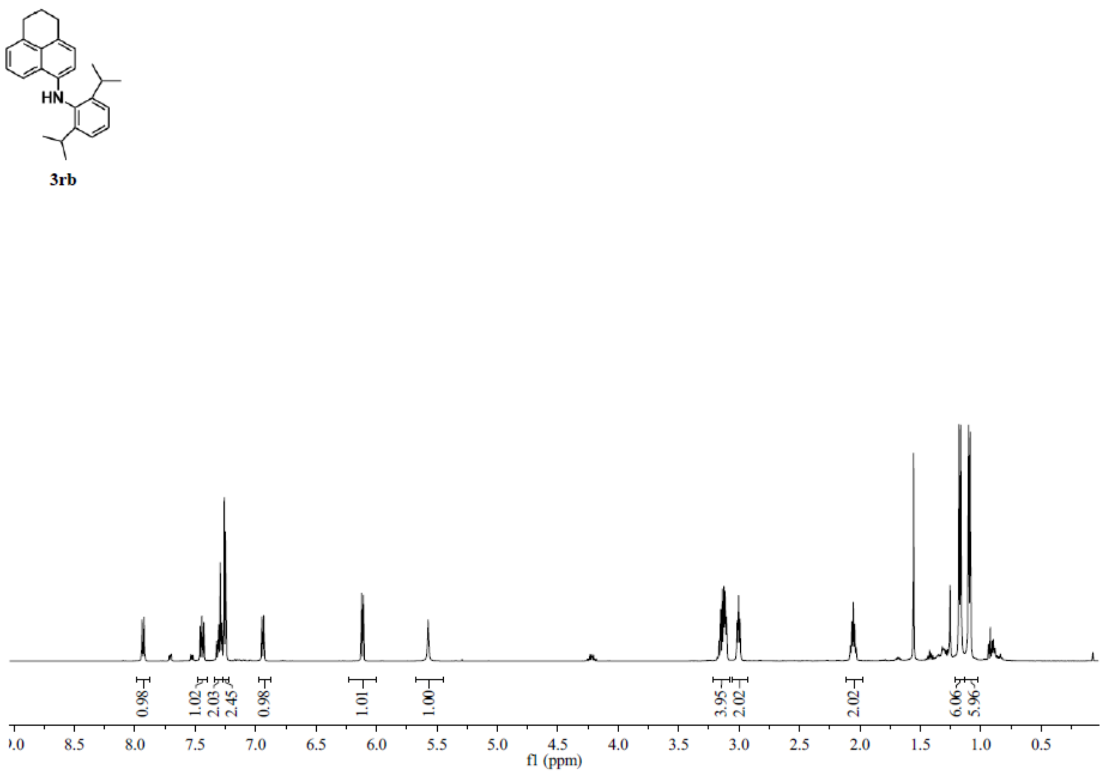

Supplementary Figure 89. ^{1}H-NMR of compound **3rb**, recorded at 500 MHz and 25 °C in $CDCl_3$.

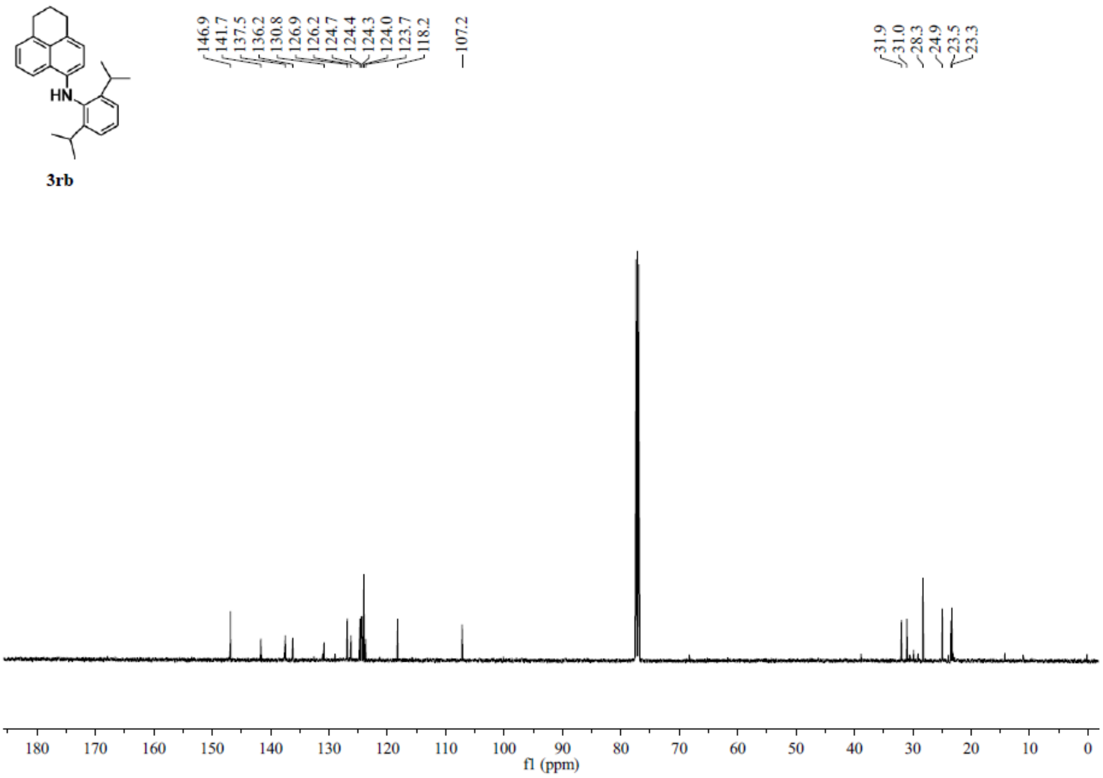

Supplementary Figure 90. ^{13}C-NMR of compound **3rb**, recorded at 125 MHz and 25 °C in $CDCl_3$.

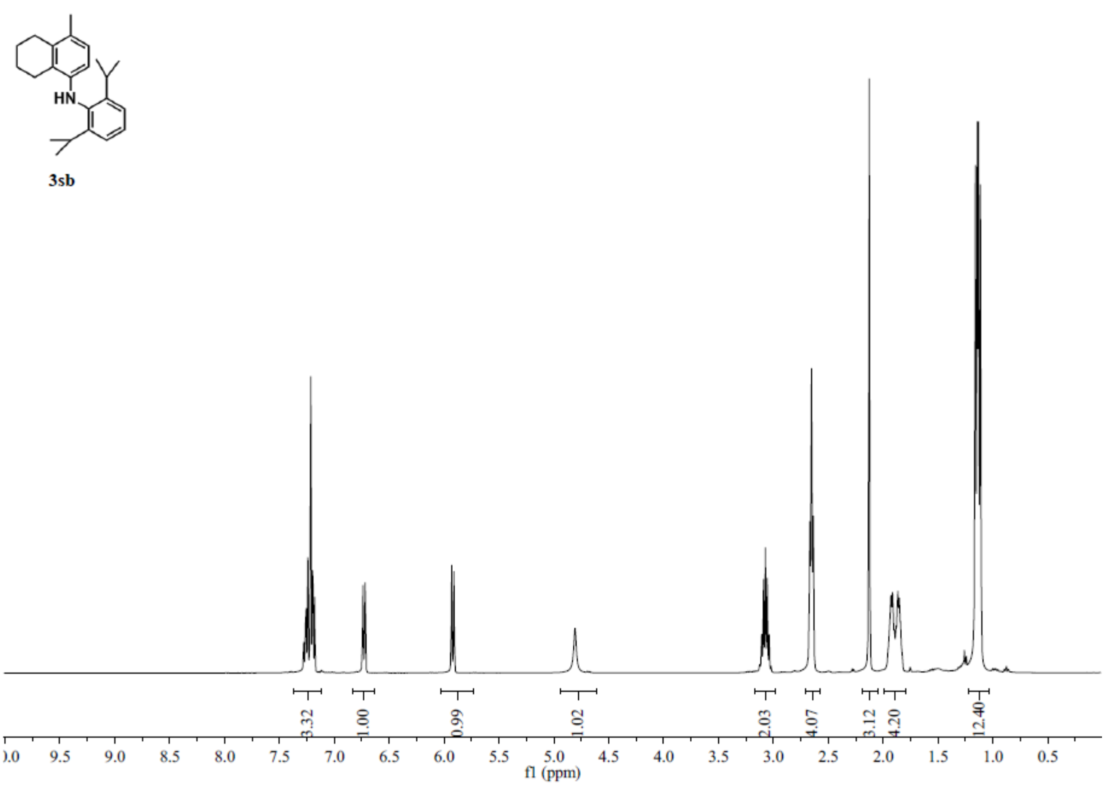

Supplementary Figure 91. ^{1}H-NMR of compound **3sb**, recorded at 400 MHz and 25 °C in $CDCl_3$.

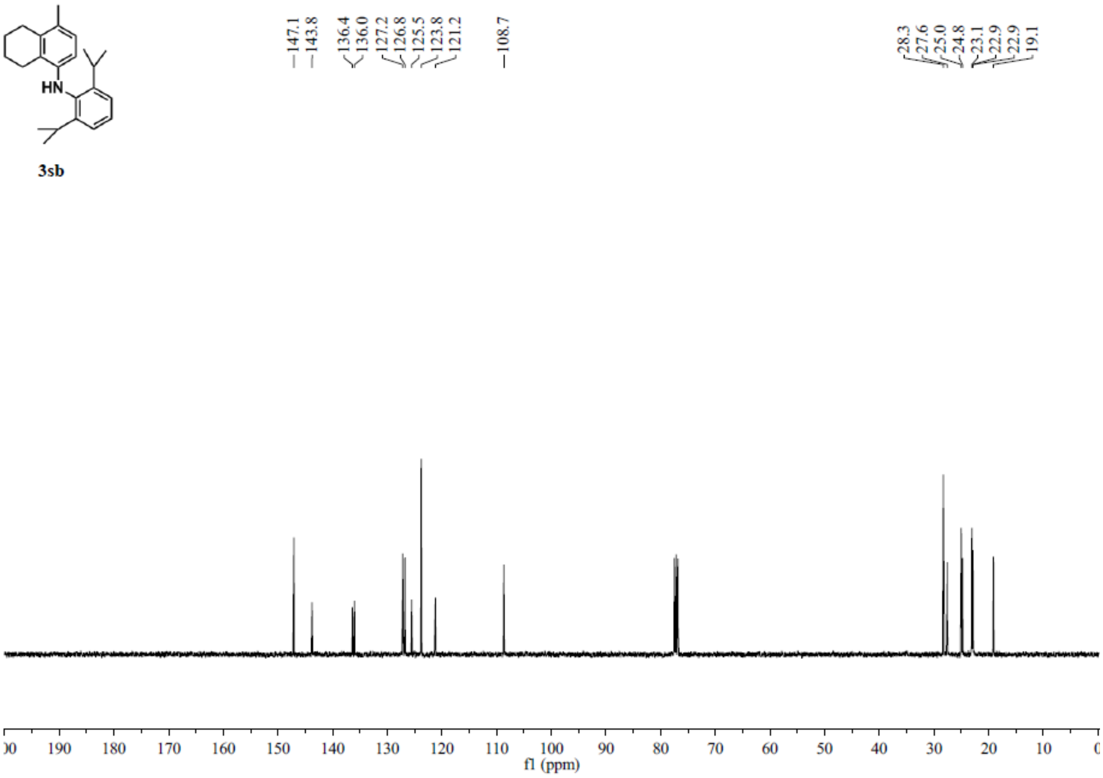

Supplementary Figure 92. ^{13}C-NMR of compound **3sb**, recorded at 100 MHz and 25 °C in $CDCl_3$.

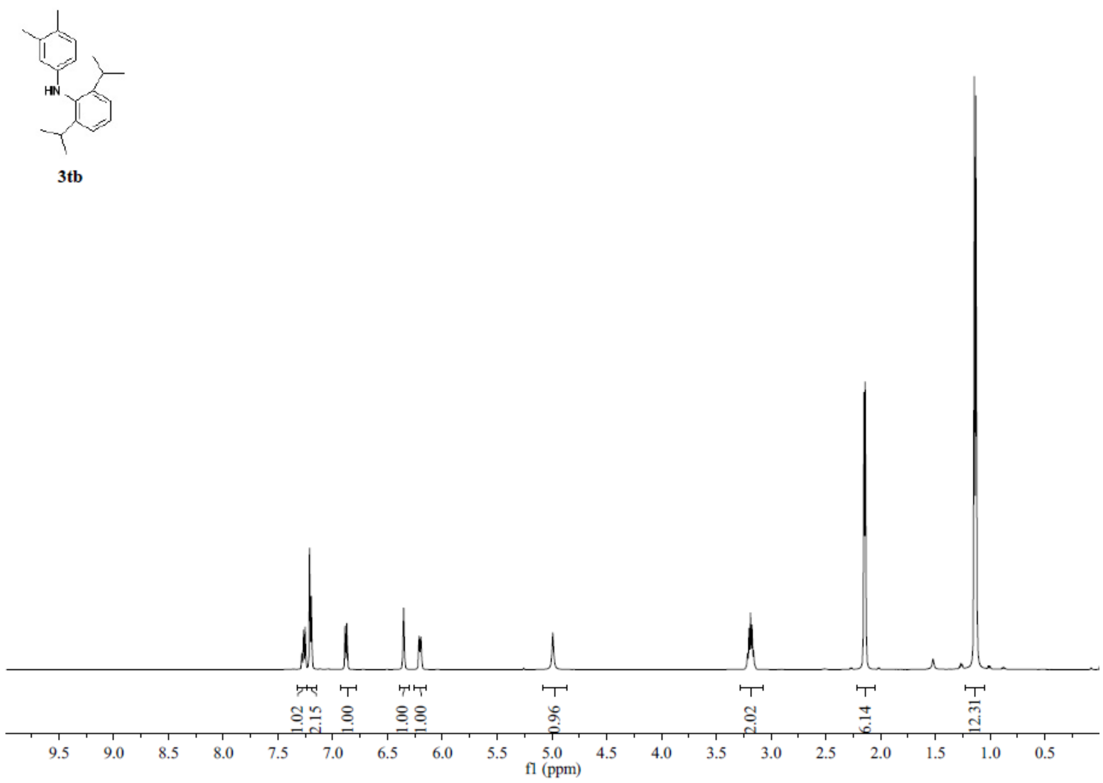

Supplementary Figure 93. ^{1}H-NMR of compound **3tb**, recorded at 500 MHz and 25 °C in $CDCl_3$.

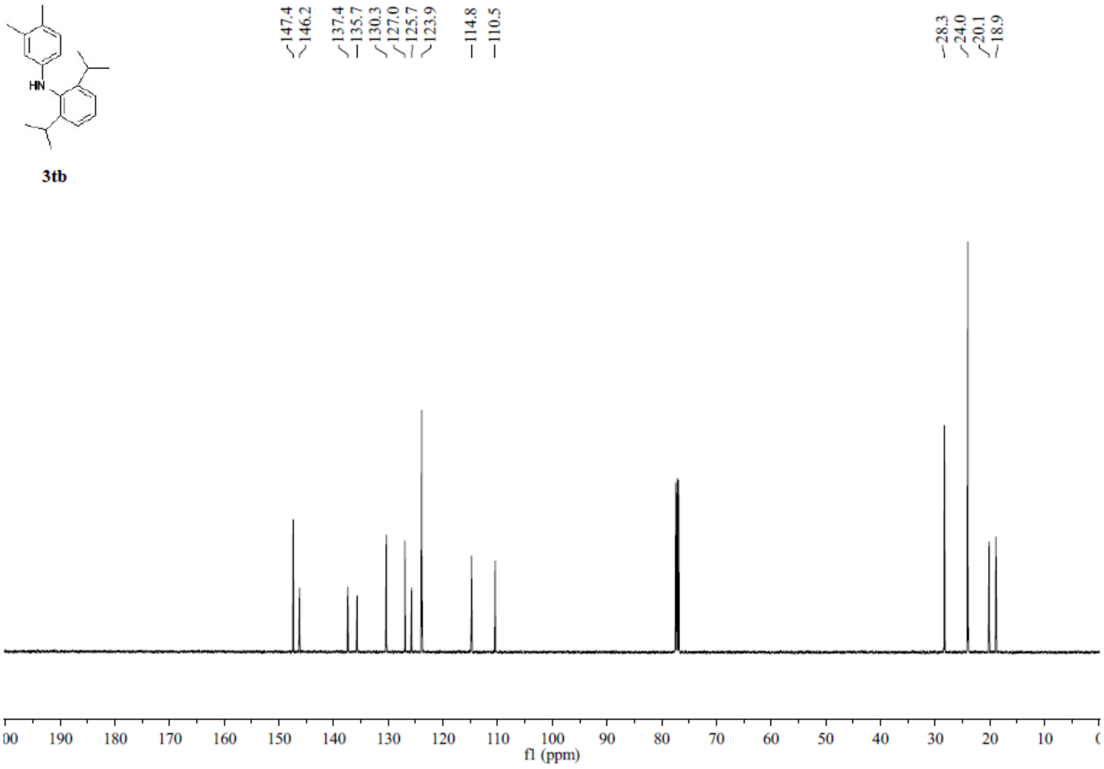

Supplementary Figure 94. ^{13}C-NMR of compound **3tb**, recorded at 125 MHz and 25 °C in $CDCl_3$.

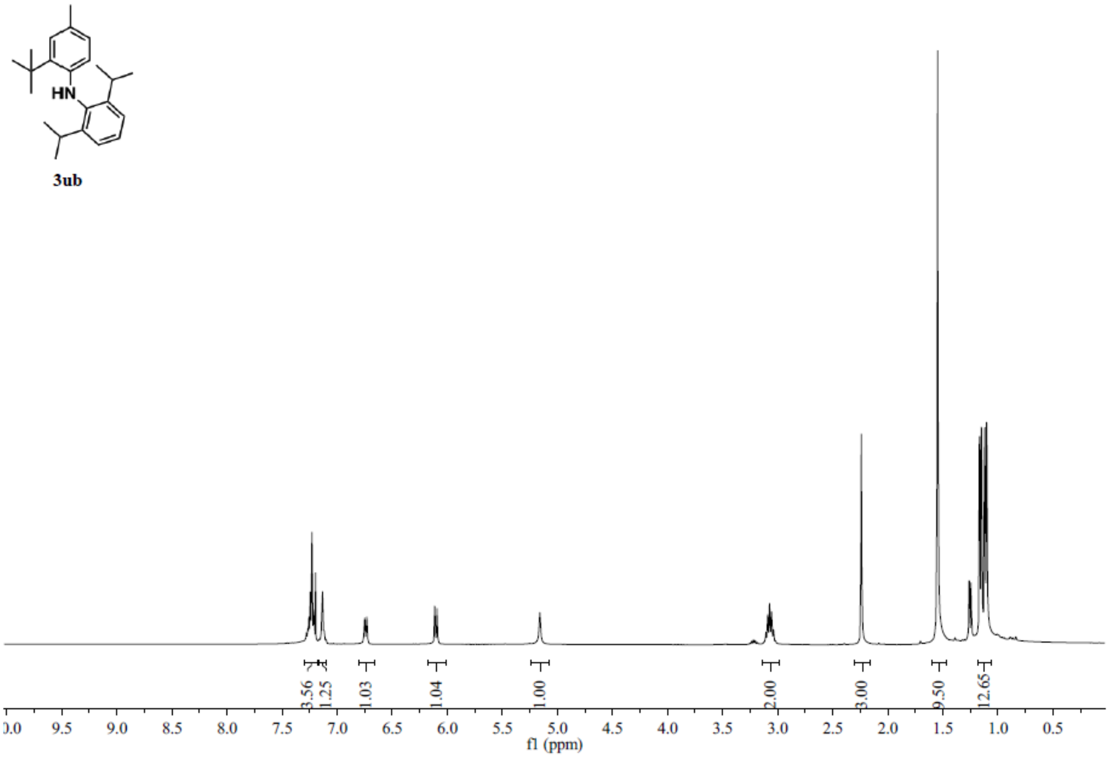

Supplementary Figure 95. ^{1}H-NMR of compound **3ub**, recorded at 400 MHz and 25 °C in $CDCl_3$.

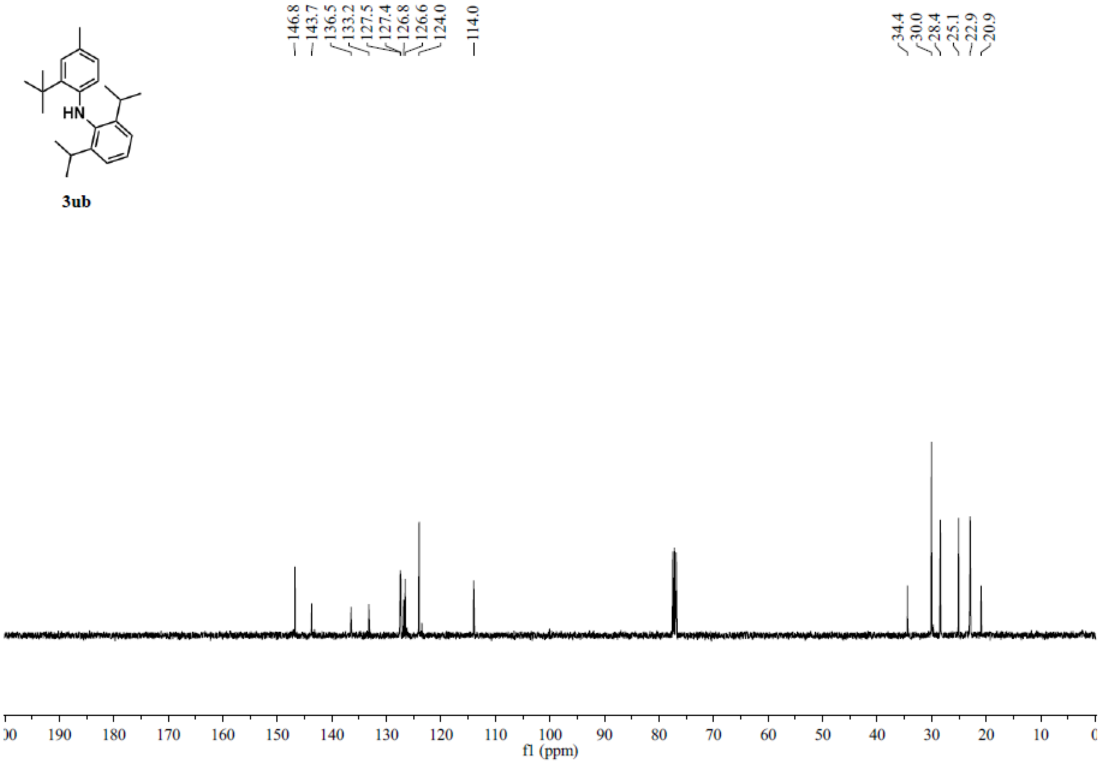

Supplementary Figure 96. ^{13}C-NMR of compound **3ub**, recorded at 125 MHz and 25 °C in $CDCl_3$.

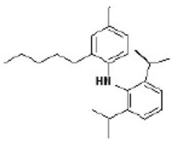

3vb

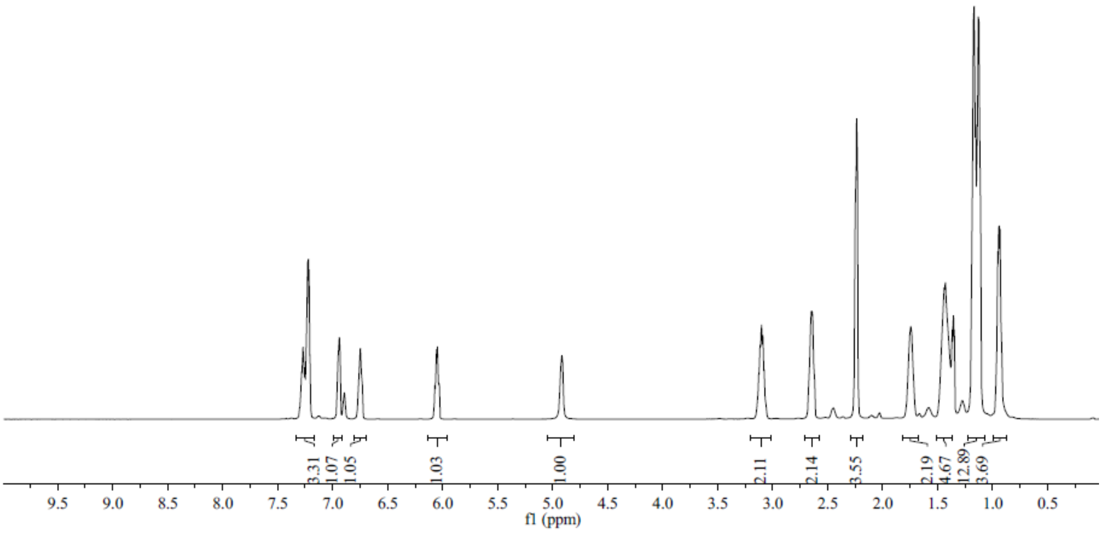

Supplementary Figure 97. ^{1}H-NMR of compound **3vb**, recorded at 500 MHz and 25 °C in $CDCl_3$.

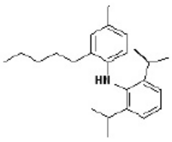

3vb

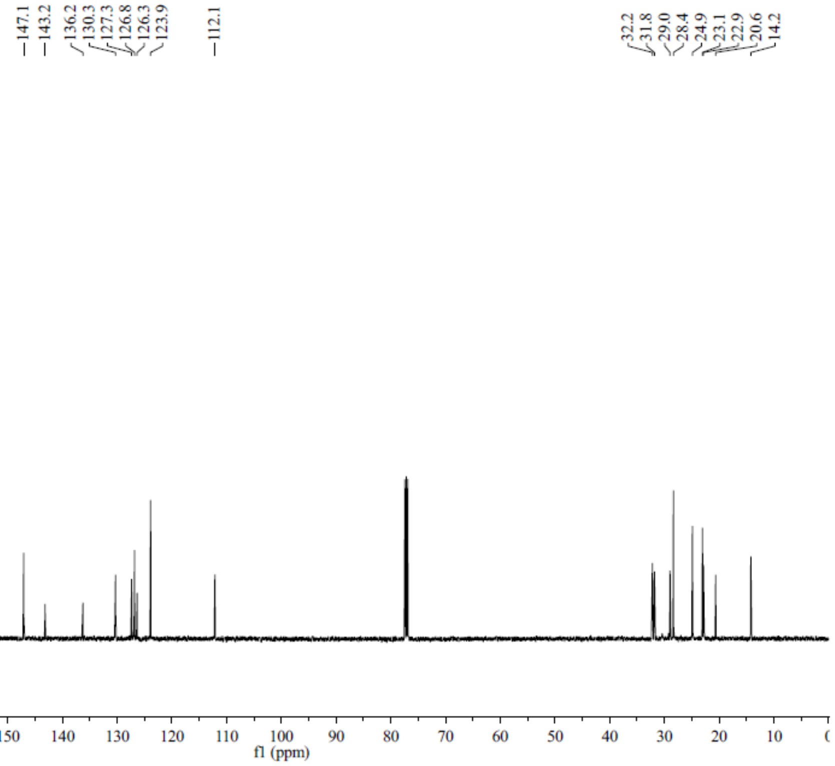

Supplementary Figure 98. ^{13}C-NMR of compound **3vb**, recorded at 125 MHz and 25 °C in $CDCl_3$.

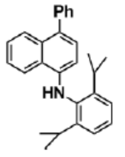

3wb

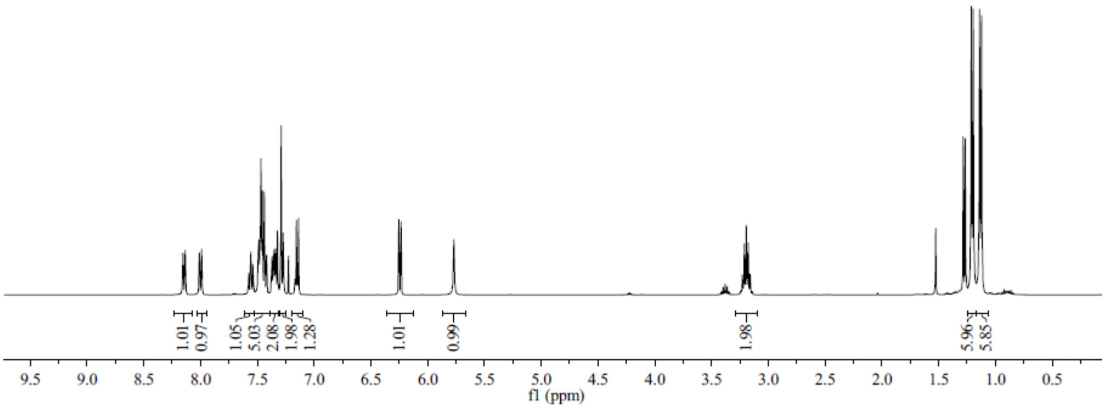

Supplementary Figure 99. ^{1}H-NMR of compound **3wb**, recorded at 400 MHz and 25 °C in $CDCl_3$.

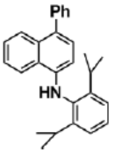

3wb

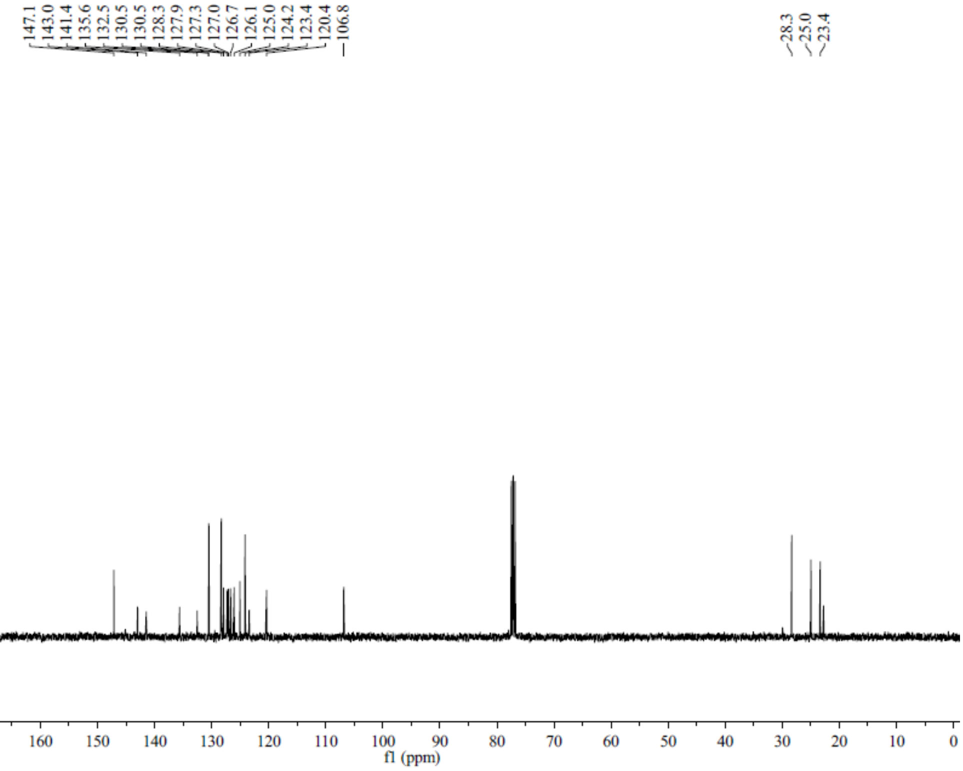

Supplementary Figure 100. ^{13}C-NMR of compound **3wb**, recorded at 100 MHz and 25 °C in $CDCl_3$.

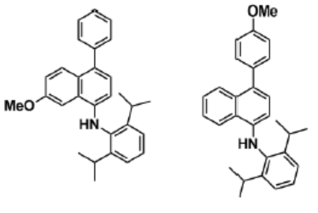

3xb and 3'xb

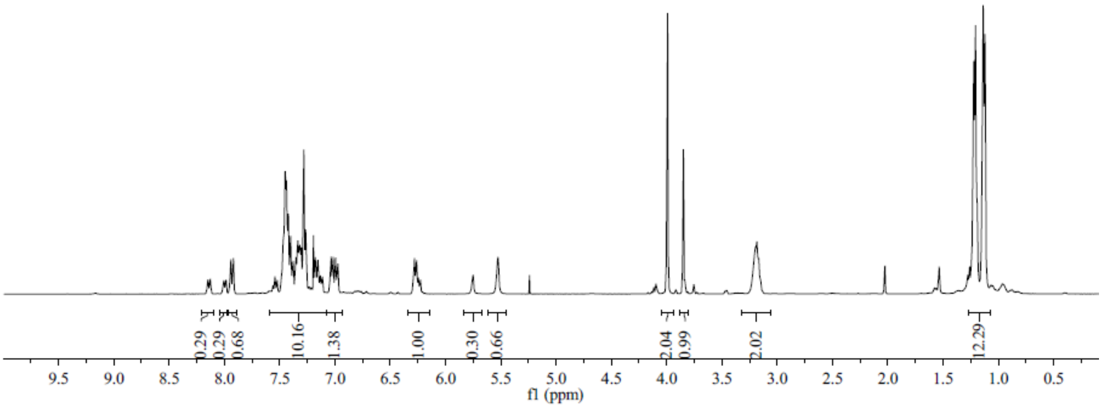

Supplementary Figure 101. ^{1}H-NMR of compound **3xb** and **3'xb**, recorded at 400 MHz and 25 °C in $CDCl_3$.

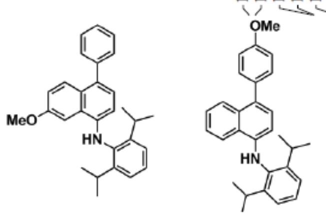

3xb and 3'xb

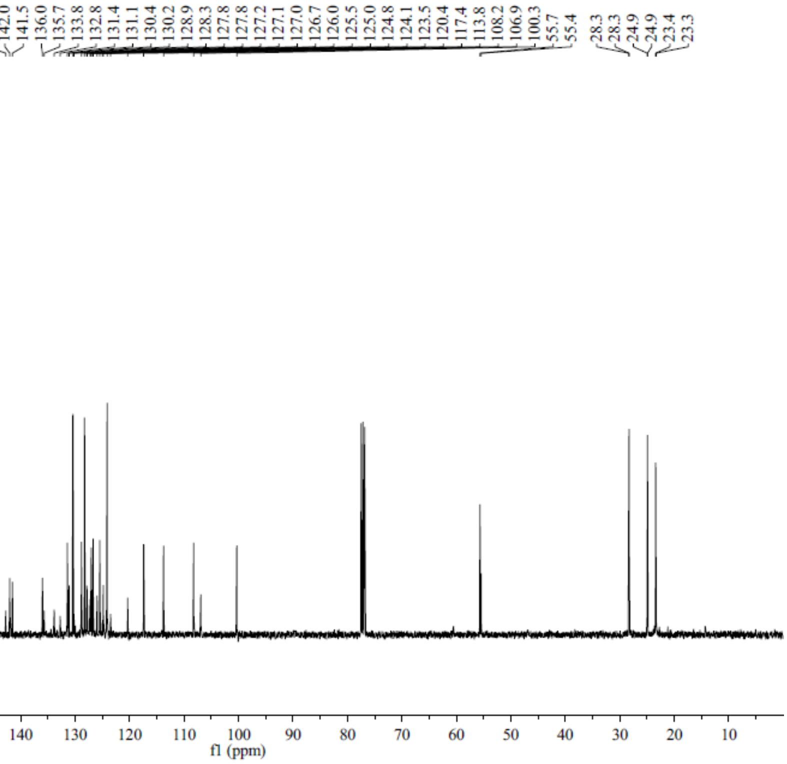

Supplementary Figure 102. ^{13}C-NMR of compound **3xb** and **3'xb**, recorded at 100 MHz and 25 °C in $CDCl_3$.

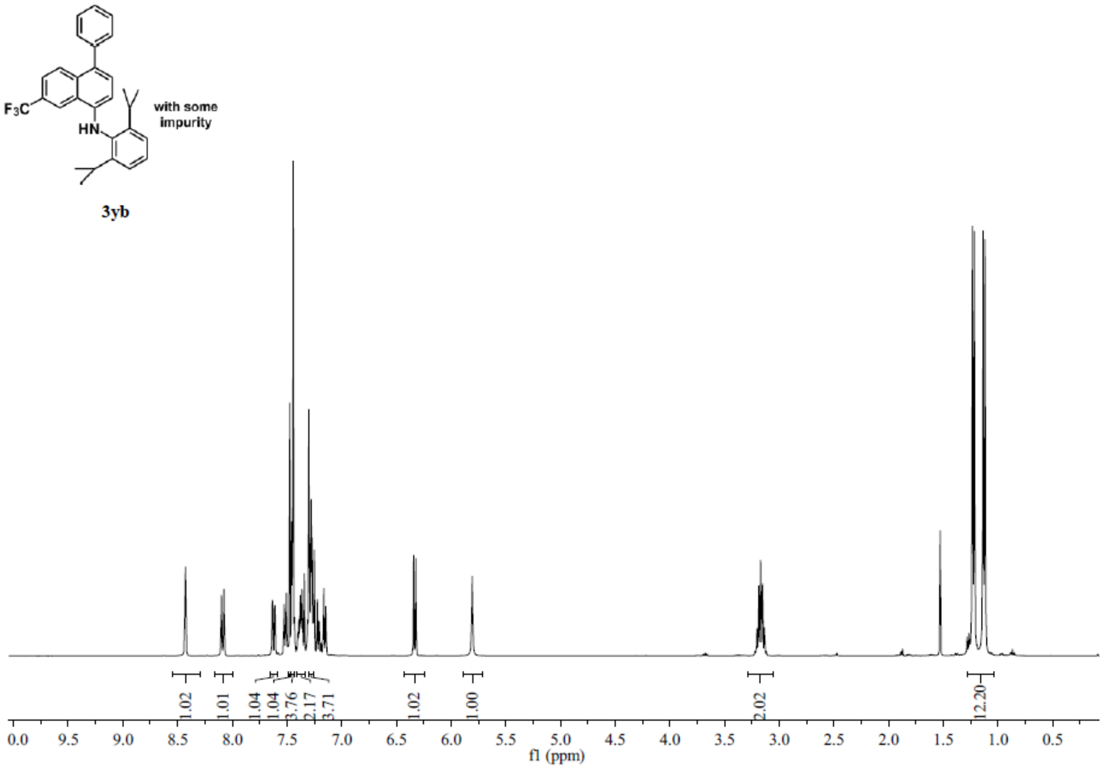

Supplementary Figure 103. ^{1}H-NMR of compound **3yb**, recorded at 400 MHz and 25 °C in $CDCl_3$.

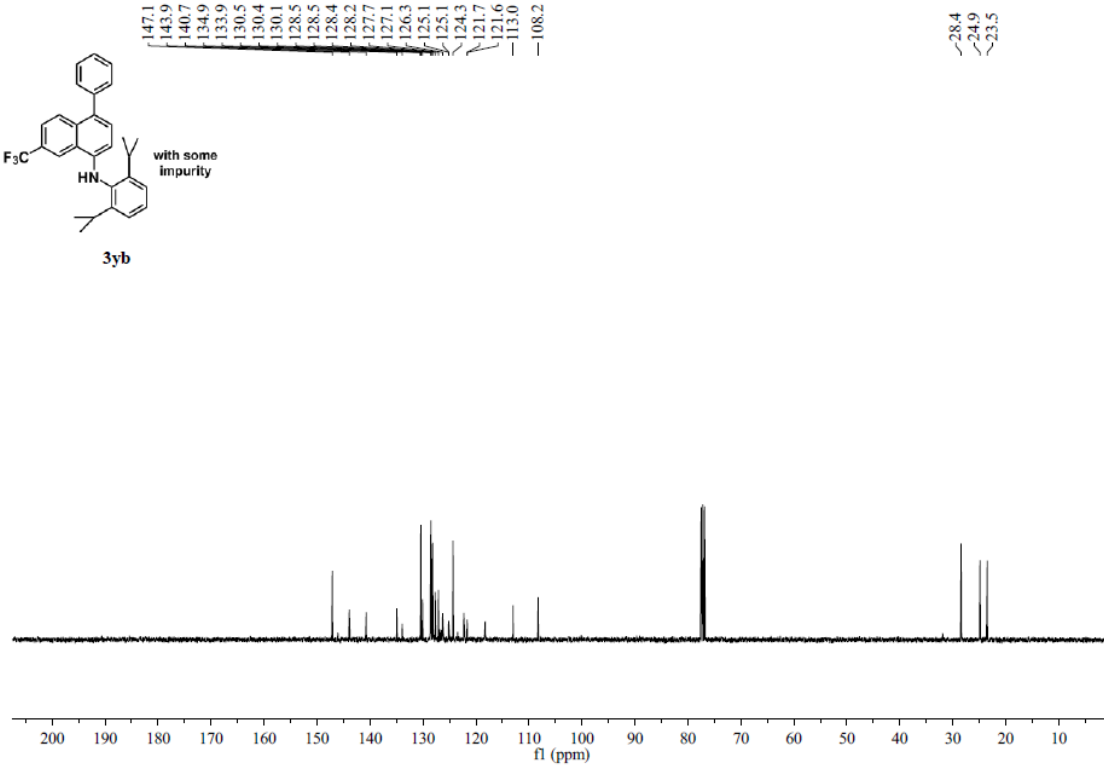

Supplementary Figure 104. ^{13}C-NMR of compound **3yb**, recorded at 100 MHz and 25 °C in $CDCl_3$.

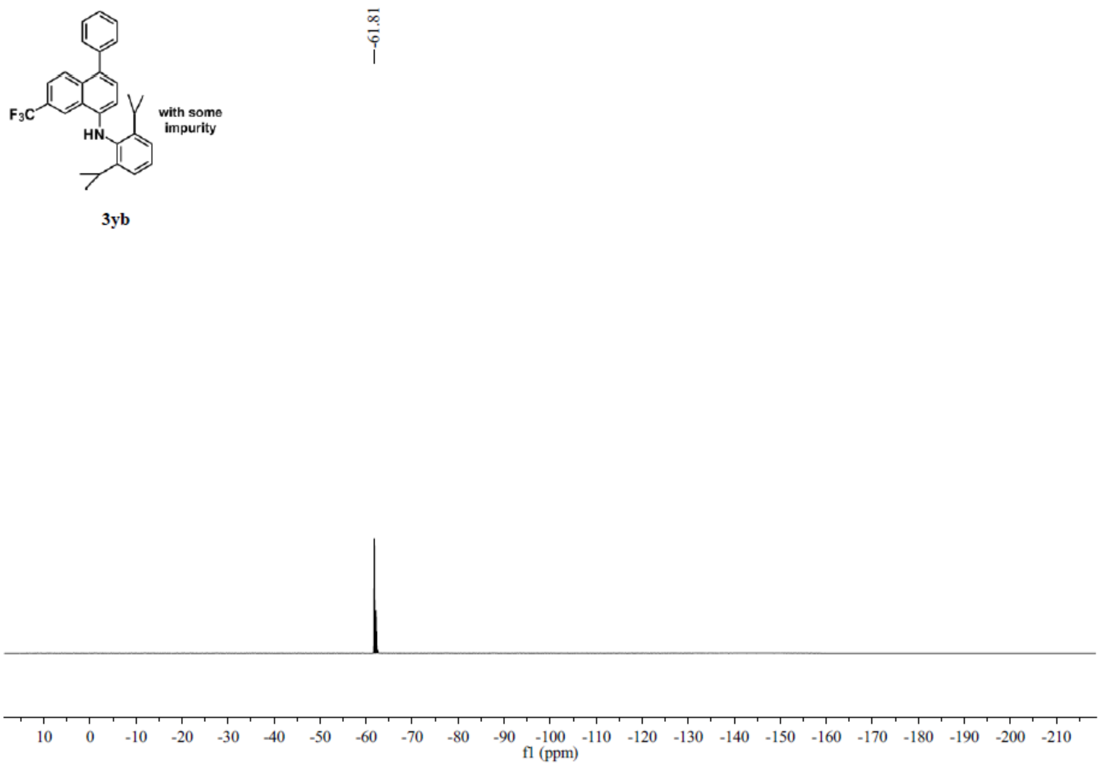

Supplementary Figure 105. ^{19}F-NMR of compound **3yb**, recorded at 376 MHz and 25 °C in $CDCl_3$.

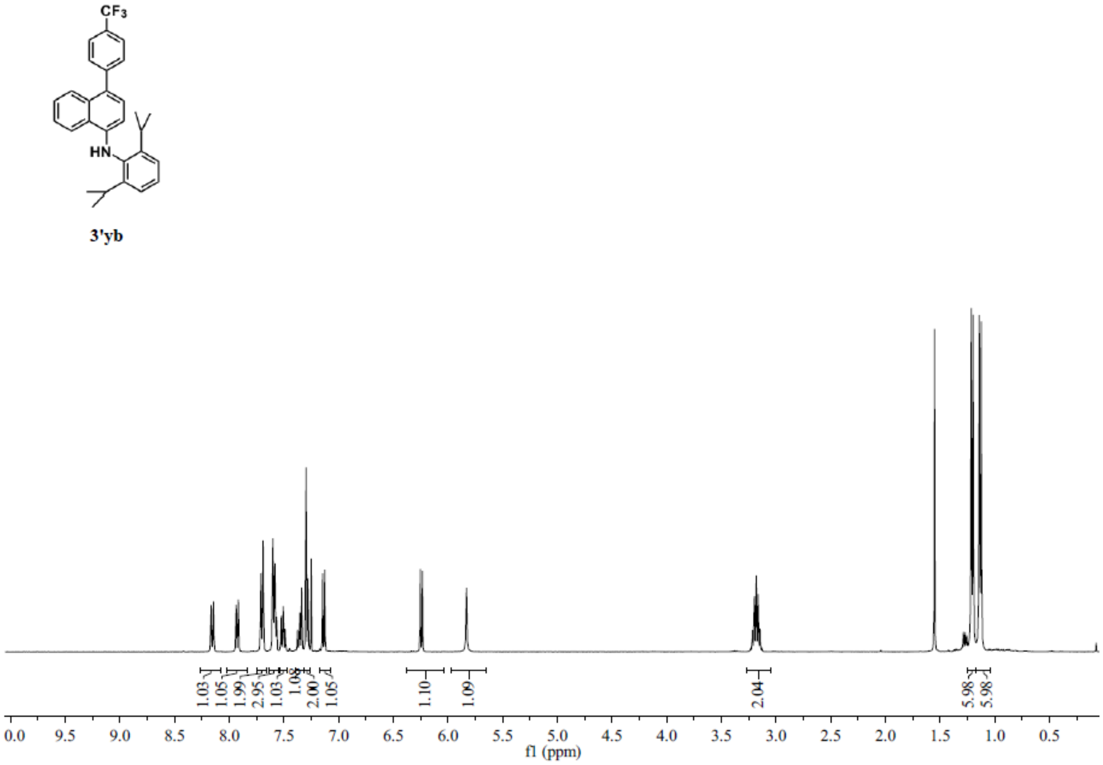

Supplementary Figure 106. ^{1}H-NMR of compound **3’yb**, recorded at 400 MHz and 25 °C in $CDCl_3$.

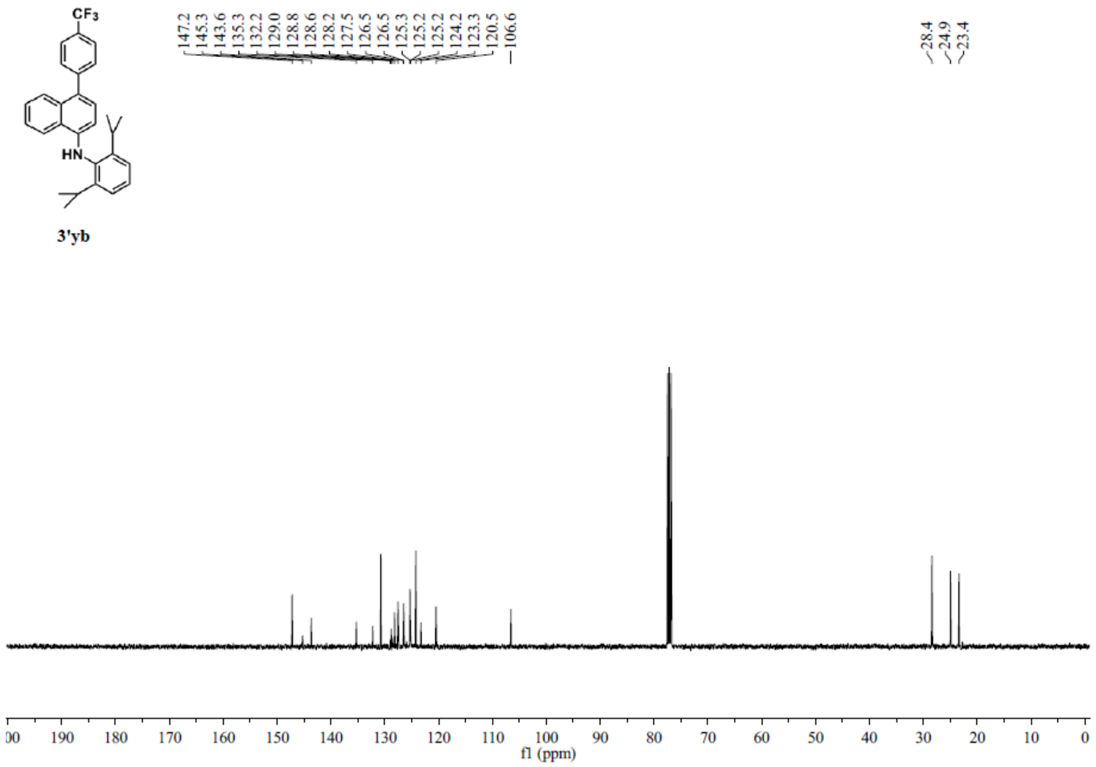

Supplementary Figure 107. ^{13}C-NMR of compound **3’yb**, recorded at 100 MHz and 25 °C in $CDCl_3$.

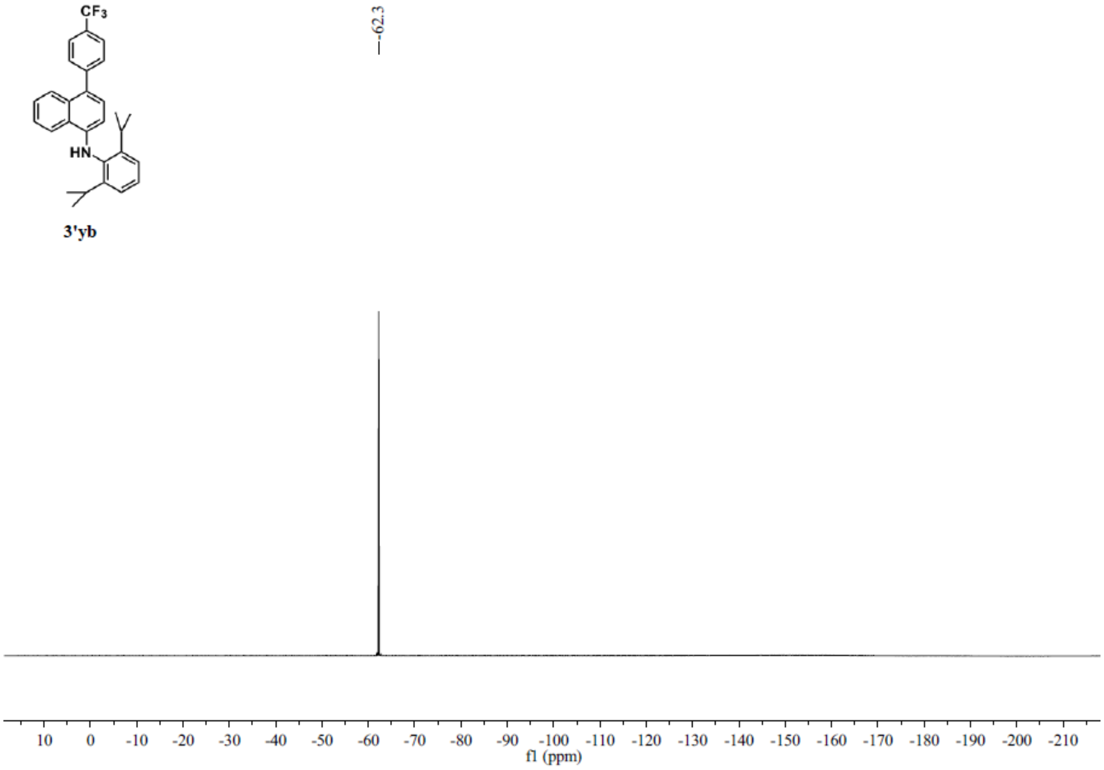

Supplementary Figure 108. ^{19}F-NMR of compound **3’yb**, recorded at 376 MHz and 25 °C in $CDCl_3$.

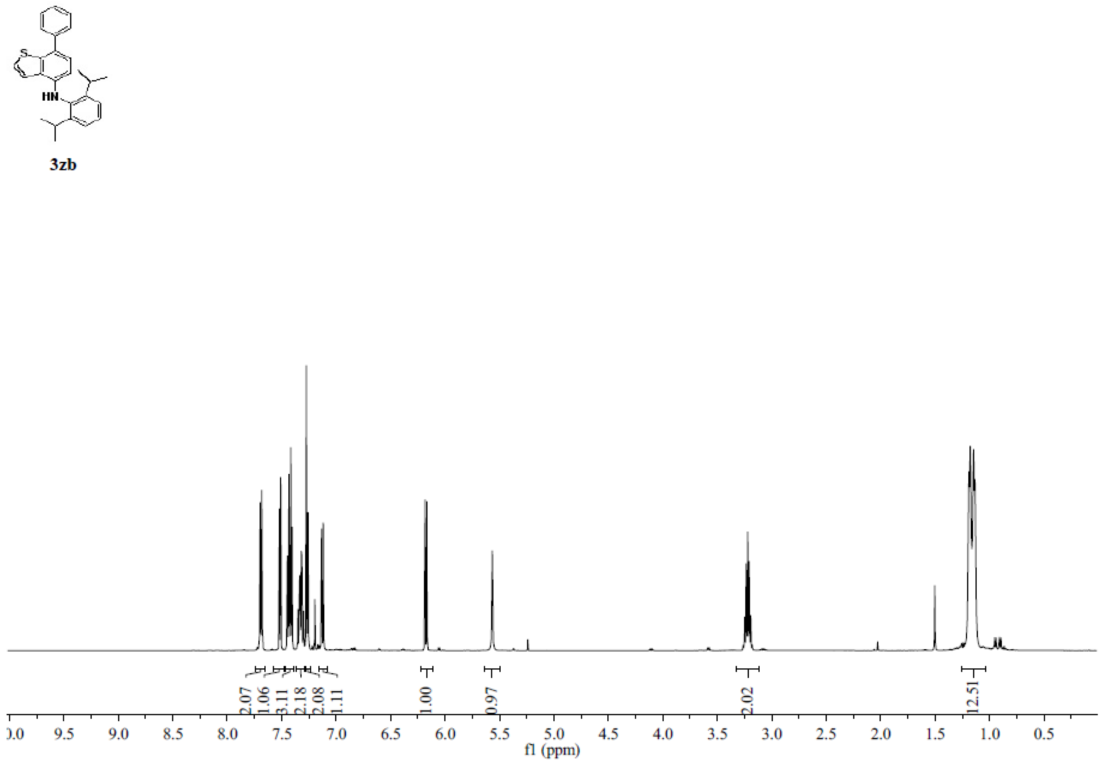

Supplementary Figure 109. ^{1}H-NMR of compound **3zb**, recorded at 500 MHz and 25 °C in $CDCl_3$.

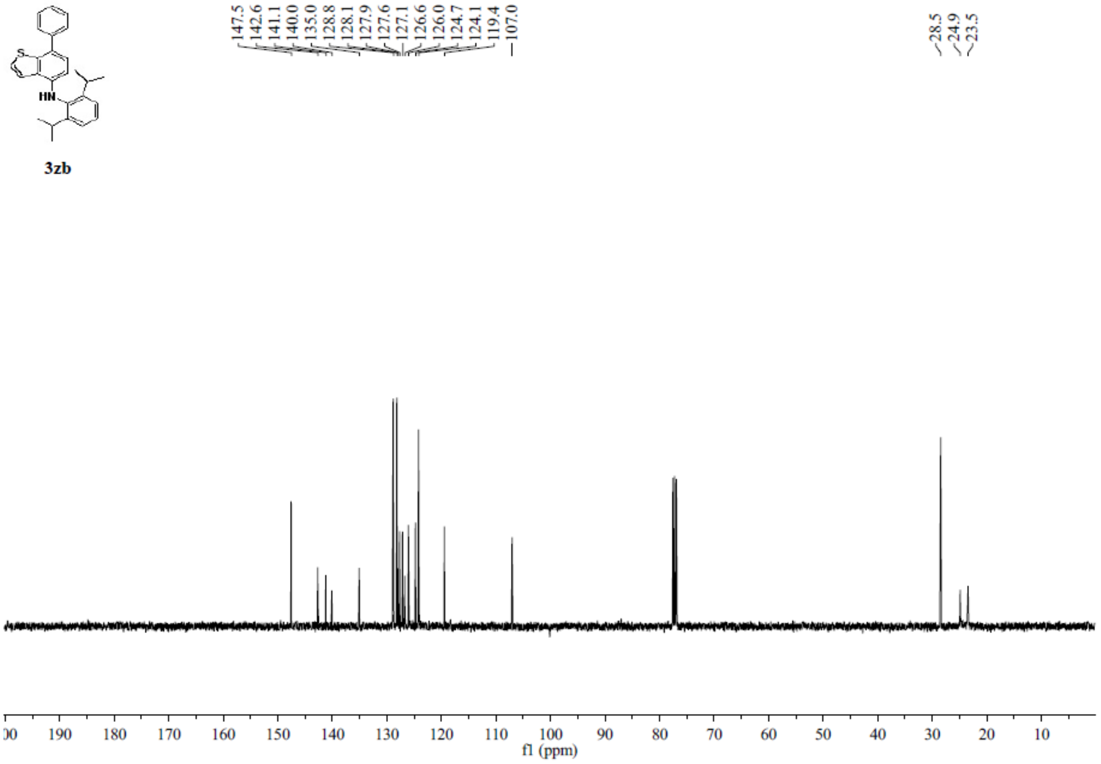

Supplementary Figure 110. ^{13}C-NMR of compound **3zb**, recorded at 100 MHz and 25 °C in $CDCl_3$.

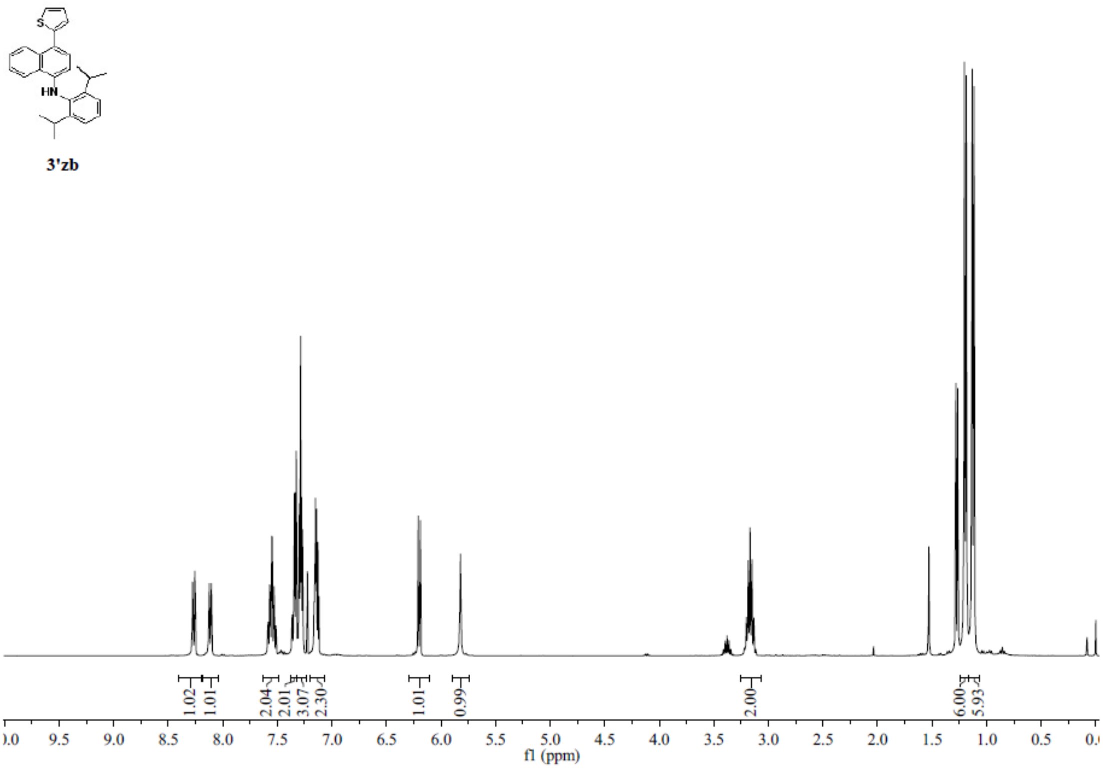

Supplementary Figure 111. ^{1}H-NMR of compound **3’zb**, recorded at 400 MHz and 25 °C in $CDCl_3$.

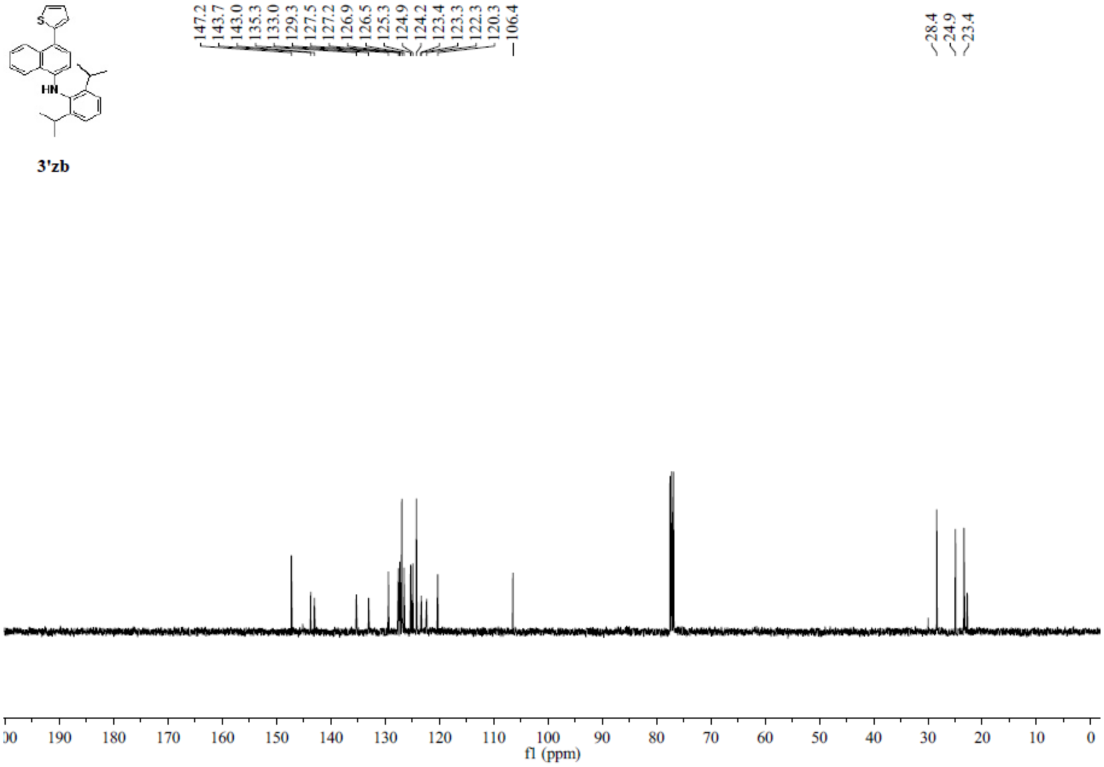

Supplementary Figure 112. ^{13}C-NMR of compound **3’zb**, recorded at 100 MHz and 25 °C in $CDCl_3$.

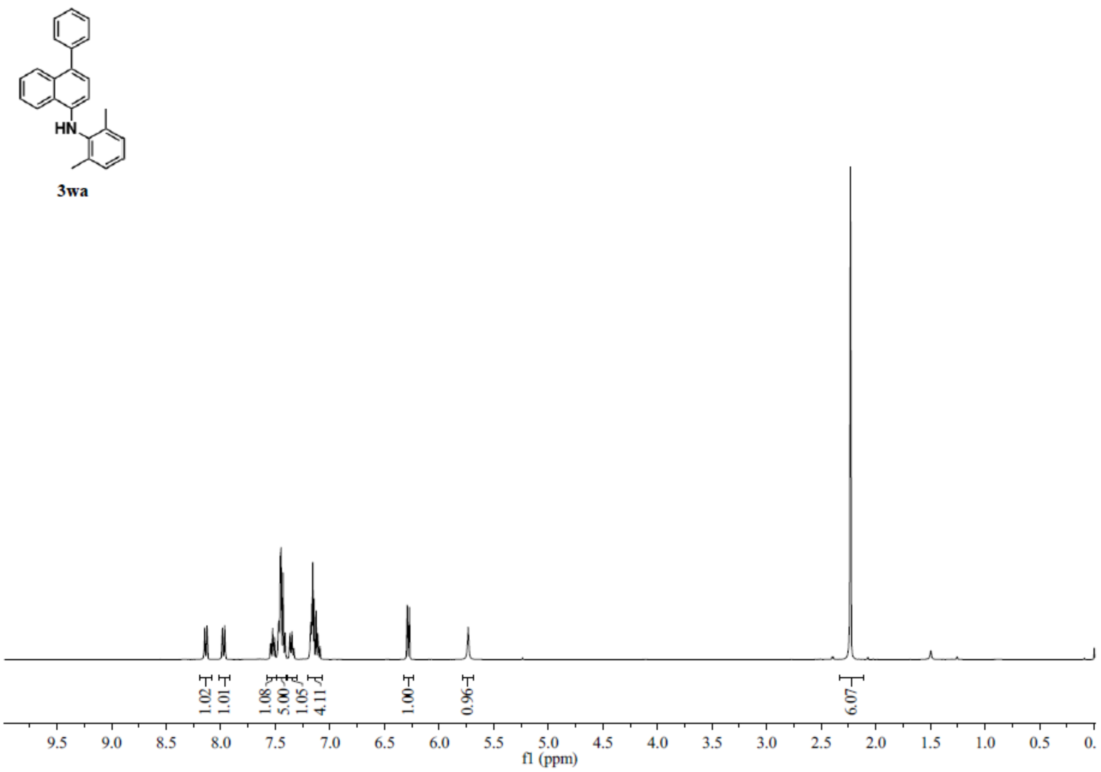

Supplementary Figure 113. ^{1}H-NMR of compound **3wa**, recorded at 400 MHz and 25 °C in $CDCl_3$.

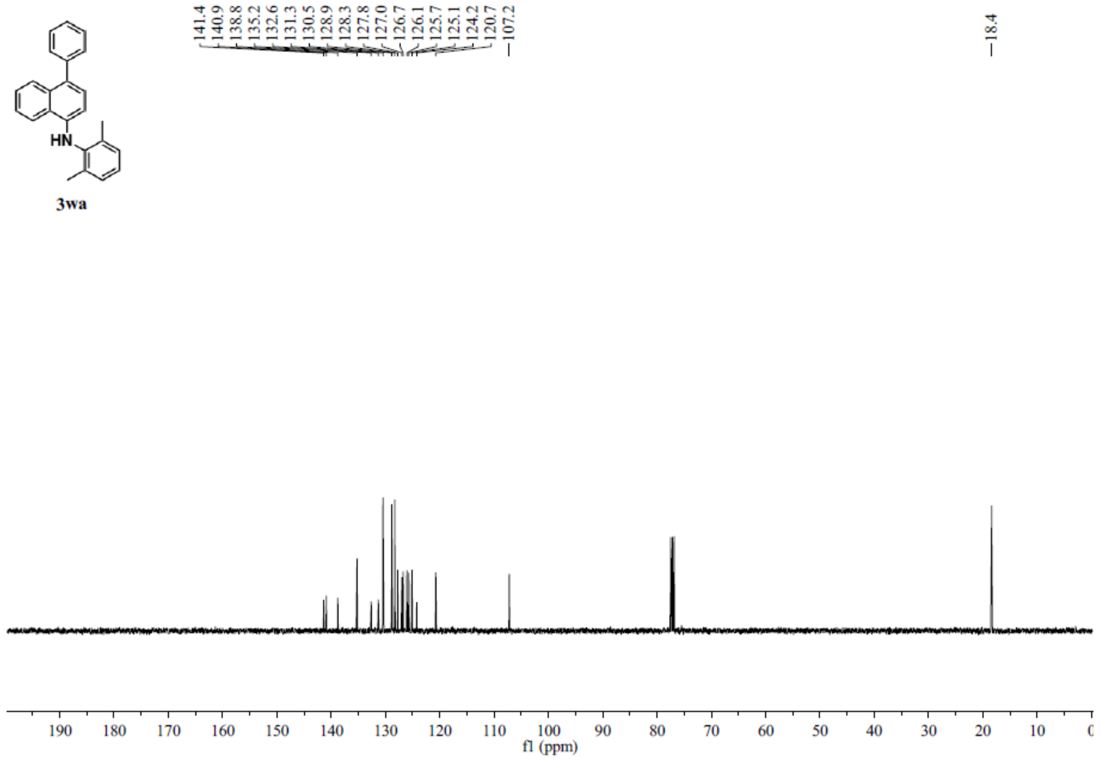

Supplementary Figure 114. ^{13}C-NMR of compound **3wa**, recorded at 100 MHz and 25 °C in $CDCl_3$.

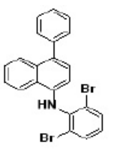

3wc

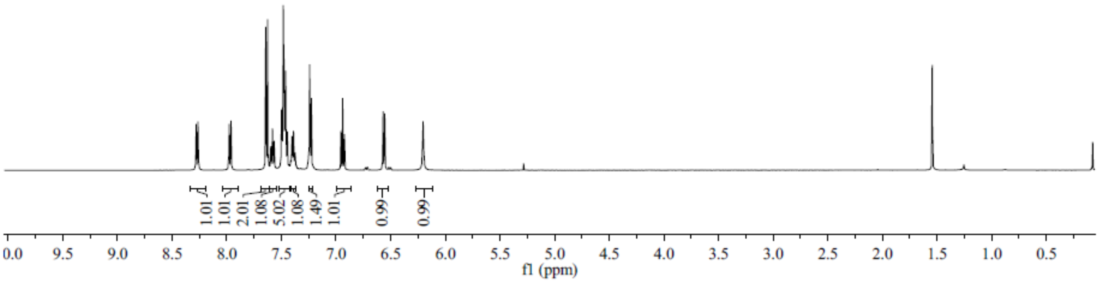

Supplementary Figure 115. ^{1}H-NMR of compound **3wc**, recorded at 500 MHz and 25 °C in $CDCl_3$.

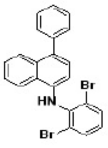

3wc

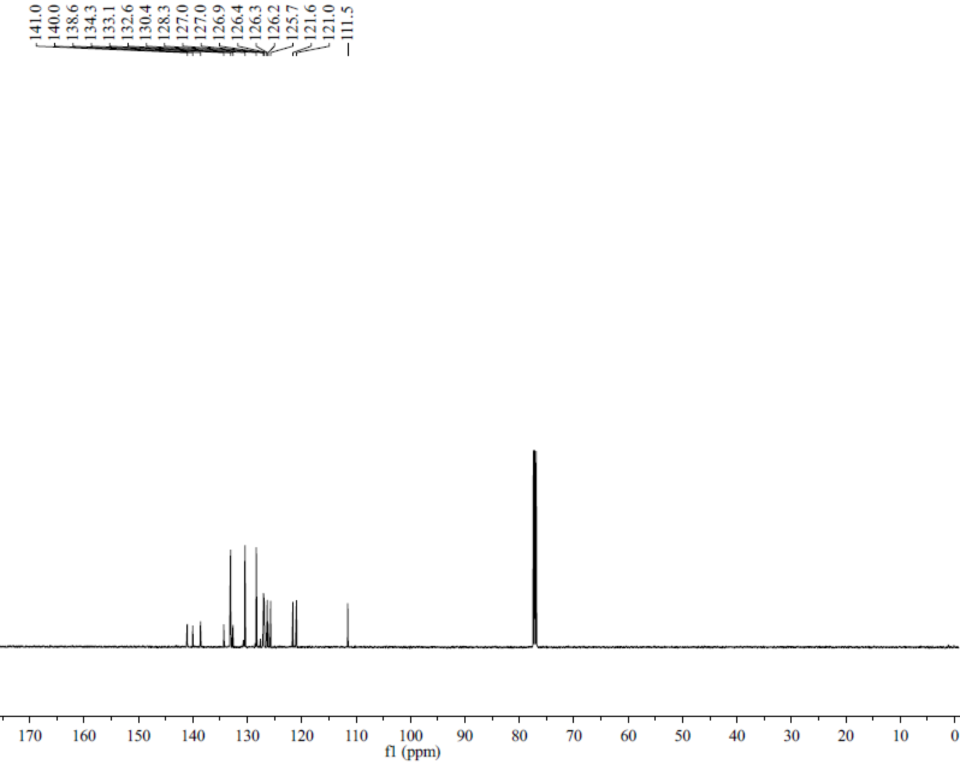

Supplementary Figure 116. ^{13}C-NMR of compound **3wc**, recorded at 125 MHz and 25 °C in $CDCl_3$.

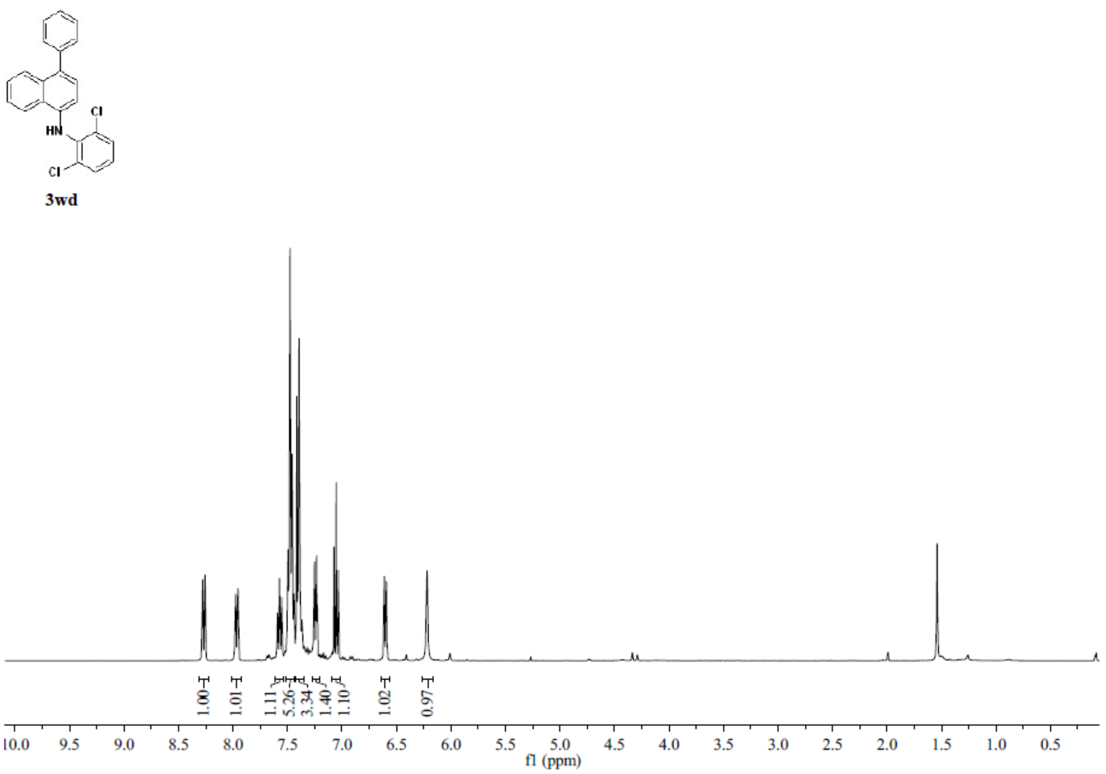

Supplementary Figure 117. ^{1}H-NMR of compound **3wd**, recorded at 400 MHz and 25 °C in $CDCl_3$.

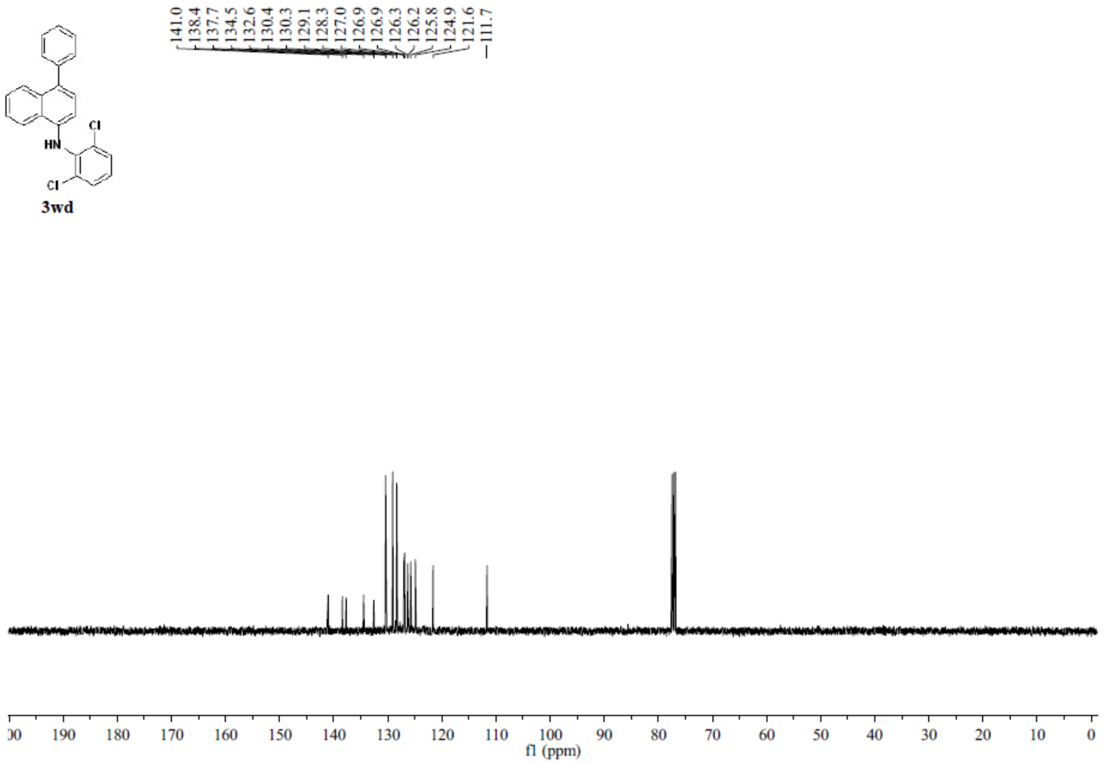

Supplementary Figure 118. ^{13}C-NMR of compound **3wd**, recorded at 100 MHz and 25 °C in $CDCl_3$.

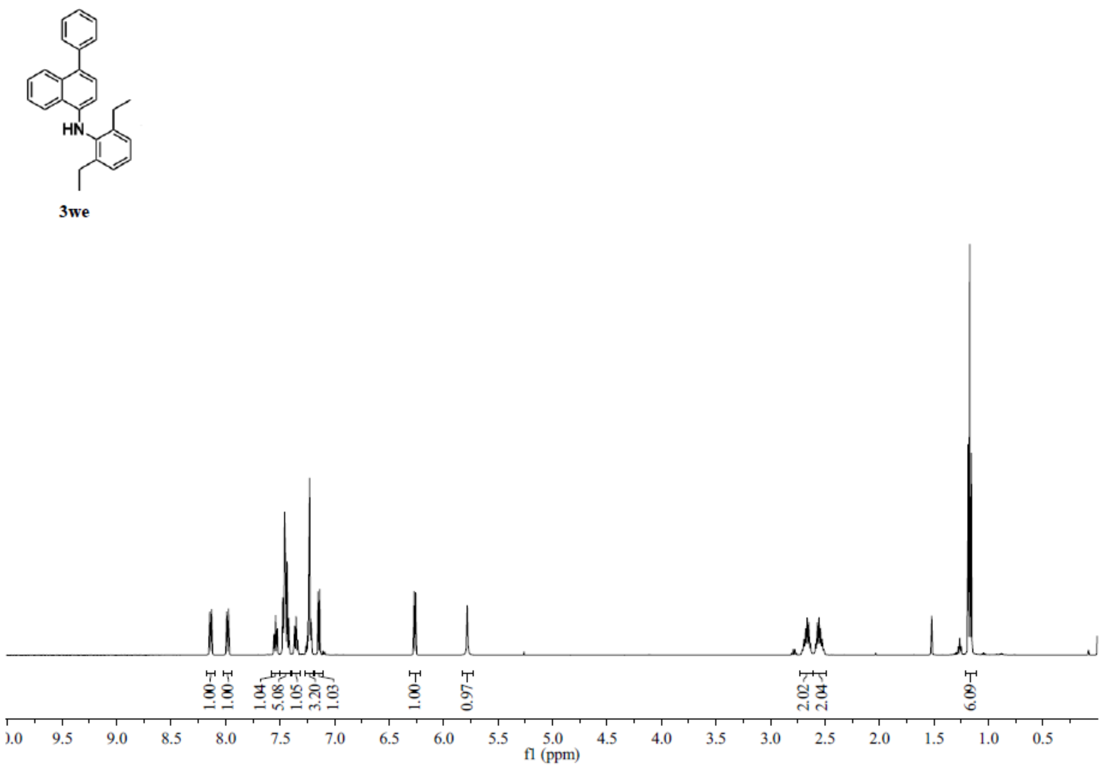

Supplementary Figure 119. ^{1}H-NMR of compound **3we**, recorded at 500 MHz and 25 °C in $CDCl_3$.

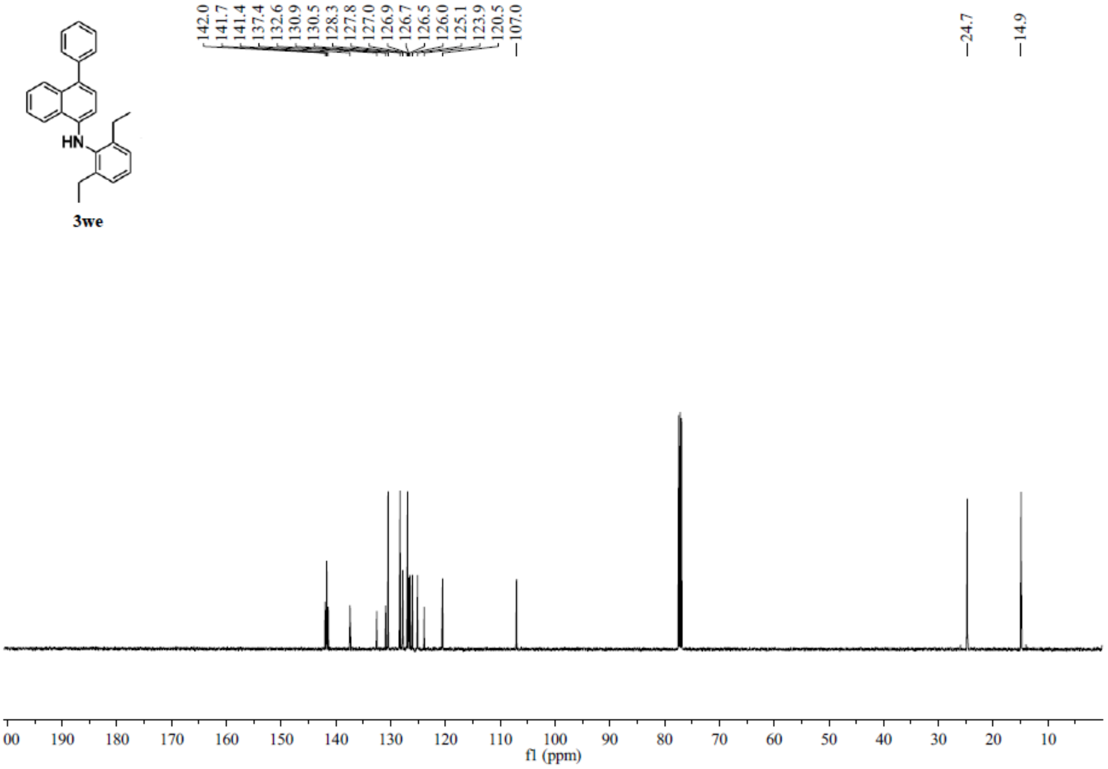

Supplementary Figure 120. ^{13}C-NMR of compound **3we**, recorded at 125 MHz and 25 °C in $CDCl_3$.

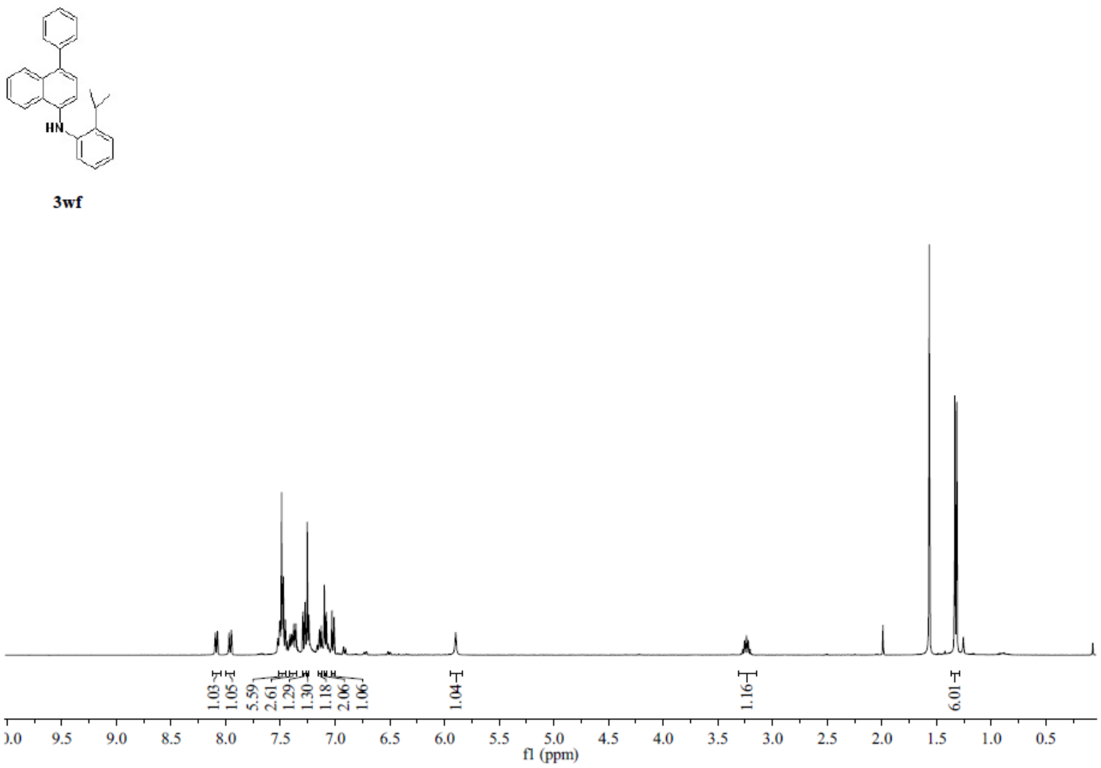

Supplementary Figure 121. ^{1}H-NMR of compound **3wf**, recorded at 400 MHz and 25 °C in $CDCl_3$.

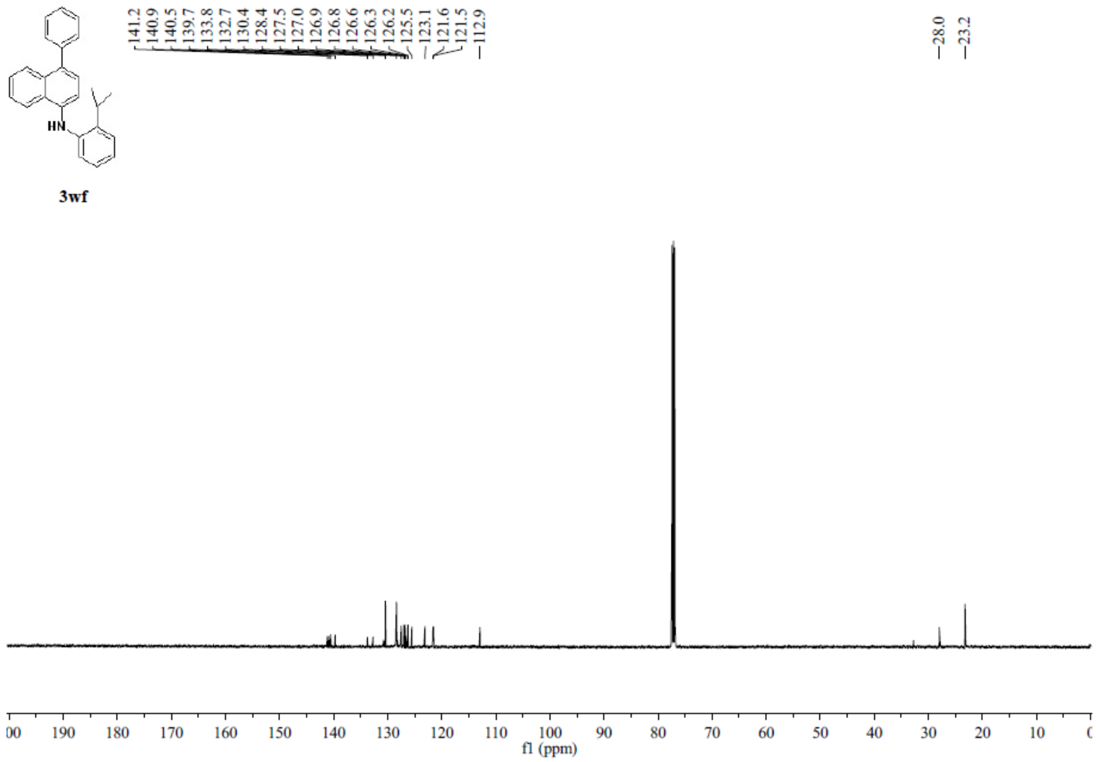

Supplementary Figure 122. ^{13}C-NMR of compound **3wf**, recorded at 100 MHz and 25 °C in $CDCl_3$.

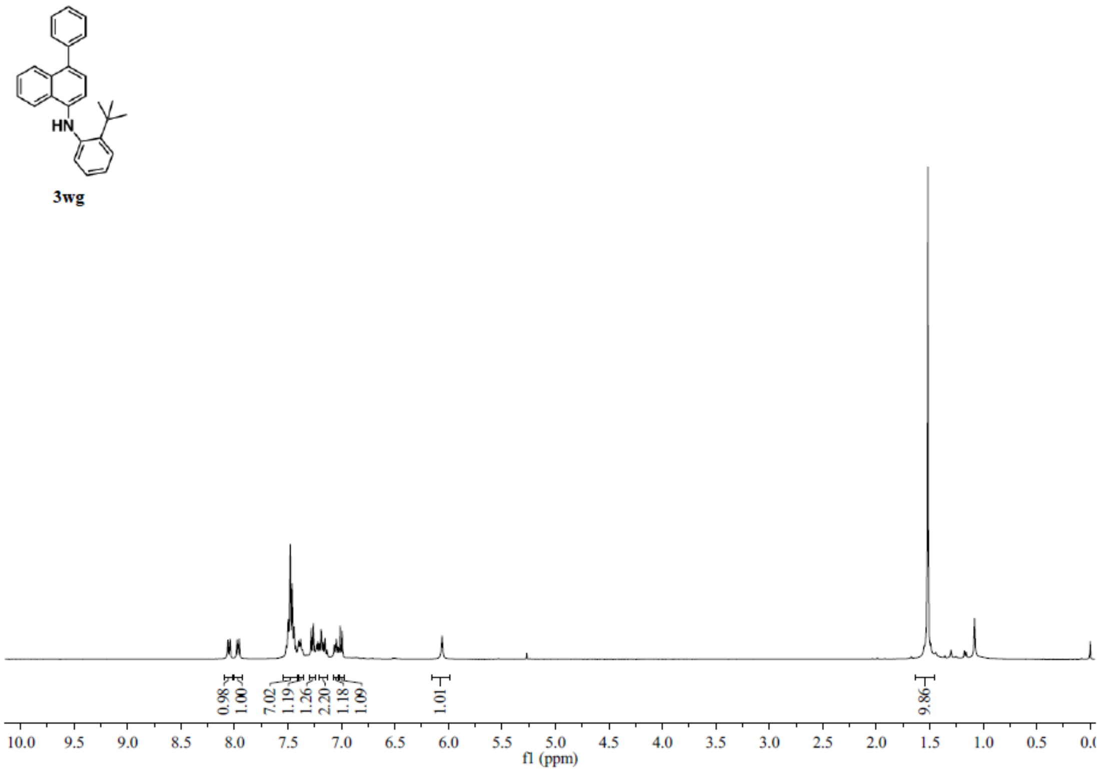

Supplementary Figure 123. ^{1}H-NMR of compound **3wg**, recorded at 400 MHz and 25 °C in $CDCl_3$.

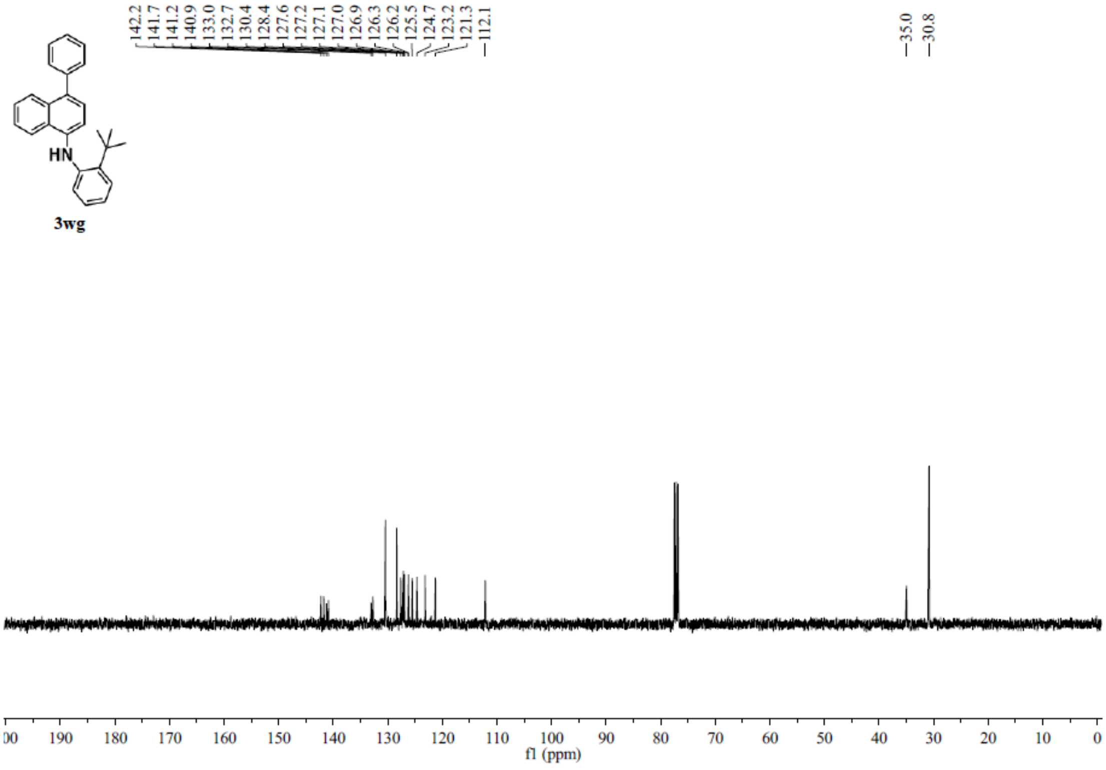

Supplementary Figure 124. ^{13}C-NMR of compound **3wg**, recorded at 100 MHz and 25 °C in $CDCl_3$.

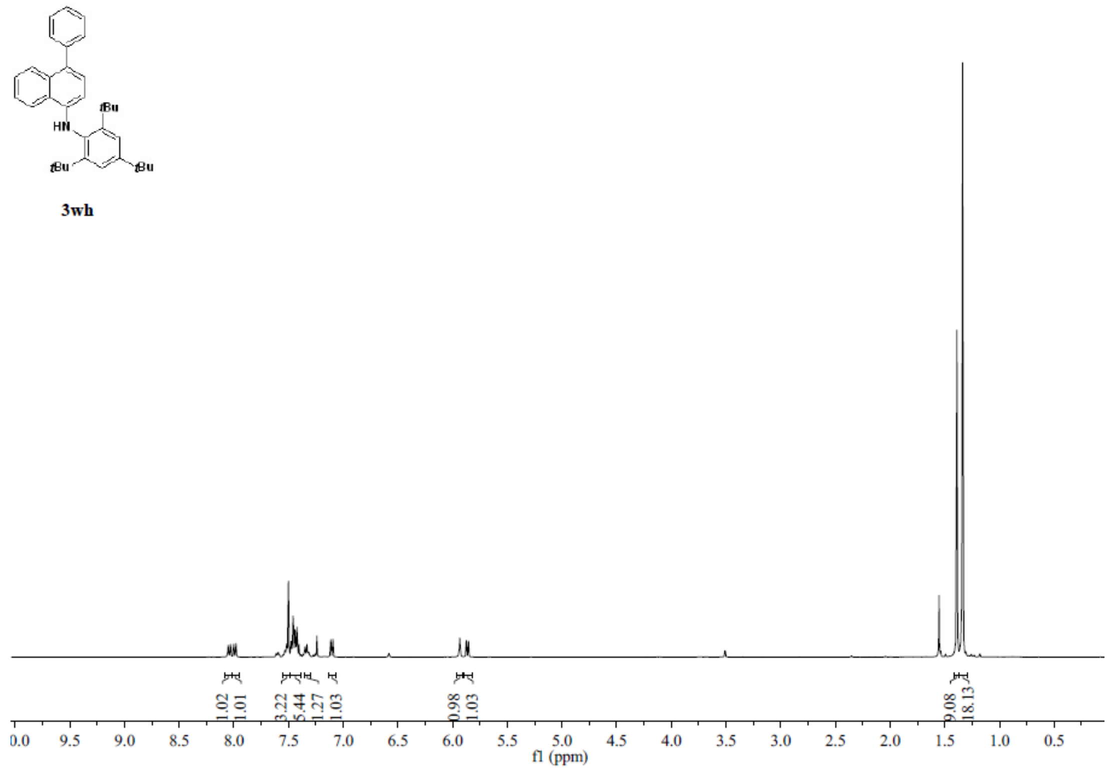

Supplementary Figure 125. ^{1}H-NMR of compound **3wh**, recorded at 400 MHz and 25 °C in $CDCl_3$.

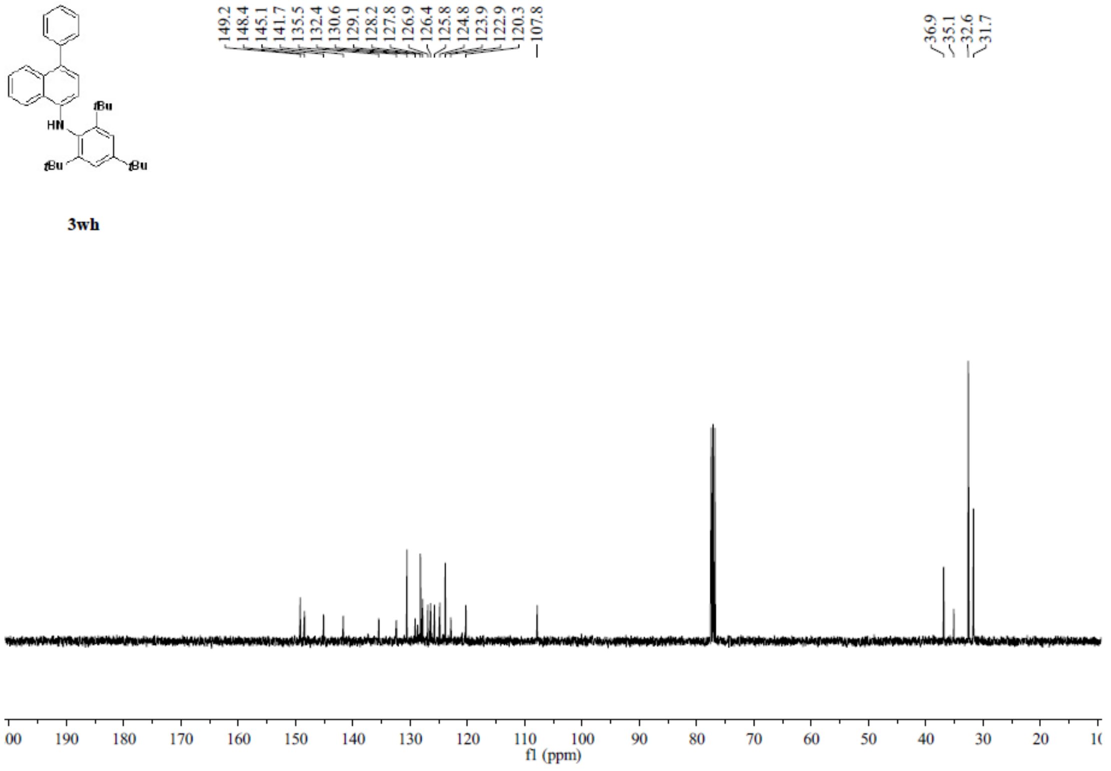

Supplementary Figure 126. ^{13}C-NMR of compound **3wh**, recorded at 100 MHz and 25 °C in $CDCl_3$.

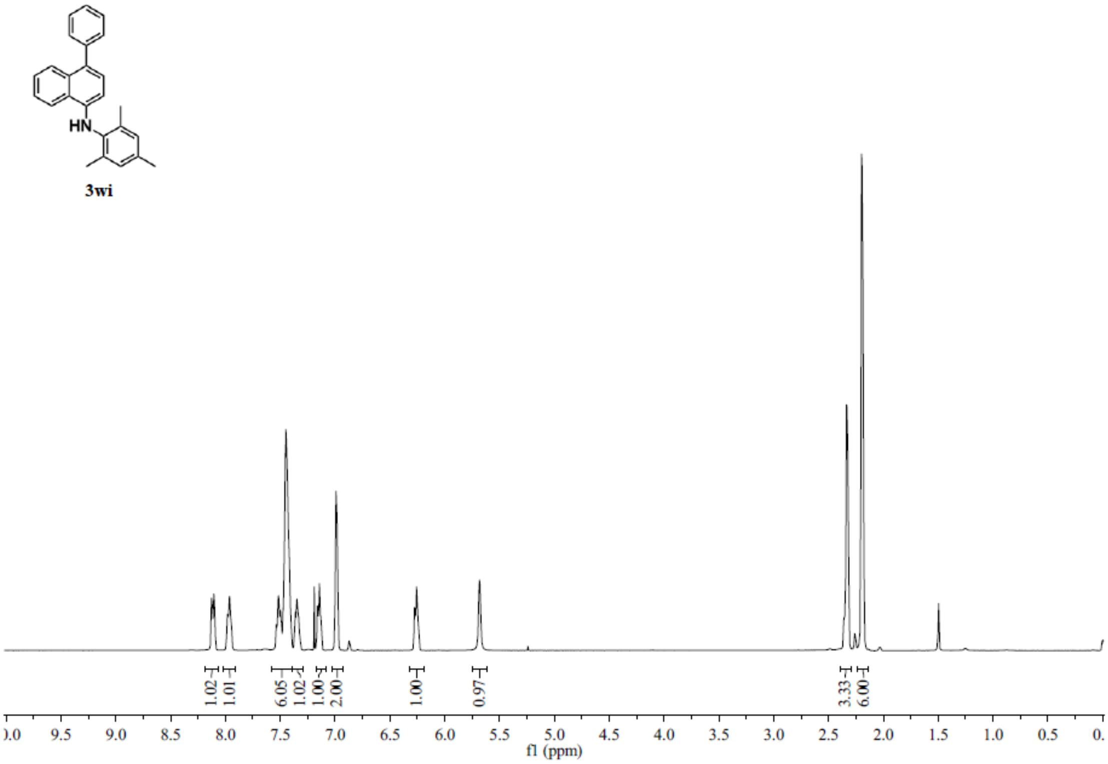

Supplementary Figure 127. ^{1}H-NMR of compound **3wi**, recorded at 400 MHz and 25 °C in $CDCl_3$.

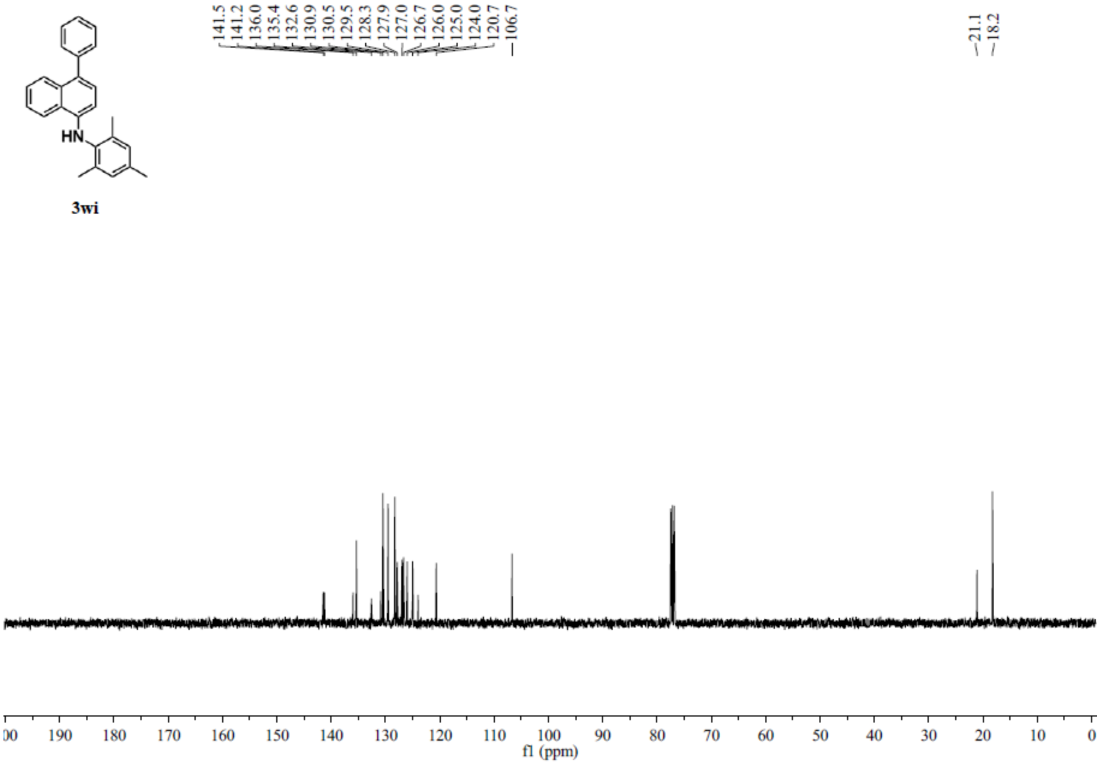

Supplementary Figure 128. ^{13}C-NMR of compound **3wi**, recorded at 100 MHz and 25 °C in $CDCl_3$.

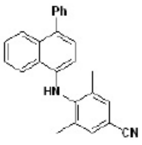

3wj

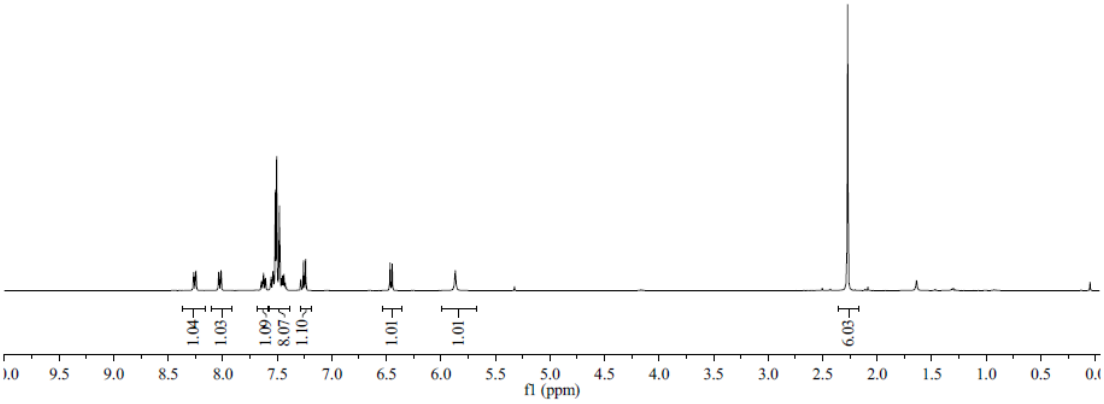

Supplementary Figure 129. ^{1}H-NMR of compound **3wj**, recorded at 400 MHz and 25 °C in $CDCl_3$.

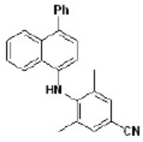

3wj

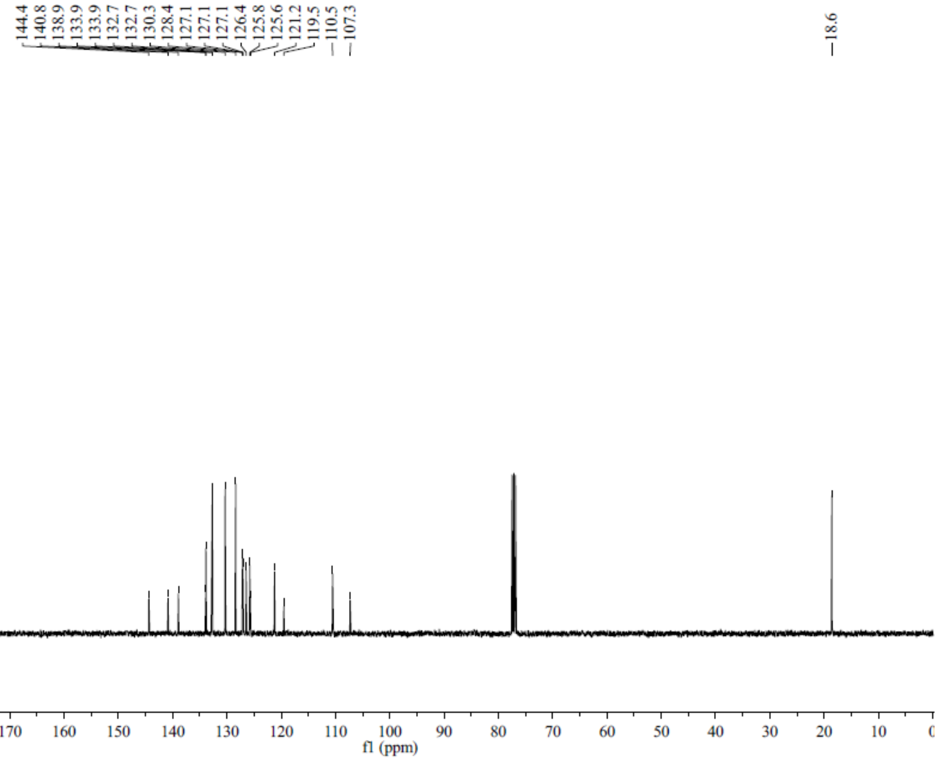

Supplementary Figure 130. ^{13}C-NMR of compound **3wj**, recorded at 100 MHz and 25 °C in $CDCl_3$.

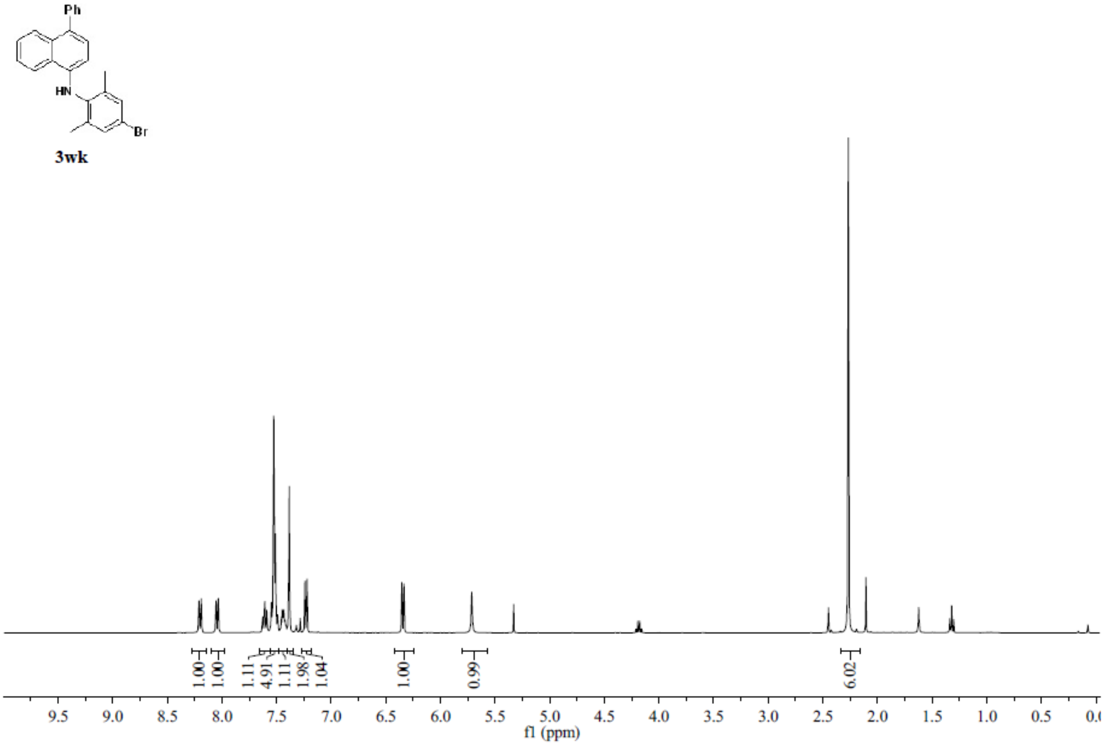

Supplementary Figure 131. ^{1}H-NMR of compound **3wk**, recorded at 400 MHz and 25 °C in $CDCl_3$.

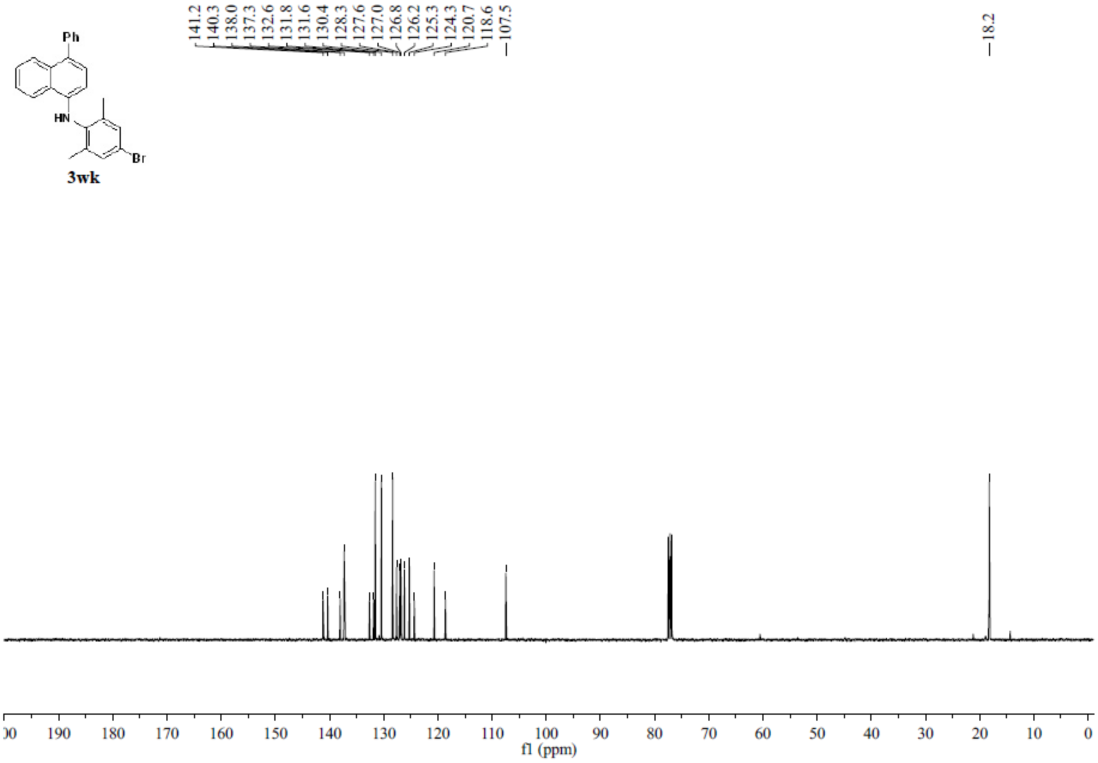

Supplementary Figure 132. ^{13}C-NMR of compound **3wk**, recorded at 100 MHz and 25 °C in $CDCl_3$.

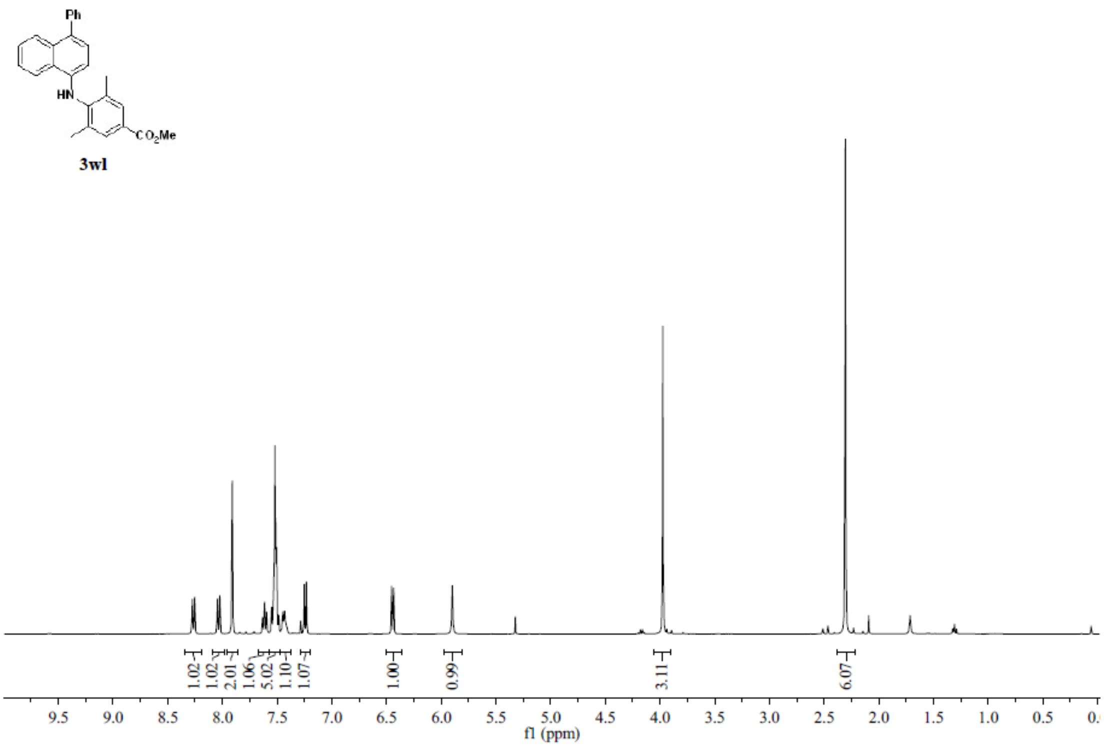

Supplementary Figure 133. ^{1}H-NMR of compound **3wl**, recorded at 400 MHz and 25 °C in $CDCl_3$.

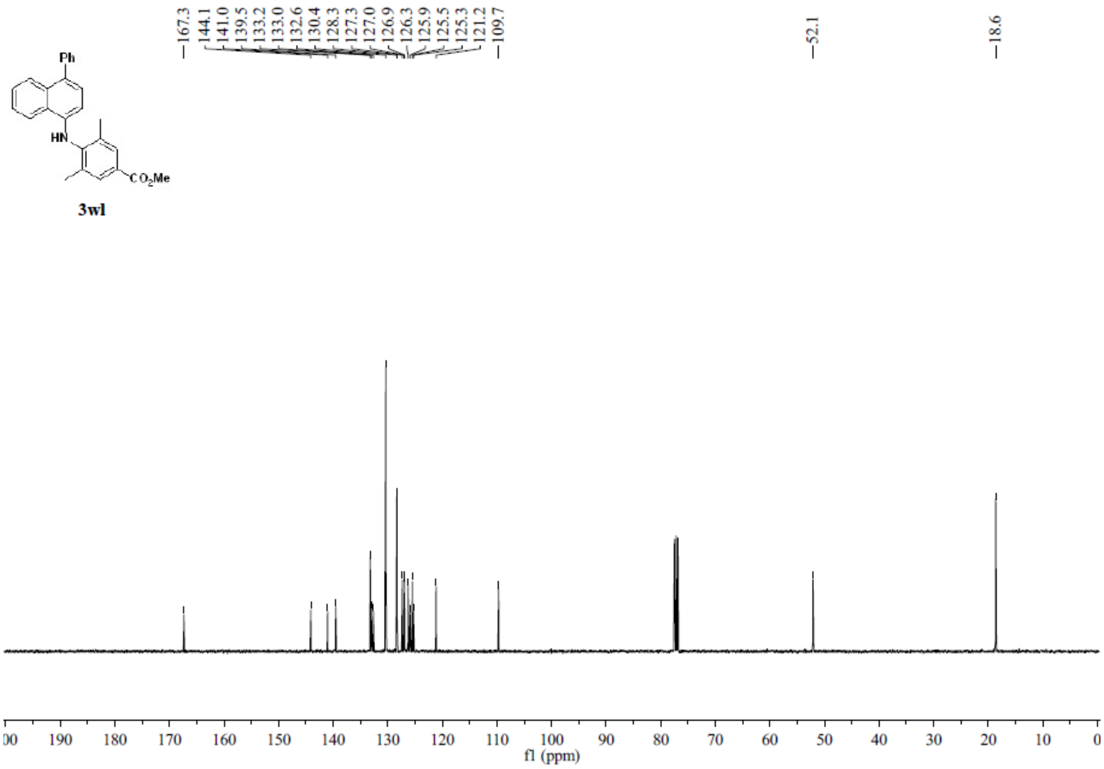

Supplementary Figure 134. ^{13}C-NMR of compound **3wl**, recorded at 100 MHz and 25 °C in $CDCl_3$.

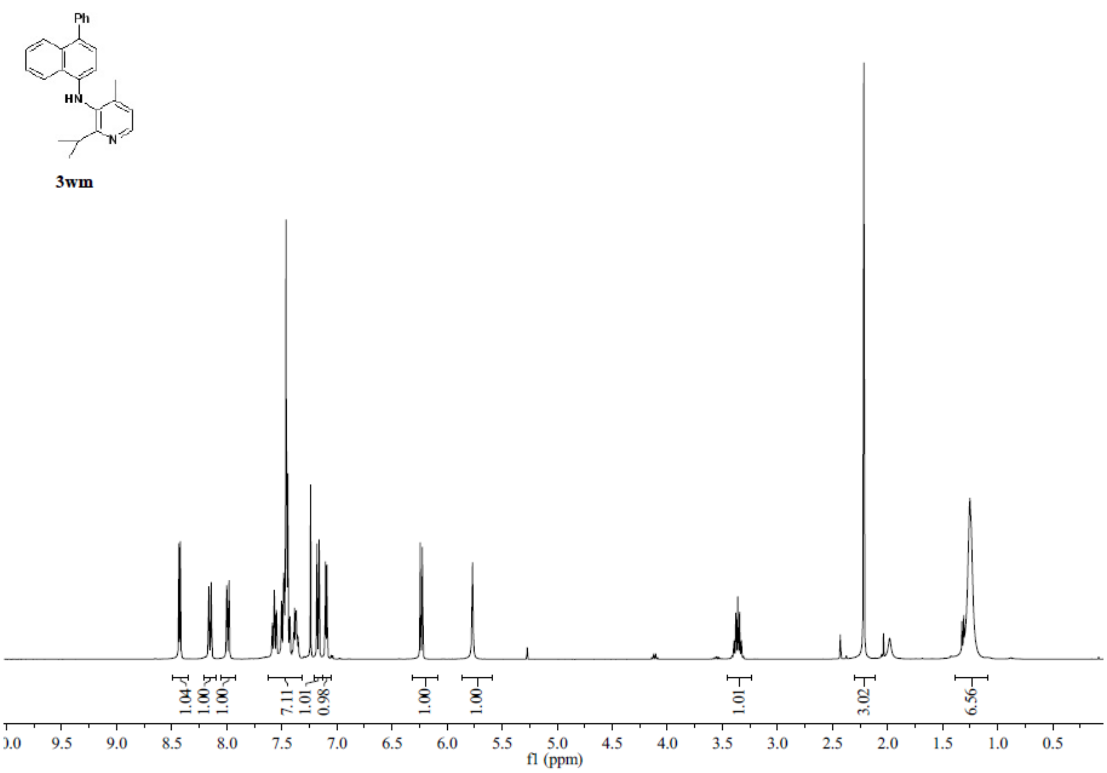

Supplementary Figure 135. ^{1}H-NMR of compound **3wm**, recorded at 400 MHz and 25 °C in $CDCl_3$.

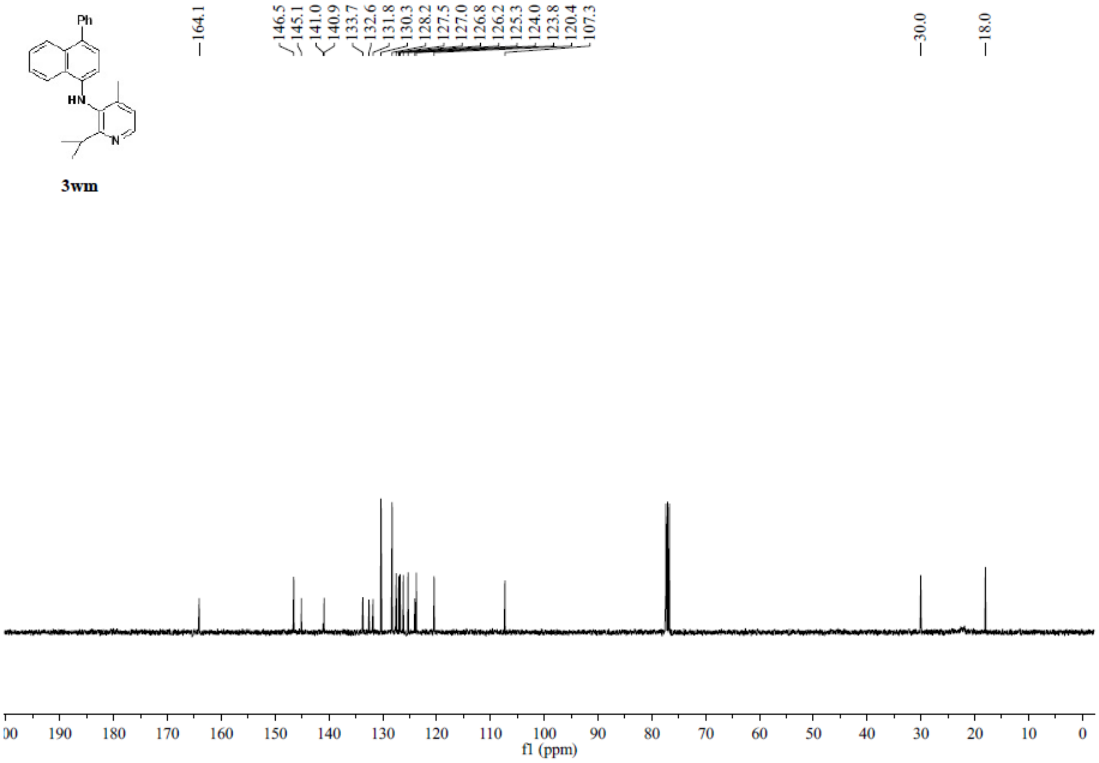

Supplementary Figure 136. ^{13}C-NMR of compound **3wm**, recorded at 100 MHz and 25 °C in $CDCl_3$.

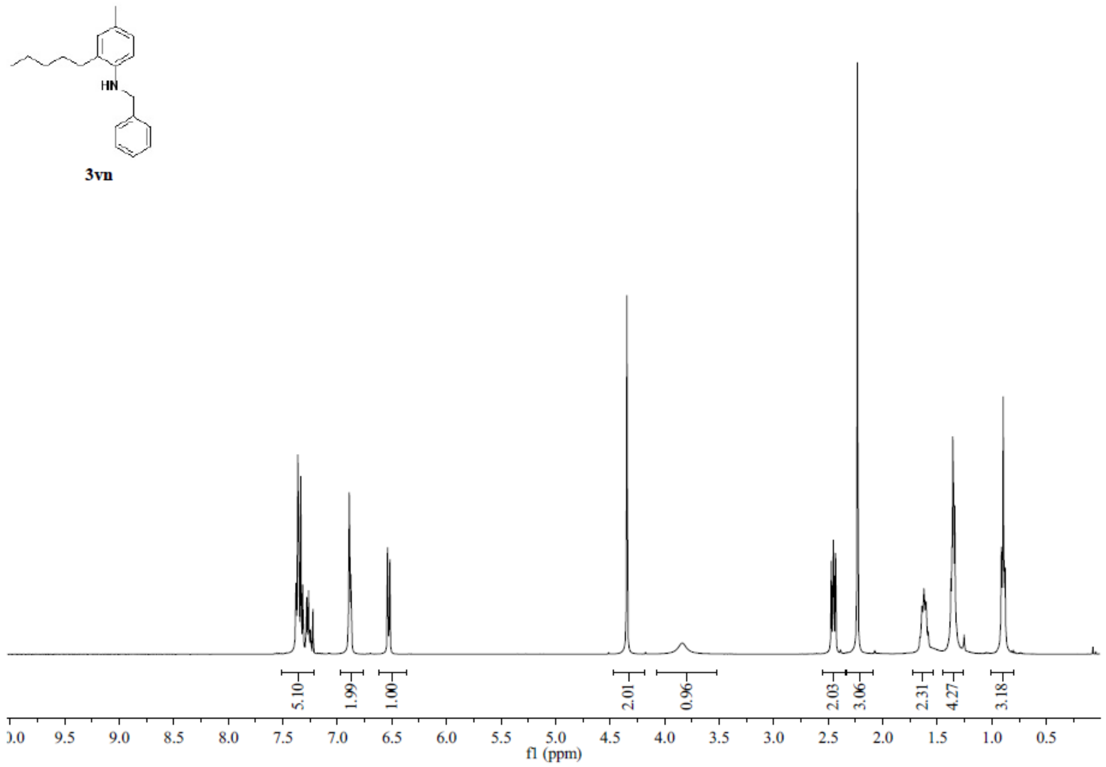

Supplementary Figure 137. ^{1}H-NMR of compound **3vn**, recorded at 400 MHz and 25 °C in $CDCl_3$.

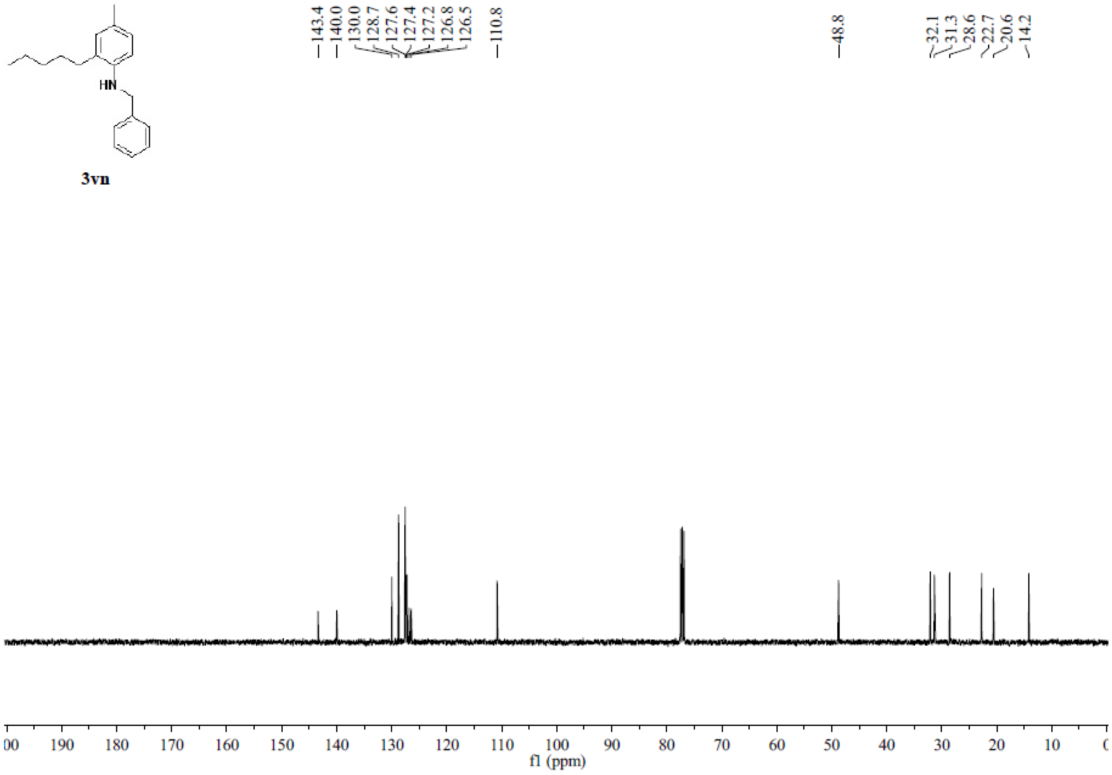

Supplementary Figure 138. ^{13}C-NMR of compound **3vn**, recorded at 100 MHz and 25 °C in $CDCl_3$.

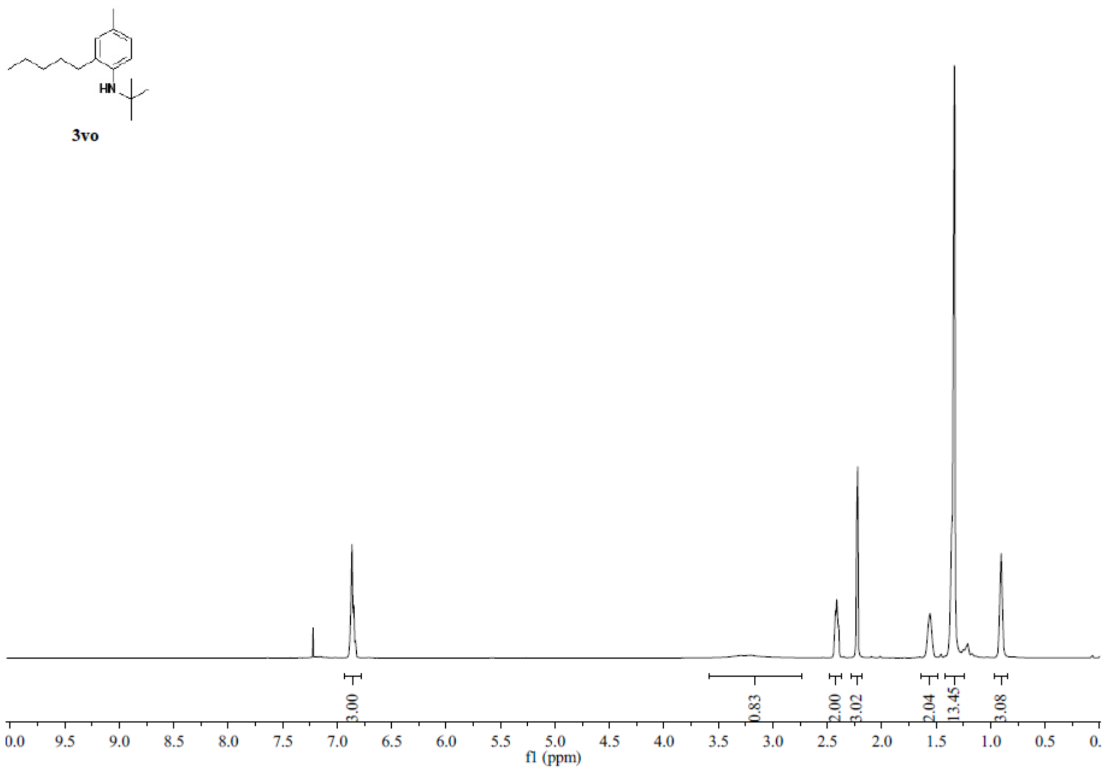

Supplementary Figure 139. ^{1}H-NMR of compound **3vo**, recorded at 500 MHz and 25 °C in $CDCl_3$.

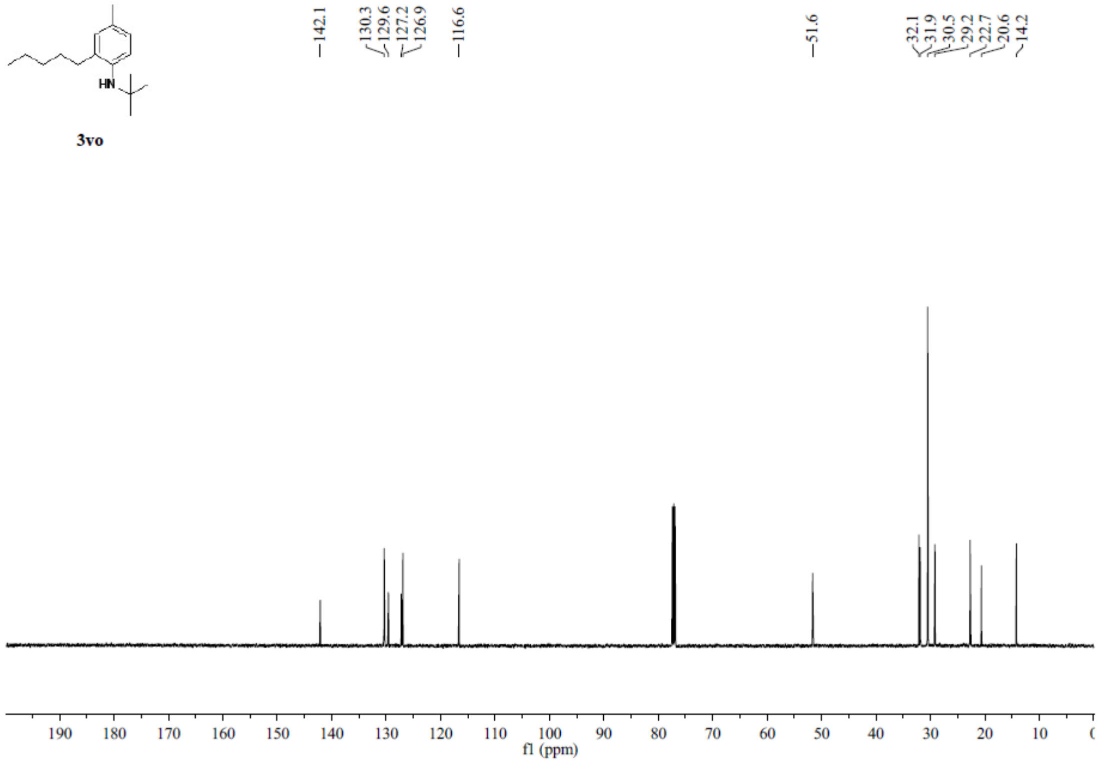

Supplementary Figure 140. ^{13}C-NMR of compound **3vo**, recorded at 125 MHz and 25 °C in $CDCl_3$.

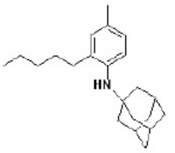

3vp

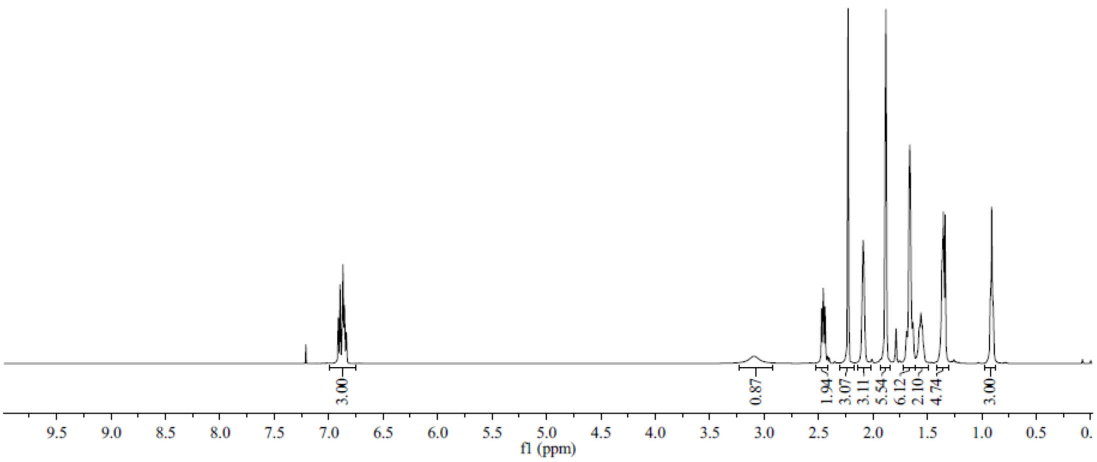

Supplementary Figure 141. ^{1}H-NMR of compound **3vp**, recorded at 500 MHz and 25 °C in $CDCl_3$.

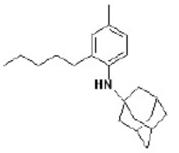

3vp

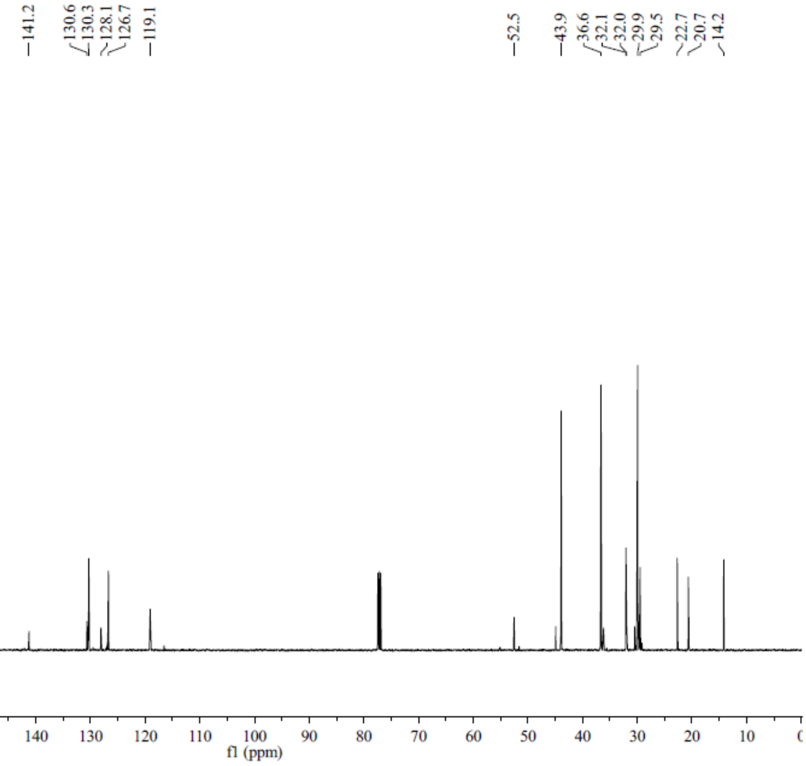

Supplementary Figure 142. ^{13}C-NMR of compound **3vp**, recorded at 125 MHz and 25 °C in $CDCl_3$.

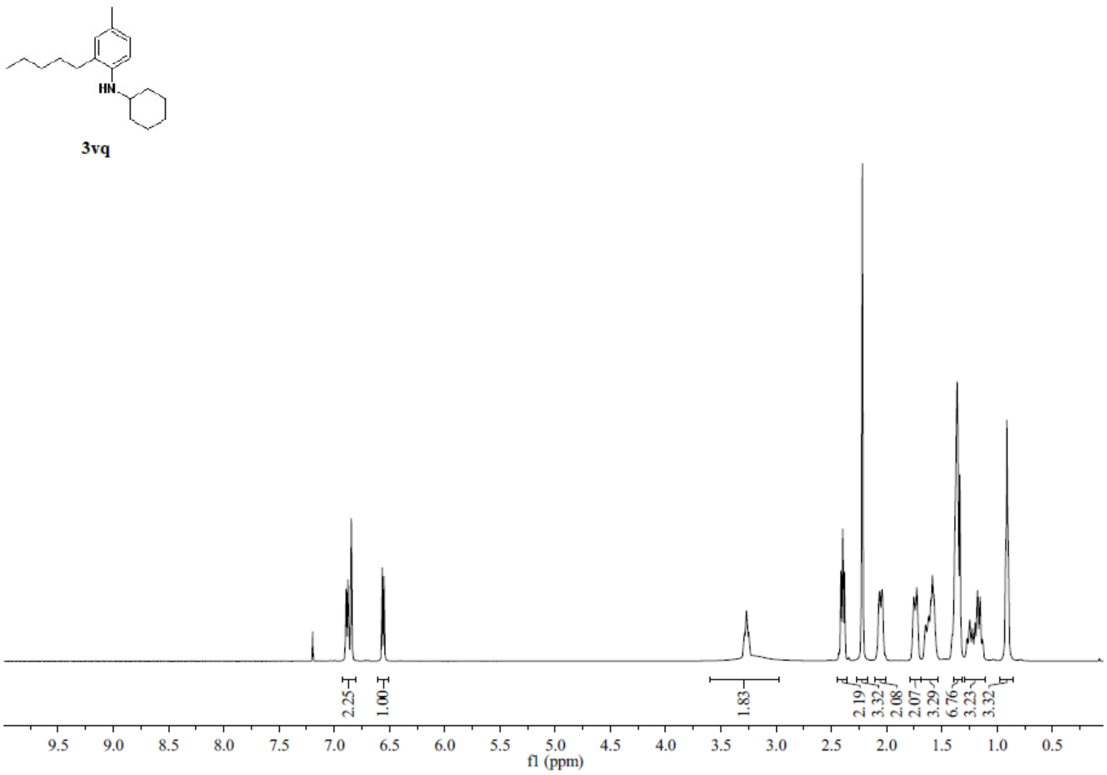

Supplementary Figure 143. ^{1}H-NMR of compound **3vq**, recorded at 500 MHz and 25 °C in $CDCl_3$.

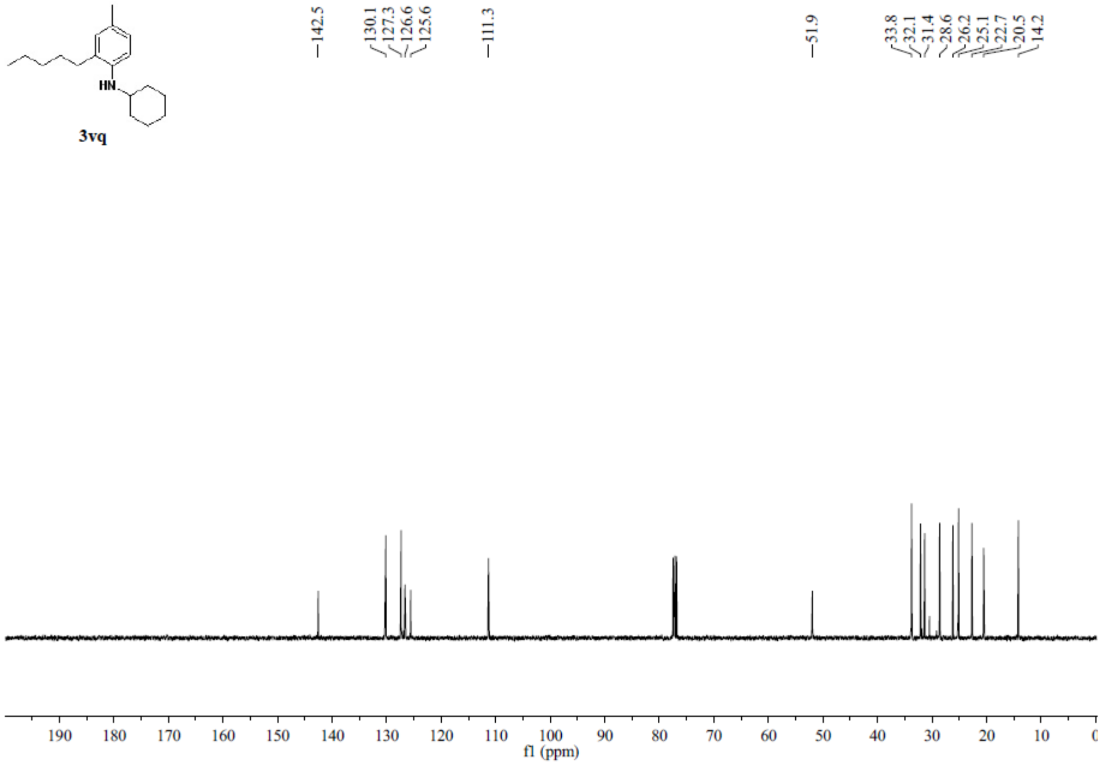

Supplementary Figure 144. ^{13}C-NMR of compound **3vq**, recorded at 100 MHz and 25 °C in $CDCl_3$.

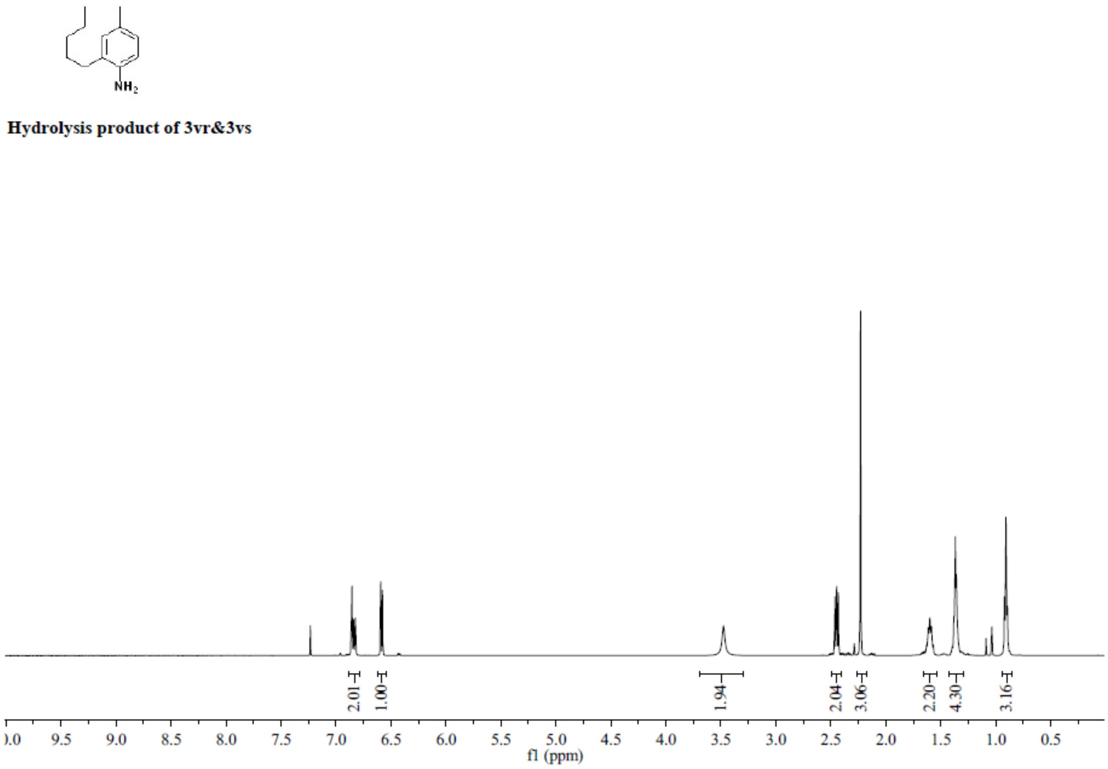

Supplementary Figure 145. ^{1}H-NMR of compound **3vr&3vs**, recorded at 500 MHz and 25 °C in $CDCl_3$.

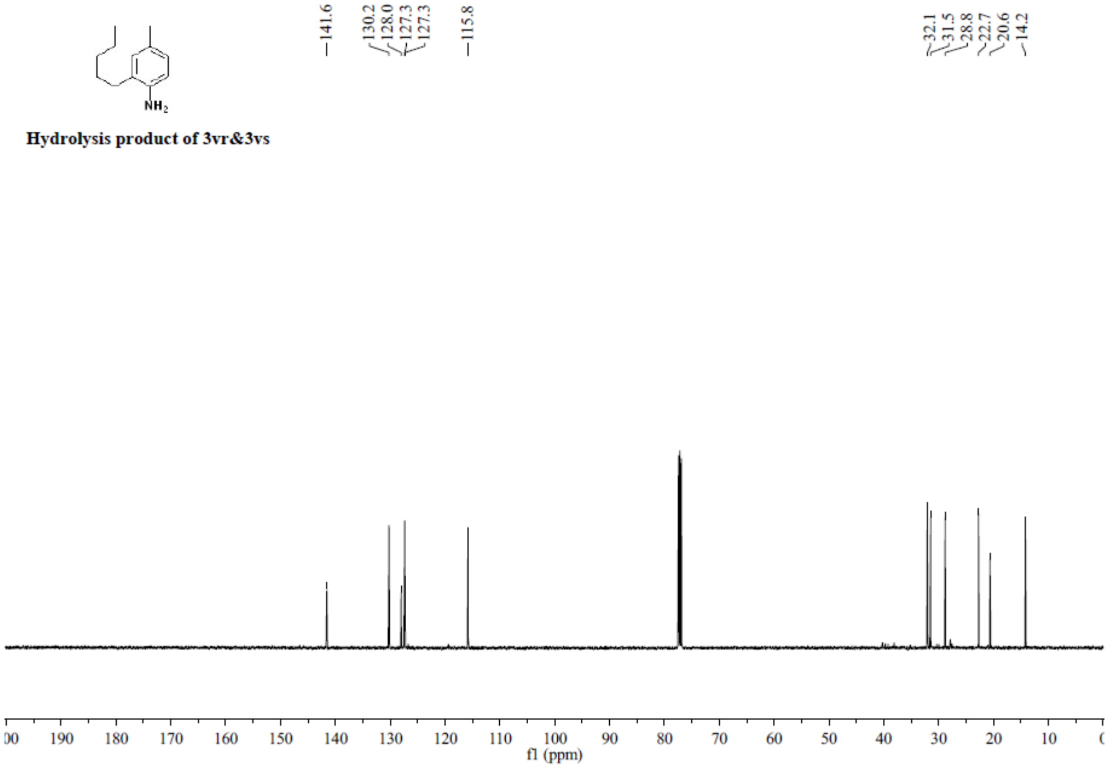

Supplementary Figure 146. ^{13}C-NMR of compound **3vr&3vs**, recorded at 125 MHz and 25 °C in $CDCl_3$.

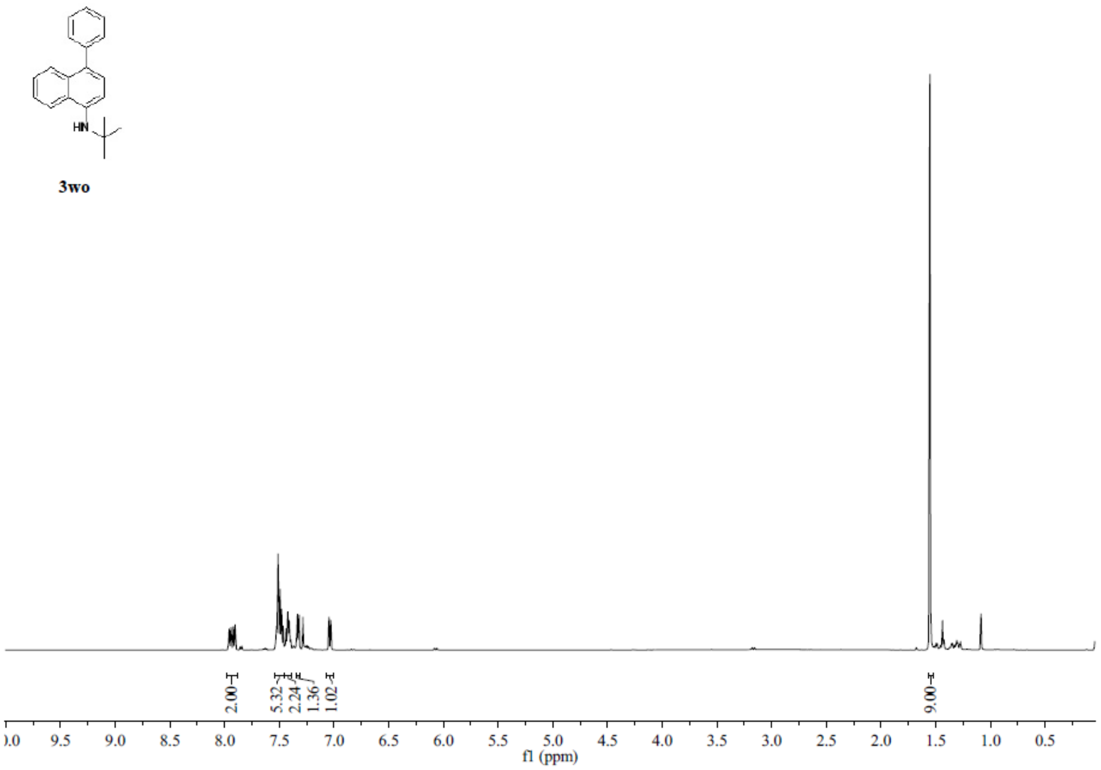

Supplementary Figure 147. ^{1}H-NMR of compound **3wo**, recorded at 500 MHz and 25 °C in $CDCl_3$.

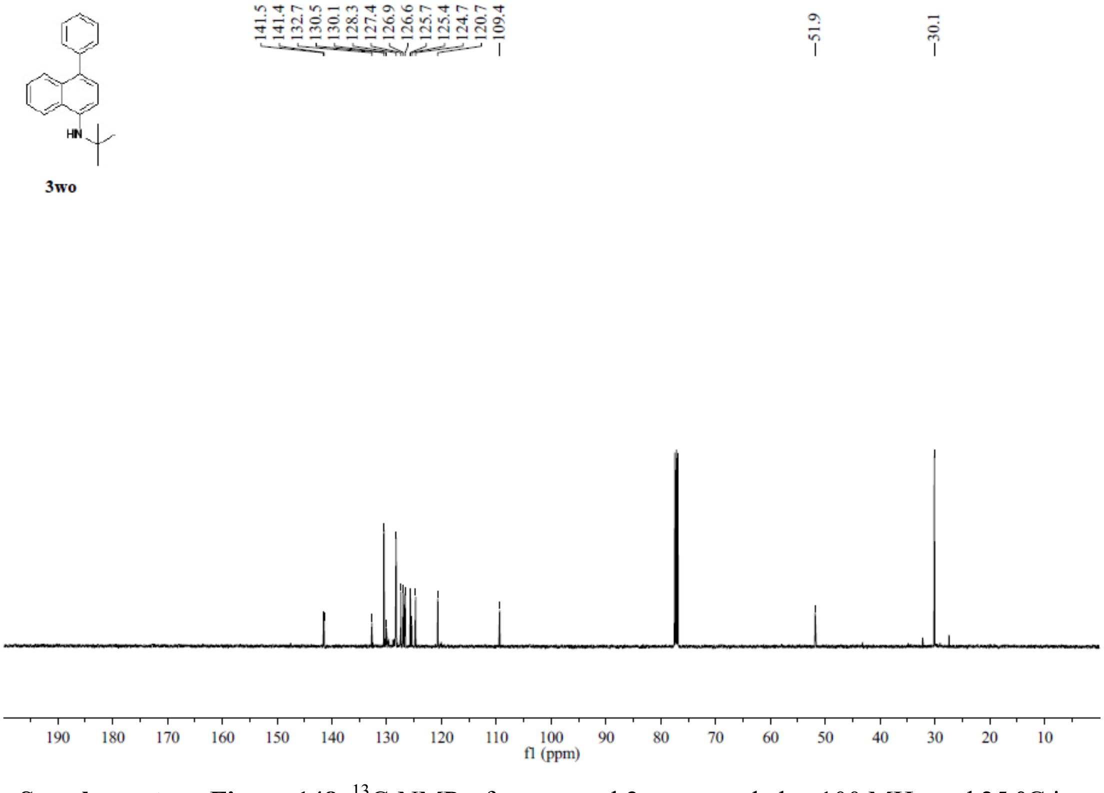

Supplementary Figure 148. ^{13}C-NMR of compound **3wo**, recorded at 100 MHz and 25 °C in $CDCl_3$.

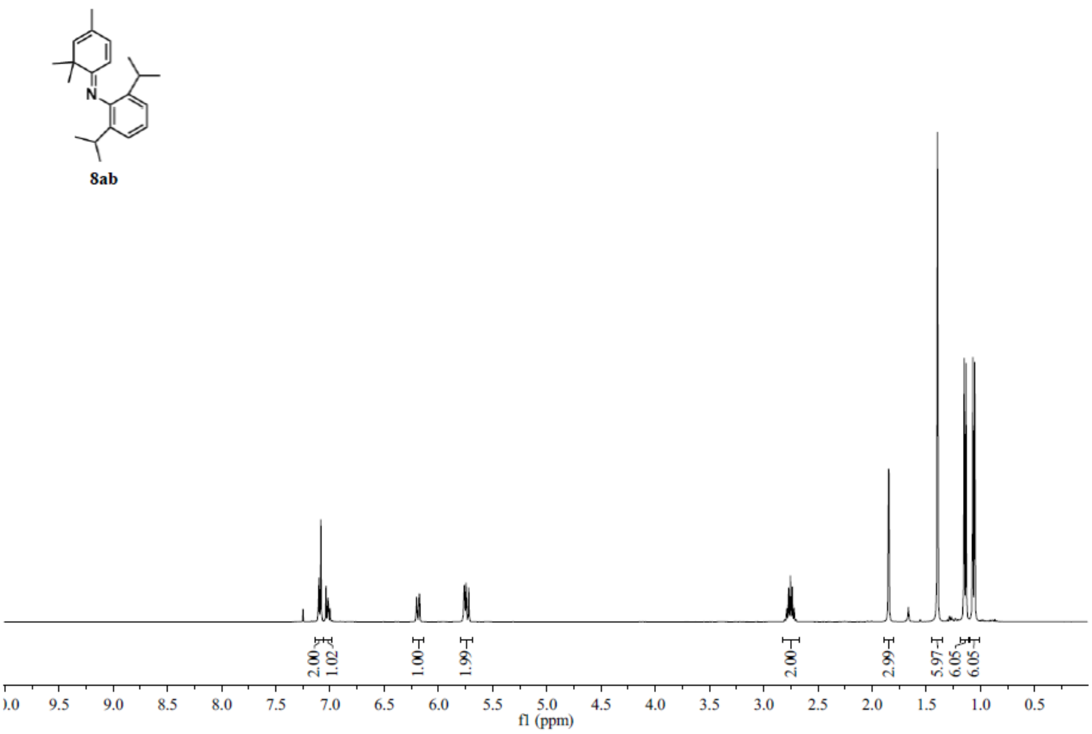

Supplementary Figure 149. ^{1}H-NMR of compound **8ab**, recorded at 400 MHz and 25 °C in $CDCl_3$.

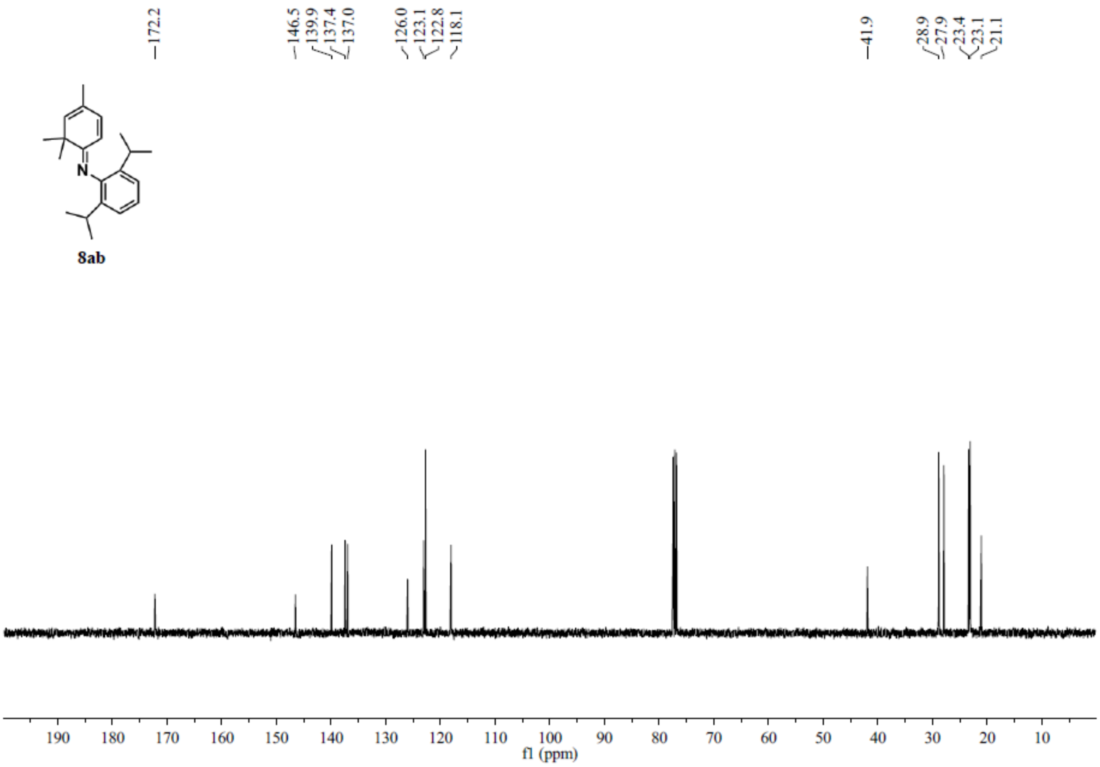

Supplementary Figure 150. ^{13}C-NMR of compound **8ab**, recorded at 100 MHz and 25 °C in $CDCl_3$.

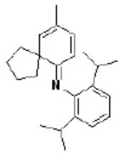

8bb

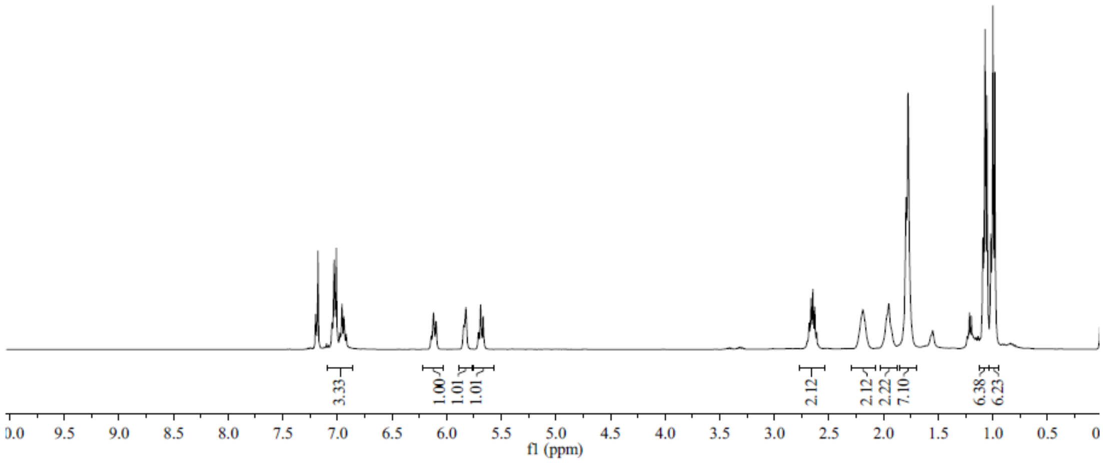

Supplementary Figure 151. ^{1}H-NMR of compound **8bb**, recorded at 400 MHz and 25 °C in $CDCl_3$.

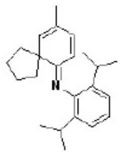

8bb

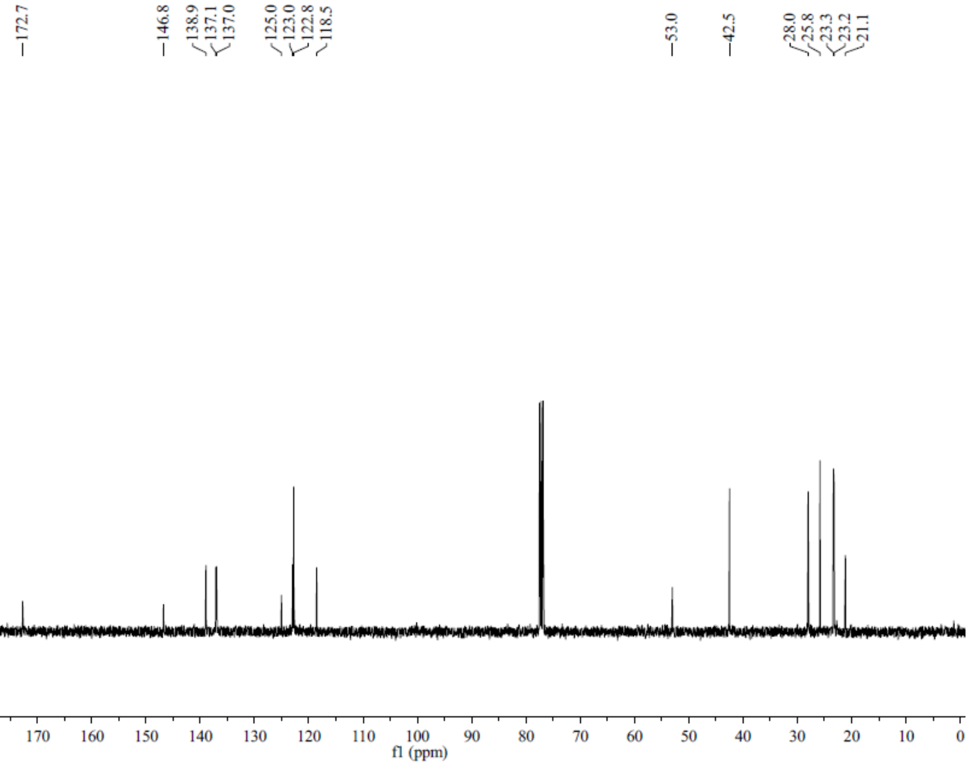

Supplementary Figure 152. ^{13}C-NMR of compound **8bb**, recorded at 100 MHz and 25 °C in $CDCl_3$.

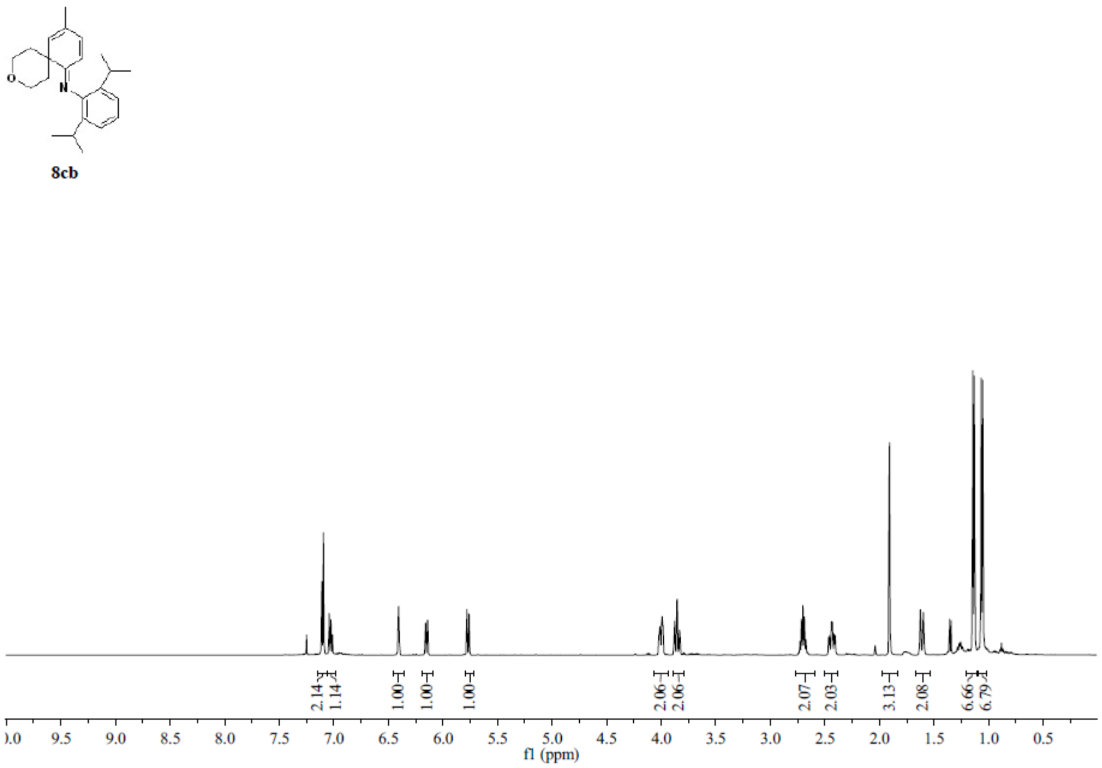

Supplementary Figure 153. ^{1}H-NMR of compound **8cb**, recorded at 500 MHz and 25 °C in $CDCl_3$.

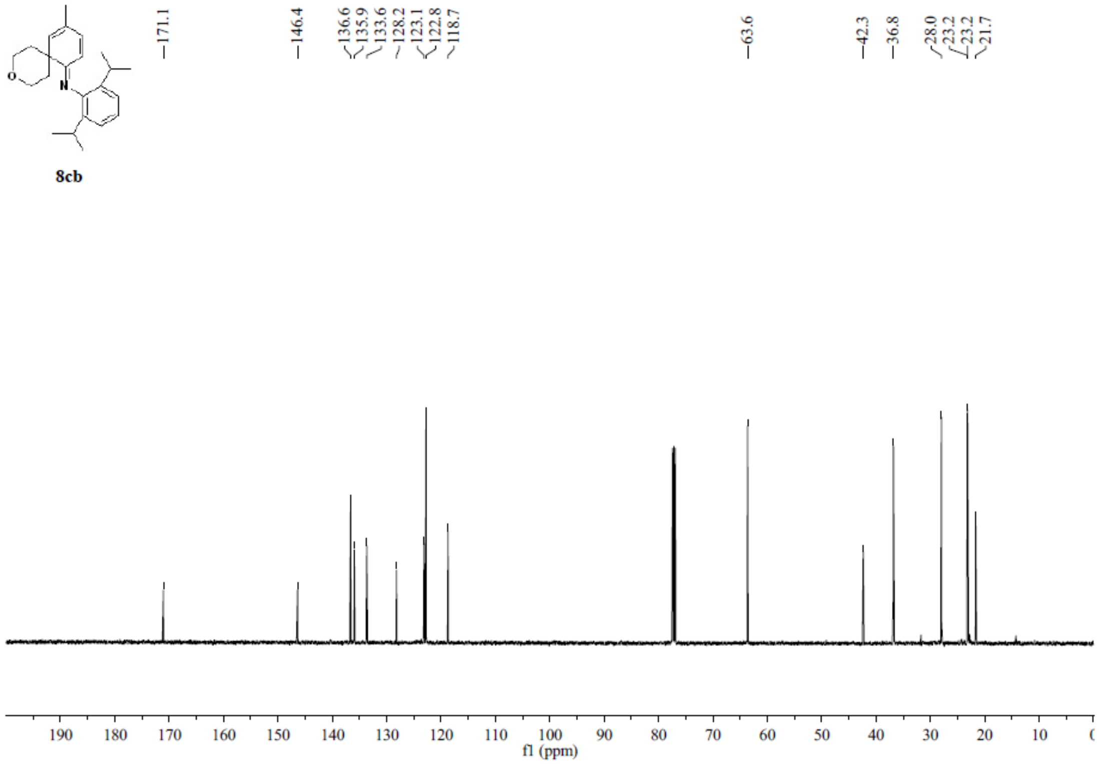

Supplementary Figure 154. ^{13}C-NMR of compound **8cb**, recorded at 125 MHz and 25 °C in $CDCl_3$.

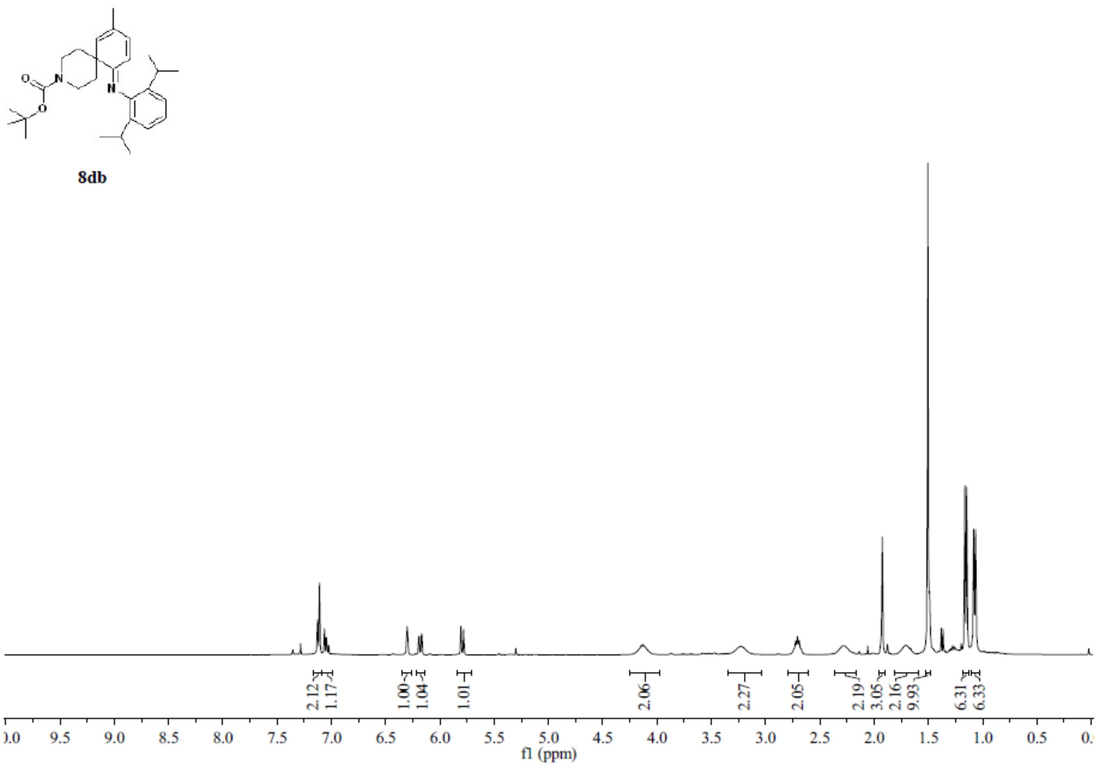

Supplementary Figure 155. ^{1}H-NMR of compound **8db**, recorded at 400 MHz and 25 °C in $CDCl_3$.

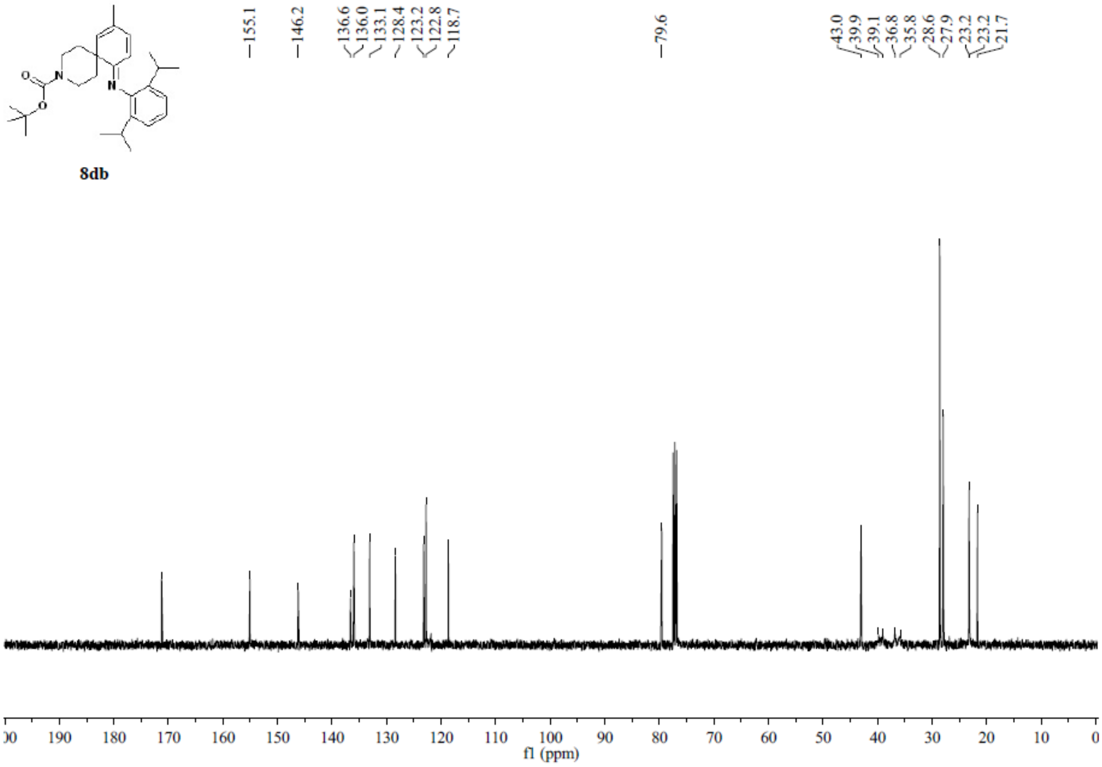

Supplementary Figure 156. ^{13}C-NMR of compound **8db**, recorded at 100 MHz and 25 °C in $CDCl_3$.

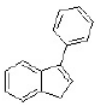

5w

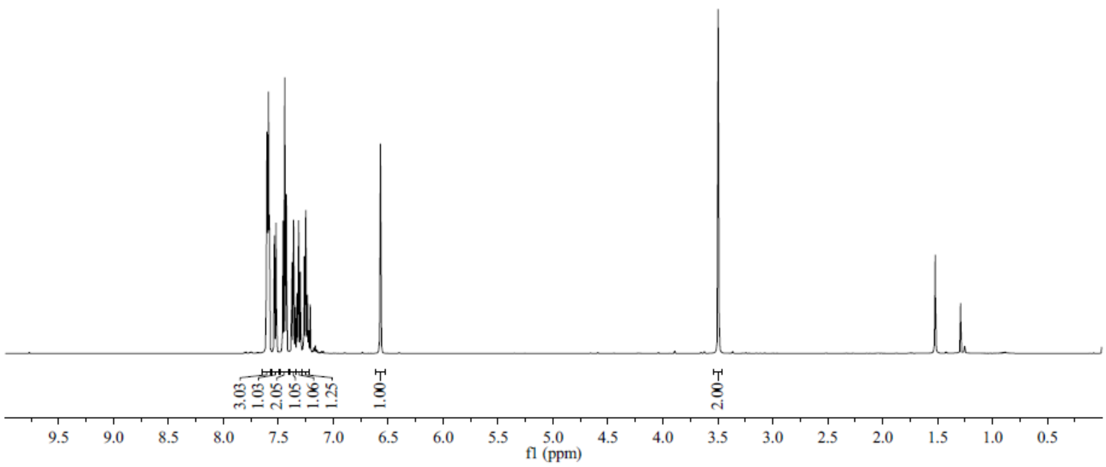

Supplementary Figure 157. ^{1}H-NMR of compound **5w**, recorded at 500 MHz and 25 °C in $CDCl_3$.

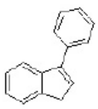

5w

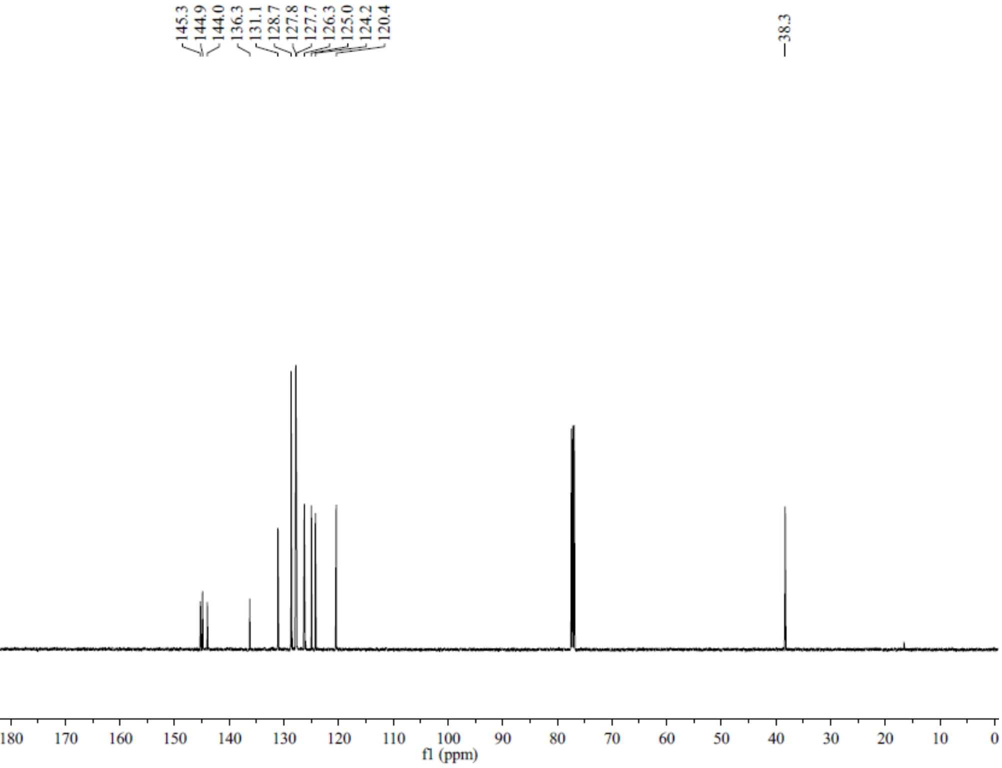

Supplementary Figure 158. ^{13}C-NMR of compound **5w**, recorded at 125 MHz and 25 °C in $CDCl_3$.

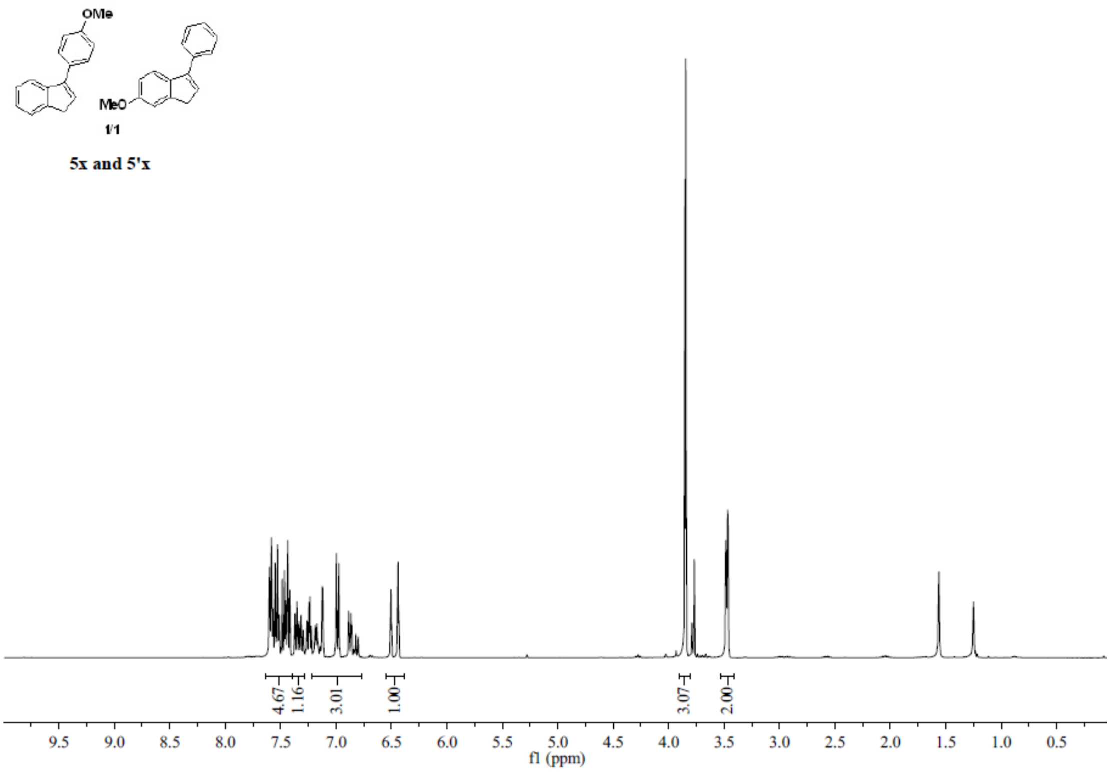

Supplementary Figure 159. ^{1}H-NMR of compound **5x** and **5’x**, recorded at 400 MHz and 25 °C in $CDCl_3$.

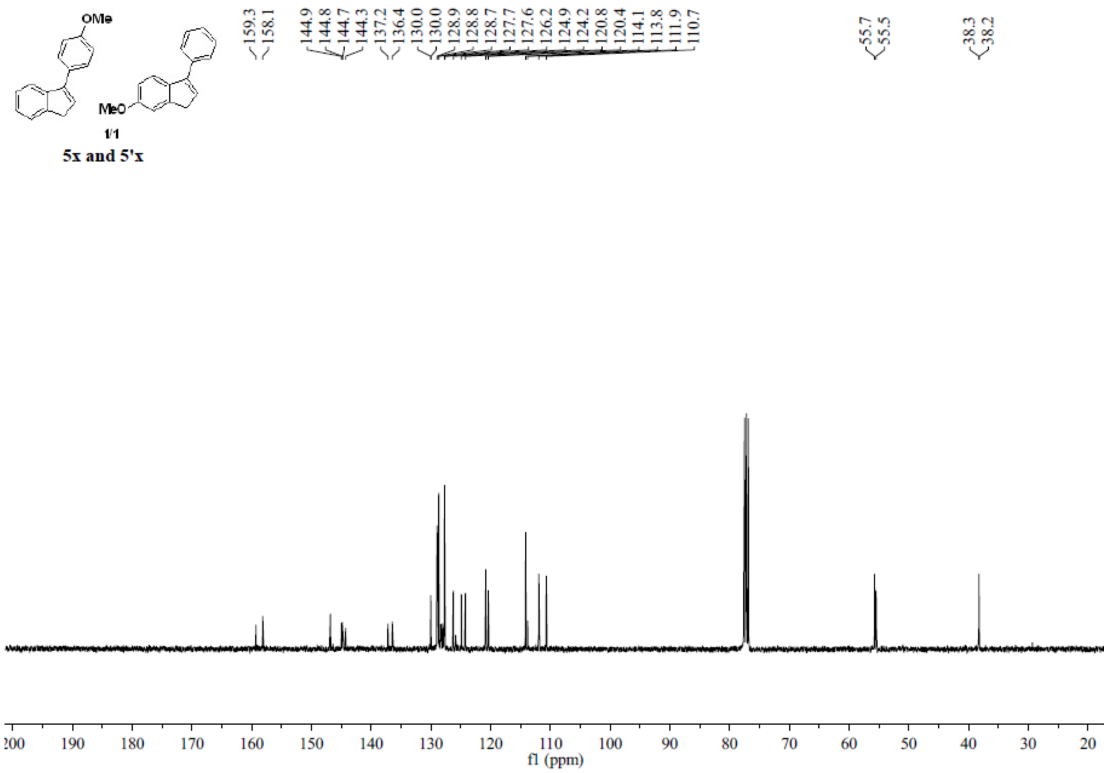

Supplementary Figure 160. ^{13}C-NMR of compound **5x** and **5’x**, recorded at 100 MHz and 25 °C in $CDCl_3$.

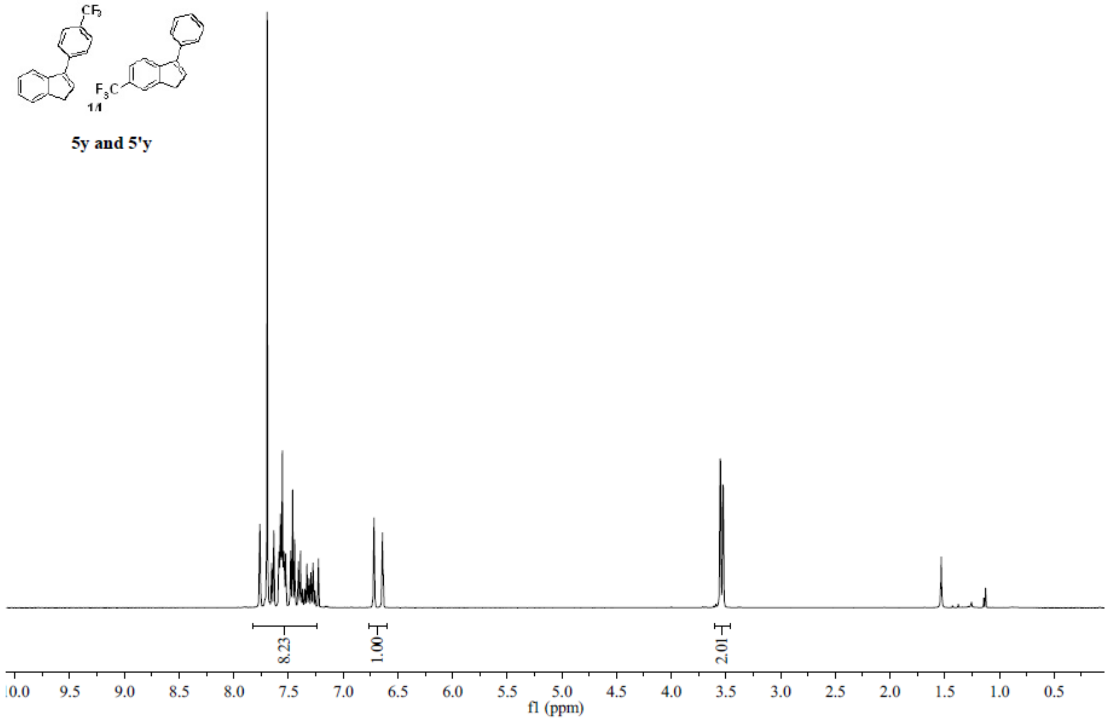

Supplementary Figure 161. ^{1}H-NMR of compound **5y** and **5’y**, recorded at 400 MHz and 25 °C in $CDCl_3$.

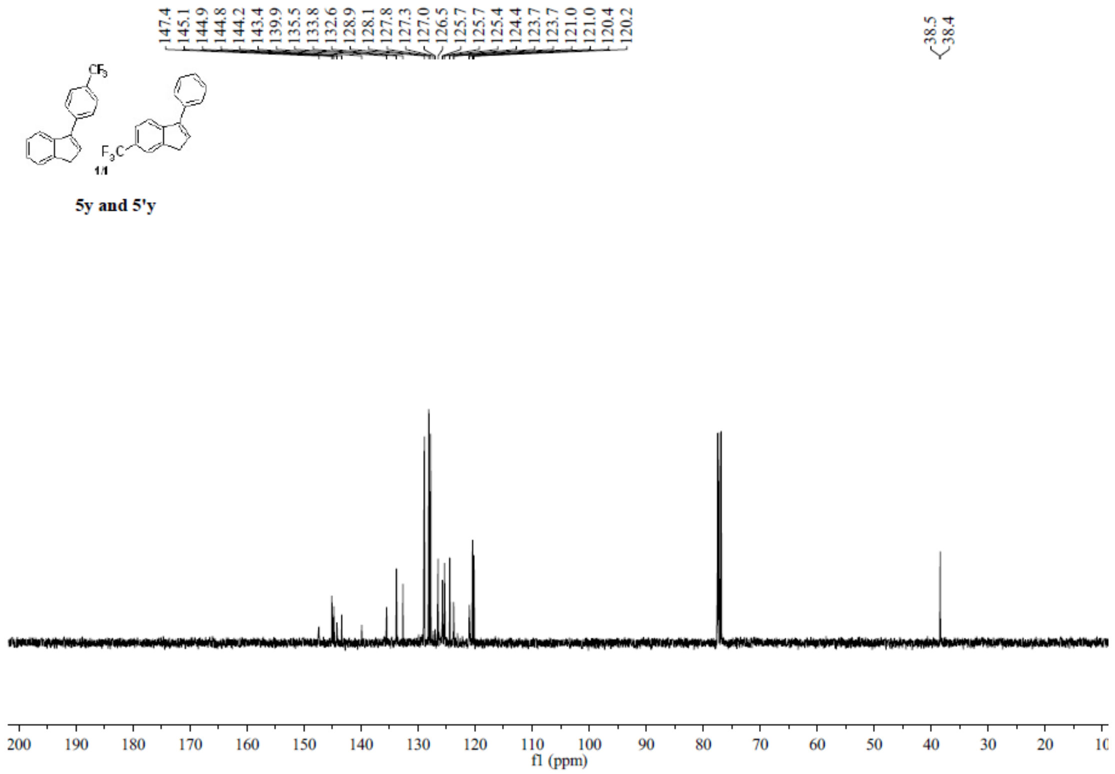

Supplementary Figure 162. ^{13}C-NMR of compound **5y** and **5’y**, recorded at 100 MHz and 25 °C in $CDCl_3$.

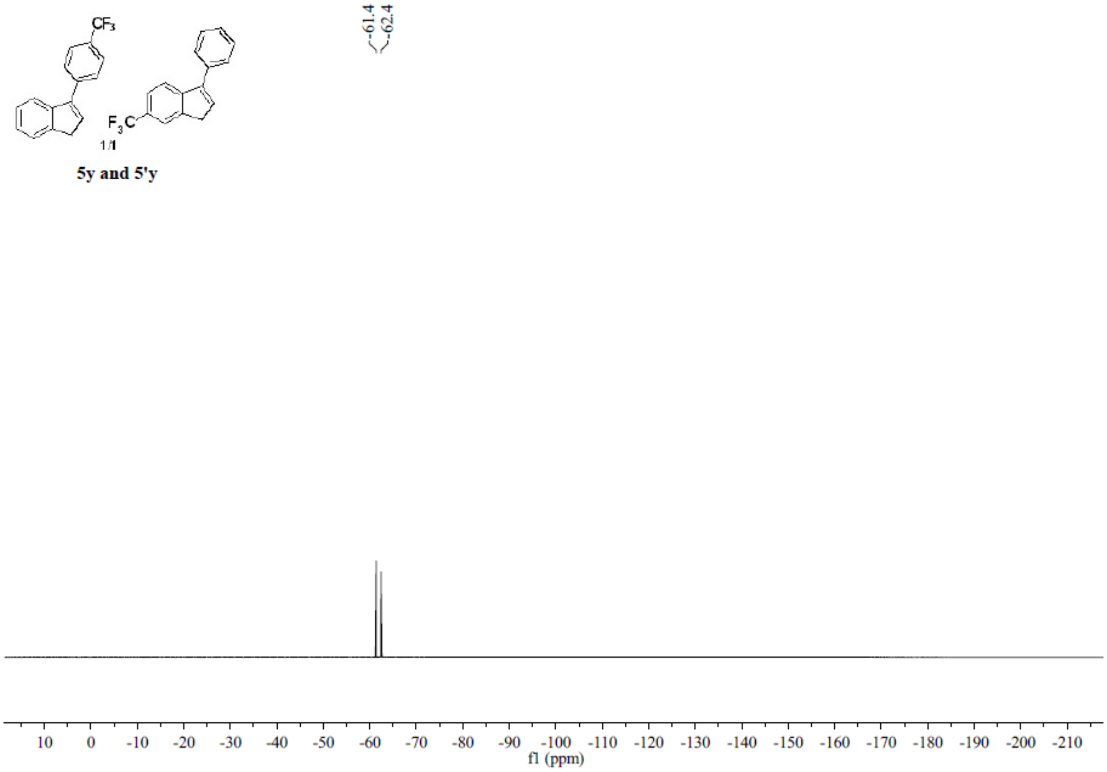

Supplementary Figure 163. ^{19}F-NMR of compound **5y** and **5'y**, recorded at 376 MHz and 25 °C in $CDCl_3$

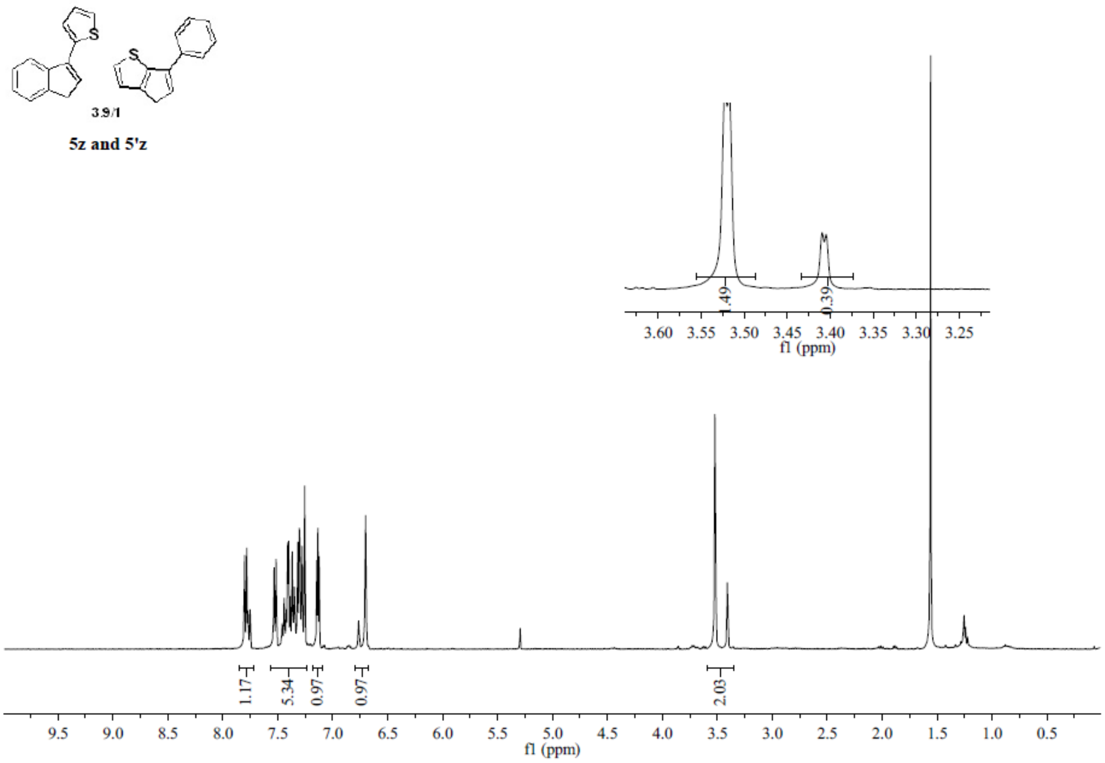

Supplementary Figure 164. ^{1}H-NMR of compound **5z** and **5'z**, recorded at 400 MHz and 25 °C in $CDCl_3$.

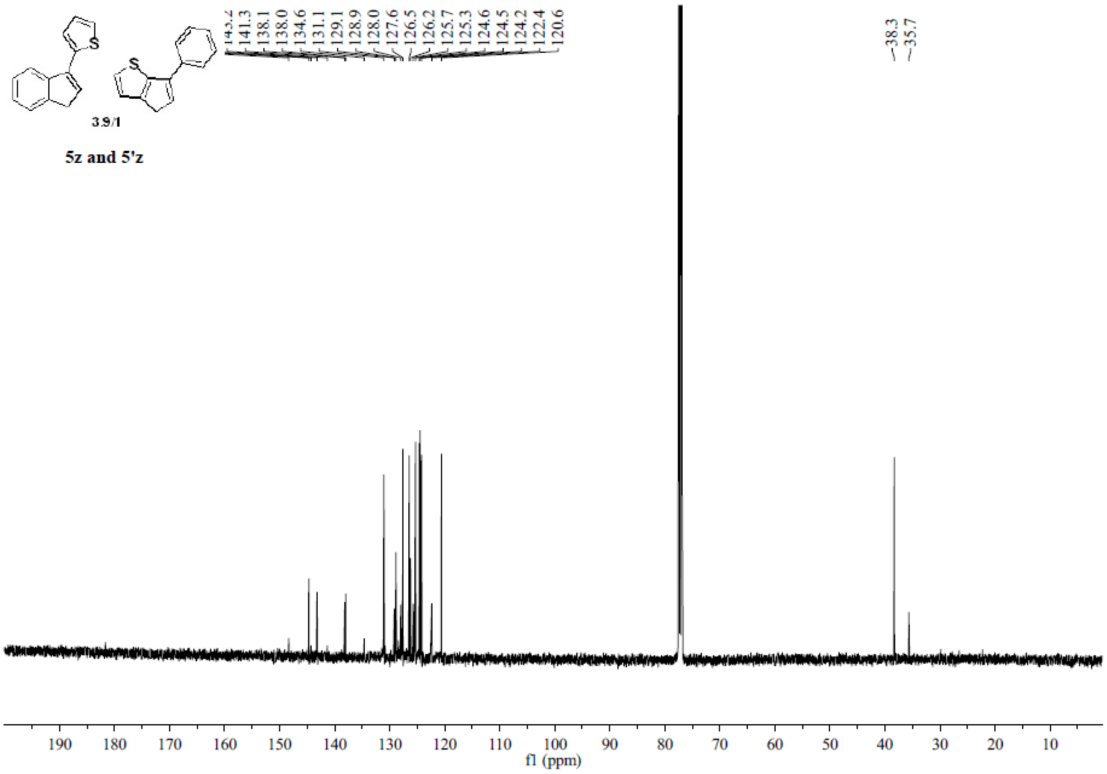

Supplementary Figure 165. ^{13}C-NMR of compound **5z** and **5'z**, recorded at 100 MHz and 25 °C in $CDCl_3$.

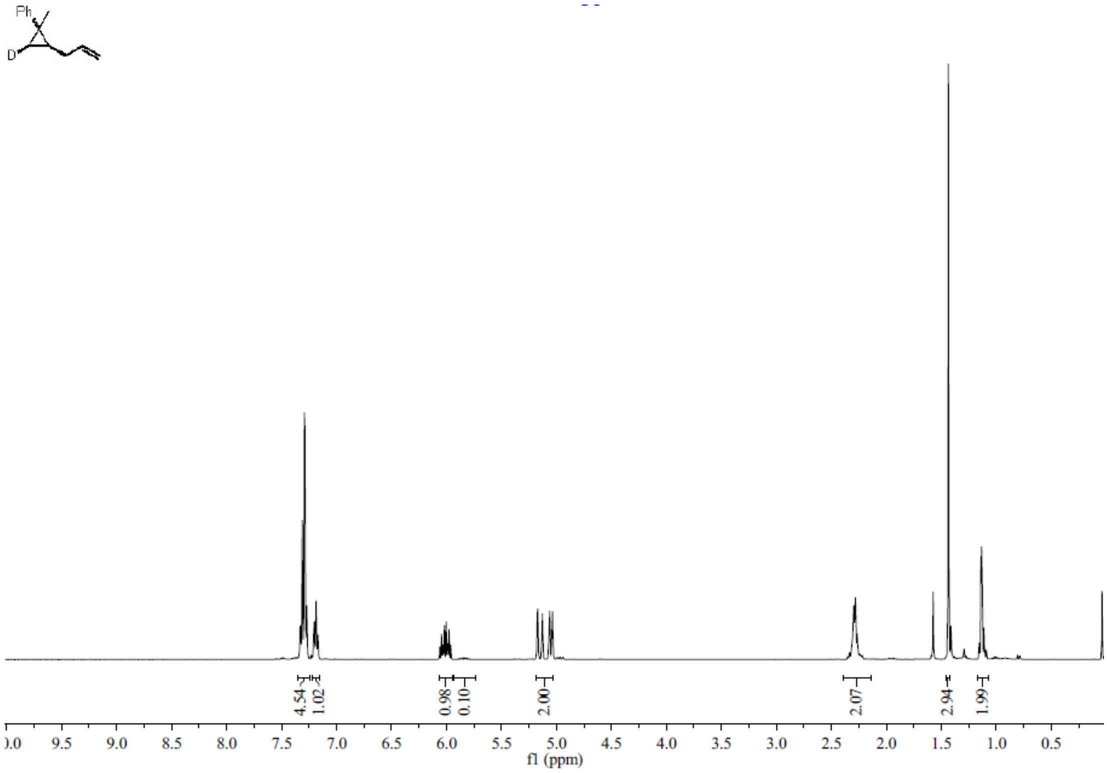

Supplementary Figure 166. ^{1}H-NMR of compound D-labelled allylcyclopropane, recorded at 400 MHz and 25 °C in $CDCl_3$.

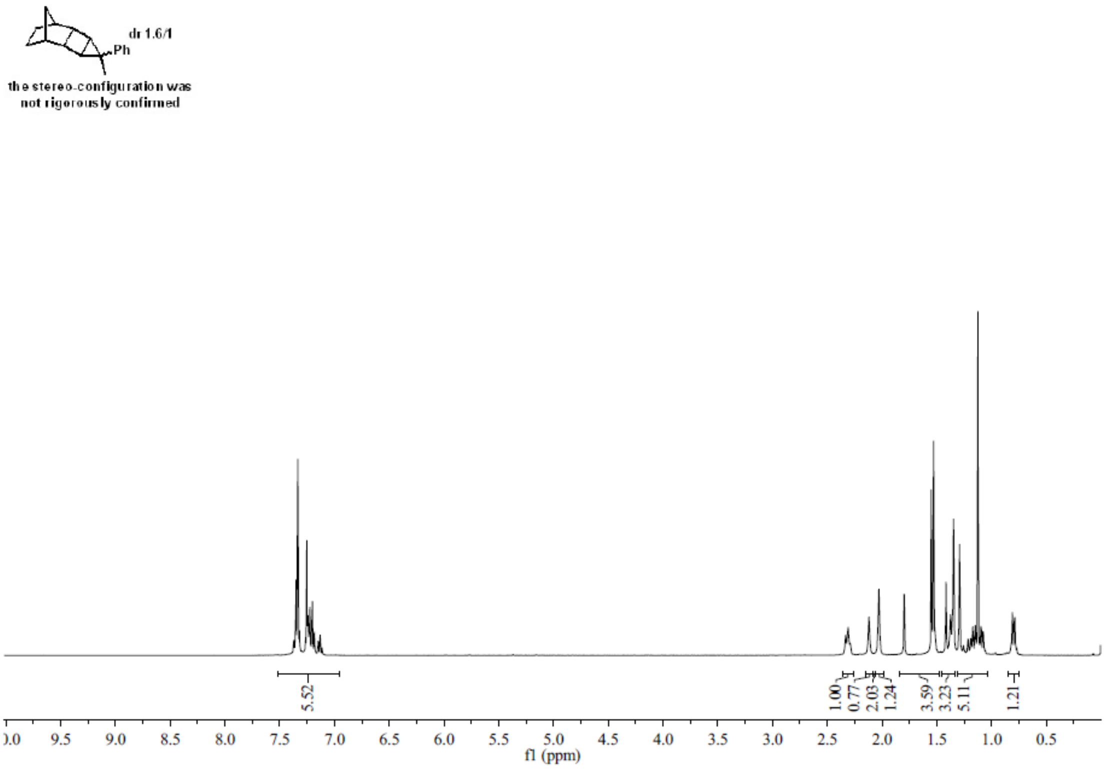

Supplementary Figure 167. ^{1}H-NMR of compound [2+2] product, recorded at 400 MHz and 25 °C in $CDCl_3$.

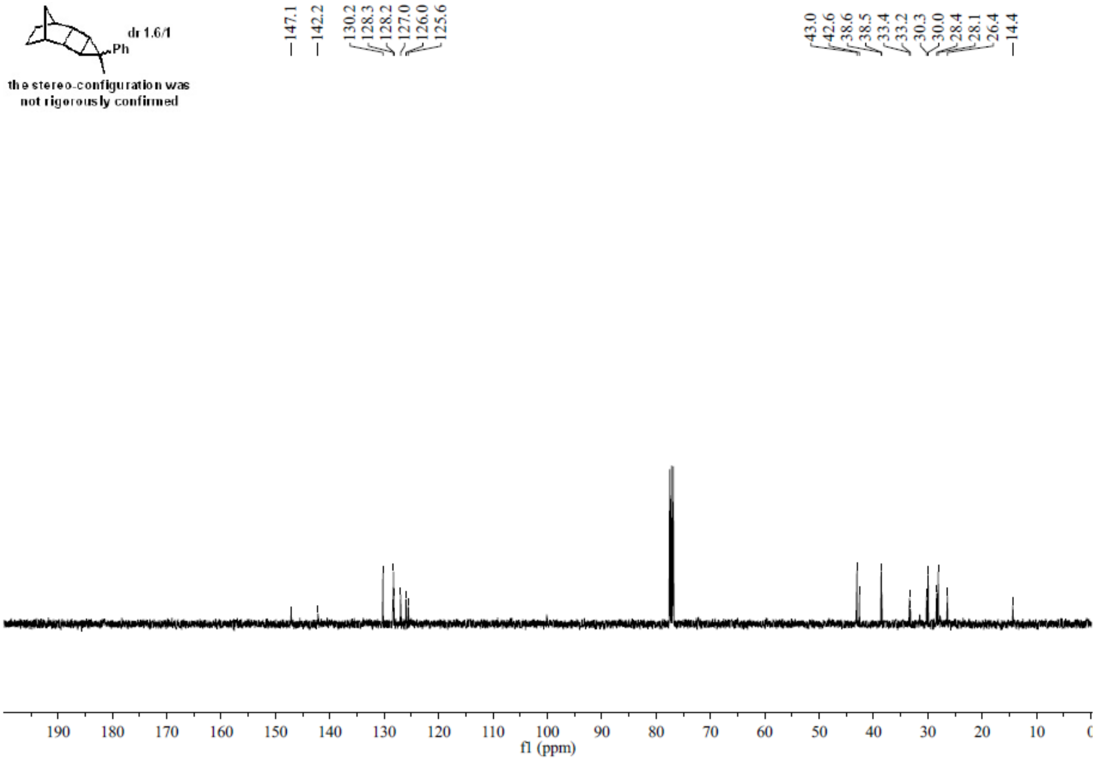

Supplementary Figure 168. ^{13}C-NMR of compound [2+2] product, recorded at 100 MHz and 25 °C in $CDCl_3$.

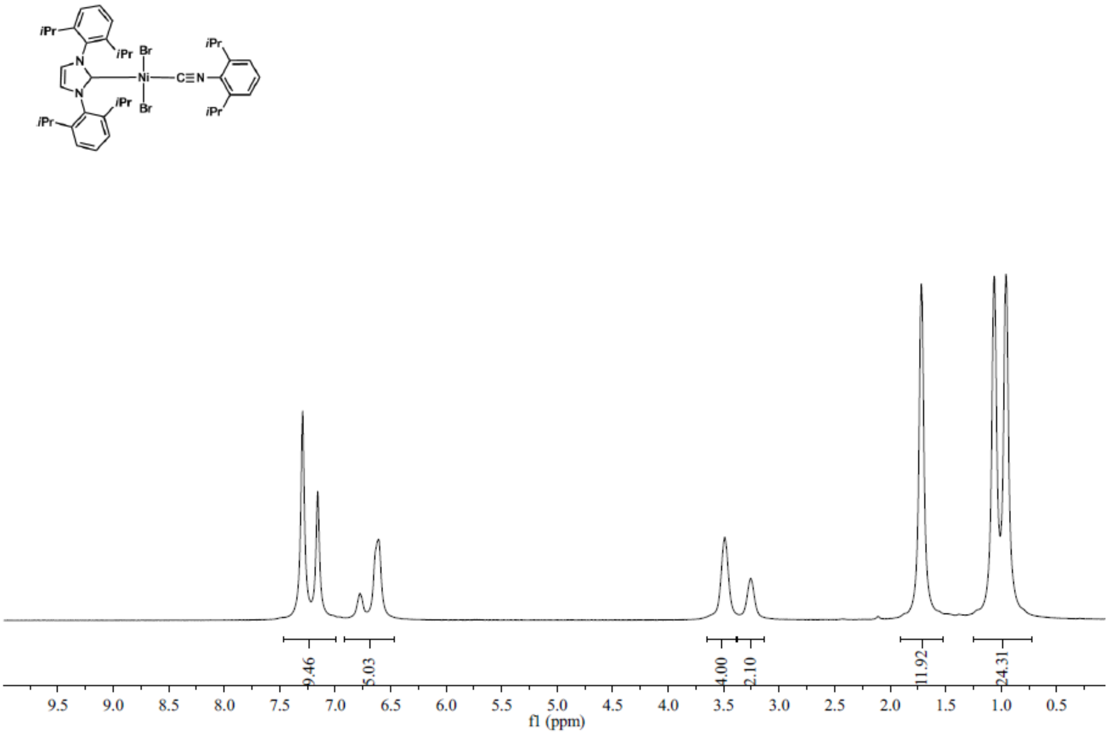

Supplementary Figure 169. ¹H-NMR of complex CCDC 2036409, recorded at 400 MHz and 25 °C in C_6D_6.

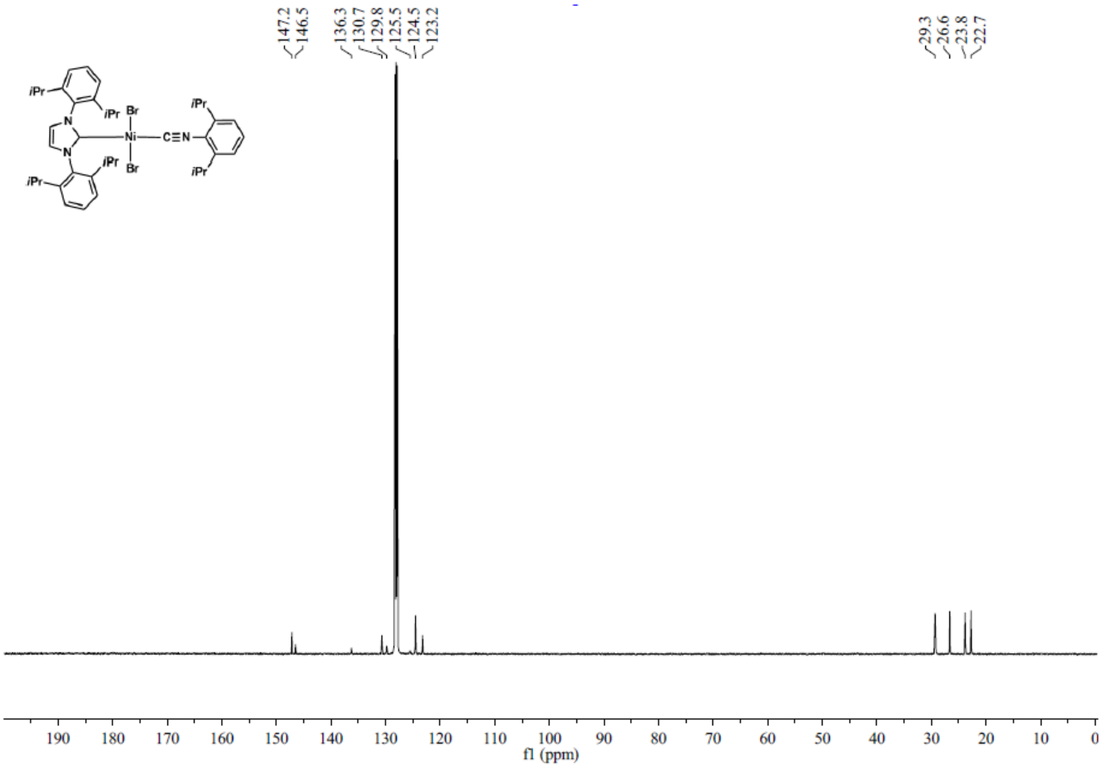

Supplementary Figure 170. ¹³C-NMR of complex CCDC 2036409, recorded at 100 MHz and 25 °C in C_6D_6.

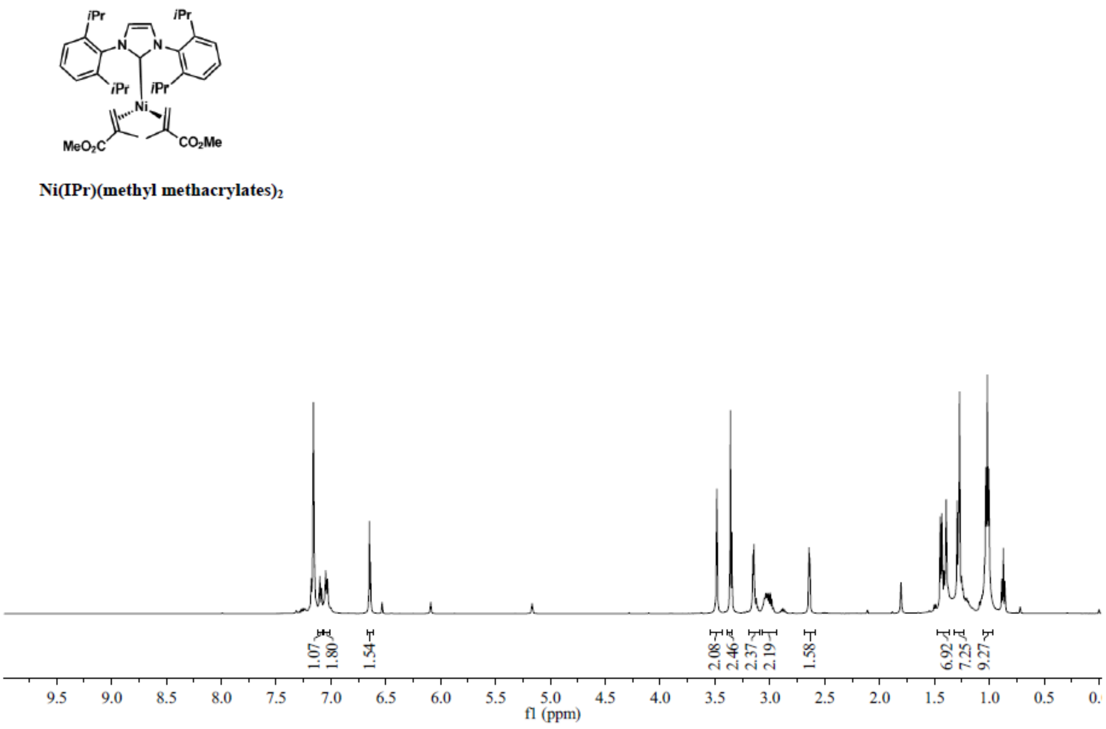

Supplementary Figure 171. ^{1}H-NMR of complex **Ni(IPr)(methyl methacrylates)$_2$**, recorded at 500 MHz and 25 °C in C_6D_6.

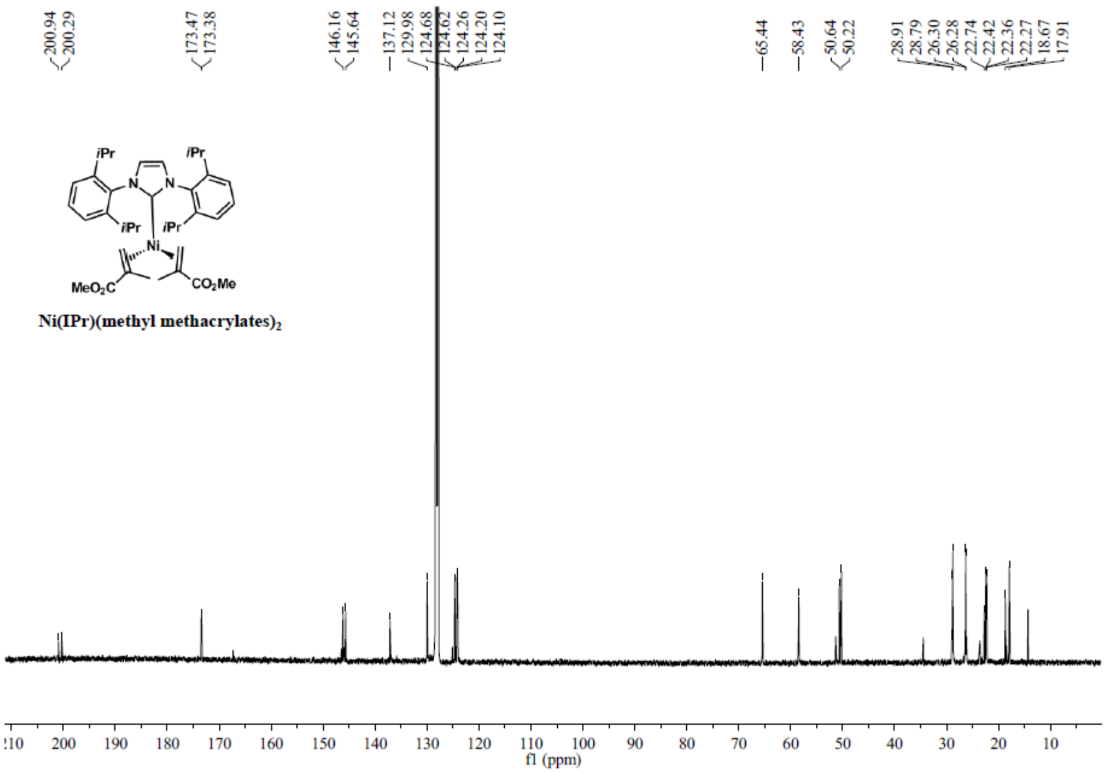

Supplementary Figure 172. ^{13}C-NMR of complex **Ni(IPr)(methyl methacrylates)$_2$**, recorded at 125 MHz and 25 °C in C_6D_6.

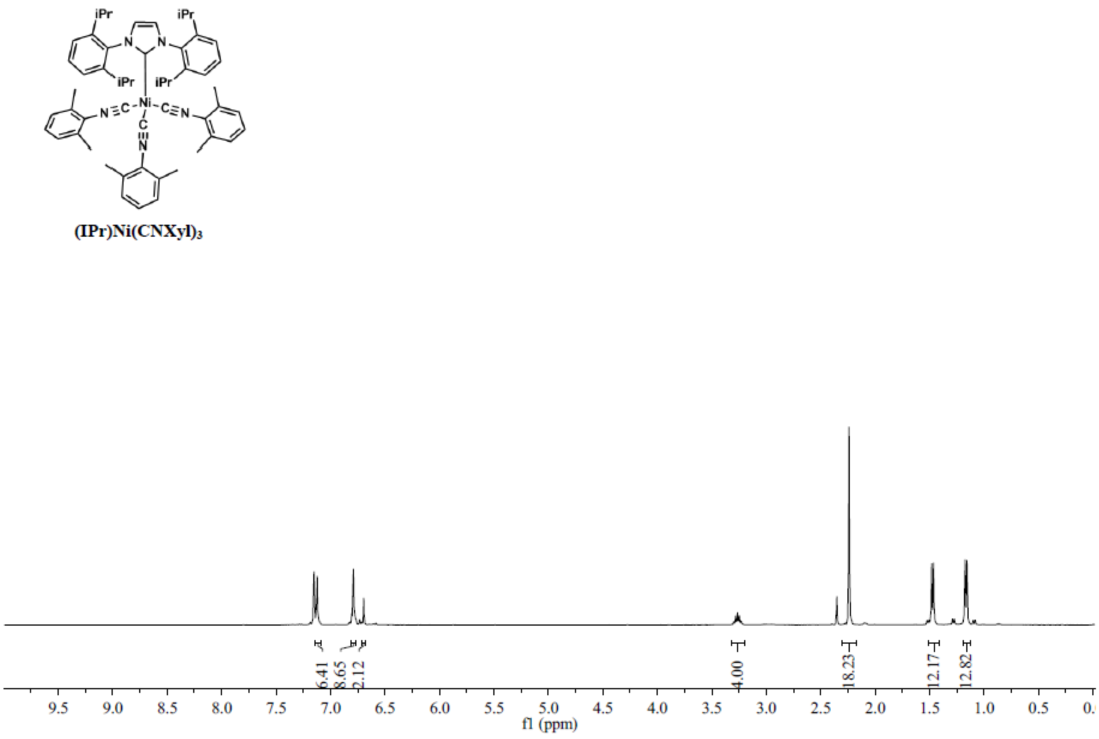

Supplementary Figure 173. ^{1}H-NMR of complex **(IPr)Ni(CNXyl)$_3$**, recorded at 400 MHz and 25 °C in C_6D_6.

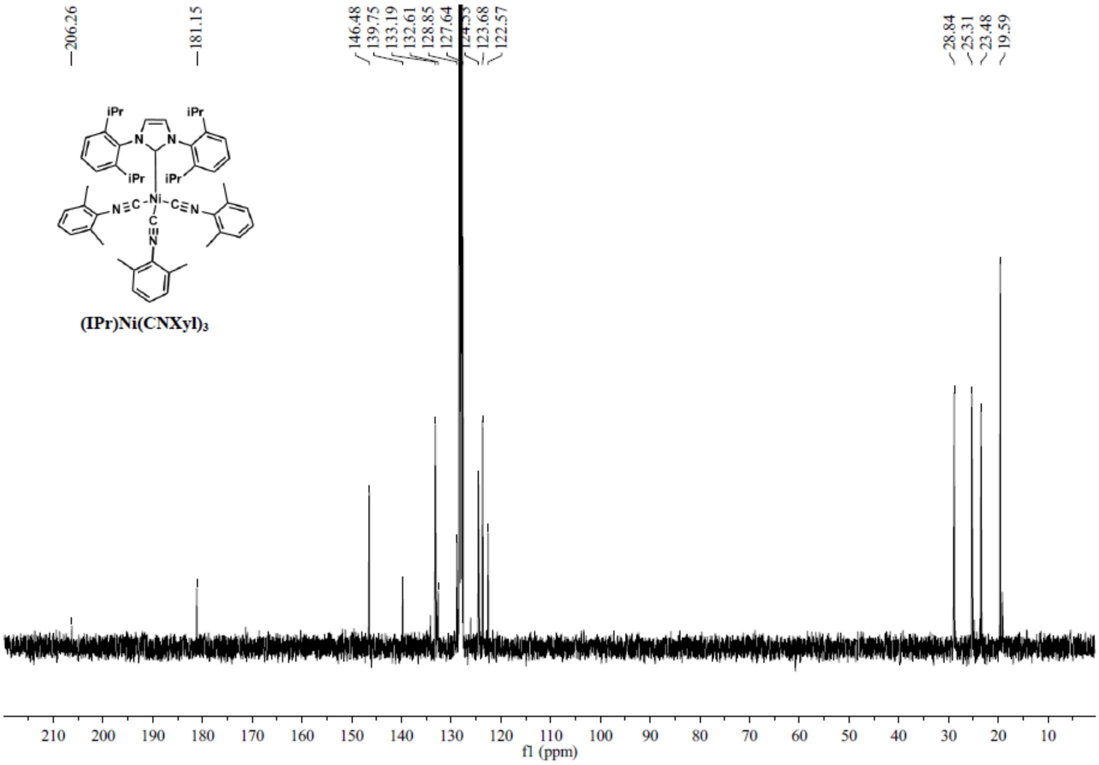

Supplementary Figure 174. ^{13}C-NMR of complex **(IPr)Ni(CNXyl)$_3$**, recorded at 100 MHz and 25 °C in C_6D_6.

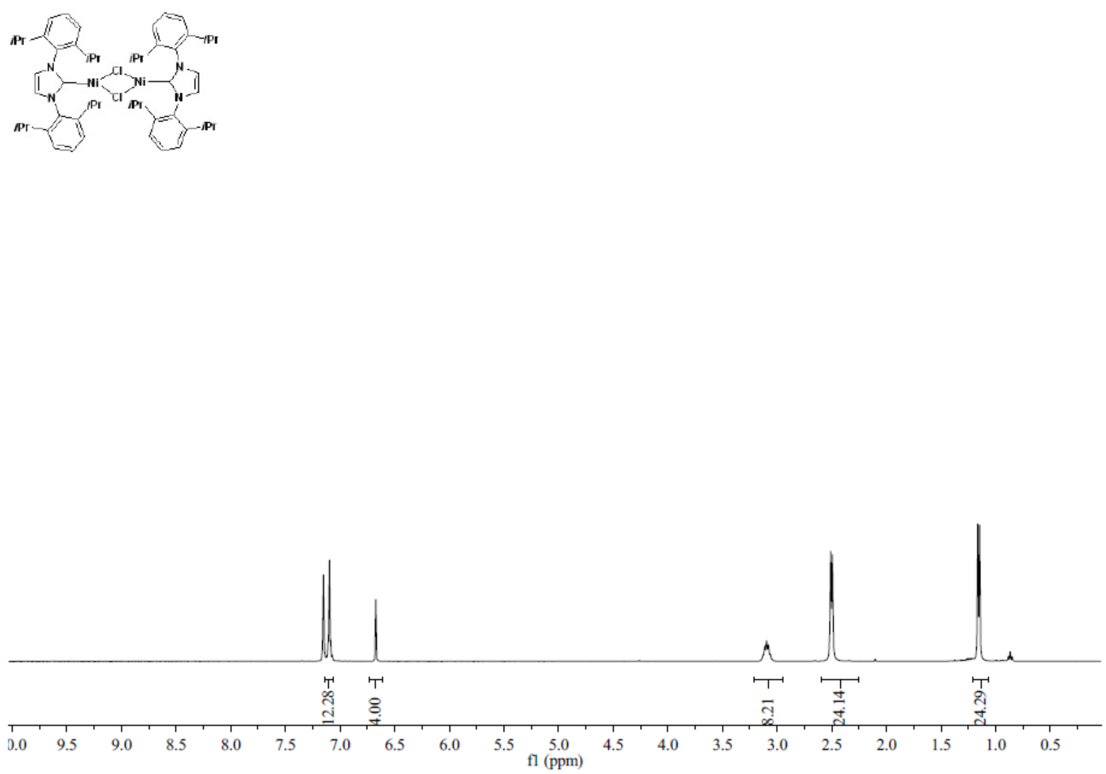

Supplementary Figure 175. ^{1}H-NMR of complex **[(IPr)NiCl]$_2$**, recorded at 400 MHz and 25 °C in C_6D_6.

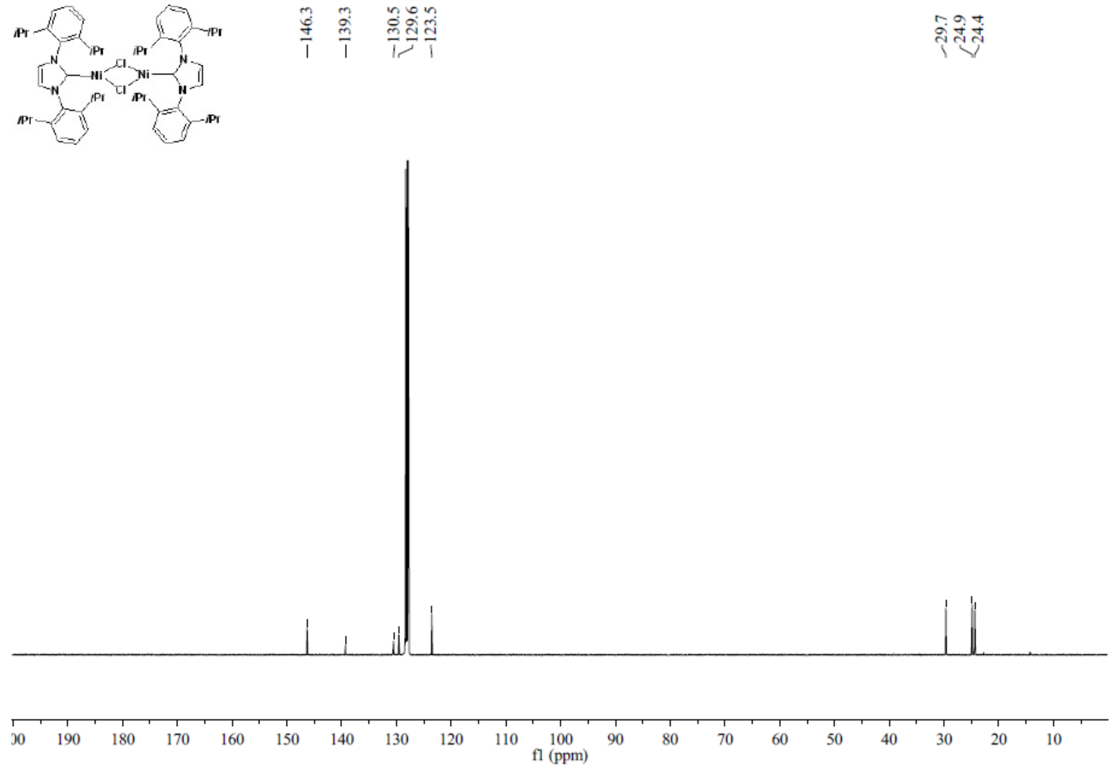

Supplementary Figure 176. ^{13}C-NMR of complex **[(IPr)NiCl]$_2$**, recorded at 100 MHz and 25 °C in C_6D_6.

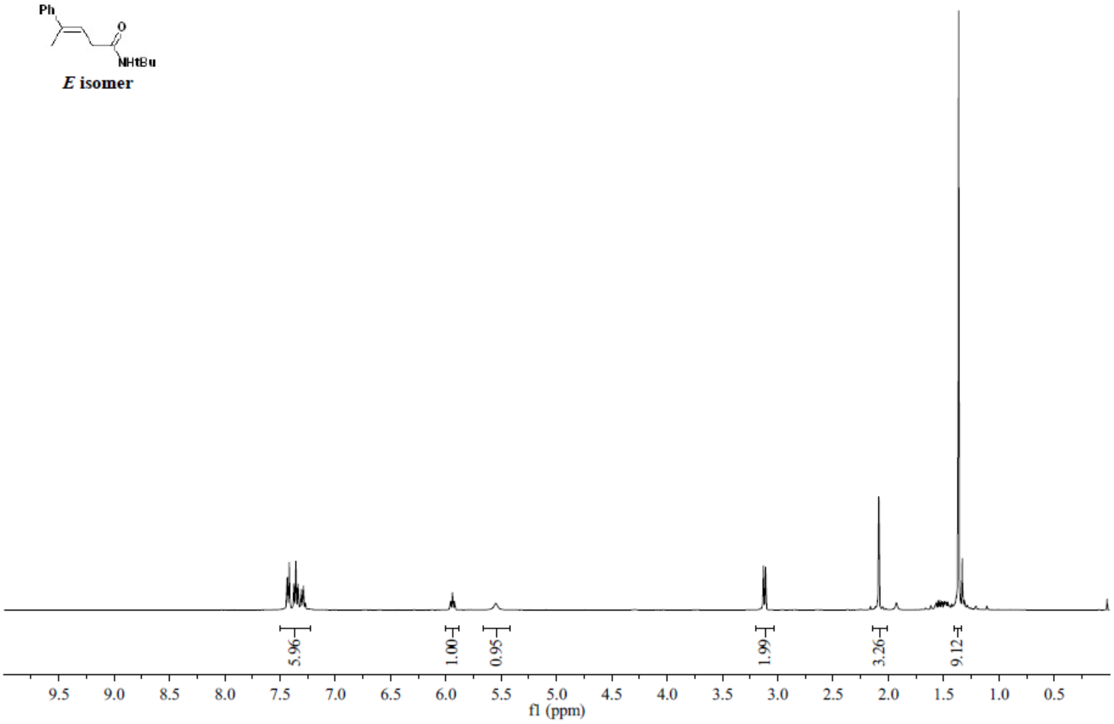

Supplementary Figure 177. ^{1}H-NMR of *E*-amide, recorded at 400 MHz and 25 °C in $CDCl_3$.

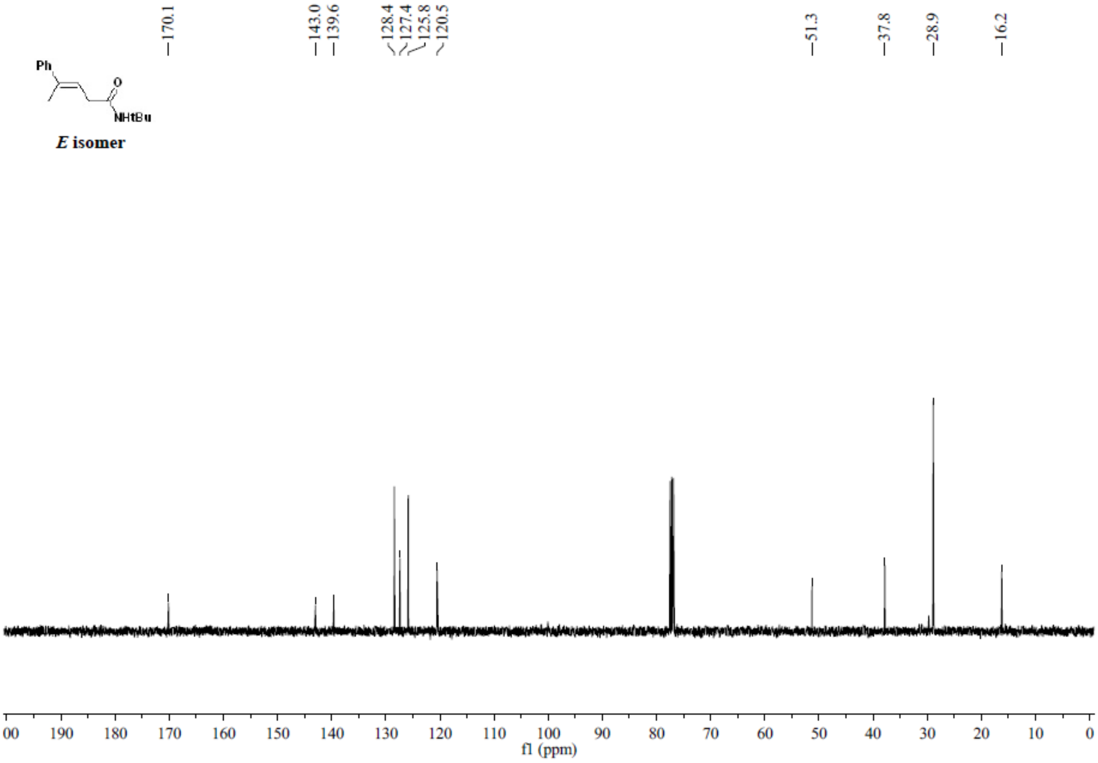

Supplementary Figure 178. ^{13}C-NMR of *E*-amide, recorded at 100 MHz and 25 °C in $CDCl_3$.

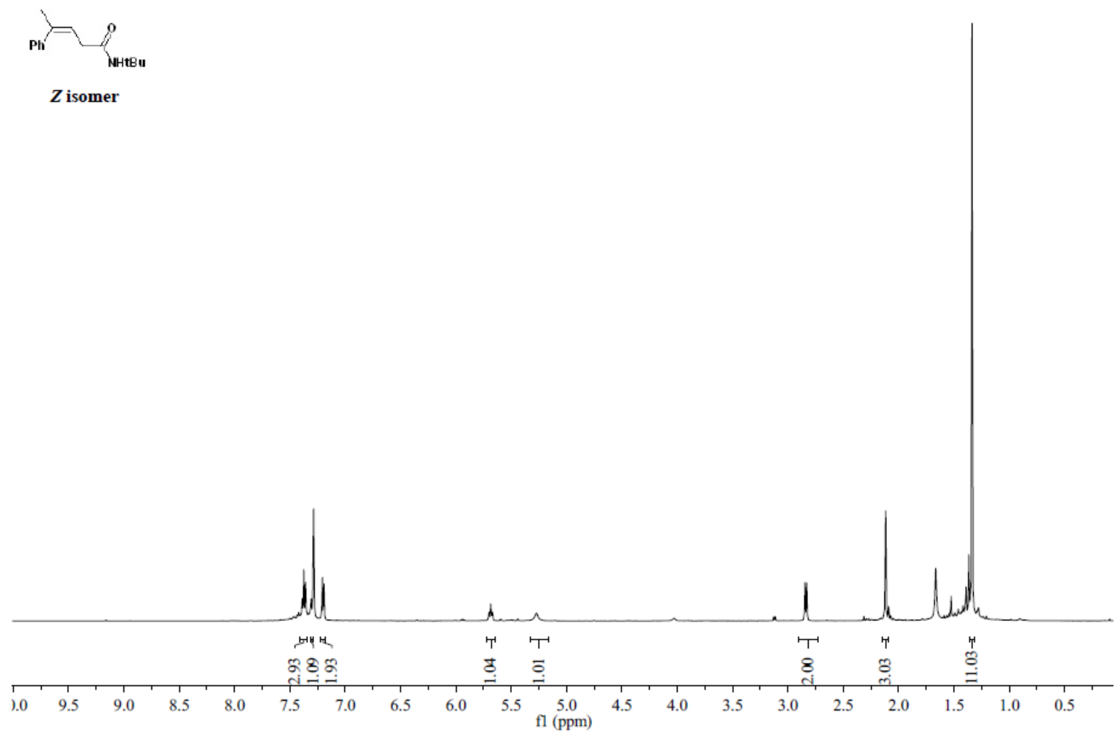

Supplementary Figure 179. ^{1}H-NMR of *Z*-amide, recorded at 500 MHz and 25 °C in $CDCl_3$.

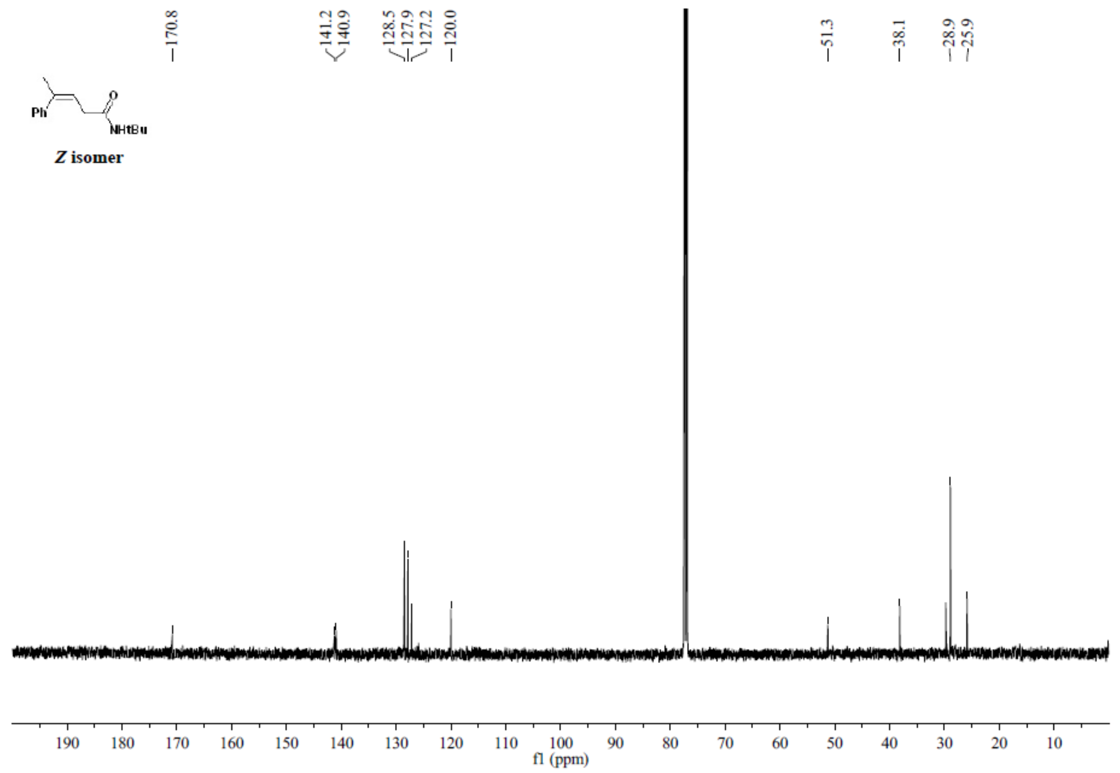

Supplementary Figure 180. ^{13}C-NMR of Z-amide, recorded at 125 MHz and 25 °C in $CDCl_3$.

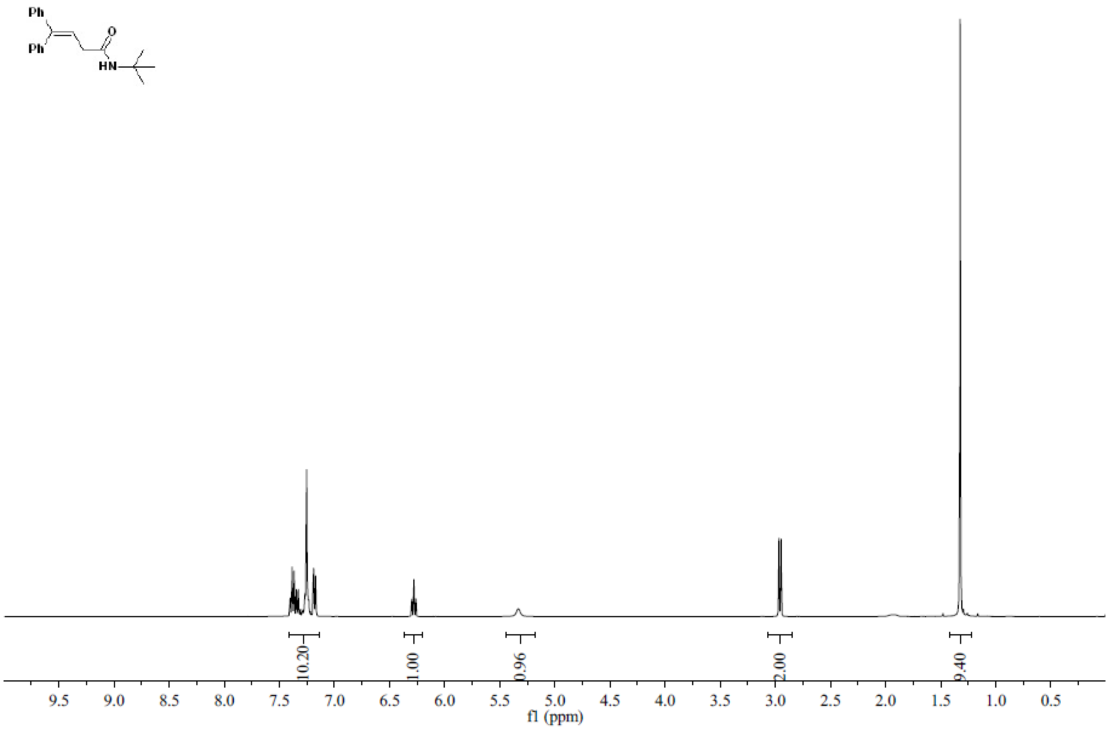

Supplementary Figure 181. ^{1}H-NMR of amide, recorded at 400 MHz and 25 °C in $CDCl_3$.

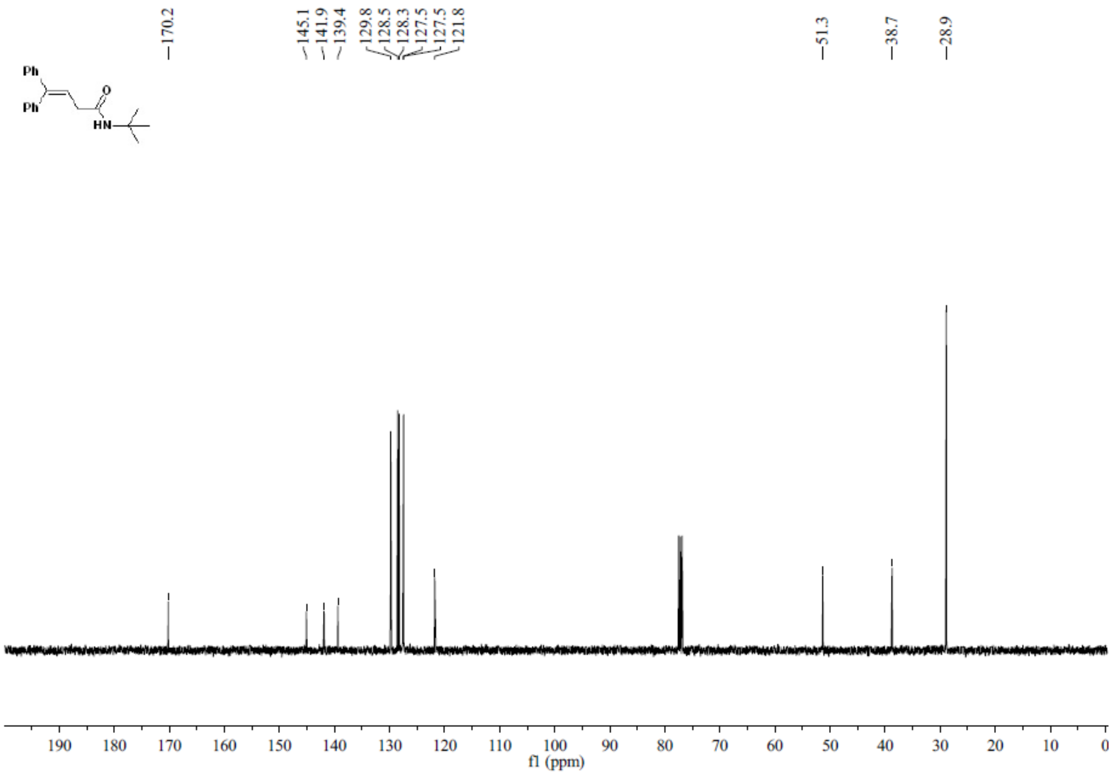

Supplementary Figure 182. ^{13}C-NMR of amide, recorded at 100 MHz and 25 °C in $CDCl_3$.

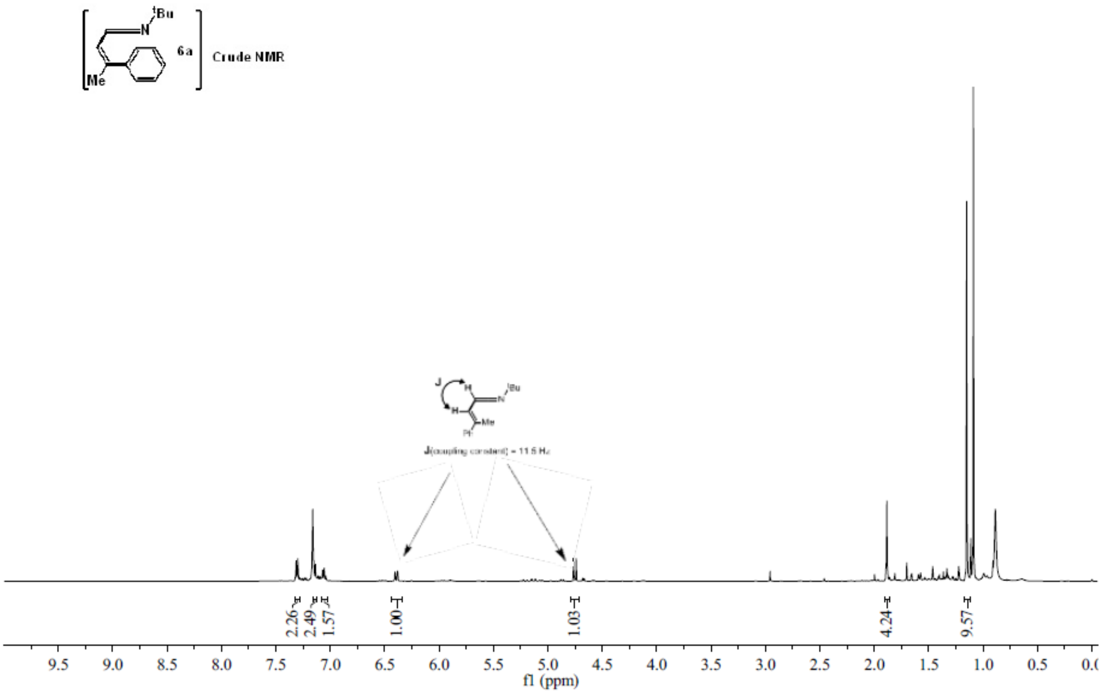

Supplementary Figure 183. ^{1}H-NMR of crude **6a**, recorded at 500 MHz and 25 °C in C_6D_6.

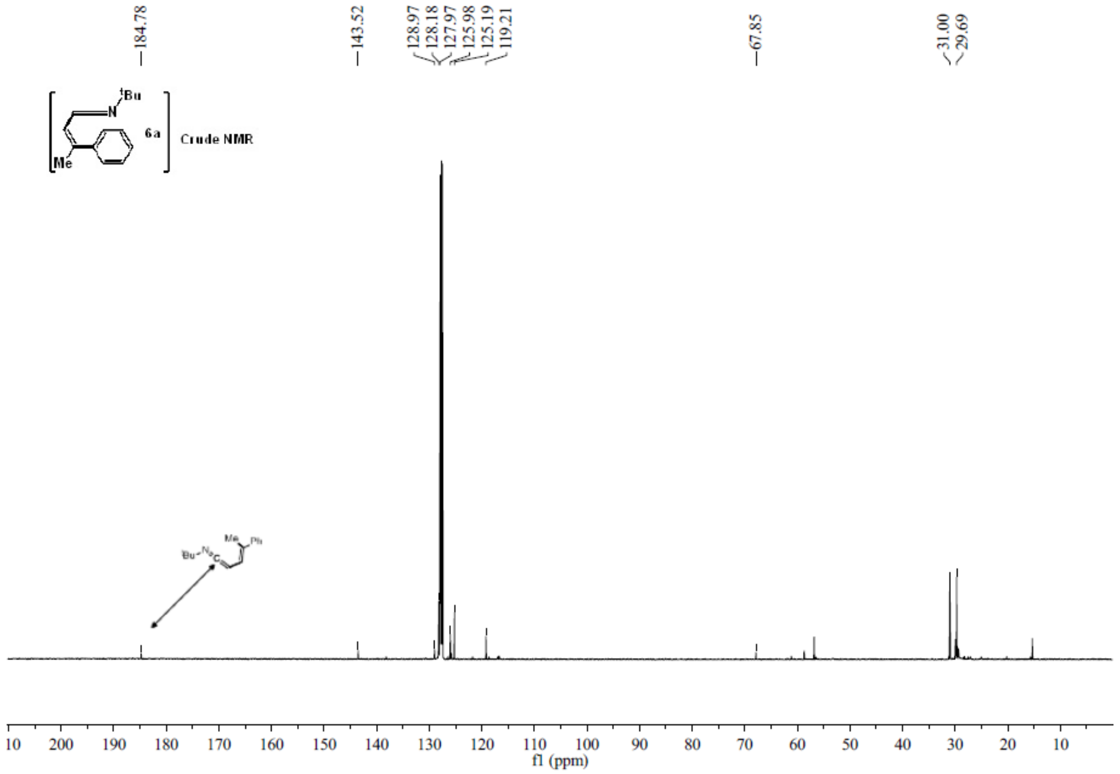

Supplementary Figure 184. ^{13}C-NMR of crude **6a**, recorded at 125 MHz and 25 °C in C_6D_6.

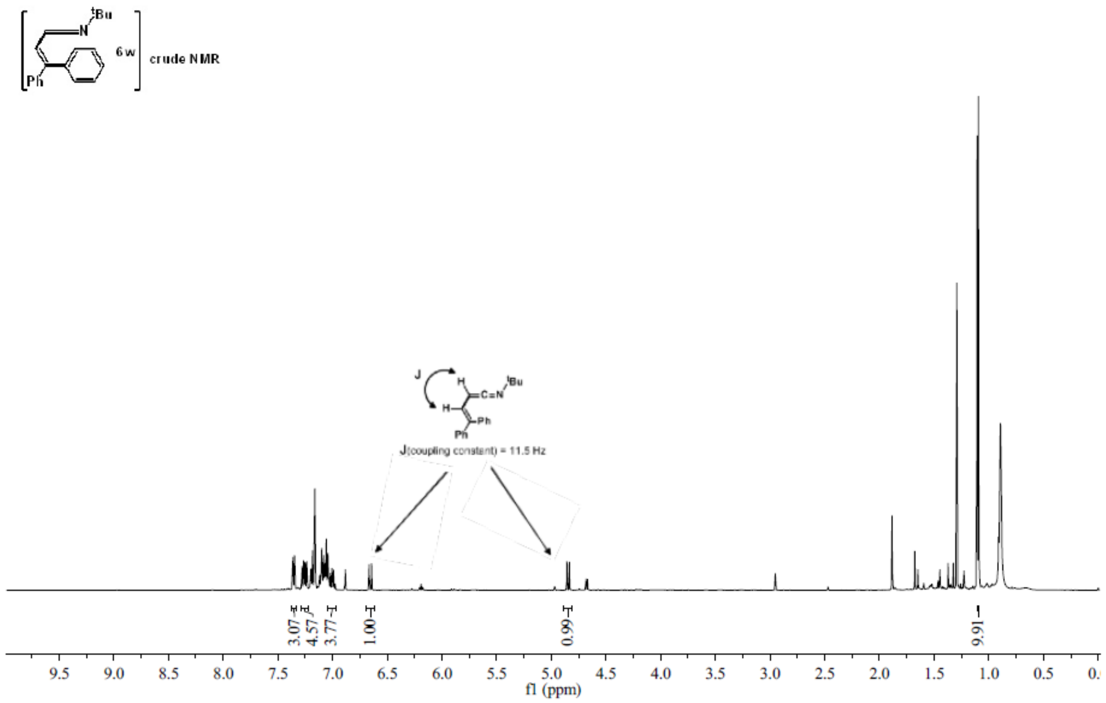

Supplementary Figure 185. ^{1}H-NMR of crude **6w**, recorded at 500 MHz and 25 °C in C_6D_6.

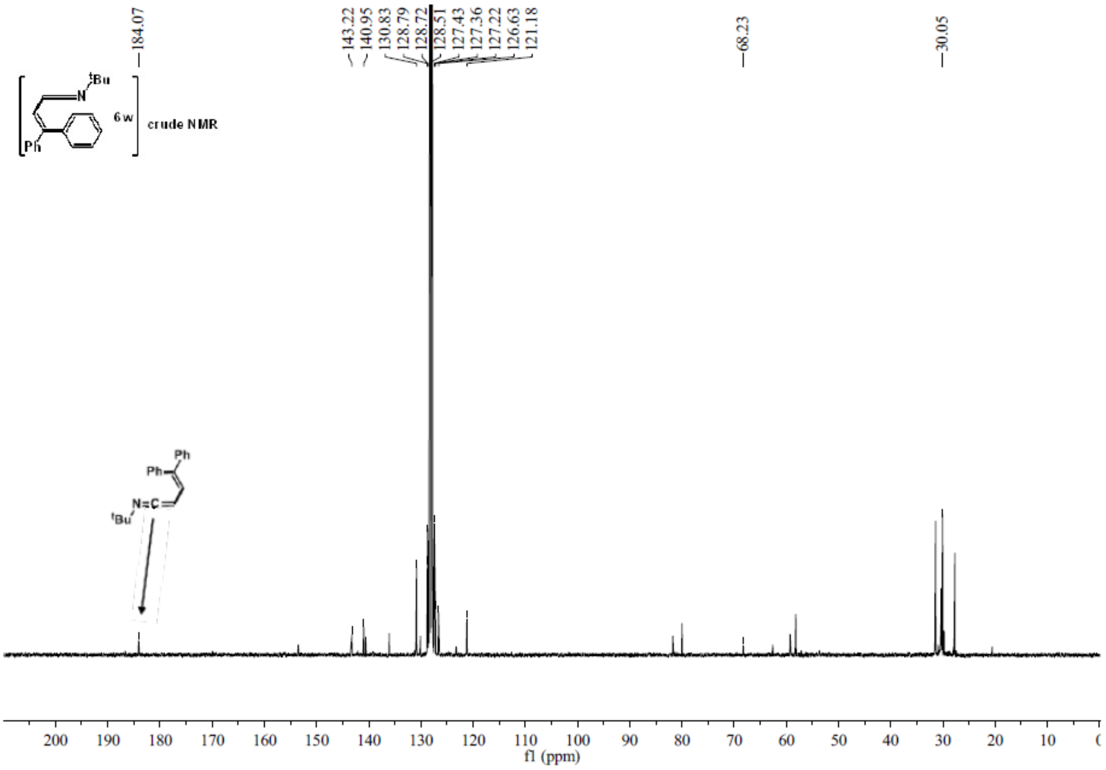

Supplementary Figure 186. ^{13}C-NMR of crude **6w**, recorded at 125 MHz and 25 °C in C_6D_6.

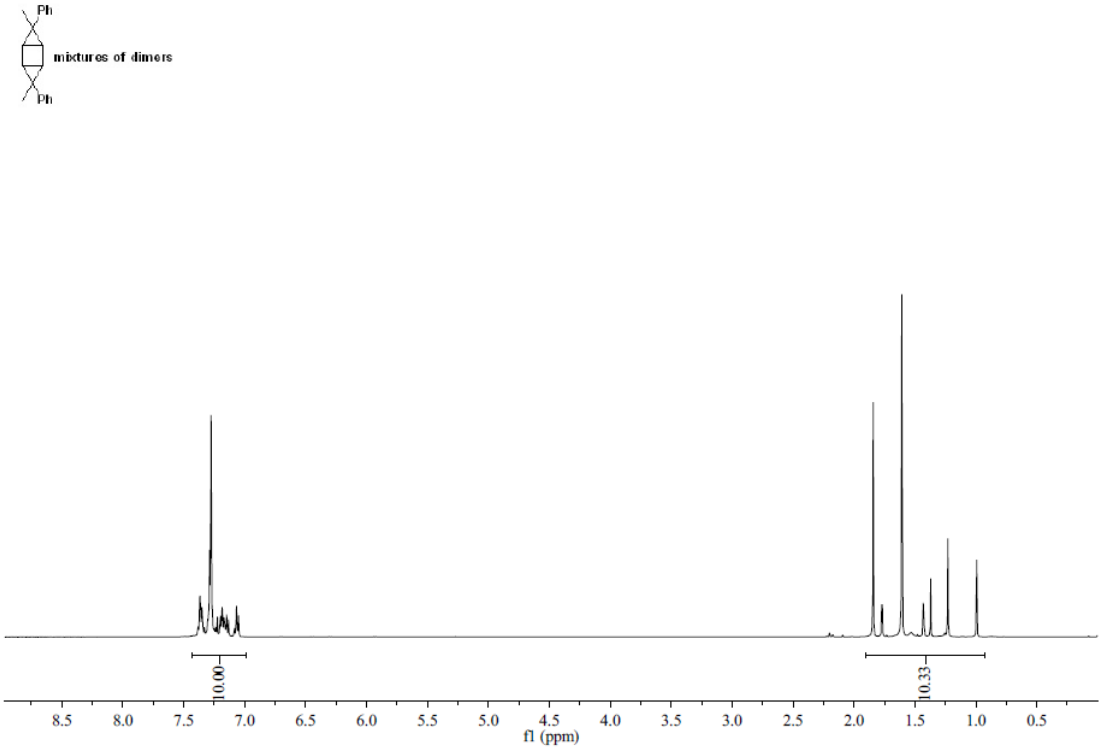

Supplementary Figure 187. ^{1}H-NMR of **dimers of 1a**, recorded at 500 MHz and 25 °C in $CDCl_3$.

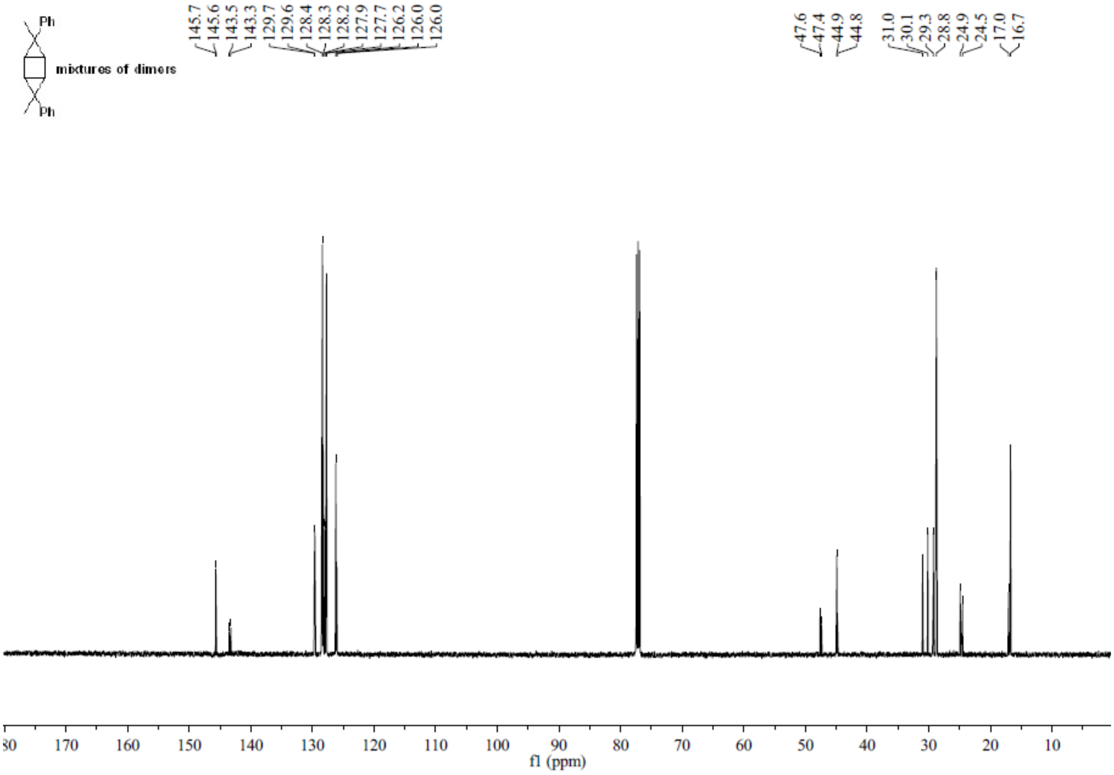

Supplementary Figure 188. ^{13}C-NMR of **dimers of 1a**, recorded at 125 MHz and 25 °C in $CDCl_3$.

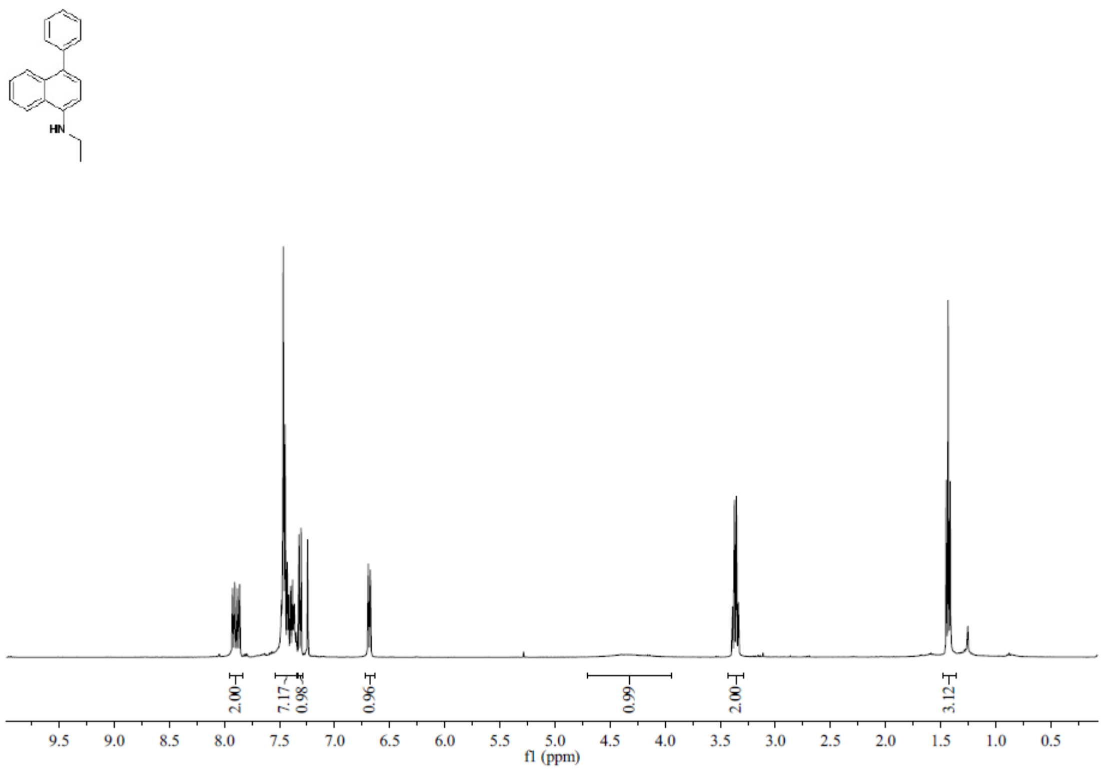

Supplementary Figure 189. ^{1}H-NMR of cyclopropyl carbonitrile alkylation/rearrangement product, recorded at 400 MHz and 25 °C in $CDCl_3$.

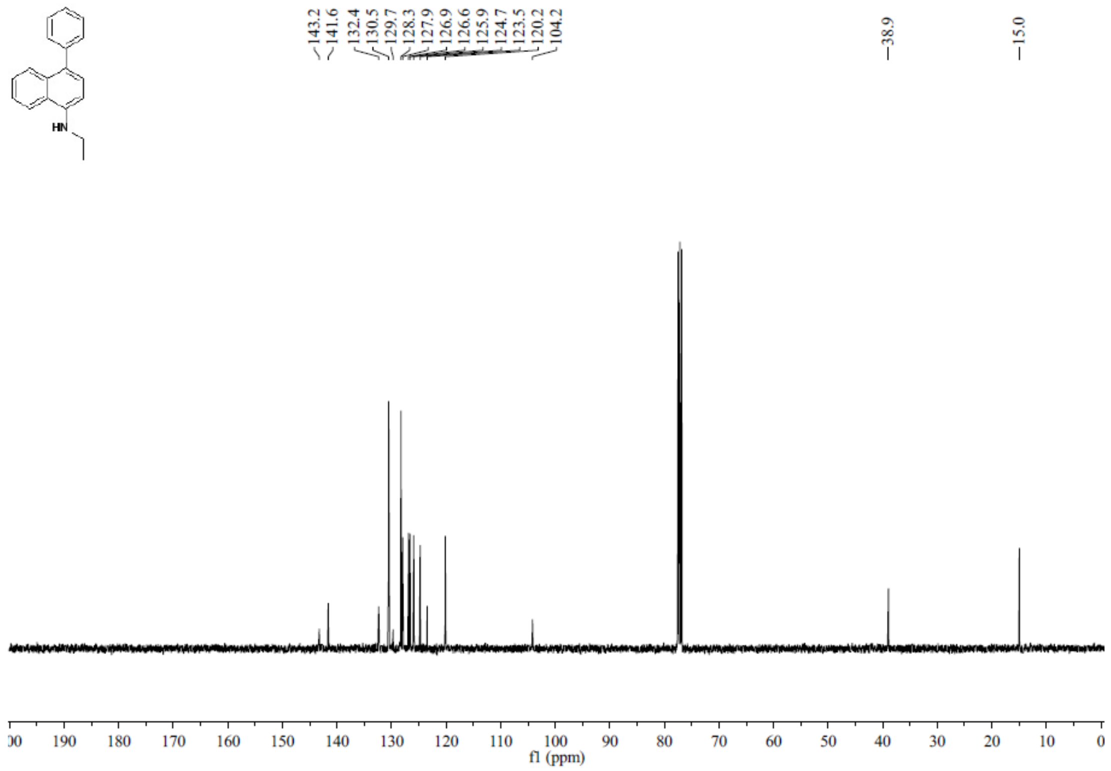

Supplementary Figure 190. ^{13}C-NMR of cyclopropyl carbonitrile alkylation/rearrangement product, recorded at 100 MHz and 25 °C in $CDCl_3$.

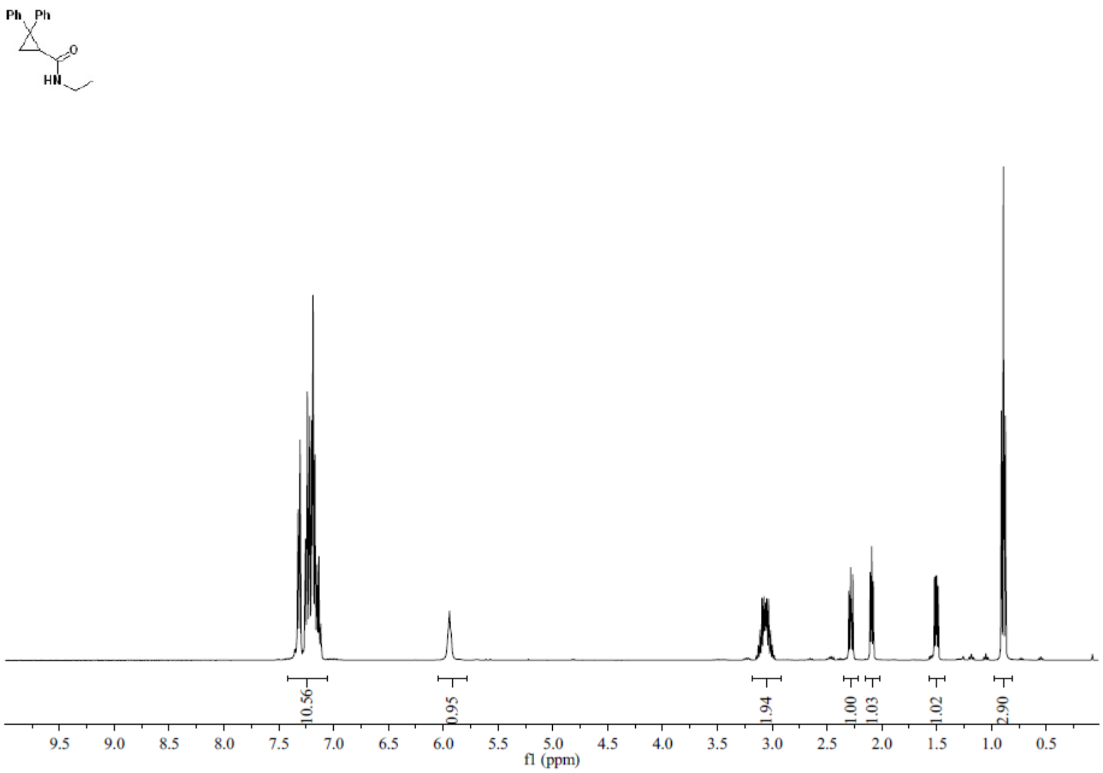

Supplementary Figure 191. ^{1}H-NMR of cyclopropyl amide, recorded at 400 MHz and 25 °C in $CDCl_3$.

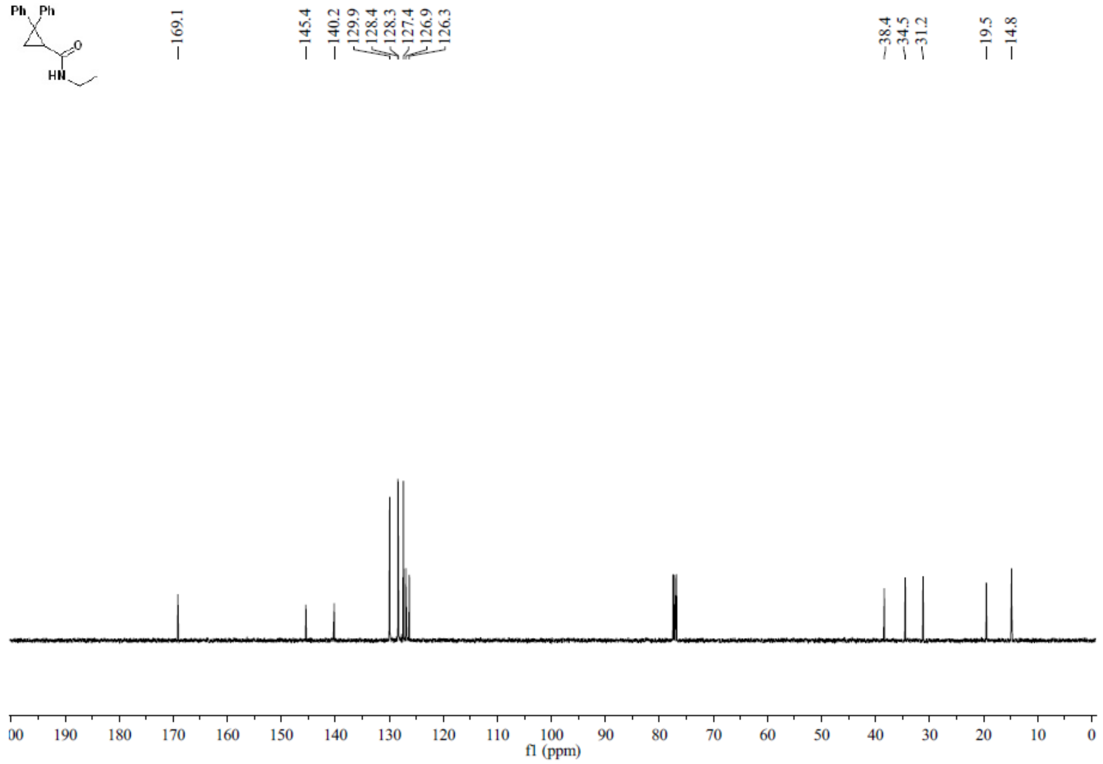

Supplementary Figure 192. ^{13}C-NMR of cyclopropyl amide, recorded at 100 MHz and 25 °C in $CDCl_3$.

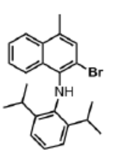

3ab^{Br}-1

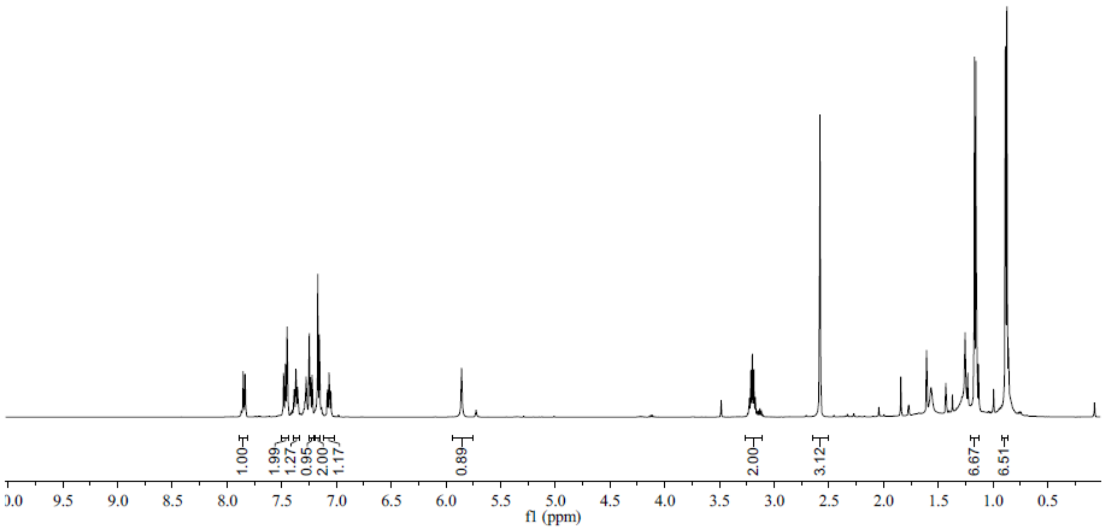

Supplementary Figure 193. ^{1}H-NMR of compound **3ab^{Br}-1**, recorded at 500 MHz and 25 °C in $CDCl_3$.

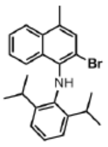

3ab^{Br}-1

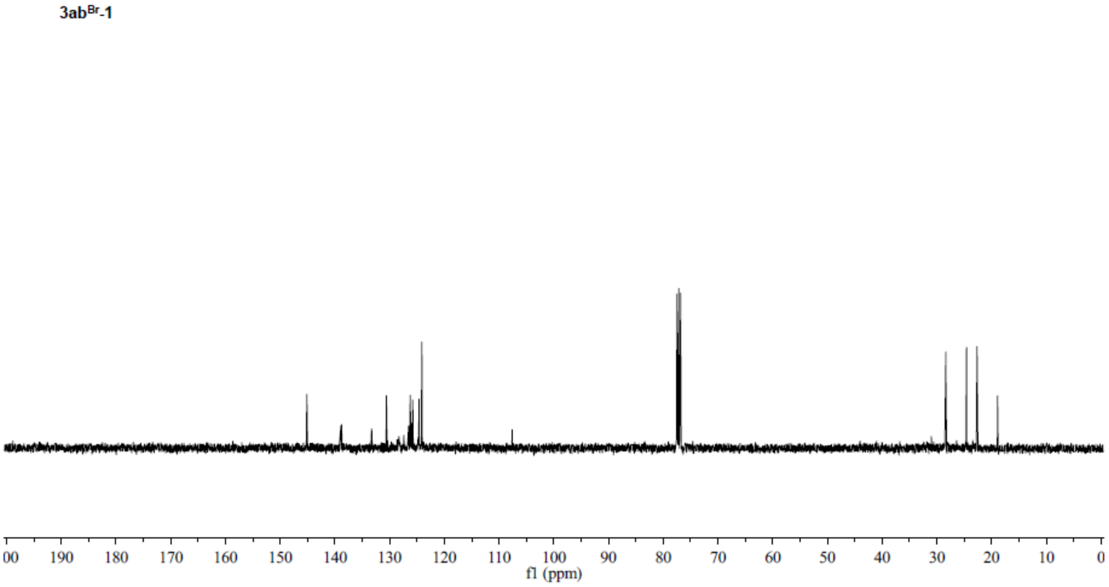

Supplementary Figure 194. ^{13}C-NMR of compound **3ab^{Br}-1**, recorded at 100 MHz and 25 °C in $CDCl_3$.

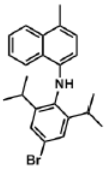

3ab^{Br}-2

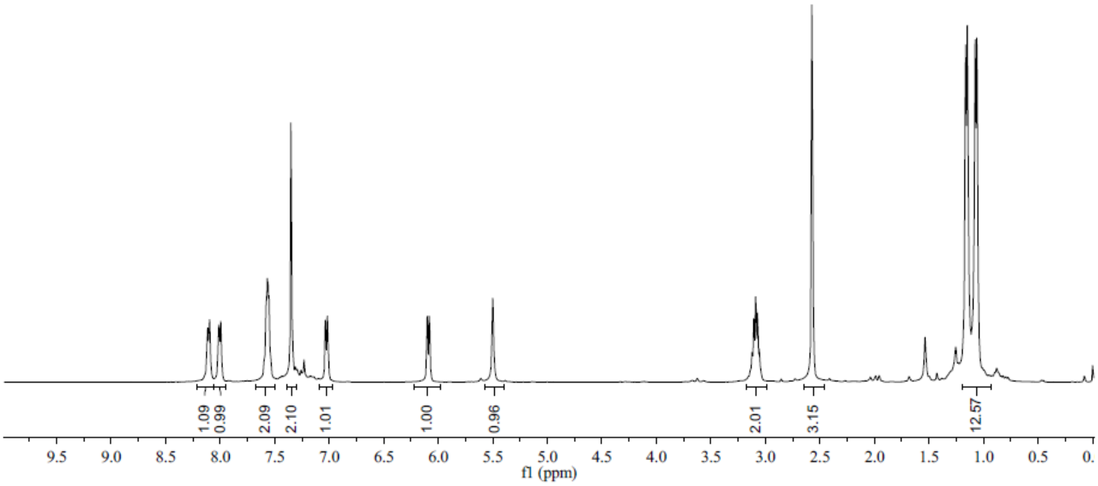

Supplementary Figure 195. ^{1}H-NMR of compound **3ab^{Br}-2**, recorded at 400 MHz and 25 °C in $CDCl_3$.

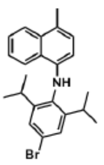

3ab^{Br}-2

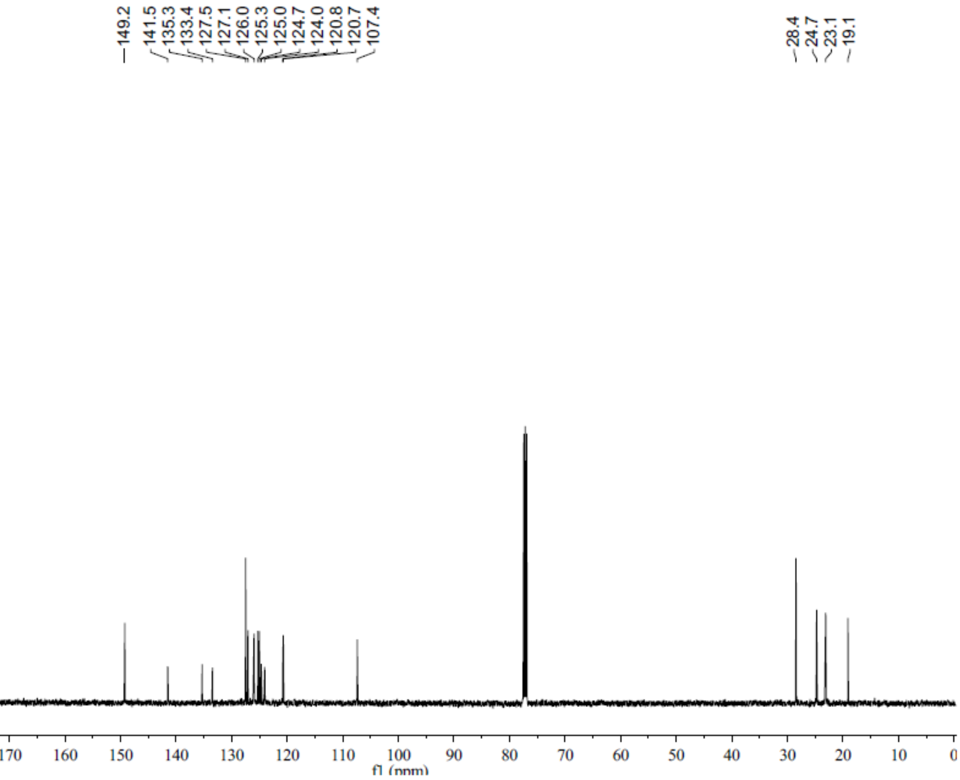

Supplementary Figure 196. ^{13}C-NMR of compound **3ab^{Br}-2**, recorded at 125 MHz and 25 °C in $CDCl_3$.

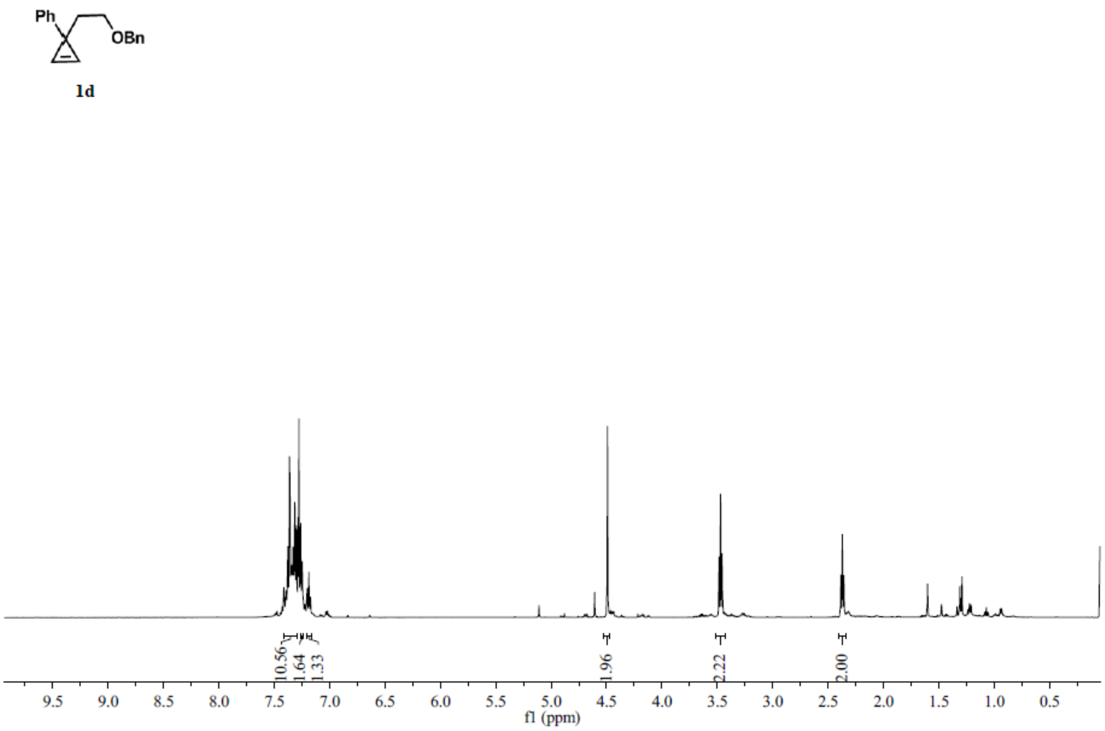

Supplementary Figure 197. ^{1}H-NMR of compound **1d**, recorded at 500 MHz and 25 °C in $CDCl_3$.

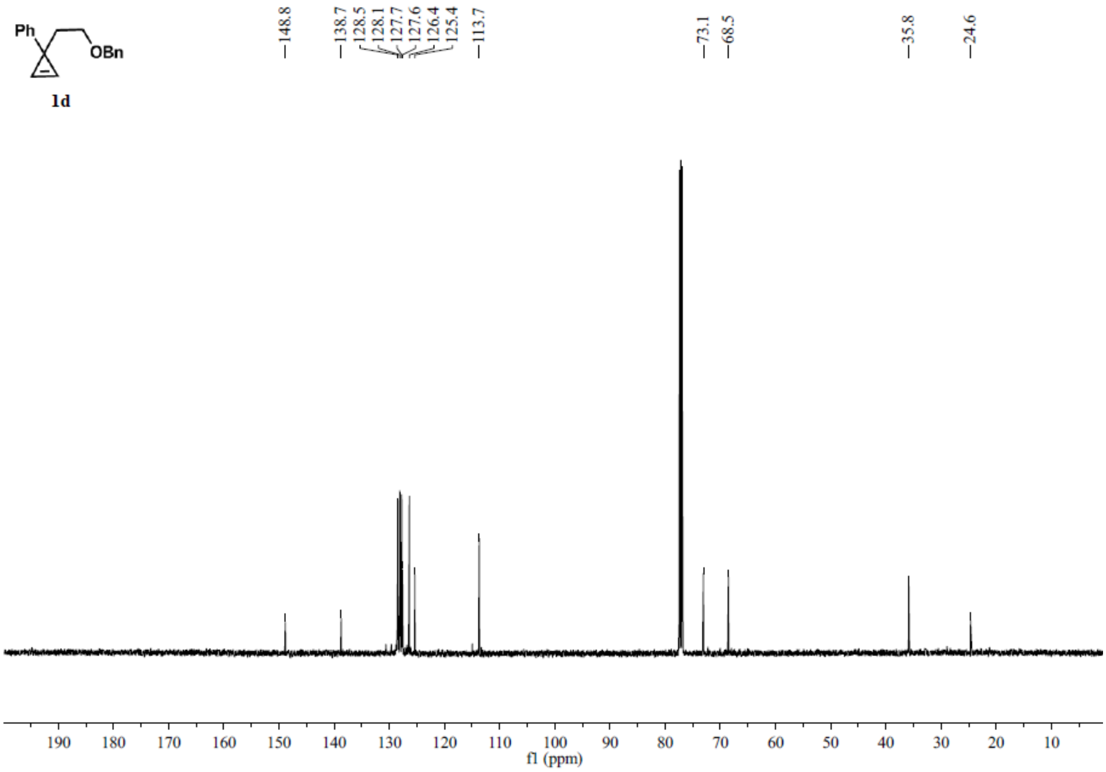

Supplementary Figure 198. ^{13}C-NMR of compound **1d**, recorded at 125 MHz and 25 °C in $CDCl_3$.

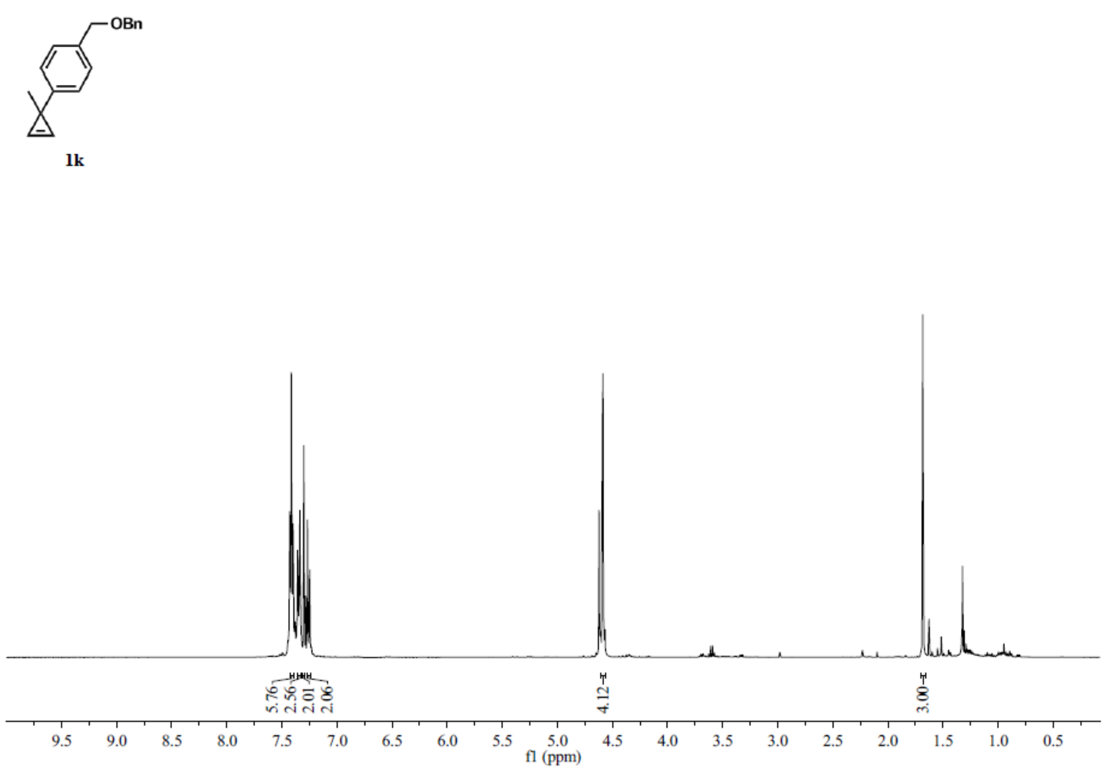

Supplementary Figure 199. ^{1}H-NMR of compound **1k**, recorded at 400 MHz and 25 °C in $CDCl_3$.

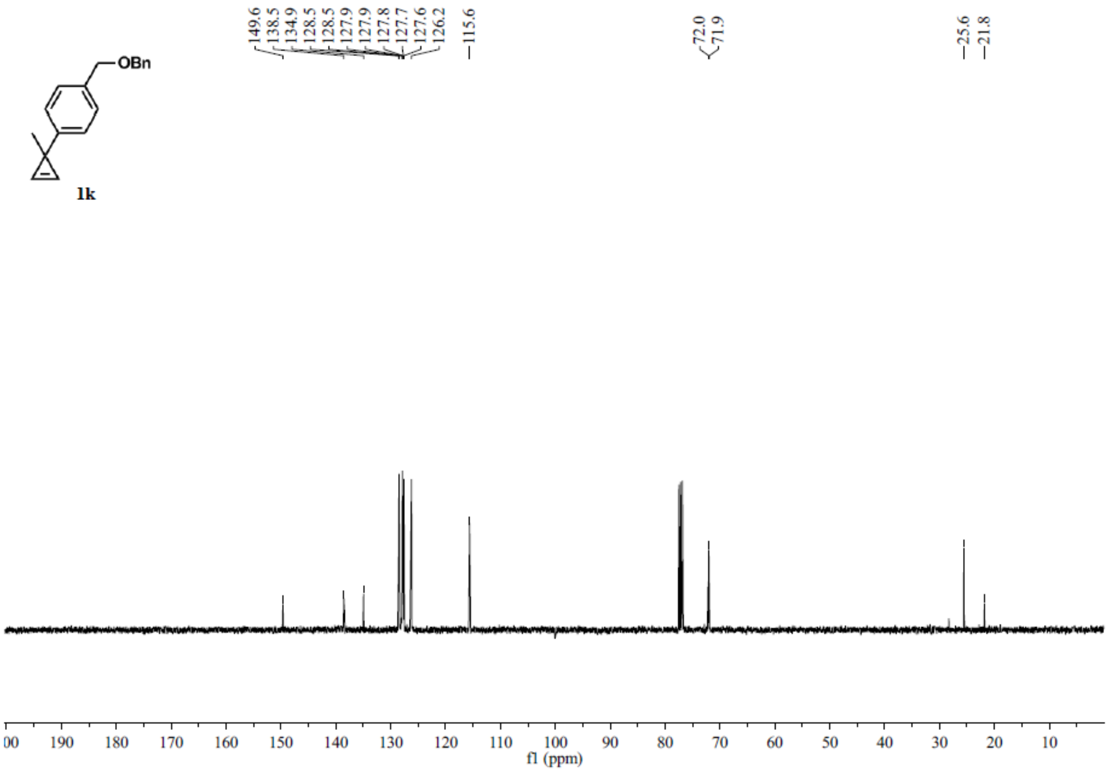

Supplementary Figure 200. ^{13}C-NMR of compound **1k**, recorded at 100 MHz and 25 °C in $CDCl_3$.

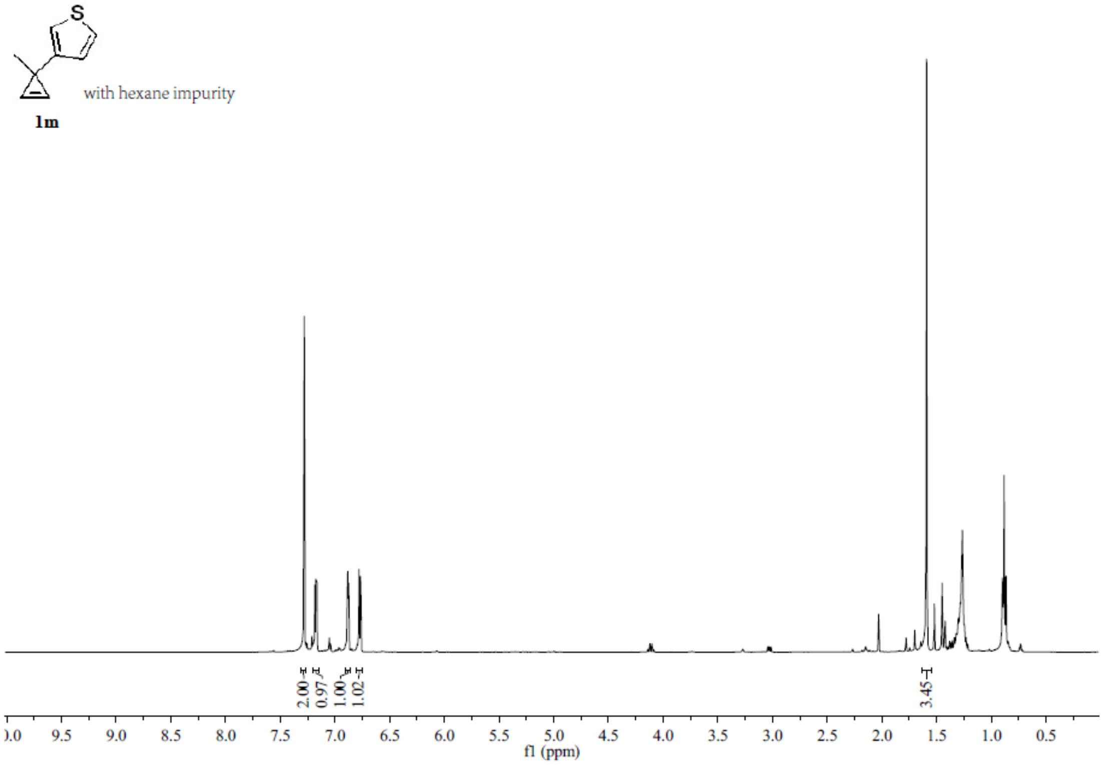

Supplementary Figure 201. ^{1}H-NMR of compound **1m**, recorded at 400 MHz and 25 °C in $CDCl_3$.

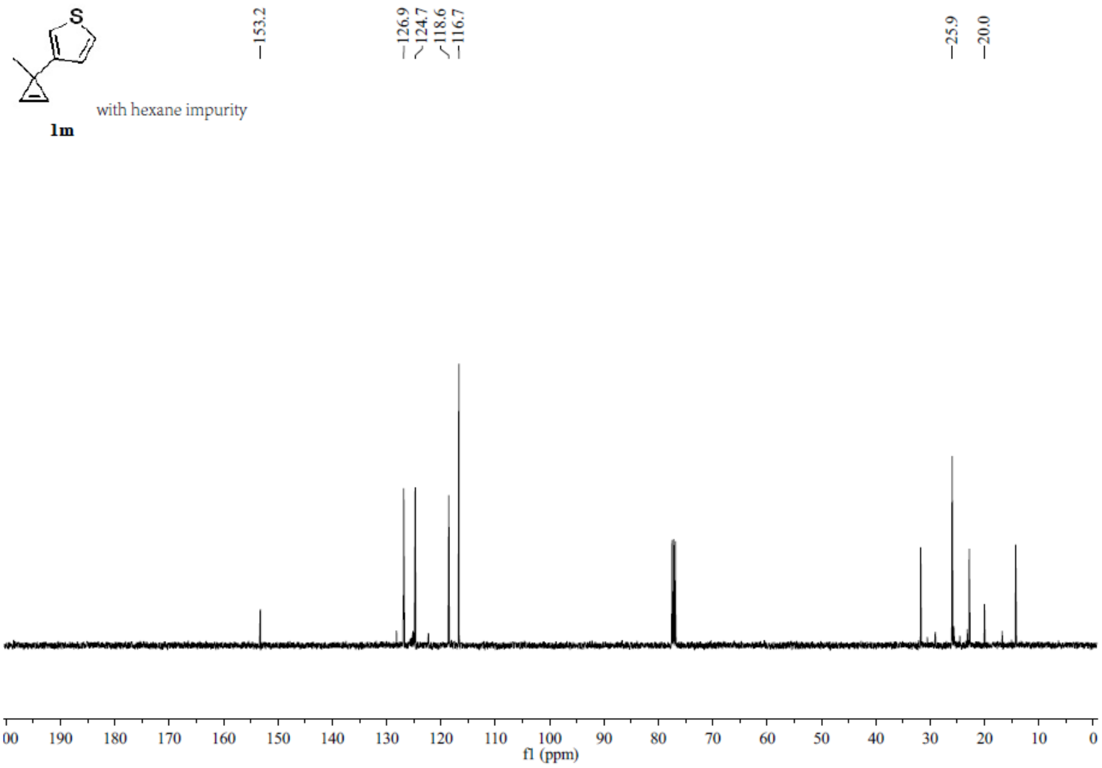

Supplementary Figure 202. ^{13}C-NMR of compound **1m**, recorded at 100 MHz and 25 °C in $CDCl_3$.

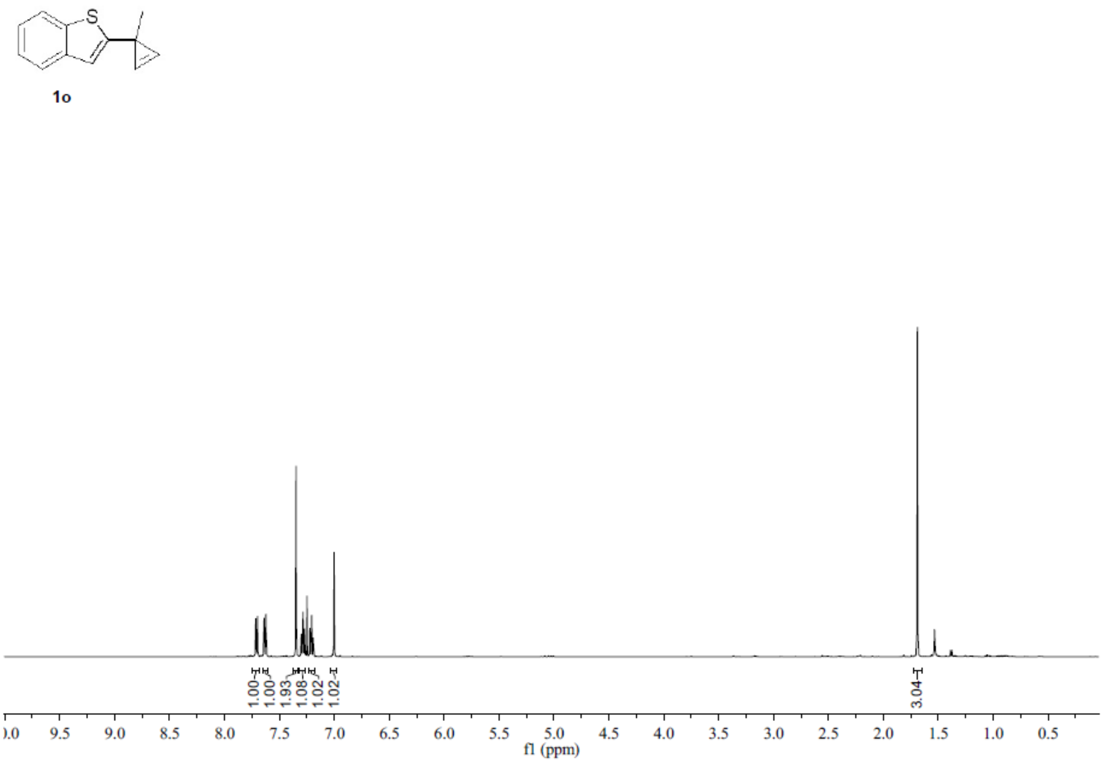

Supplementary Figure 203. ^{1}H-NMR of compound **1o**, recorded at 500 MHz and 25 °C in $CDCl_3$.

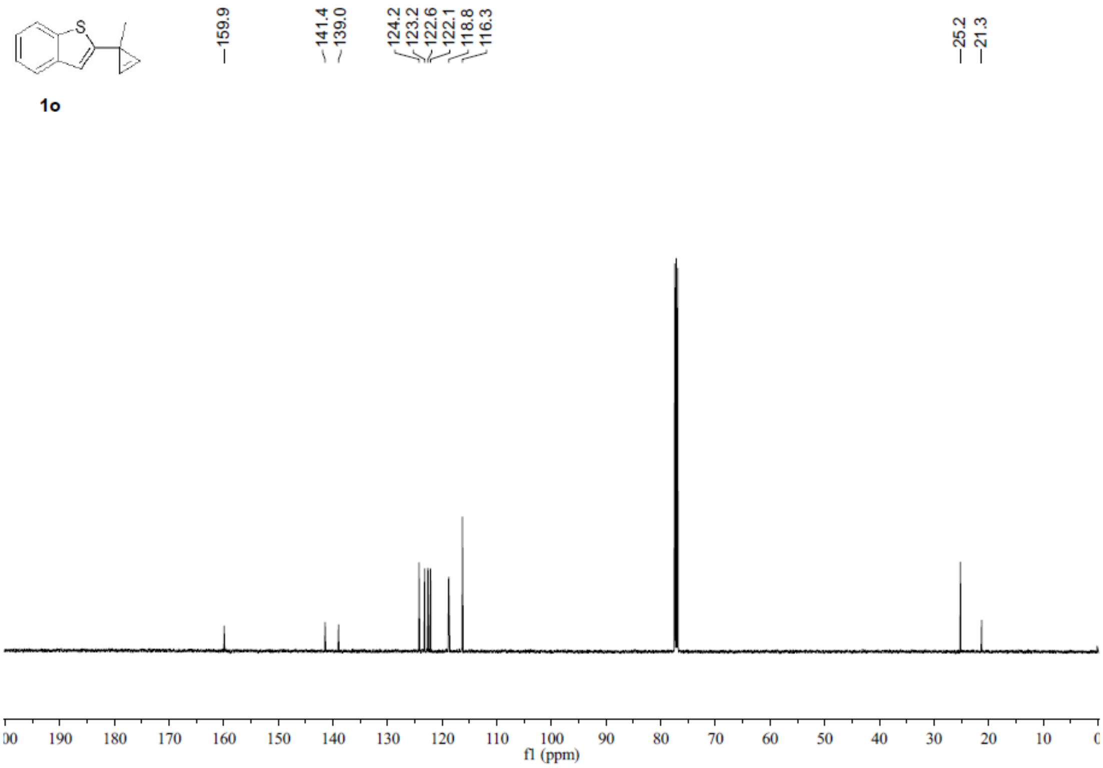

Supplementary Figure 204. ^{13}C-NMR of compound **1o**, recorded at 125 MHz and 25 °C in $CDCl_3$.

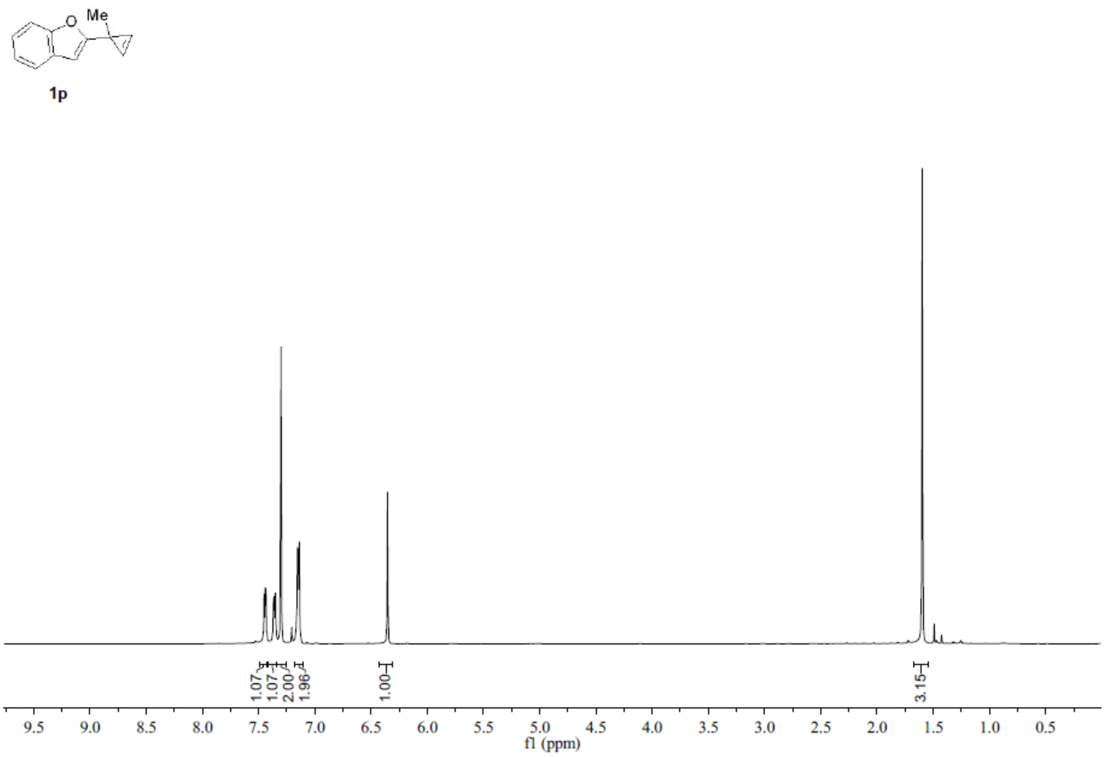

Supplementary Figure 205. ^{1}H-NMR of compound **1p**, recorded at 500 MHz and 25 °C in $CDCl_3$.

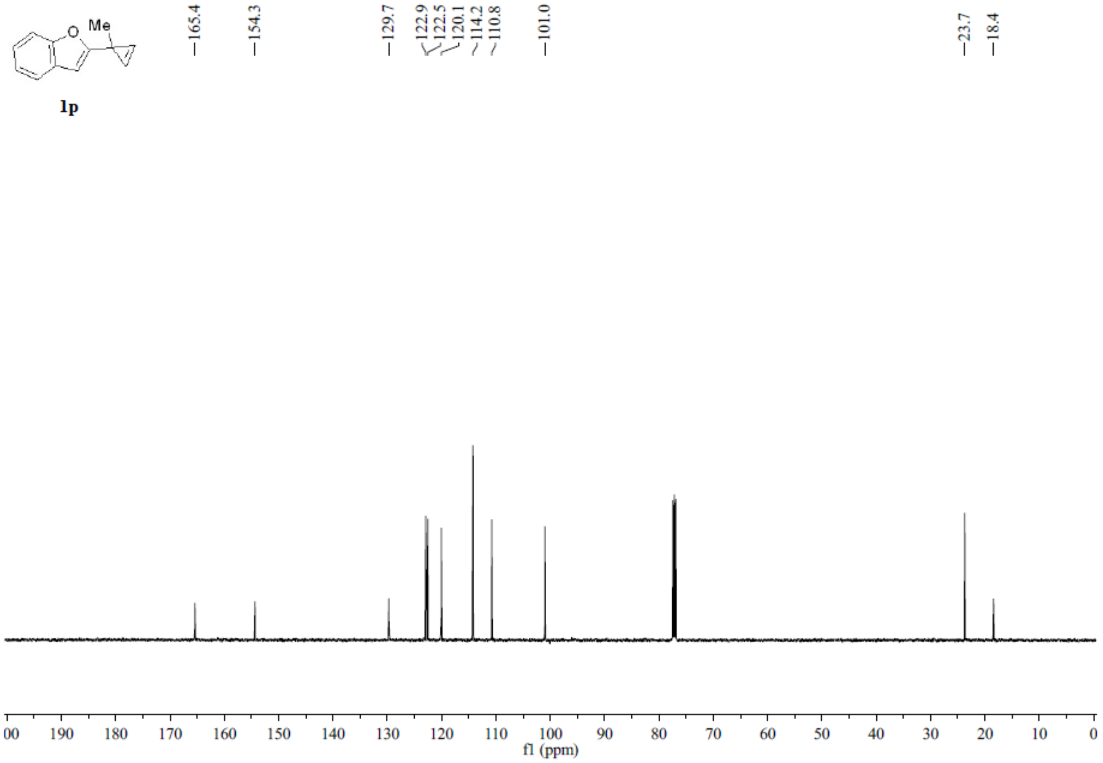

Supplementary Figure 206. ^{13}C-NMR of compound **1p**, recorded at 125 MHz and 25 °C in $CDCl_3$.

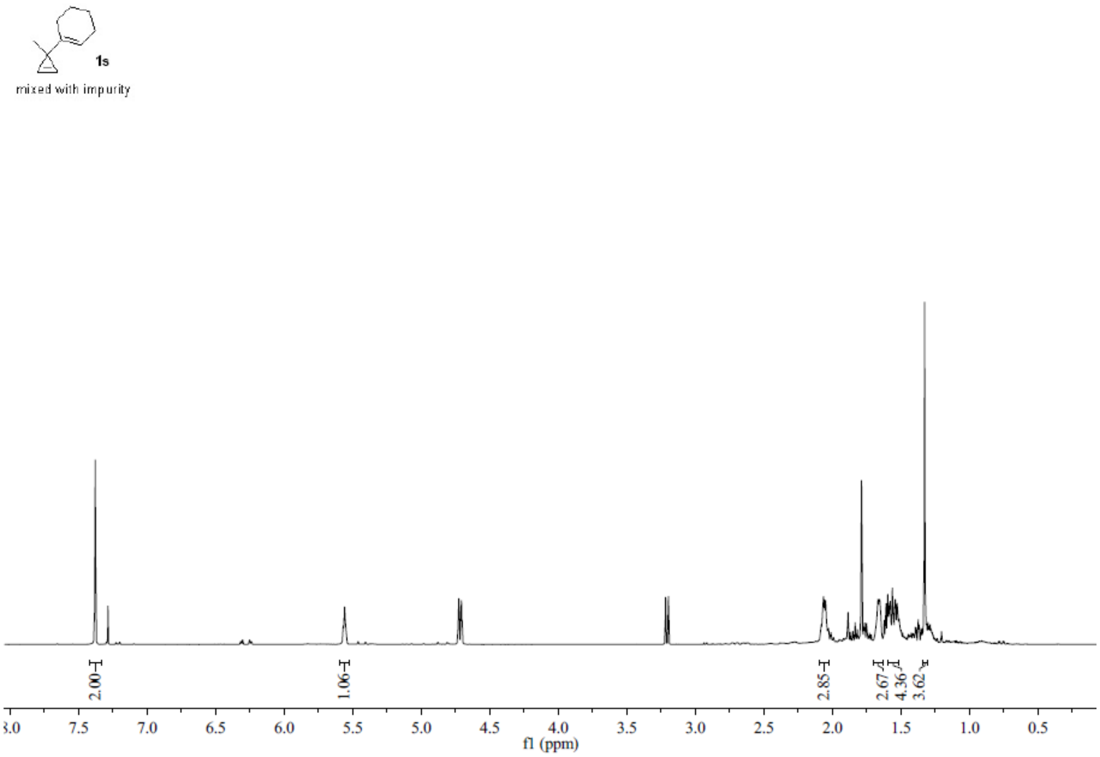

Supplementary Figure 207. ^{1}H-NMR of compound **1s**, recorded at 400 MHz and 25 °C in $CDCl_3$.

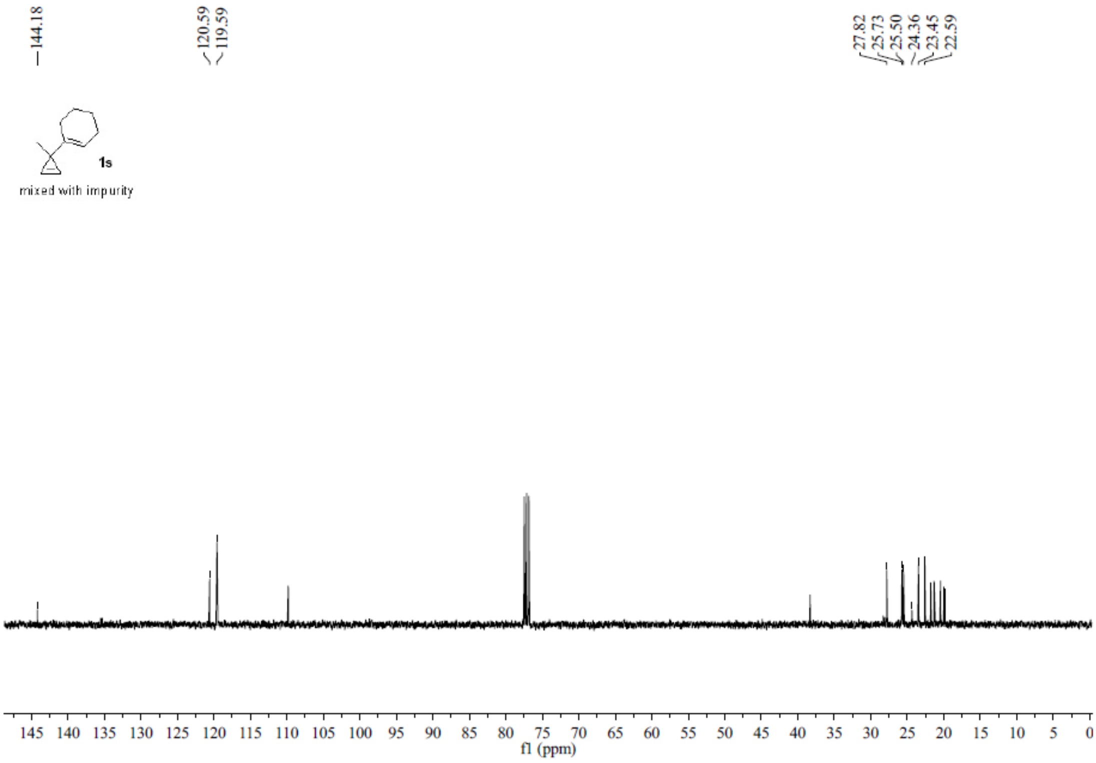

Supplementary Figure 208. ^{13}C-NMR of compound **1s**, recorded at 100 MHz and 25 °C in $CDCl_3$.

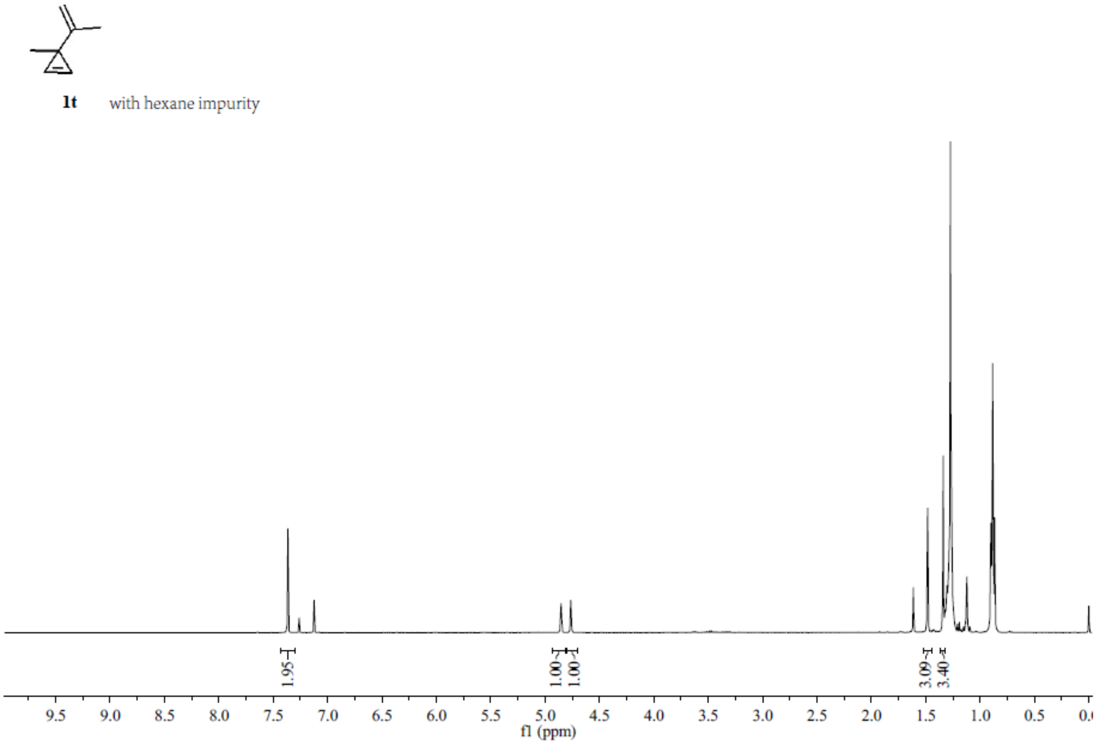

Supplementary Figure 209. ^{1}H-NMR of compound **1t**, recorded at 400 MHz and 25 °C in $CDCl_3$.

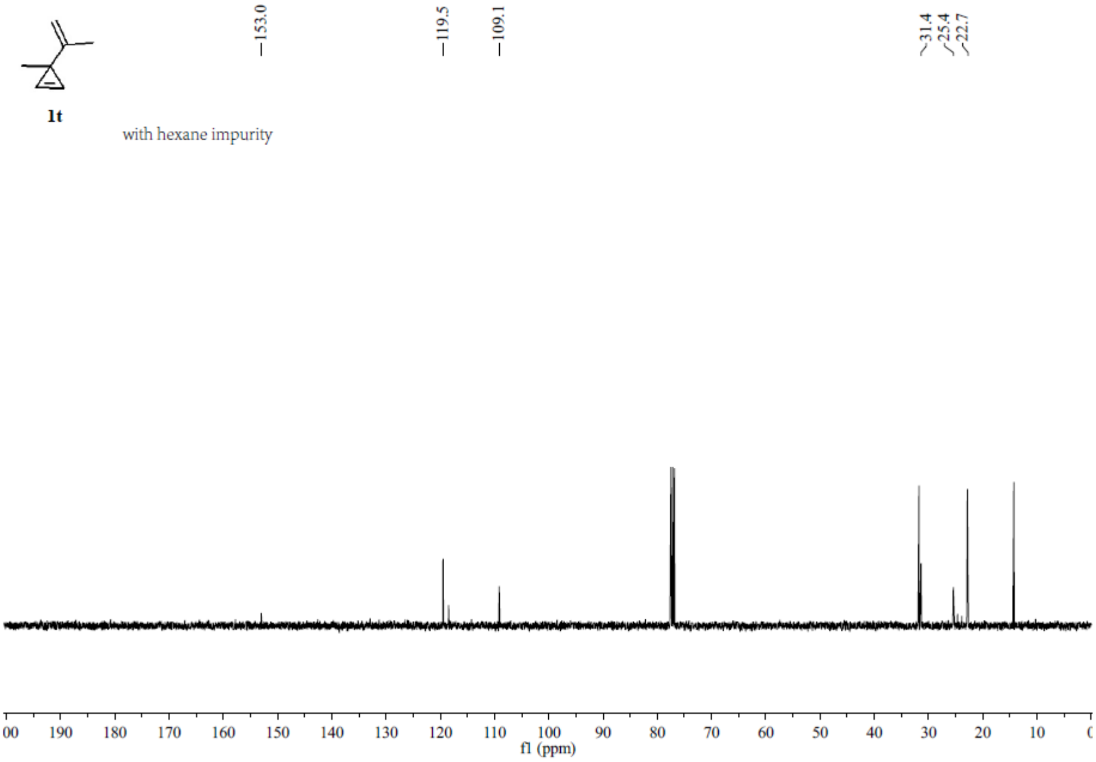

Supplementary Figure 210. ^{13}C-NMR of compound **1t**, recorded at 100 MHz and 25 °C in $CDCl_3$.

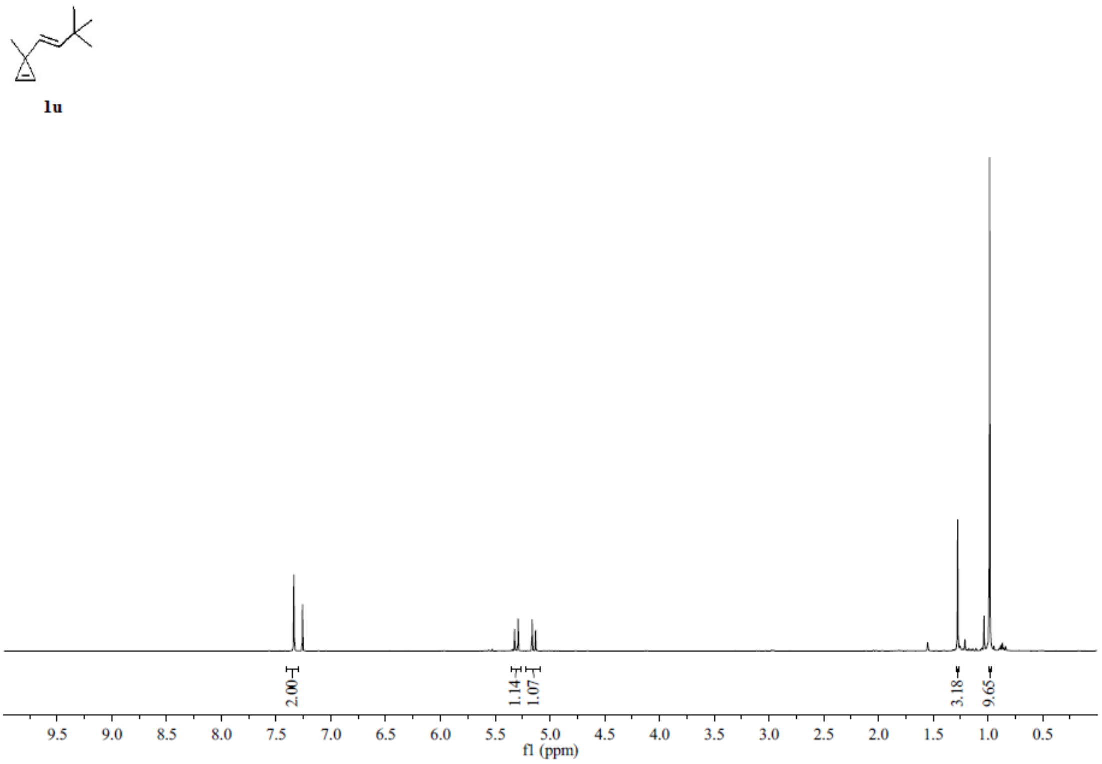

Supplementary Figure 211. ^{1}H-NMR of compound **1u**, recorded at 500 MHz and 25 °C in $CDCl_3$.

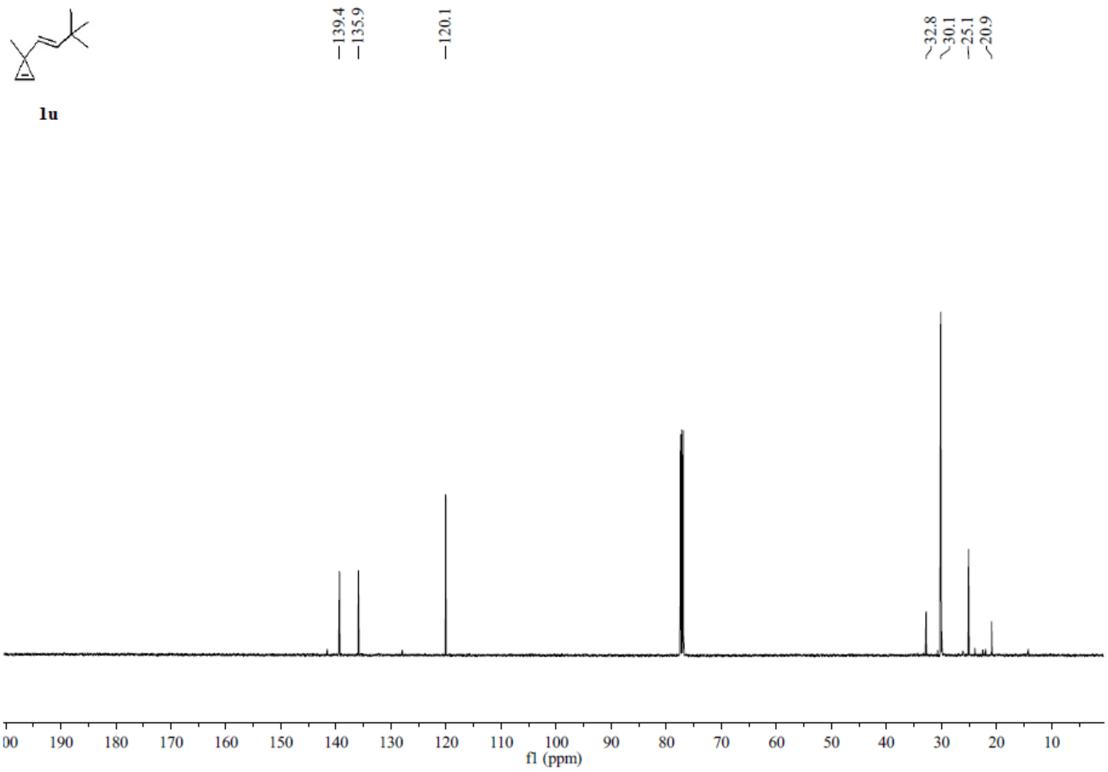

Supplementary Figure 212. ^{13}C-NMR of compound **1u**, recorded at 125 MHz and 25 °C in $CDCl_3$.

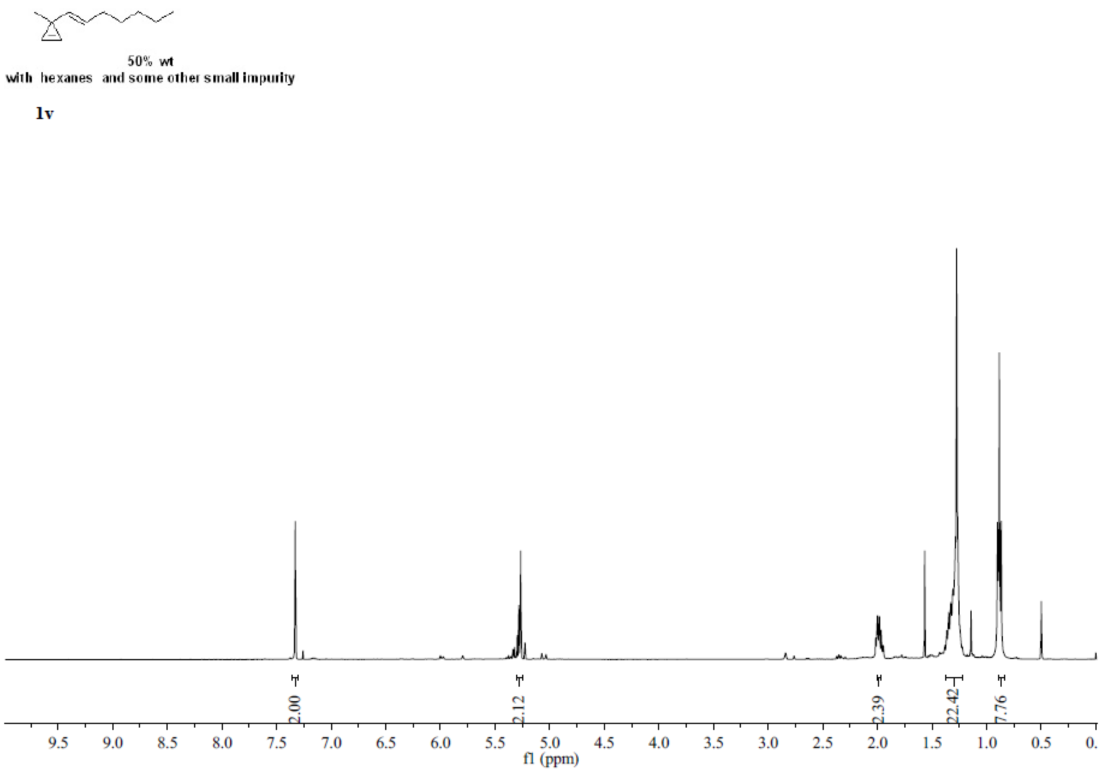

Supplementary Figure 213. ^{1}H-NMR of compound **1v**, recorded at 400 MHz and 25 °C in $CDCl_3$.

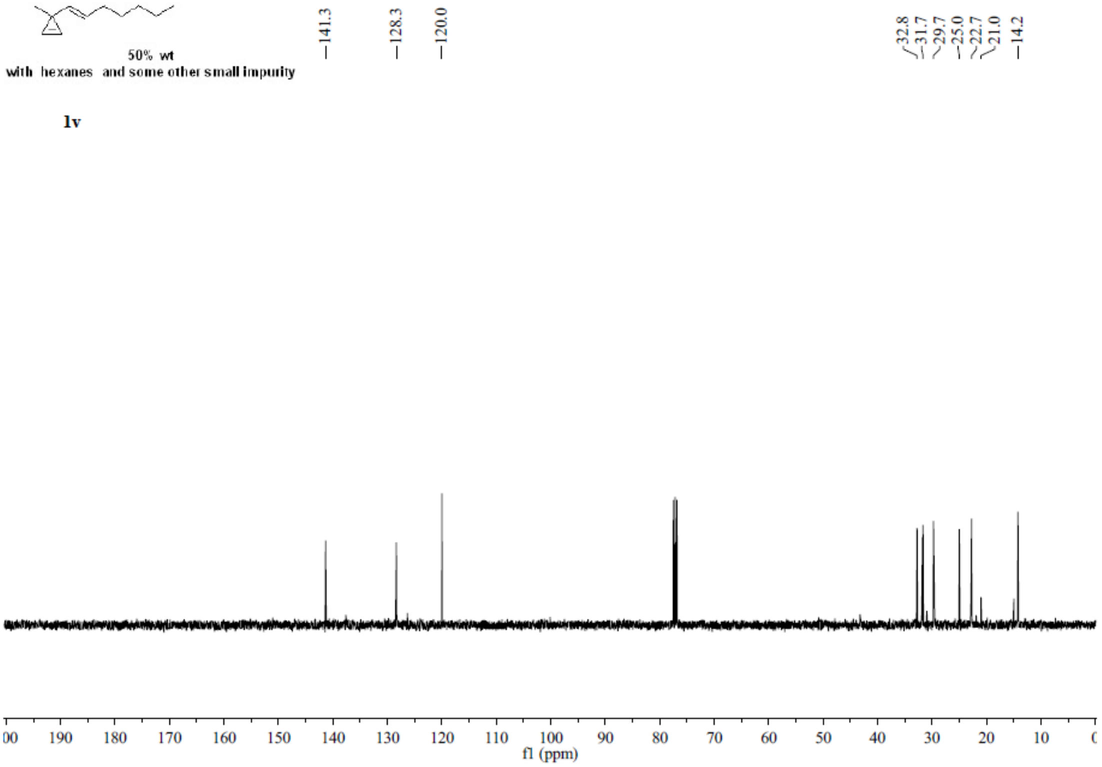

Supplementary Figure 214. ^{13}C-NMR of compound **1v**, recorded at 100 MHz and 25 °C in $CDCl_3$.

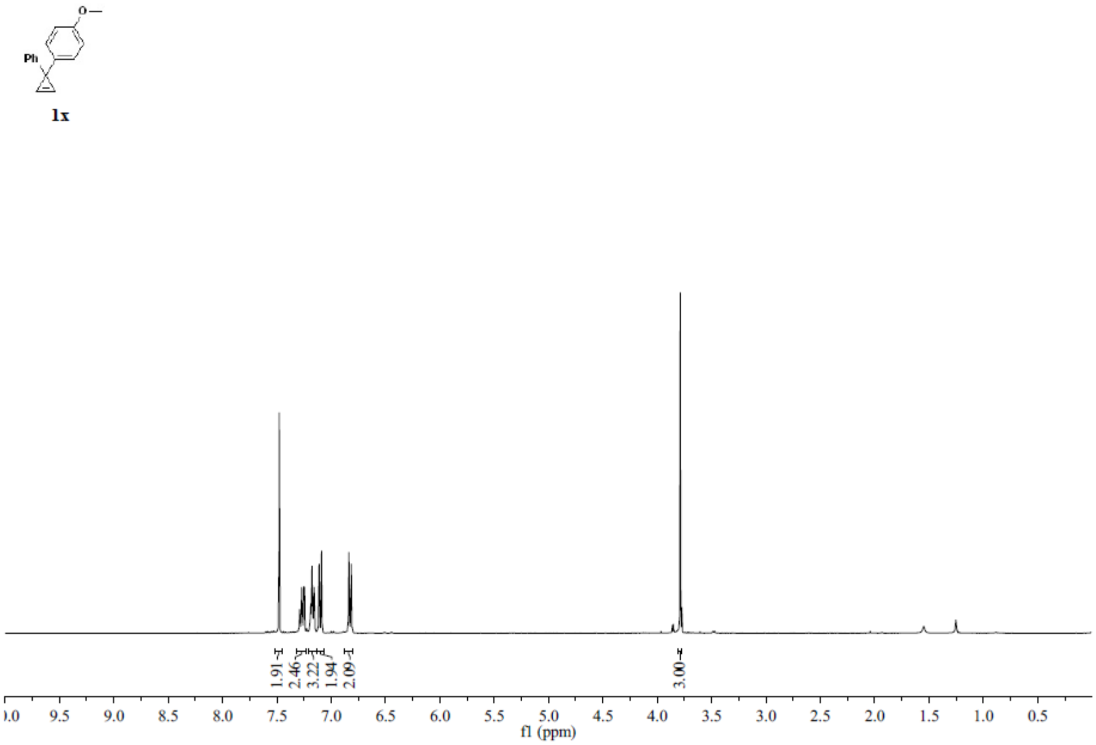

Supplementary Figure 215. ^{1}H-NMR of compound **1x**, recorded at 400 MHz and 25 °C in $CDCl_3$.

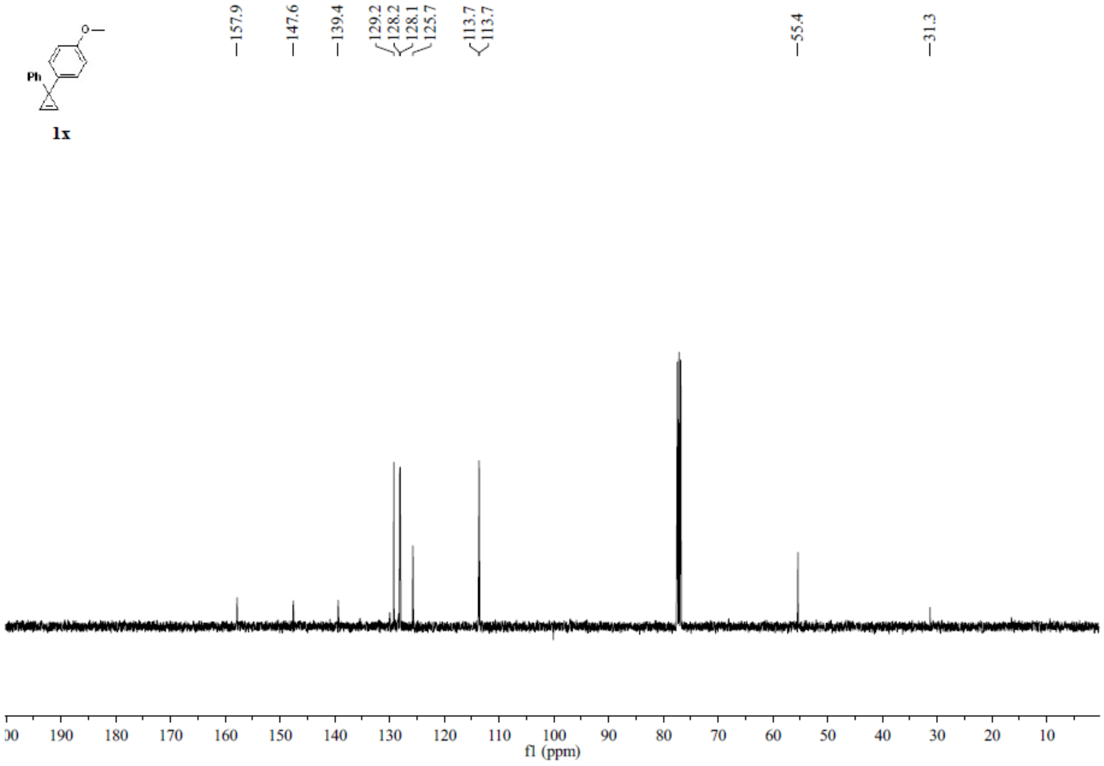

Supplementary Figure 216. ^{13}C-NMR of compound **1x**, recorded at 100 MHz and 25 °C in $CDCl_3$.

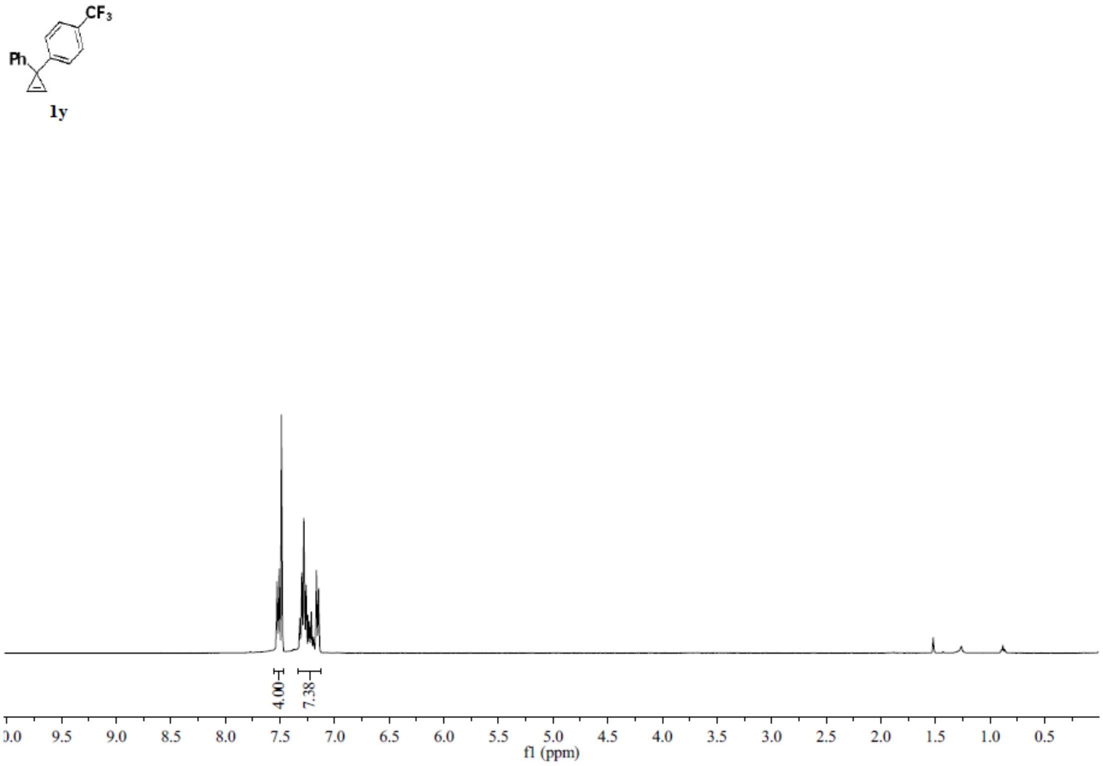

Supplementary Figure 217. ^{1}H-NMR of compound **1y**, recorded at 400 MHz and 25 °C in $CDCl_3$.

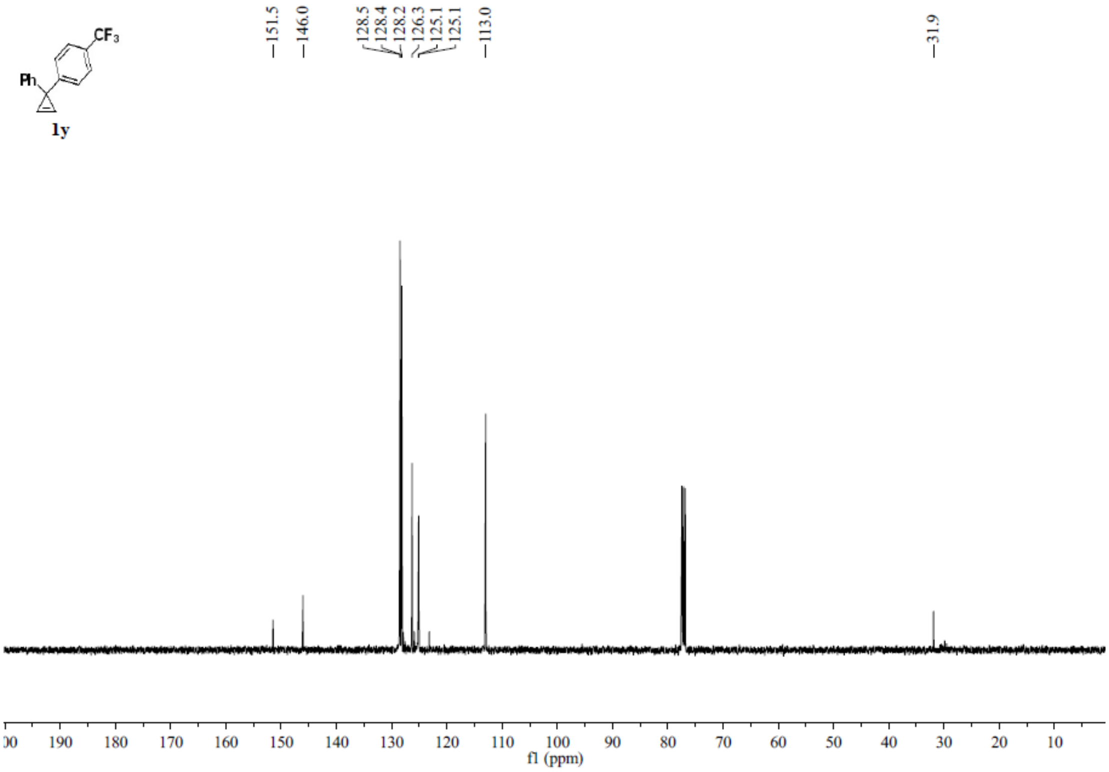

Supplementary Figure 218. ^{13}C-NMR of compound **1y**, recorded at 100 MHz and 25 °C in $CDCl_3$.

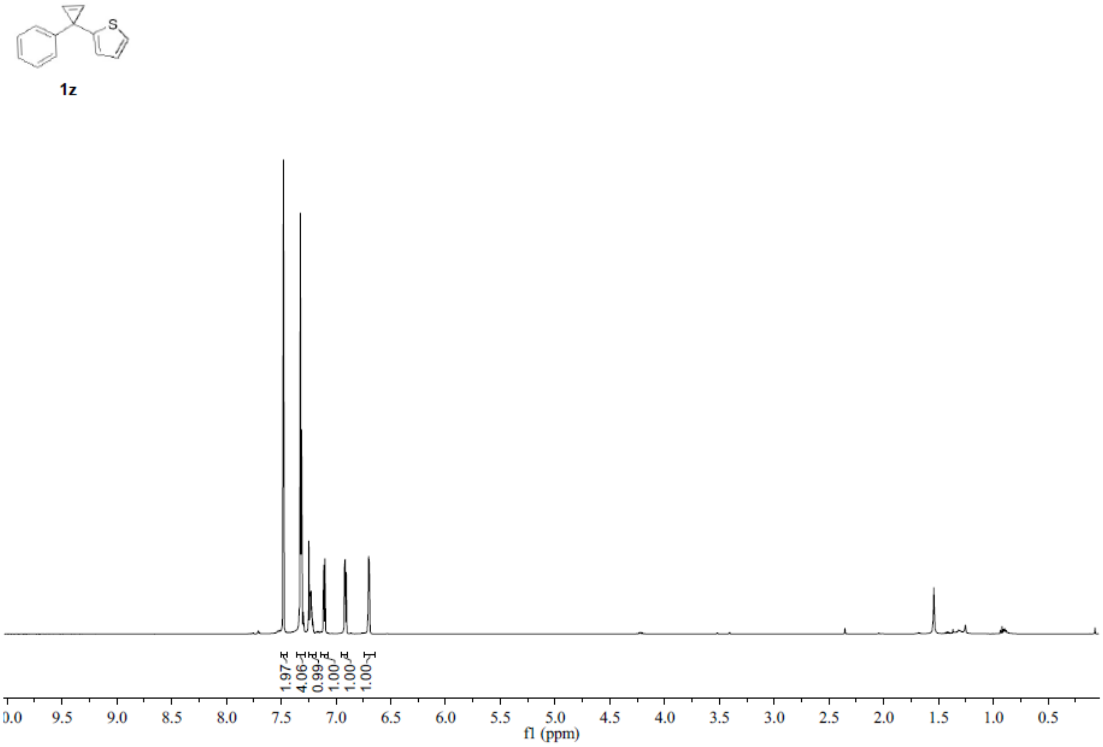

Supplementary Figure 219. ^{1}H-NMR of compound **1z**, recorded at 500 MHz and 25 °C in $CDCl_3$.

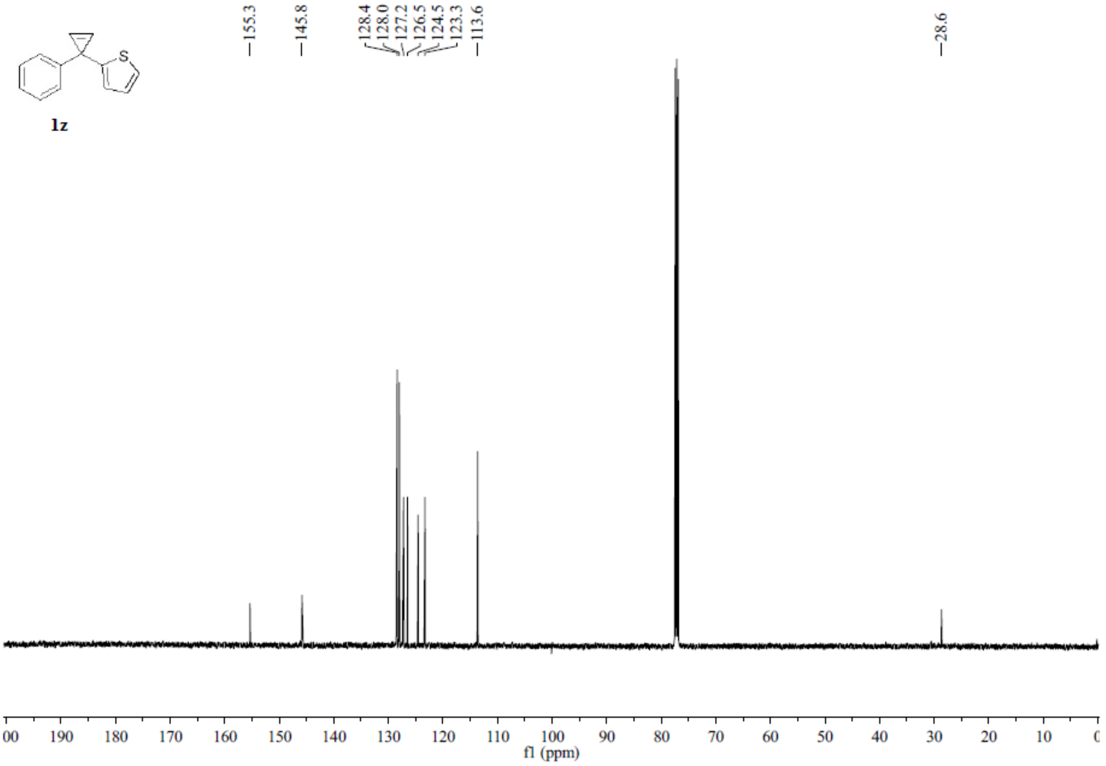

Supplementary Figure 220. ^{13}C-NMR of compound **1z**, recorded at 125 MHz and 25 °C in $CDCl_3$.

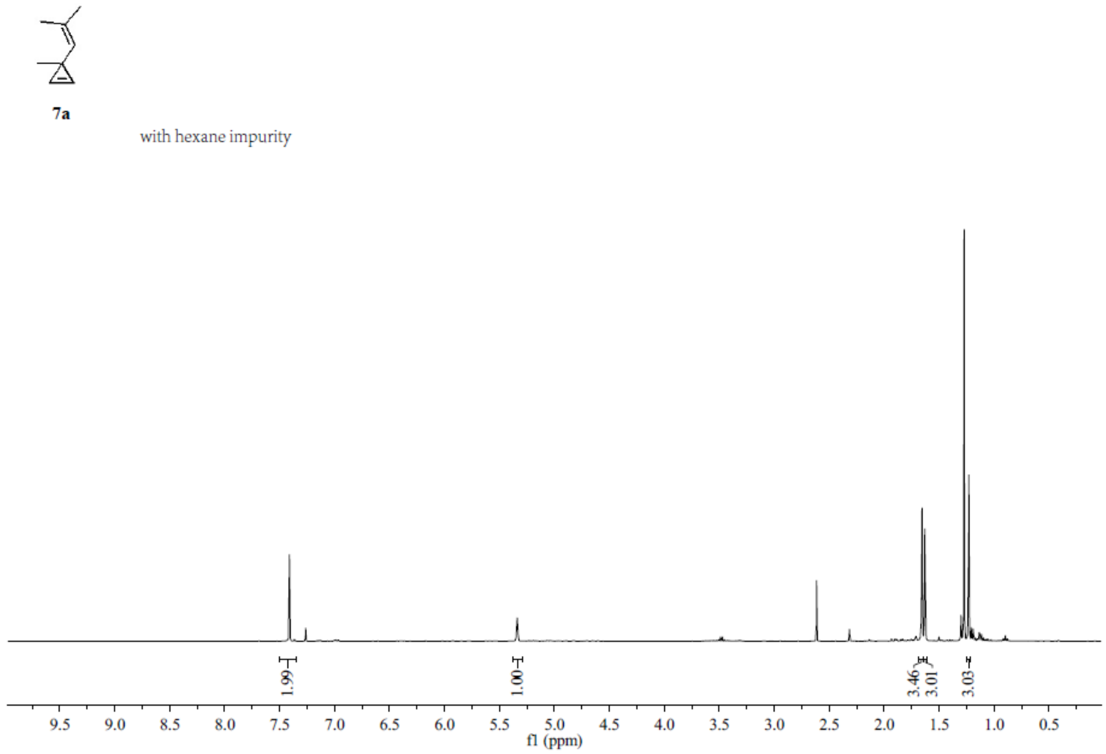

Supplementary Figure 221. ^{1}H-NMR of compound **7a**, recorded at 400 MHz and 25 °C in $CDCl_3$.

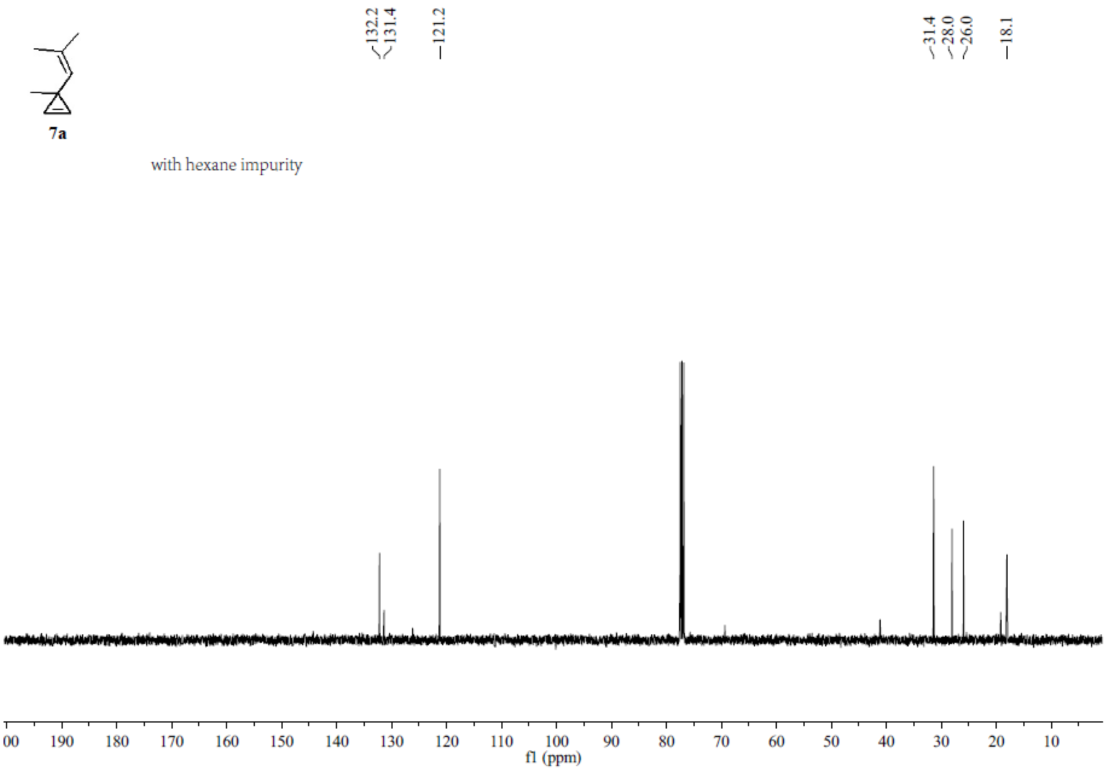

Supplementary Figure 222. ^{13}C-NMR of compound **7a**, recorded at 100 MHz and 25 °C in $CDCl_3$.

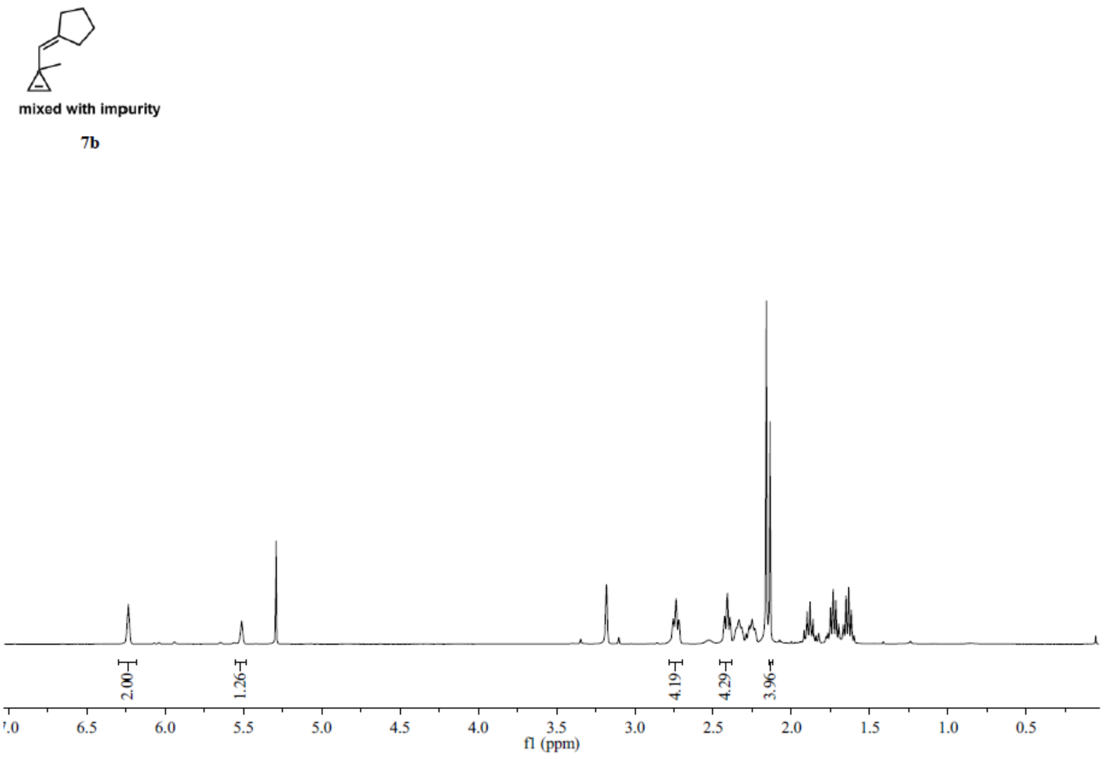

Supplementary Figure 223. ^{1}H-NMR of compound **7b**, recorded at 400 MHz and 25 °C in $CDCl_3$.

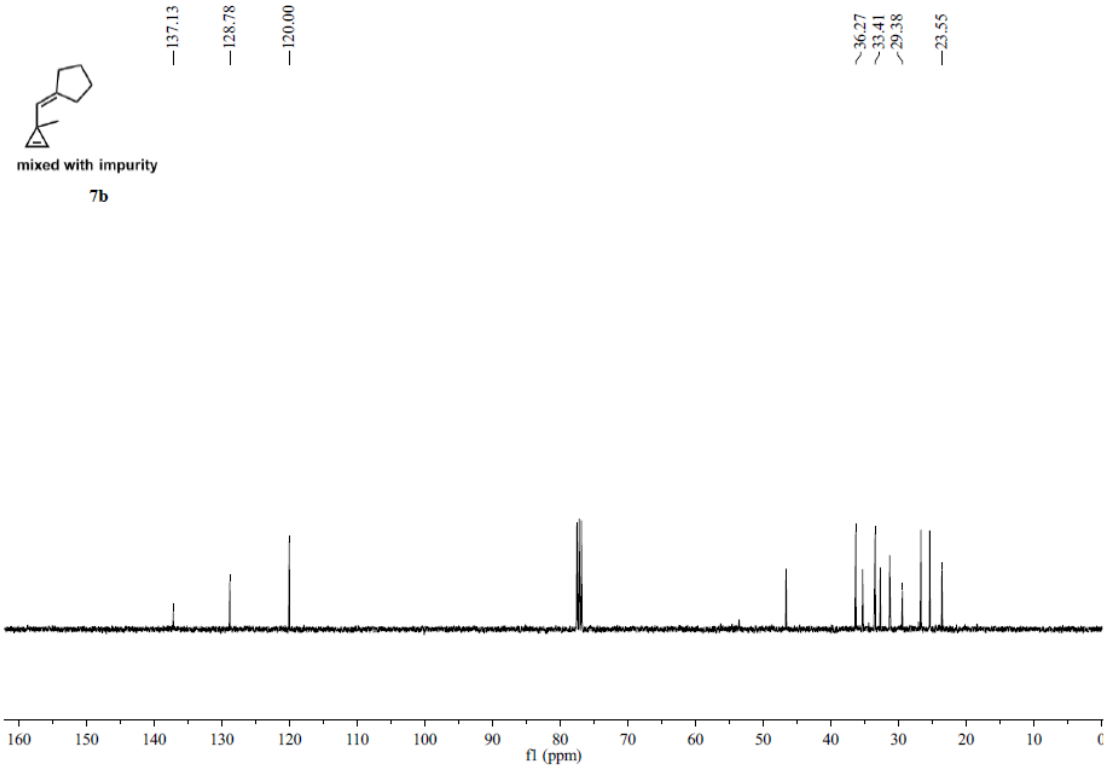

Supplementary Figure 224. ^{13}C-NMR of compound **7b**, recorded at 100 MHz and 25 °C in $CDCl_3$.

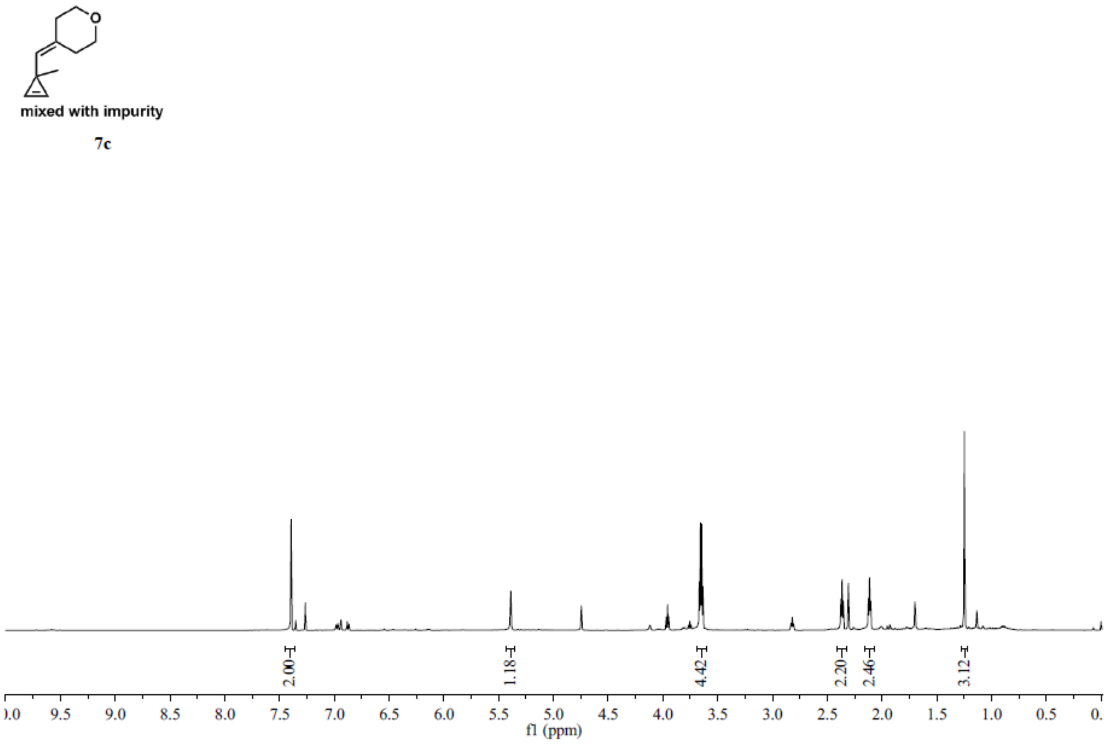

Supplementary Figure 225. ^{1}H-NMR of compound **7c**, recorded at 500 MHz and 25 °C in $CDCl_3$.

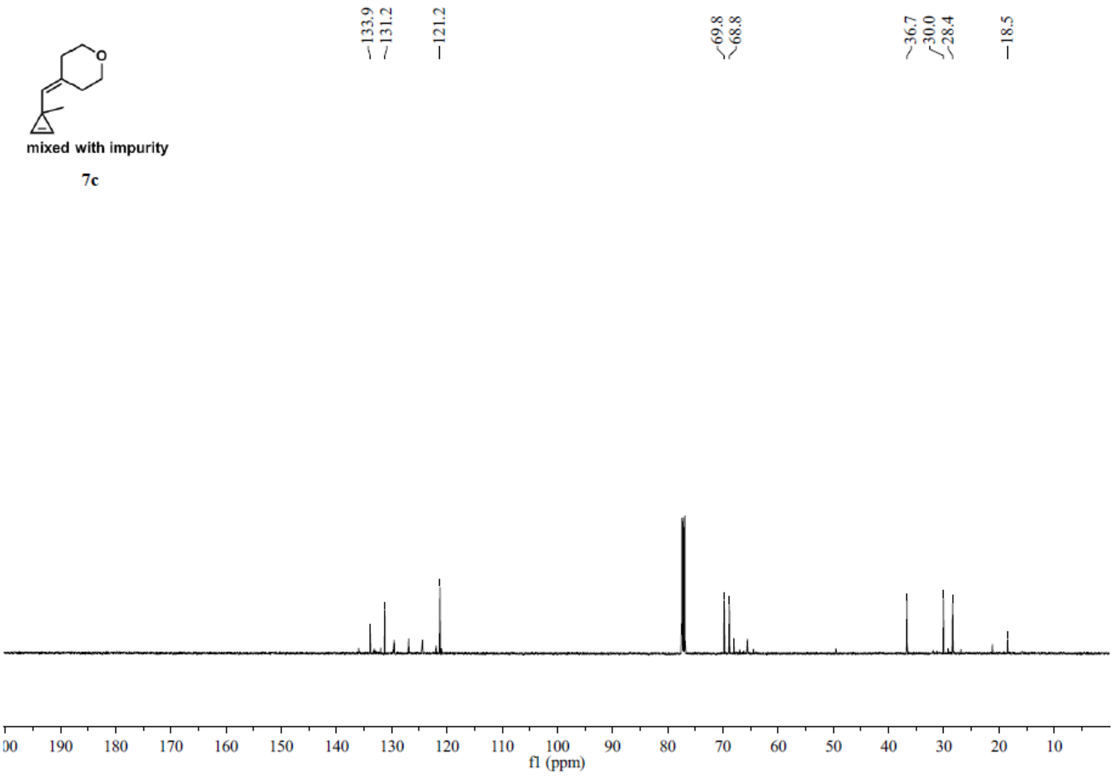

Supplementary Figure 226. ^{13}C-NMR of compound **7c**, recorded at 125 MHz and 25 °C in $CDCl_3$.

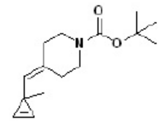

7d

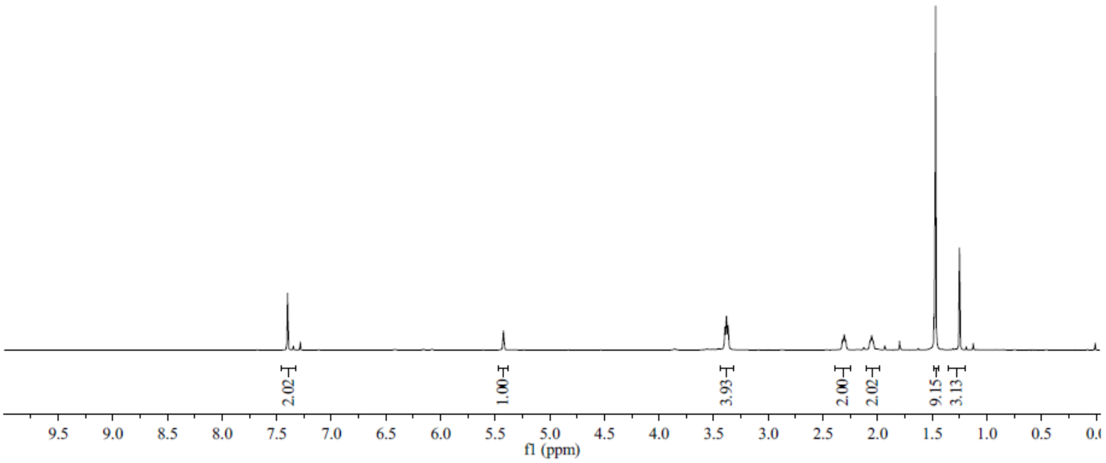

Supplementary Figure 227. ^{1}H-NMR of compound **7d**, recorded at 400 MHz and 25 °C in $CDCl_3$.

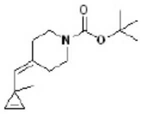

7d

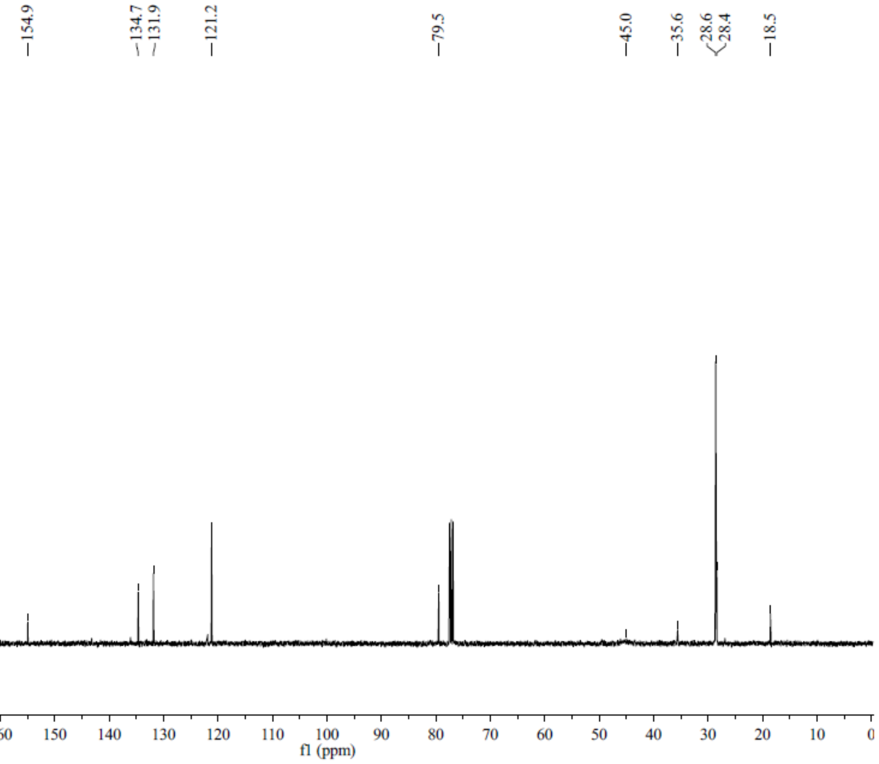

Supplementary Figure 228. ^{13}C-NMR of compound **7d**, recorded at 100 MHz and 25 °C in $CDCl_3$.

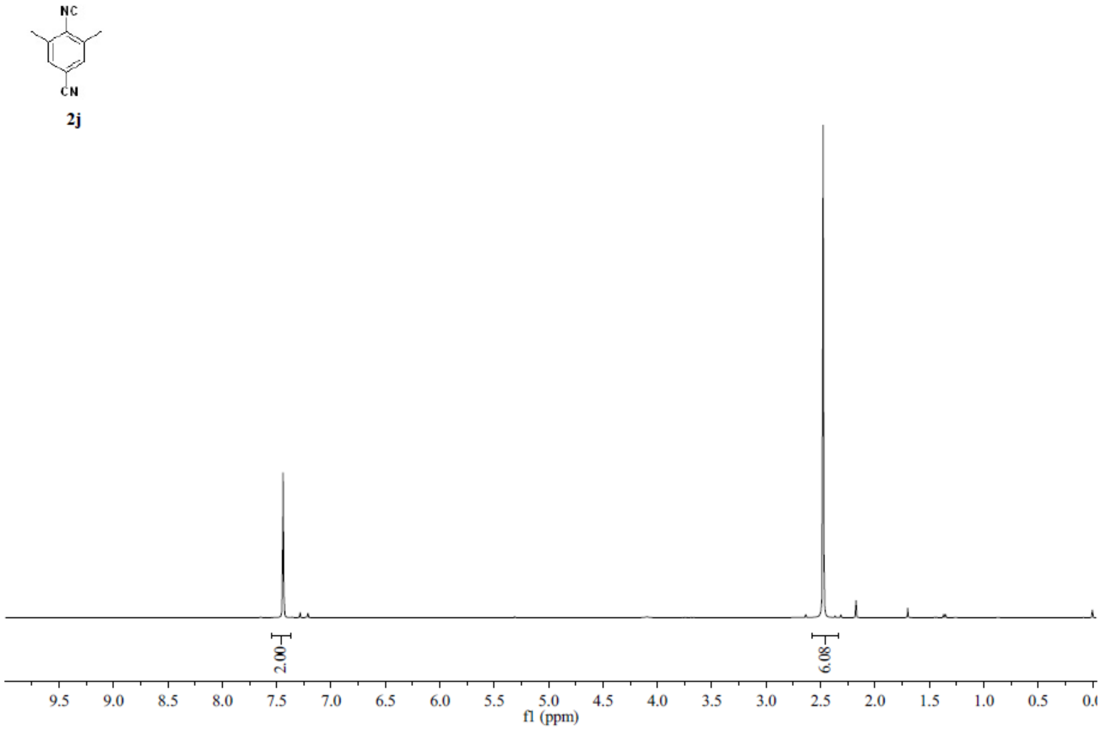

Supplementary Figure 229. ^{1}H-NMR of compound **2j**, recorded at 400 MHz and 25 °C in $CDCl_3$.

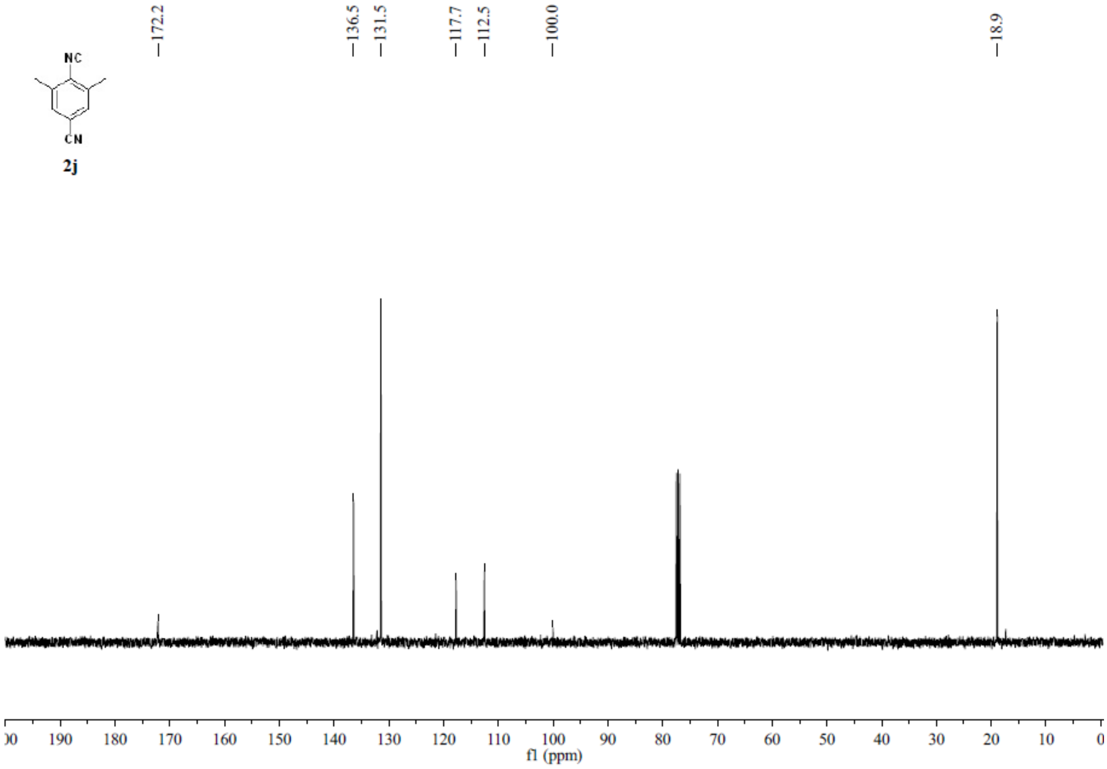

Supplementary Figure 230. ^{13}C-NMR of compound **2j**, recorded at 100 MHz and 25 °C in $CDCl_3$.

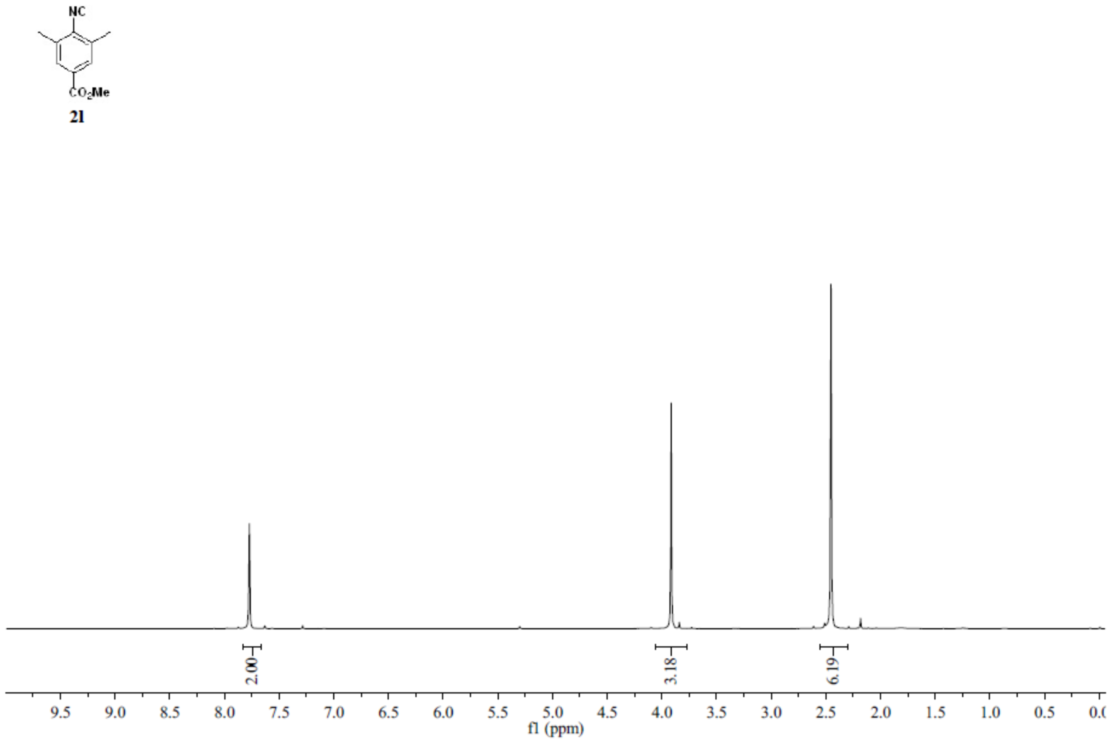

Supplementary Figure 231. ^{1}H-NMR of compound **2l**, recorded at 400 MHz and 25 °C in $CDCl_3$.

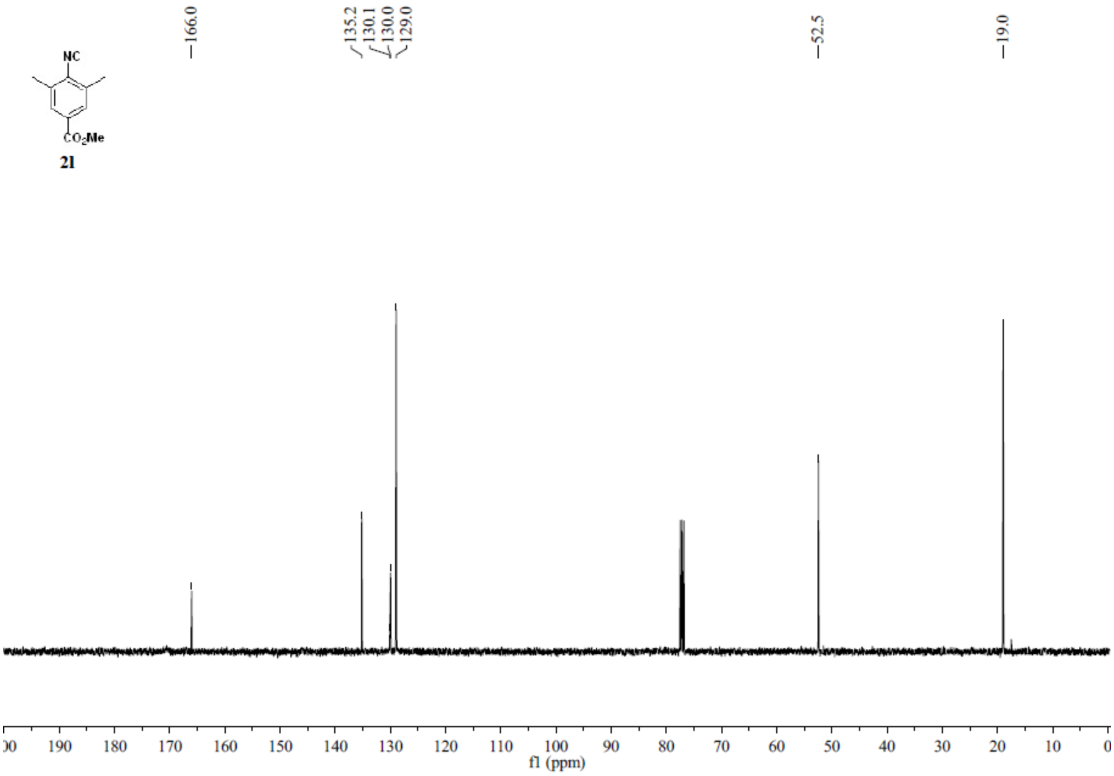

Supplementary Figure 232. ^{13}C-NMR of compound **2l**, recorded at 100 MHz and 25 °C in $CDCl_3$.

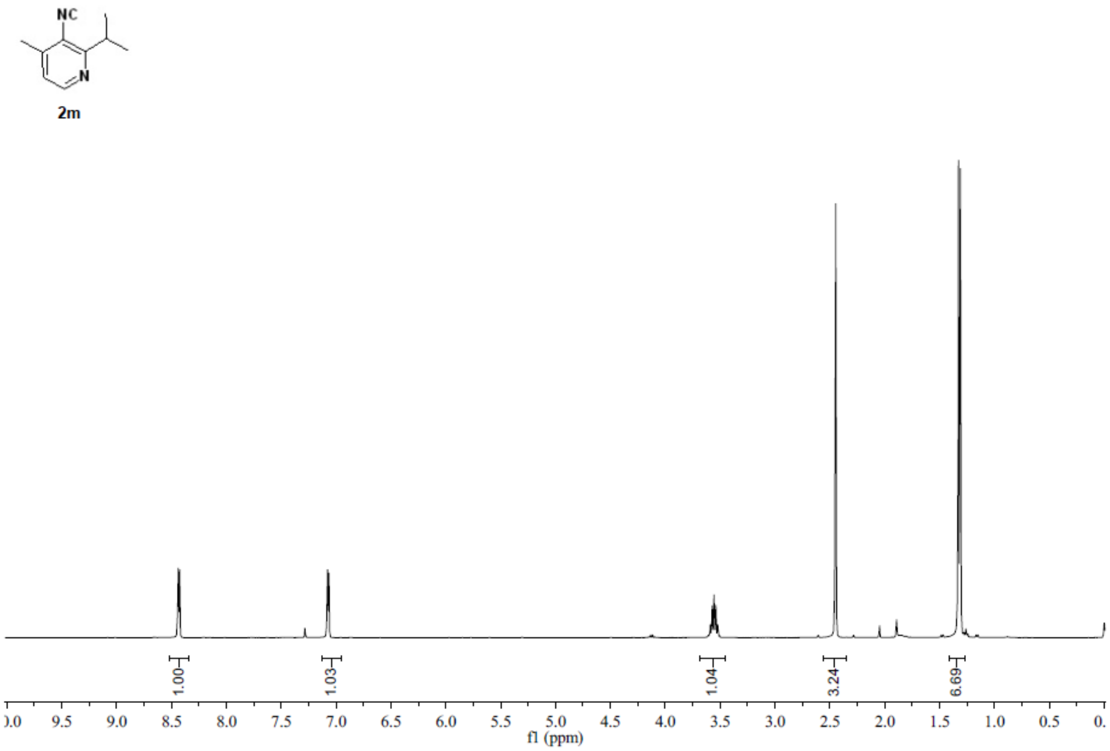

Supplementary Figure 233. ^{1}H-NMR of compound **2m**, recorded at 400 MHz and 25 °C in $CDCl_3$.

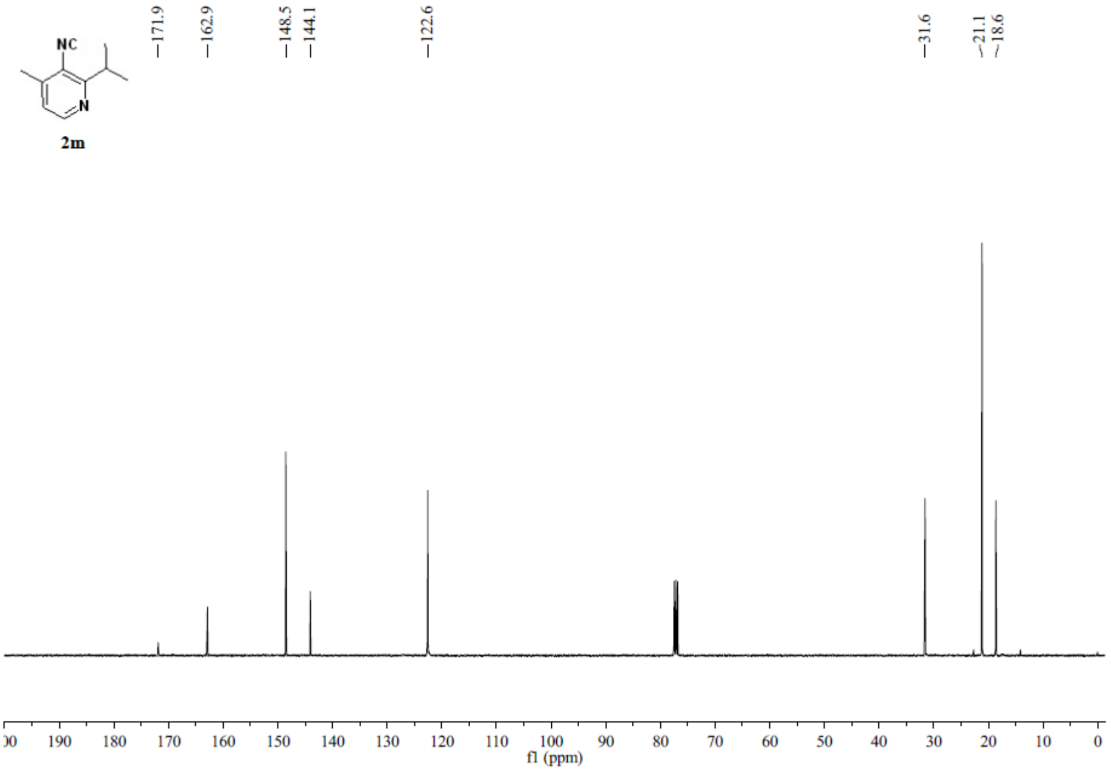

Supplementary Figure 234. ^{13}C-NMR of compound **2m**, recorded at 100 MHz and 25 °C in $CDCl_3$.

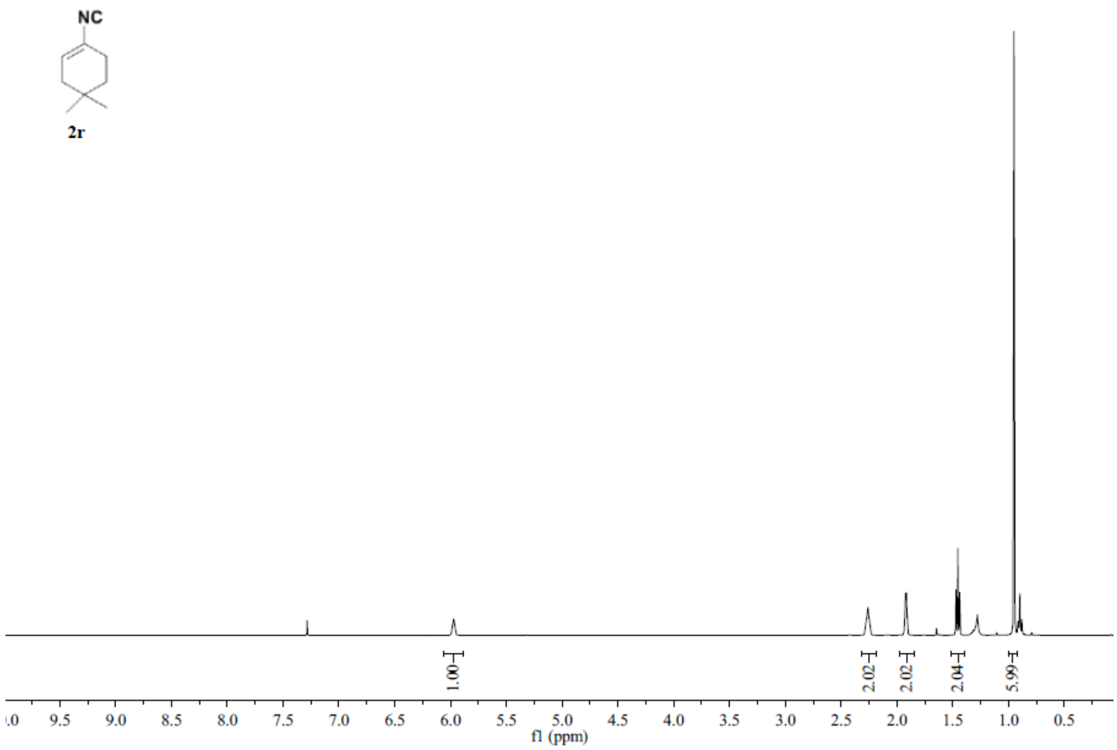

Supplementary Figure 235. ^{1}H-NMR of compound **2r**, recorded at 400 MHz and 25 °C in $CDCl_3$.

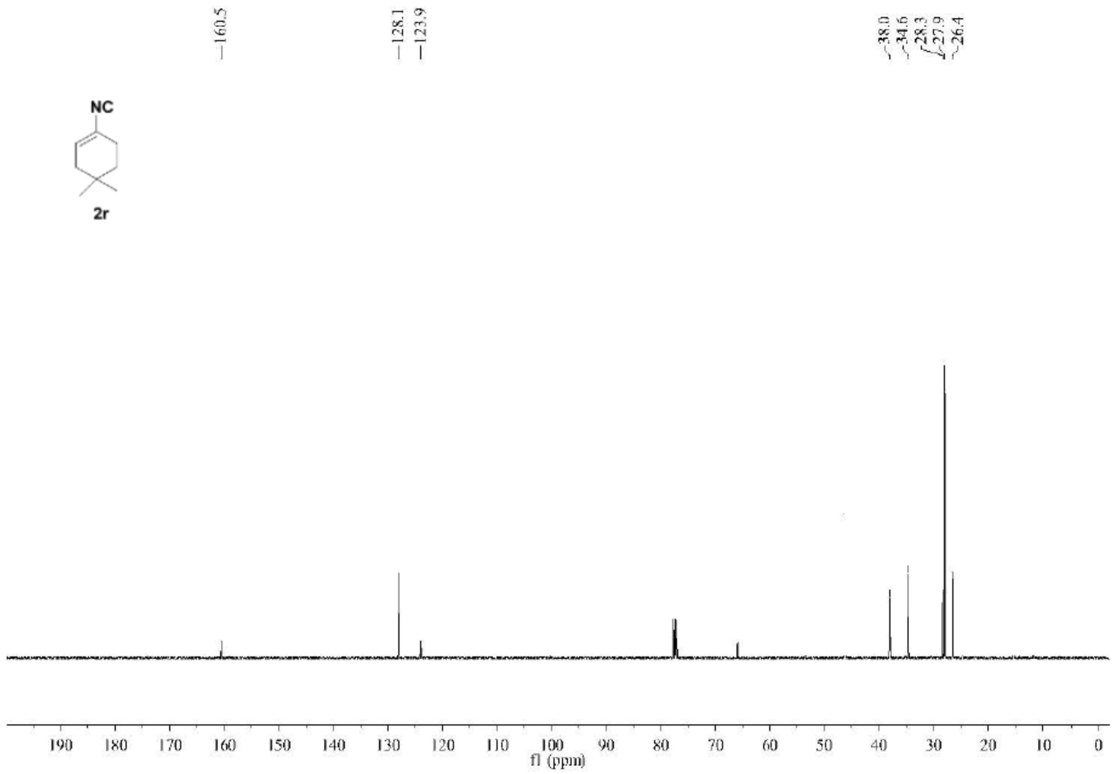

Supplementary Figure 236. ^{13}C-NMR of compound **2r**, recorded at 100 MHz and 25 °C in $CDCl_3$.

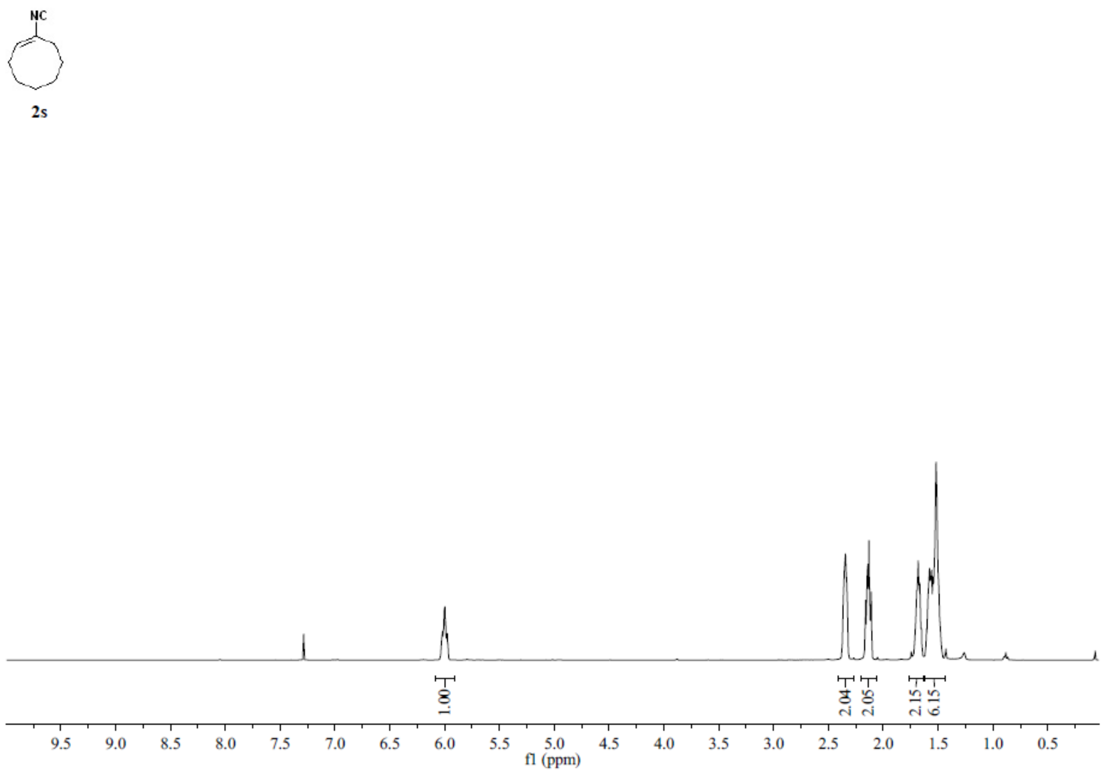

Supplementary Figure 237. ^{1}H-NMR of compound **2s**, recorded at 400 MHz and 25 °C in $CDCl_3$.

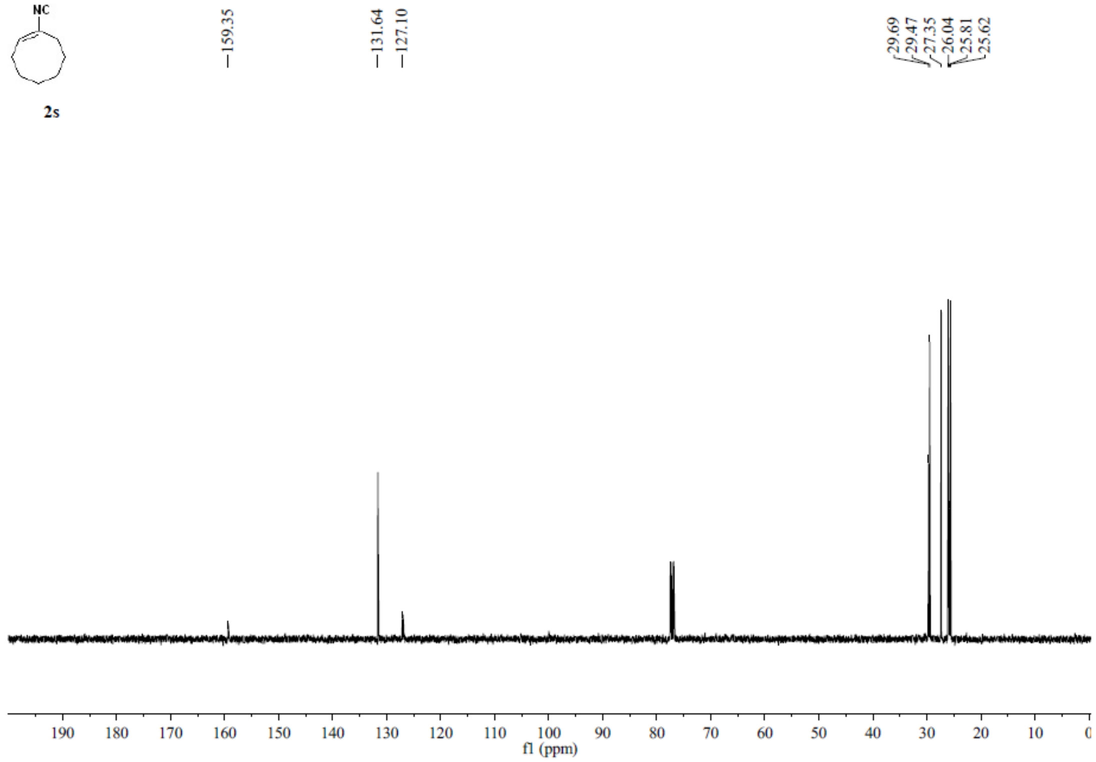

Supplementary Figure 238. ^{13}C-NMR of compound **2s**, recorded at 100 MHz and 25 °C in $CDCl_3$.

4. Supplementary References

1. Sherrill, W. M.; Kim, R.; Rubin, M., Improved preparative route toward 3-arylcyclopropenes. *Tetrahedron* **2008,** *64* (37), 8610-8617.
2. Edwards, A.; Rubina, M.; Rubin, M., Directed Rh-I-Catalyzed Asymmetric Hydroboration of Prochiral 1-Arylcycloprop-2-Ene-1-Carboxylic Acid Derivatives. *Chem-Eur J* **2018,** *24* (6), 1394-1403.
3. Parra, A.; Amenós, L.; Guisán-Ceinos, M.; López, A.; García Ruano, J. L.; Tortosa, M., Copper-Catalyzed Diastereo- and Enantioselective Desymmetrization of Cyclopropenes: Synthesis of Cyclopropylboronates. *J. Am. Chem. Soc.* **2014,** *136* (45), 15833-15836.
4. Zhang, L.; Oestreich, M., Copper-Catalyzed Enantio- and Diastereoselective Addition of Silicon Nucleophiles to 3,3-Disubstituted Cyclopropenes. **2019,** *25* (63), 14304-14307.
5. Rubina, M.; Rubin, M.; Gevorgyan, V., Transition Metal-Catalyzed Hydro-, Sila-, and Stannastannation of Cyclopropenes: Stereo- and Regioselective Approach toward Multisubstituted Cyclopropyl Synthons. *J. Am. Chem. Soc.* **2002,** *124* (39), 11566-11567.
6. Phan, D. H. T.; Kou, K. G. M.; Dong, V. M., Enantioselective Desymmetrization of Cyclopropenes by Hydroacylation. *J. Am. Chem. Soc.* **2010,** *132* (46), 16354-16355.
7. Shintani, R.; Iino, R.; Nozaki, K., Rhodium-catalyzed polymerization of 3,3-diarylcyclopropenes involving a 1,4-rhodium migration. *J Am Chem Soc* **2014,** *136* (22), 7849-52.
8. Wennekes, T.; Bonger, K. M.; Vogel, K.; van den Berg, R. J. B. H. N.; Strijland, A.; Donker-Koopman, W. E.; Aerts, J. M. F. G.; van der Marel, G. A.; Overkleeft, H. S., The Development of an Aza-C-Glycoside Library Based on a Tandem Staudinger/Aza-Wittig/Ugi Three-Component Reaction. *European Journal of Organic Chemistry* **2012,** *2012* (32), 6420-6454.
9. Schultz, E. E.; Pujanauski, B. G.; Sarpong, R., Synthetic Studies toward Lapidilectine-Type Kopsia Alkaloids. *Org. Lett.* **2012,** *14* (2), 648-651.
10. Tobisu, M.; Kitajima, A.; Yoshioka, S.; Hyodo, I.; Oshita, M.; Chatani, N., Brønsted Acid Catalyzed Formal Insertion of Isocyanides into a C−O Bond of Acetals. *J. Am. Chem. Soc.* **2007,** *129* (37), 11431-11437.
11. Guirado, A.; Zapata, A.; Gómez, J. L.; Trabalón, L.; Gálvez, J., Electrochemical generation of alkyl and aryl isocyanides. *Tetrahedron* **1999,** *55* (31), 9631-9640.
12. Blanco Jaimes, M. C.; Böhling, C. R. N.; Serrano-Becerra, J. M.; Hashmi, A. S. K., Highly Active Mononuclear NAC–Gold(I) Catalysts. **2013,** *52* (31), 7963-7966.
13. Zheng, Q.; Boltjes, A.; Dömling, A., An Ugi Reaction/Intramolecular Cyclization/Oxidation Cascade towards Tetrazole-Linked Dibenzoxazepines. *Synthesis* **2021,** *53* (11), 1980-1988.
14. Wang, Y.; Wang, H.; Peng, J.; Zhu, Q., Palladium-Catalyzed Intramolecular C(sp2)–H Amidination by Isonitrile Insertion Provides Direct Access to 4-Aminoquinazolines from N-Arylamidines. *Org. Lett.* **2011,** *13* (17), 4604-4607.
15. Braunschweig, H.; Radacki, K.; Shang, R.; Tate, C. W., Reversible Intramolecular Coupling of the Terminal Borylene and a Carbonyl Ligand of [Cp(CO)2Mn□B□tBu]. **2013,** *52* (2), 729-733.
16. Tran, C. C.; Kawaguchi, S.-i.; Kobiki, Y.; Matsubara, H.; Tran, D. P.; Kodama, S.; Nomoto, A.; Ogawa, A., Palladium-Catalyzed Diarylation of Isocyanides with Tetraarylleads for the Selective Synthesis of Imines and α-Diimines. *The Journal of Organic Chemistry* **2019,** *84*

(18), 11741-11751.
17. Ko, C.-C.; Lo, L. T.-L.; Ng, C.-O.; Yiu, S.-M., Photochemical Synthesis of Intensely Luminescent Isocyano Rhenium(I) Complexes with Readily Tunable Structural Features. **2010,** *16* (46), 13773-13782.
18. Szymański, W.; Velema, W. A.; Feringa, B. L., Photocaging of Carboxylic Acids: A Modular Approach. *Angew. Chem. Int. Ed.* **2014,** *53* (33), 8682-8686.
19. Cao, Q.; Howard, J. L.; Wheatley, E.; Browne, D. L., Mechanochemical Activation of Zinc and Application to Negishi Cross-Coupling. *Angew. Chem. Int. Ed.* **2018,** *57* (35), 11339-11343.
20. Ho, C. Y.; Huang, j.-q., NHC-Ni(II)-Catalyzed [3+2] Cross-Dimerization of Unactivated Olefin and Methylenecyclopropane. *Angew. Chem. Int. Ed.* **2020**.
21. Meiries, S.; Le Duc, G.; Chartoire, A.; Collado, A.; Speck, K.; Arachchige, K. S. A.; Slawin, A. M. Z.; Nolan, S. P., Large yet Flexible N-Heterocyclic Carbene Ligands for Palladium Catalysis. *Chemistry – A European Journal* **2013,** *19* (51), 17358-17368.
22. Nett, A. J.; Cañellas, S.; Higuchi, Y.; Robo, M. T.; Kochkodan, J. M.; Haynes, M. T.; Kampf, J. W.; Montgomery, J., Stable, Well-Defined Nickel(0) Catalysts for Catalytic C–C and C–N Bond Formation. *Acs Catal* **2018,** *8* (7), 6606-6611.
23. Beattie, D. D.; Lascoumettes, G.; Kennepohl, P.; Love, J. A.; Schafer, L. L., Disproportionation Reactions of an Organometallic Ni(I) Amidate Complex: Scope and Mechanistic Investigations. *Organometallics* **2018,** *37* (9), 1392-1399.
24. Terao, J.; Watanabe, H.; Ikumi, A.; Kuniyasu, H.; Kambe, N., Nickel-Catalyzed Cross-Coupling Reaction of Grignard Reagents with Alkyl Halides and Tosylates: Remarkable Effect of 1,3-Butadienes. *J. Am. Chem. Soc.* **2002,** *124* (16), 4222-4223.
25. Iwasaki, T.; Min, X.; Fukuoka, A.; Zhu, L.; Qiu, R.; Yang, T.; Ehara, M.; Sudalai, A.; Kambe, N., Ni-Catalyzed Dimerization and Hydroperfluoroarylation of 1,3-Dienes. *The Journal of Organic Chemistry* **2018,** *83* (16), 9267-9277.
26. Qiu, G.; Mamboury, M.; Wang, Q.; Zhu, J., Ketenimines from Isocyanides and Allyl Carbonates: Palladium-Catalyzed Synthesis of beta,gamma-Unsaturated Amides and Tetrazoles. *Angew. Chem. Int. Ed. Engl.* **2016,** *55* (49), 15377-15381.
27. Baird, R. L.; Weigert, F. J.; Shapley, J. R., Palladium chloride catalyzed cyclodimerization of 1-methylcyclopropene. *J. Am. Chem. Soc.* **1970,** *92* (22), 6630-6635.
28. Kapat, A.; Sperger, T.; Guven, S.; Schoenebeck, F., E-Olefins through intramolecular radical relocation. *Science* **2019,** *363* (6425), 391-+.
